# Supplementary material for: Design and Synthesis of 1,3-Diarylpyrazoles and Investigation of Their Cytotoxicity and Antiparasitic Profile
Source: Int J Mol Sci. 2024 Apr 25;25(9):4693. doi: 10.3390/ijms25094693 (PMC11487390; doi:10.3390/ijms25094693)

Supplementary Materials

Design and Synthesis of 1,3-Diarylpyrazoles and Investigation of Their Cytotoxicity and Antiparasitic Profile

Murat Bozdag [1], Freke Mertens [2], An Matheeussen [3], Natascha Van Pelt [3], Kenn Foubert [4], Nina Hermans [4], Guido R. Y. De Meyer [2], Koen Augustyns [1], Wim Martinet [2], Guy Caljon [3] and Pieter Van der Veken 1,*

1 Laboratory of Medicinal Chemistry, Department of Pharmaceutical Sciences, Infla-Med Centre of Excellence, University of Antwerp, Universiteitsplein 1, 2610 Wilrijk-Antwerp, Belgium; koen.augustyns@uantwerpen.be (K.A.)

2 L aboratory of Physiopharmacology, Department of Pharmaceutical Sciences, Infla-Med Centre of Excellence, University of Antwerp, Universiteitsplein 1, 2610 Wilrijk-Antwerp, Belgium; freke.mertens@uantwerpen.be (F.M.); guido.demeyer@uantwerpen.be (G.R.Y.D.M.); wim.martinet@uantwerpen.be (W.M.)

3 Laboratory of Microbiology, Parasitology and Hygiene, Department of Biomedical Sciences, Infla-Med Centre of Excellence, University of Antwerp, Universiteitsplein 1, 2610 Wilrijk-Antwerp, Belgium; an.matheeussen@uantwerpen.be (A.M.); natascha.vanpelt@uantwerpen.be (N.V.P.); guy.caljon@uantwerpen.be (G.C.)

4 Natural Products and Food Research and Analysis–Pharmaceutical Technology, Department of Pharmaceutical Sciences, University of Antwerp, Universiteitsplein 1, 2610 Wilrijk-Antwerp, Belgium; kenn.foubert@uantwerpen.be (K.F.); nina.hermans@uantwerpen.be (N.H.)

* Correspondence: pieter.vanderveken@uantwerpen.be

Table of Contents:

1) ^{1}H and ^{13}C-NMR spectra of compounds 13–69……………………………..…...2–115
2) UPLC and HRMS spectra of the studied samples…………………………..….116–147

1. **^{1}H and ^{13}C-NMR spectra of compounds 13–69**

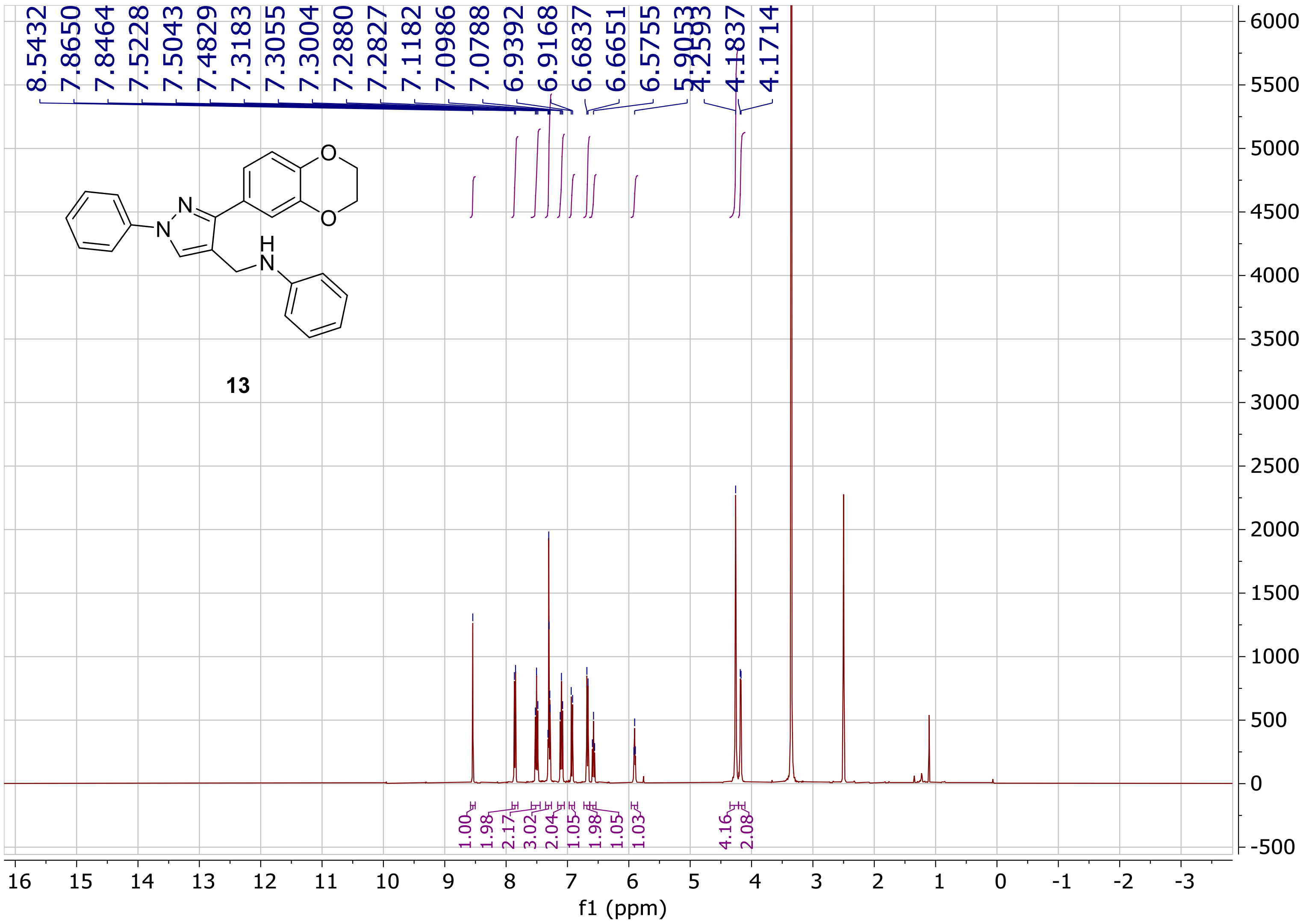

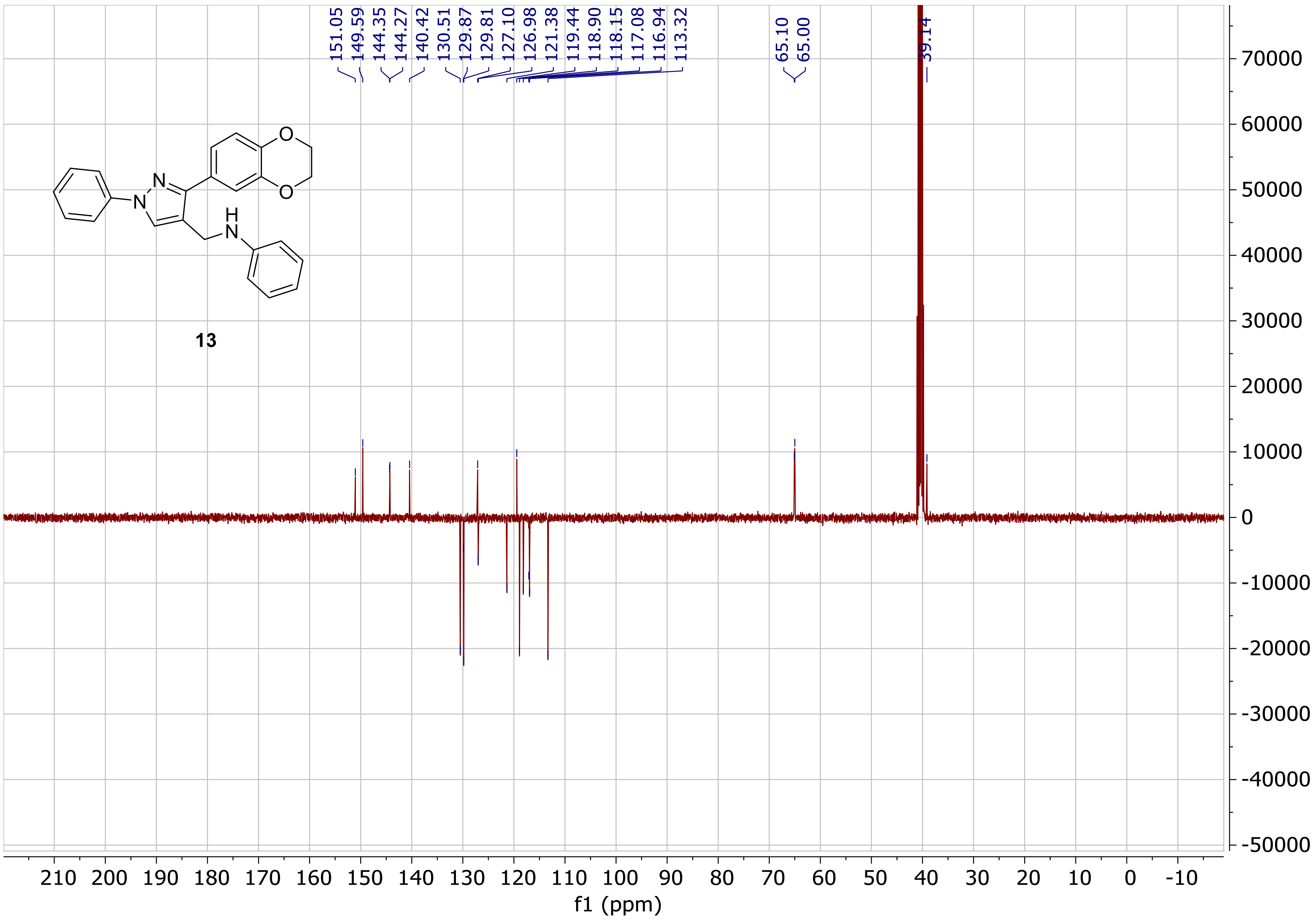
151.05
149.59
144.35
144.27
140.42
130.51
129.87
129.81
127.10
126.98
121.38
119.44
118.90
118.15
117.08
116.94
113.32
65.10
65.00
39.14
N
N
O
O
H
N
13
70000
60000
50000
40000
30000
20000
10000
0
-10000
-20000
-30000
-40000
-50000
210 200 190 180 170 160 150 140 130 120 110 100 90 80 70 60 50 40 30 20 10 0 -10
f1 (ppm)

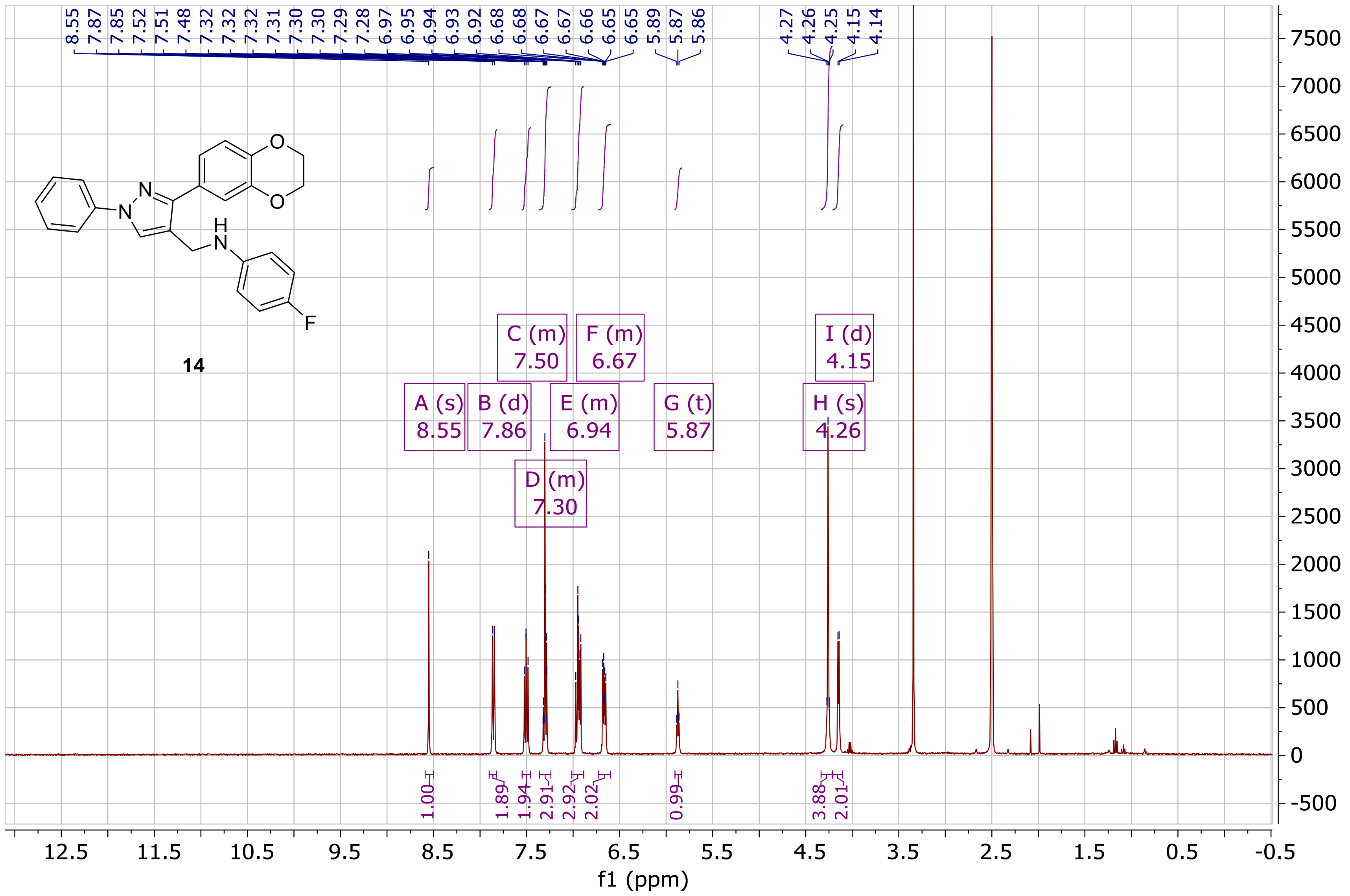
8.55 7.87 7.85 7.52 7.51 7.48 7.32 7.32 7.32 7.31 7.30 7.30 7.29 7.28 6.97 6.95 6.94 6.93 6.92 6.68 6.68 6.67 6.67 6.66 6.65 6.65 5.89 5.87 5.86
4.27 4.26 4.25 4.15 4.14
N
N
O
O
H
N
F
14
A (s) 8.55
B (d) 7.86
C (m) 7.50
D (m) 7.30
E (m) 6.94
F (m) 6.67
G (t) 5.87
H (s) 4.26
I (d) 4.15
1.00
1.89
1.94
2.91
2.92
2.02
0.99
3.88
2.01
12.5 11.5 10.5 9.5 8.5 7.5 6.5 5.5 4.5 3.5 2.5 1.5 0.5 -0.5
f1 (ppm)
7500 7000 6500 6000 5500 5000 4500 4000 3500 3000 2500 2000 1500 1000 500 0 -500

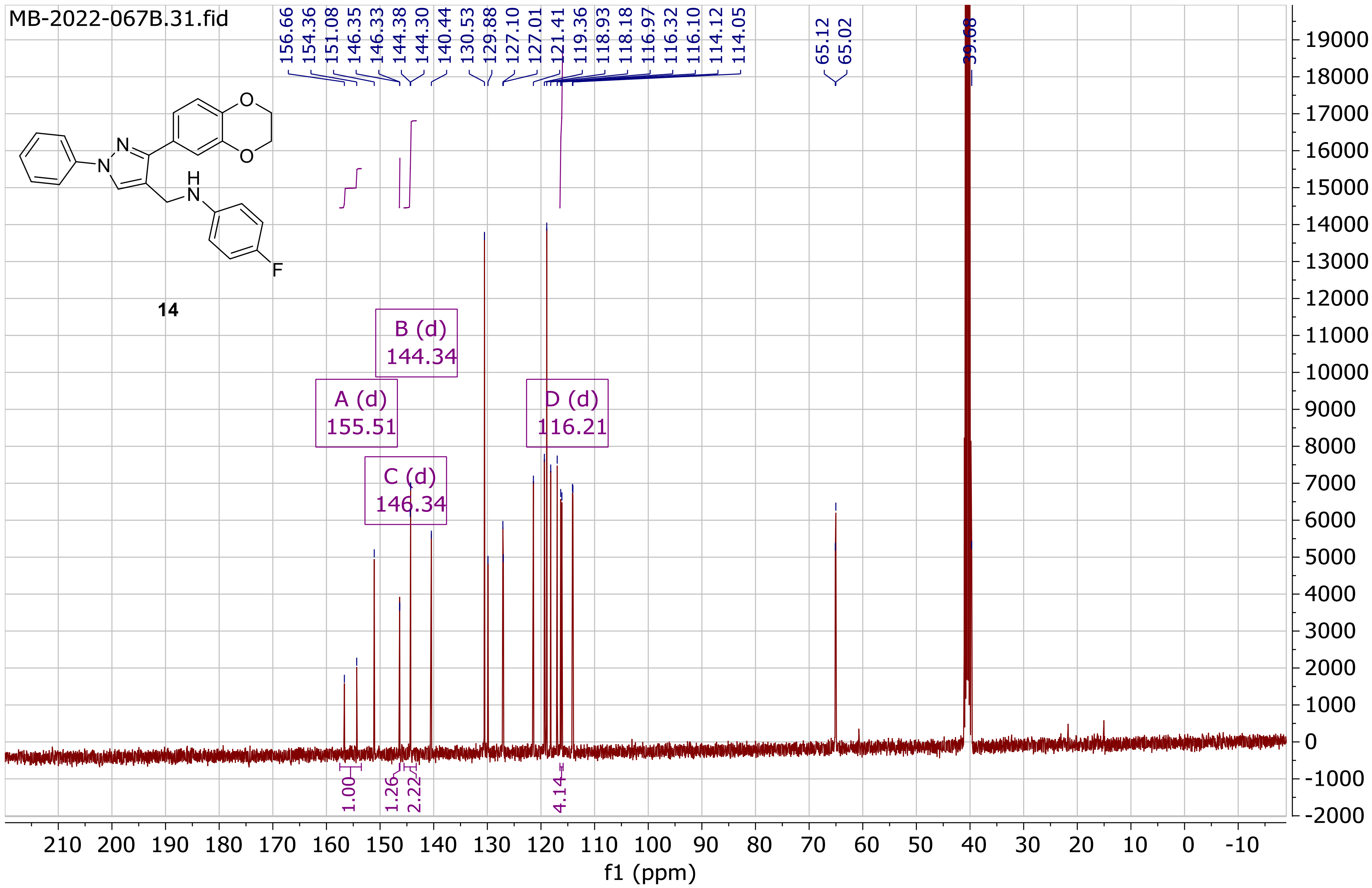
MB-2022-067B.31.fid
156.66
154.36
151.08
146.35
146.33
144.38
144.30
140.44
130.53
129.88
127.10
127.01
121.41
119.36
118.93
118.18
116.97
116.32
116.10
114.12
114.05
65.12
65.02
39.68
14
A (d)
155.51
B (d)
144.34
C (d)
146.34
D (d)
116.21
1.00
1.26
2.22
4.14
f1 (ppm)

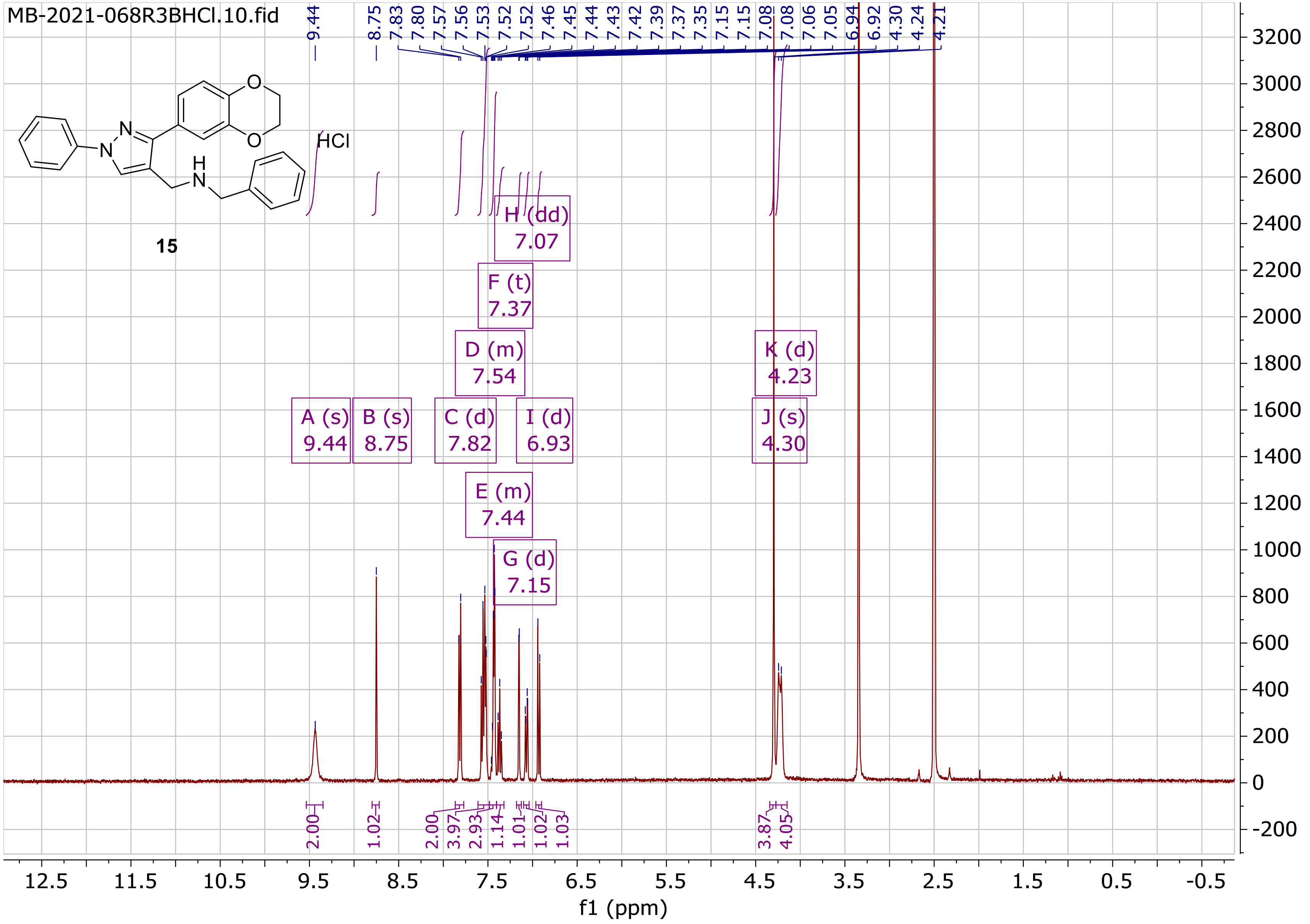
MB-2021-068R3BHCl.10.fid
HCl
15
A (s) 9.44
B (s) 8.75
C (d) 7.82
D (m) 7.54
E (m) 7.44
F (t) 7.37
G (d) 7.15
H (dd) 7.07
I (d) 6.93
J (s) 4.30
K (d) 4.23
f1 (ppm)

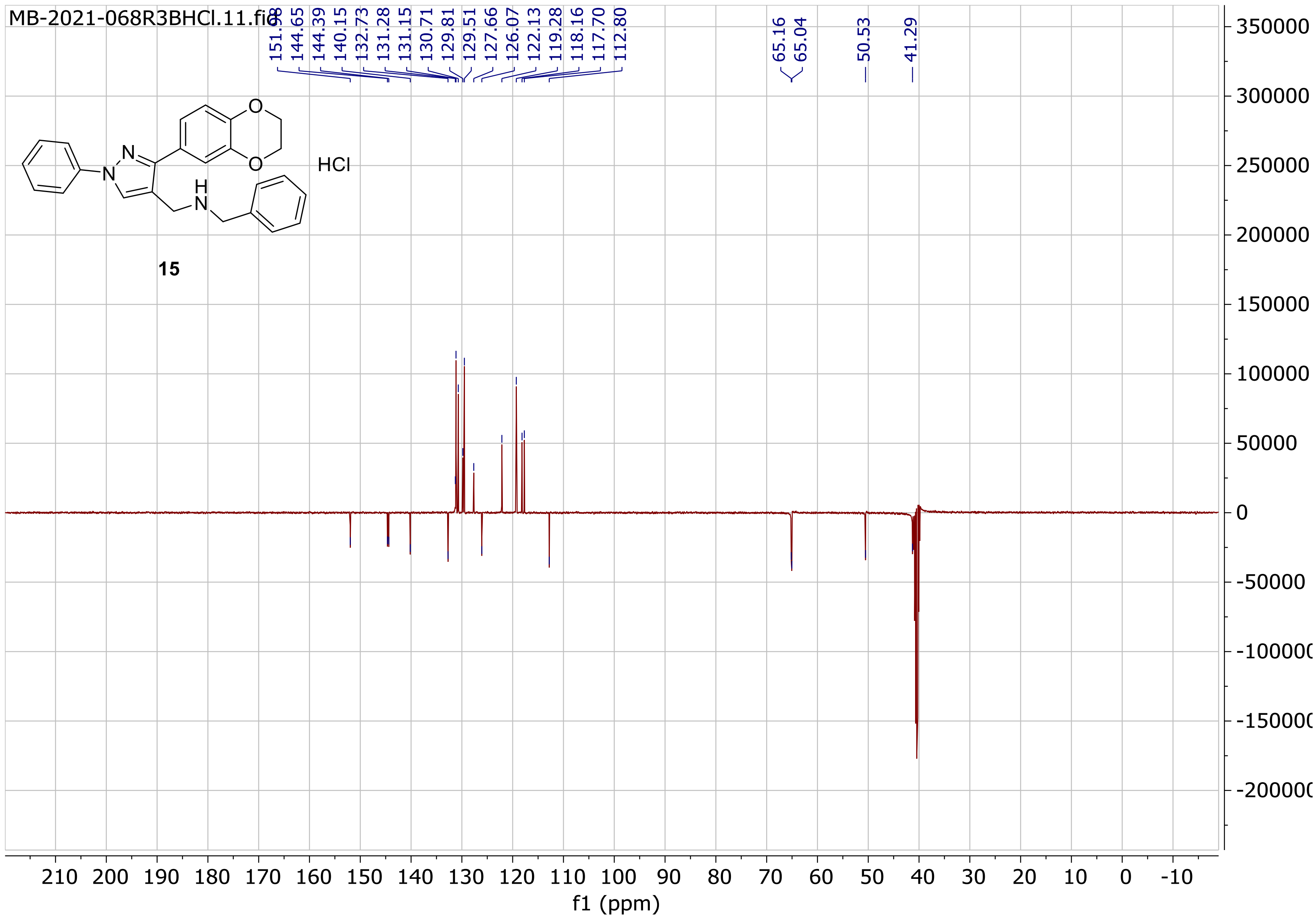

MB-2021-068R3BHCl.11.fid
151.98
144.65
144.39
140.15
132.73
131.28
131.15
130.71
129.81
129.51
127.66
126.07
122.13
119.28
118.16
117.70
112.80
65.16
65.04
50.53
41.29
N
N
O
O
HCl
H
N
15
350000
300000
250000
200000
150000
100000
50000
0
-50000
-100000
-150000
-200000
210 200 190 180 170 160 150 140 130 120 110 100 90 80 70 60 50 40 30 20 10 0 -10
f1 (ppm)

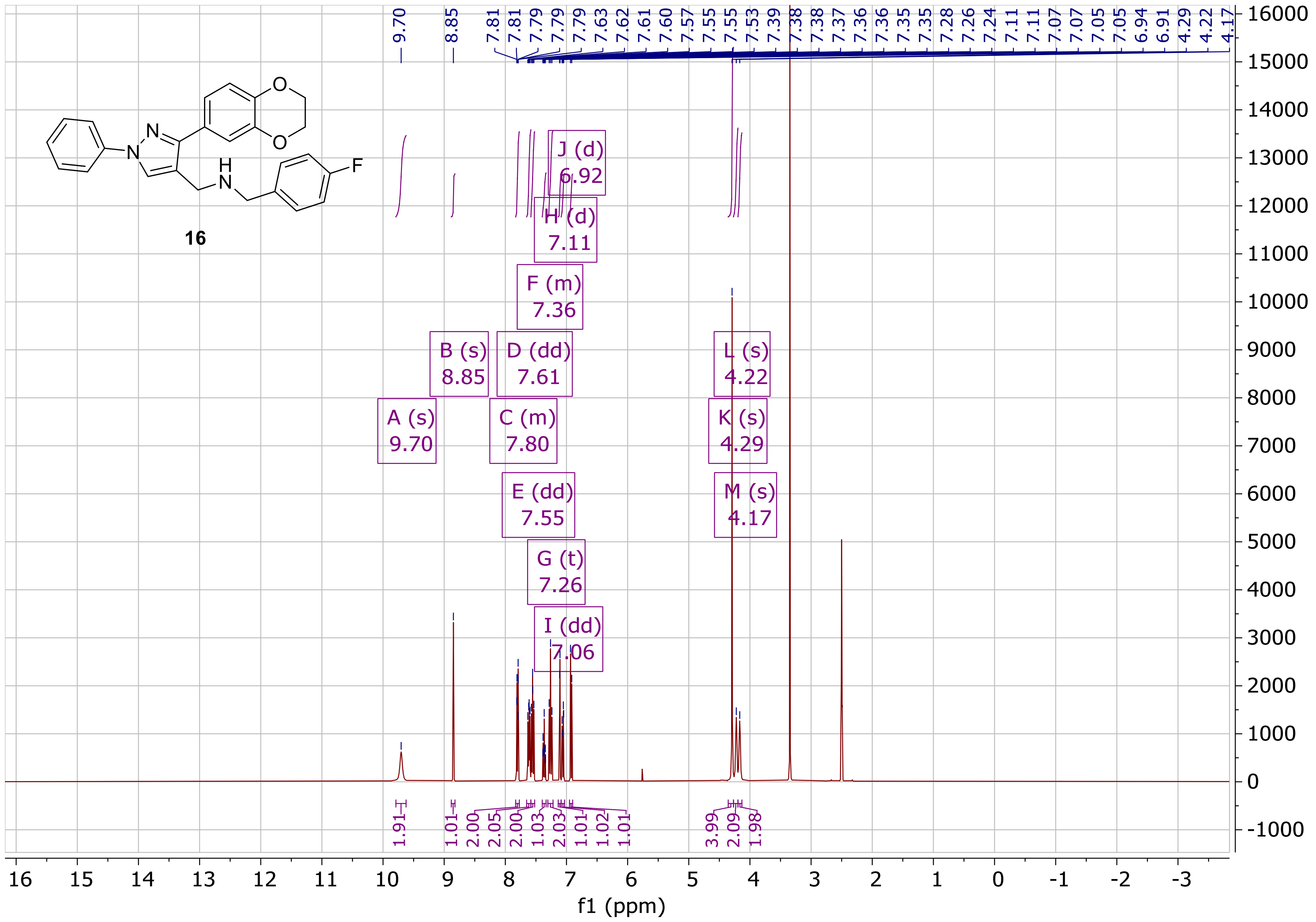

9.70
8.85
7.81 7.81 7.79 7.79 7.79 7.63 7.62 7.61 7.60 7.57 7.55 7.55 7.53 7.39 7.38 7.38 7.37 7.36 7.36 7.35 7.35 7.28 7.26 7.24 7.11 7.11 7.07 7.07 7.05 7.05 6.94 6.91 4.29 4.22 4.17
A (s) 9.70
B (s) 8.85
C (m) 7.80
D (dd) 7.61
E (dd) 7.55
F (m) 7.36
G (t) 7.26
H (d) 7.11
I (dd) 7.06
J (d) 6.92
K (s) 4.29
L (s) 4.22
M (s) 4.17
1.91
1.01
2.00
2.05
2.00
1.03
2.03
1.01
1.02
1.01
3.99
2.09
1.98
16
f1 (ppm)

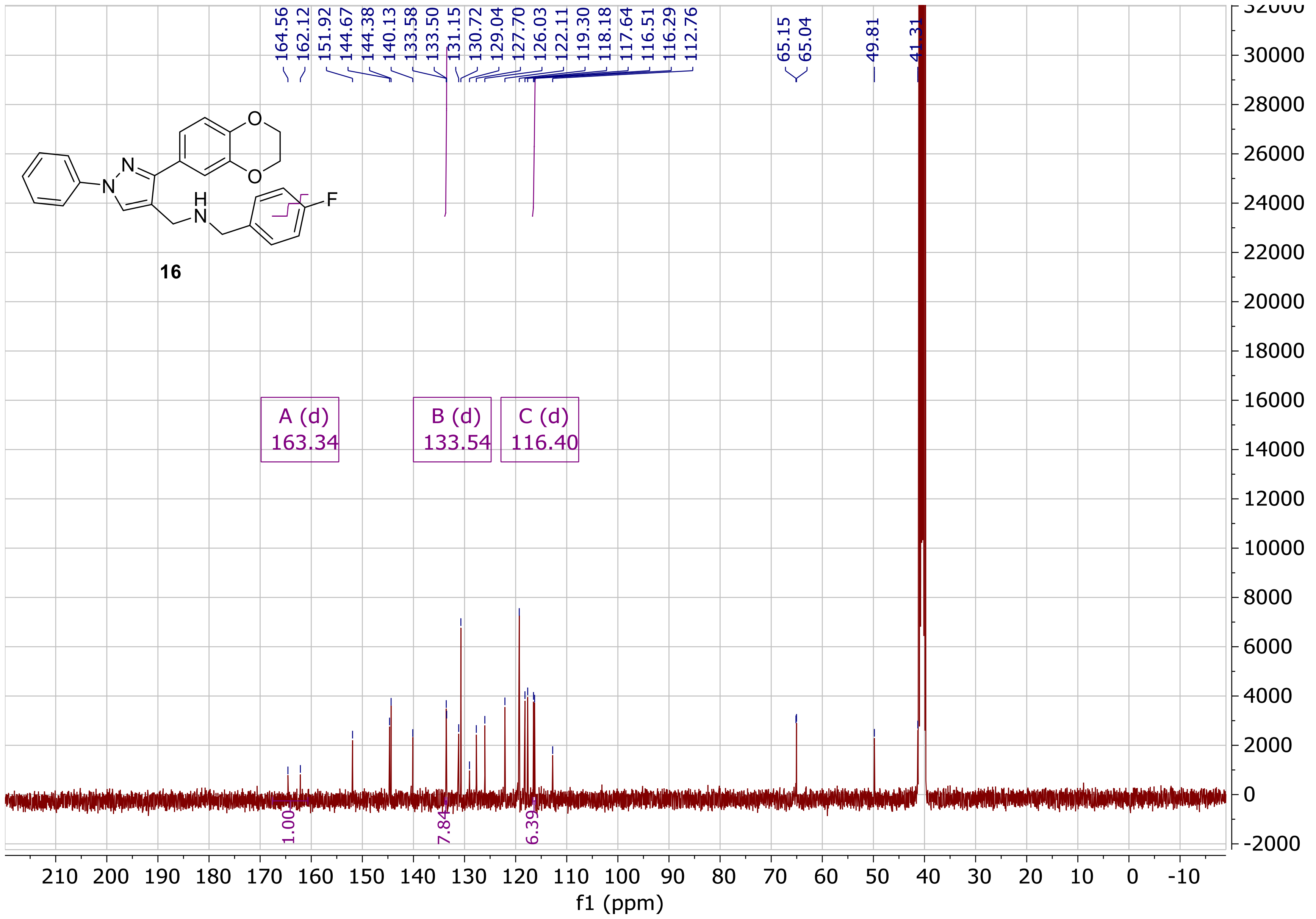

164.56
162.12
151.92
144.67
144.38
140.13
133.58
133.50
131.15
130.72
129.04
127.70
126.03
122.11
119.30
118.18
117.64
116.51
116.29
112.76
65.15
65.04
49.81
41.31
N
N
O
O
H
N
F
16
A (d)
163.34
B (d)
133.54
C (d)
116.40
1.00
7.84
6.39
32000
30000
28000
26000
24000
22000
20000
18000
16000
14000
12000
10000
8000
6000
4000
2000
0
-2000
210 200 190 180 170 160 150 140 130 120 110 100 90 80 70 60 50 40 30 20 10 0 -10
f1 (ppm)

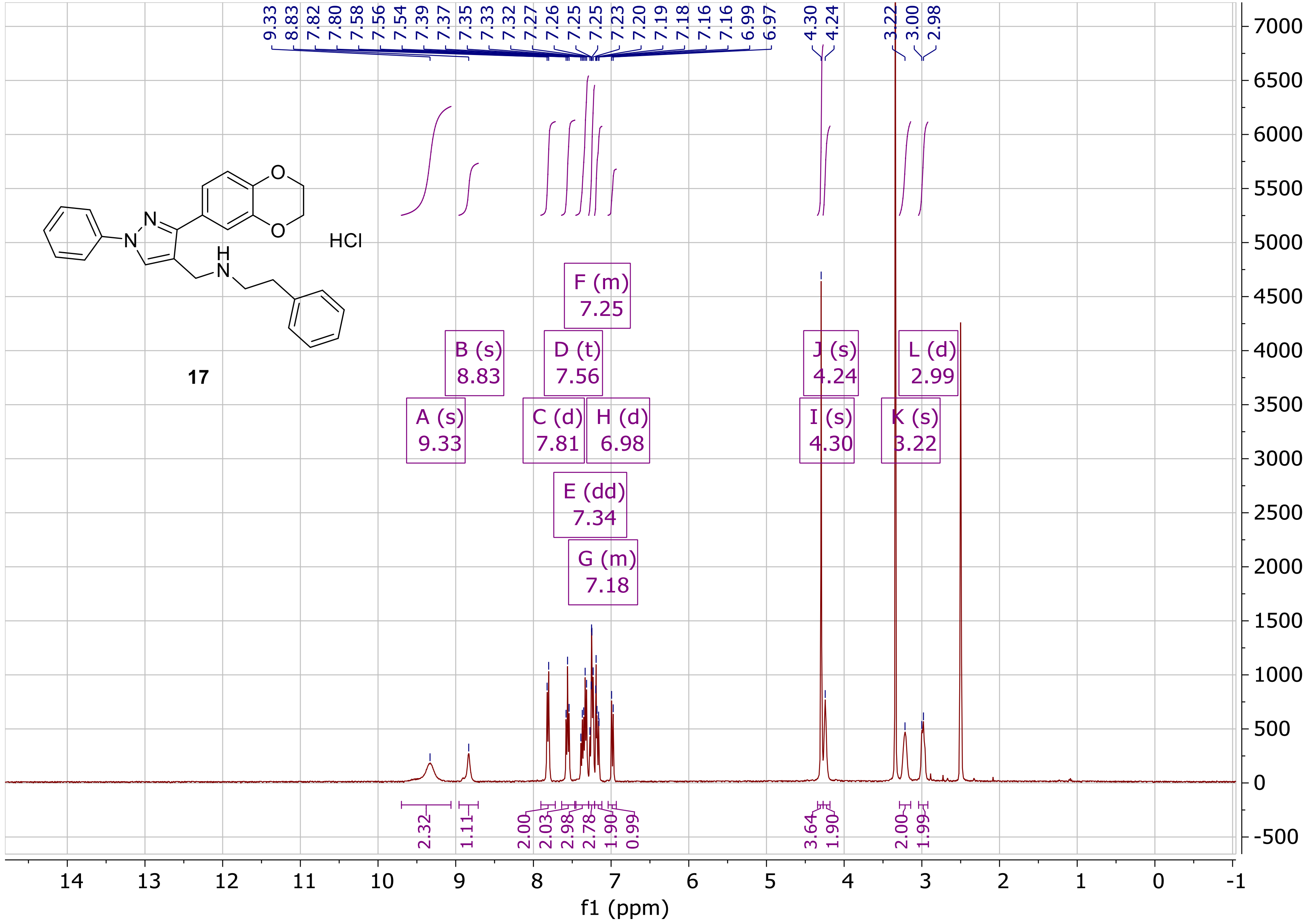

HCl
17
9.33 8.83 7.82 7.80 7.58 7.56 7.54 7.39 7.37 7.35 7.33 7.32 7.27 7.26 7.25 7.25 7.23 7.20 7.19 7.18 7.16 7.16 6.99 6.97 4.30 4.24 3.22 3.00 2.98
A (s) 9.33
B (s) 8.83
C (d) 7.81
D (t) 7.56
E (dd) 7.34
F (m) 7.25
G (m) 7.18
H (d) 6.98
I (s) 4.30
J (s) 4.24
K (s) 3.22
L (d) 2.99
2.32 1.11 2.00 2.03 2.98 2.78 1.90 0.99 3.64 1.90 2.00 1.99
f1 (ppm)

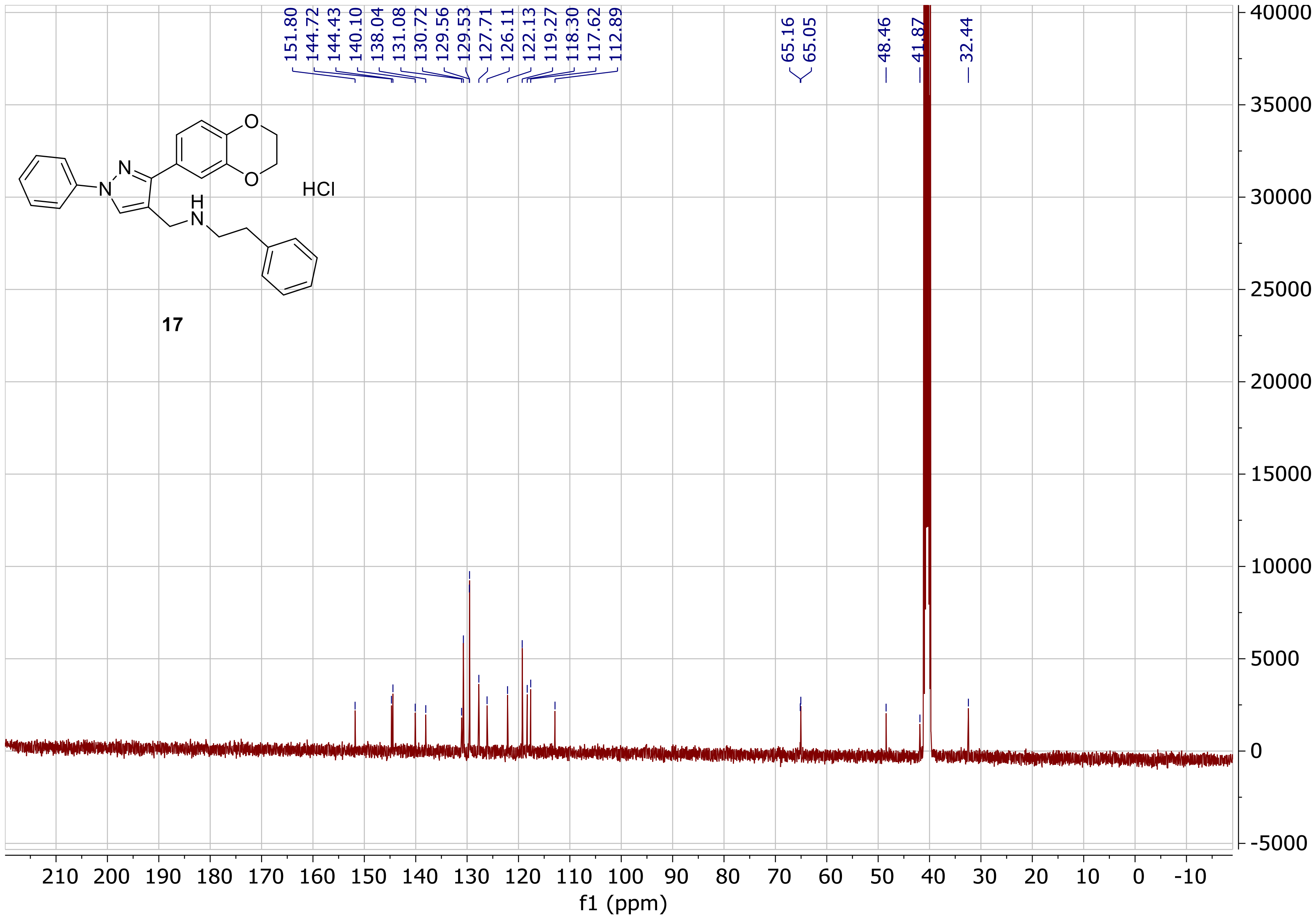

HCl
151.80
144.72
144.43
140.10
138.04
131.08
130.72
129.56
129.53
127.71
126.11
122.13
119.27
118.30
117.62
112.89
65.16
65.05
48.46
41.87
32.44
40000
35000
30000
25000
20000
15000
10000
5000
0
-5000
210 200 190 180 170 160 150 140 130 120 110 100 90 80 70 60 50 40 30 20 10 0 -10
f1 (ppm)

17

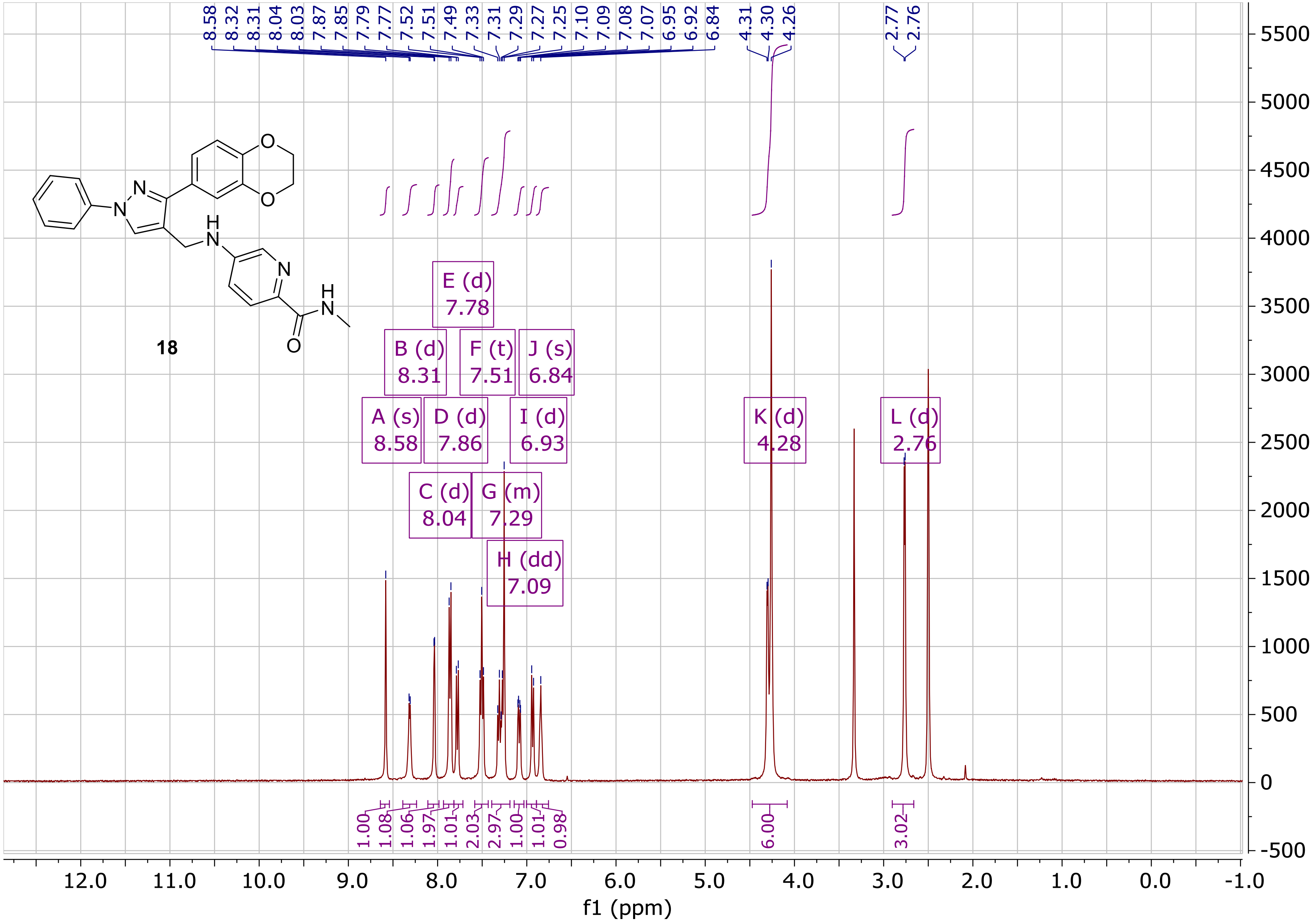
8.58
8.32
8.31
8.04
8.03
7.87
7.85
7.79
7.77
7.52
7.51
7.49
7.33
7.31
7.29
7.27
7.25
7.10
7.09
7.08
7.07
6.95
6.92
6.84
4.31
4.30
4.26
2.77
2.76
A (s) 8.58
B (d) 8.31
C (d) 8.04
D (d) 7.86
E (d) 7.78
F (t) 7.51
G (m) 7.29
H (dd) 7.09
I (d) 6.93
J (s) 6.84
K (d) 4.28
L (d) 2.76
1.00
1.08
1.06
1.97
1.01
2.03
2.97
1.00
1.01
0.98
6.00
3.02
f1 (ppm)

18

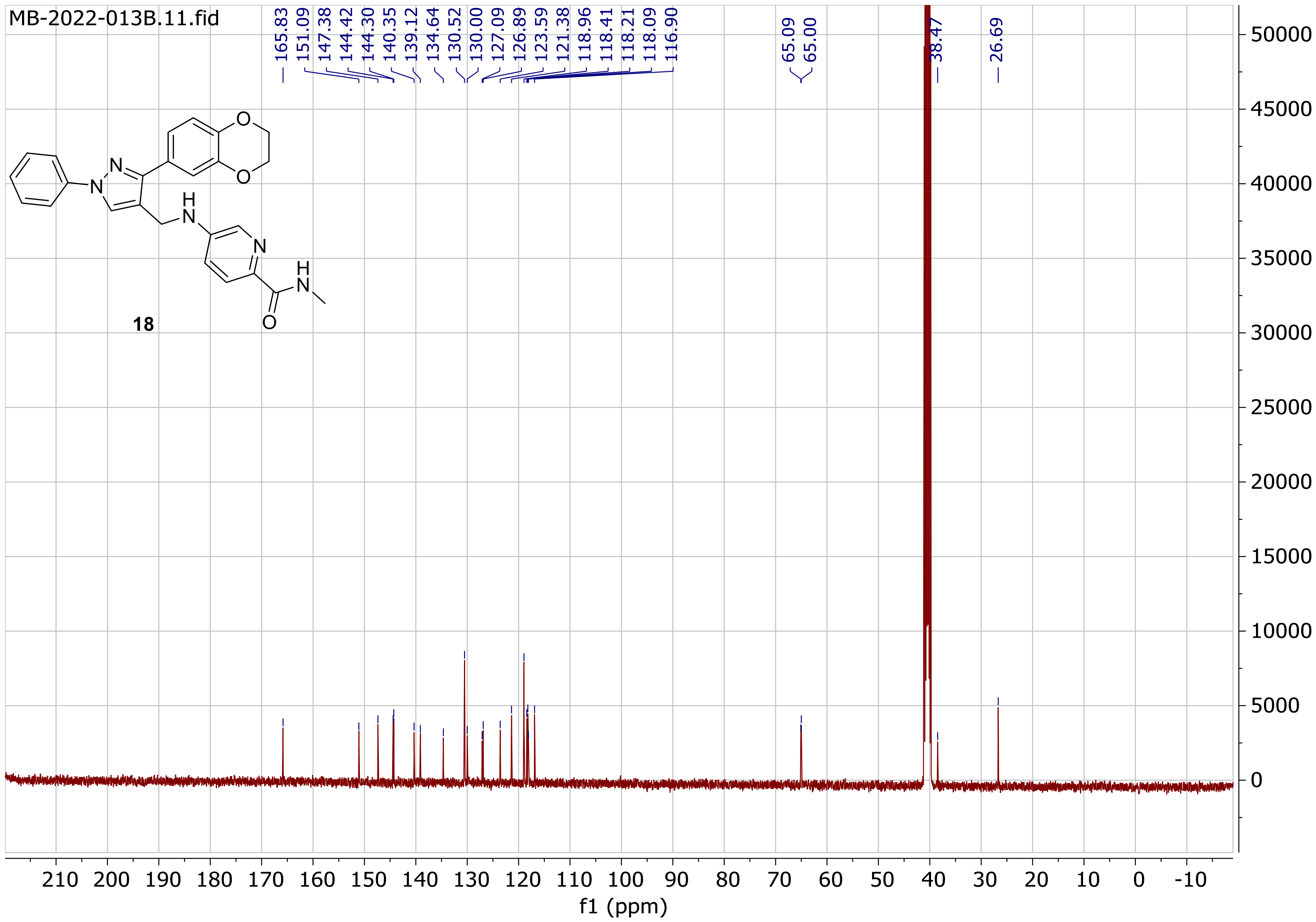
MB-2022-013B.11.fid
165.83
151.09
147.38
144.42
144.30
140.35
139.12
134.64
130.52
130.00
127.09
126.89
123.59
121.38
118.96
118.41
118.21
118.09
116.90
65.09
65.00
38.47
26.69
N
N
O
O
H
N
N
H
N
O
18
f1 (ppm)

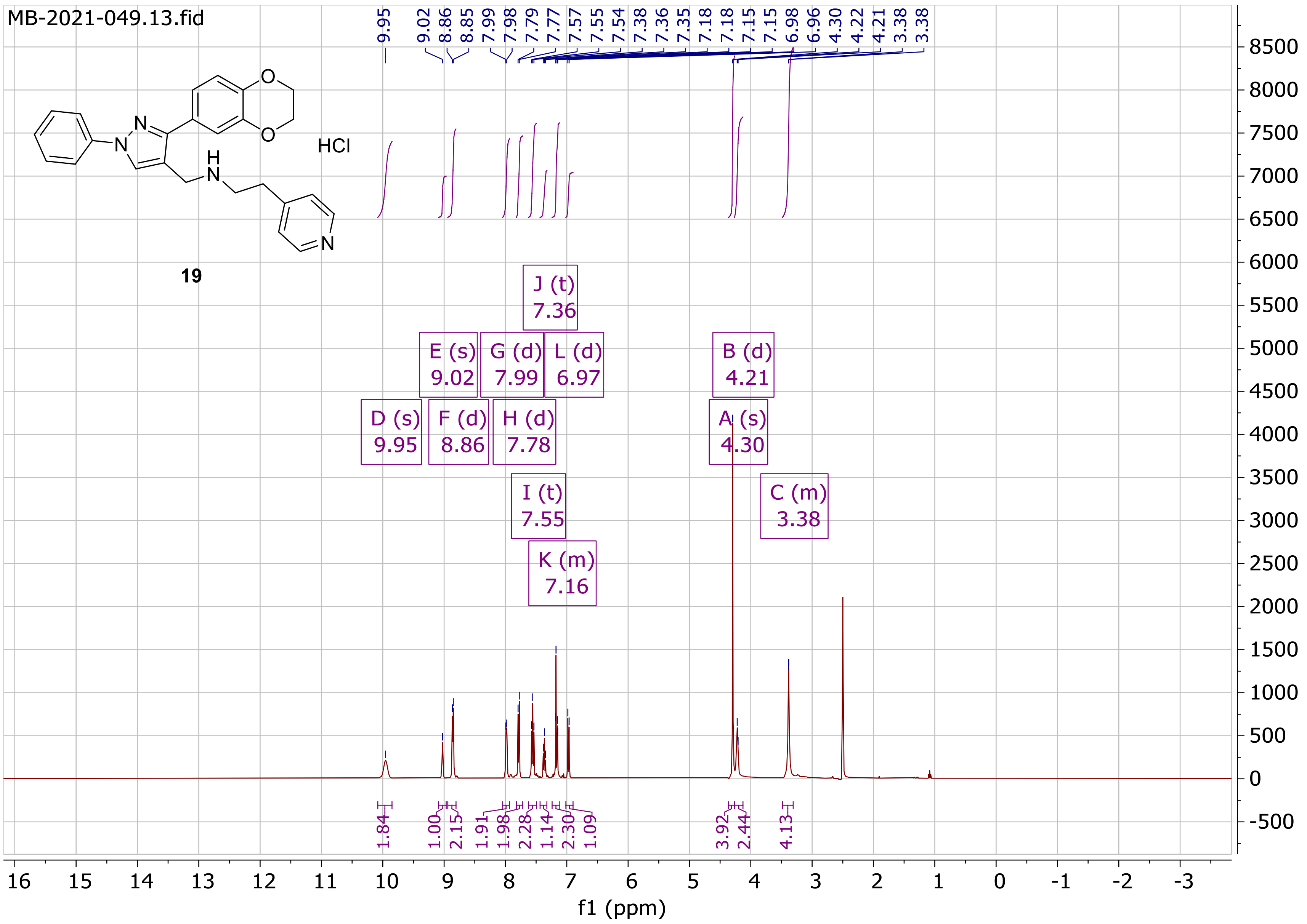

MB-2021-049.13.fid
HCl
19
9.95 9.02 8.86 8.85 7.99 7.98 7.79 7.77 7.57 7.55 7.54 7.38 7.36 7.35 7.18 7.18 7.15 7.15 6.98 6.96 4.30 4.22 4.21 3.38 3.38
D (s) 9.95
E (s) 9.02
F (d) 8.86
G (d) 7.99
H (d) 7.78
I (t) 7.55
J (t) 7.36
K (m) 7.16
L (d) 6.97
A (s) 4.30
B (d) 4.21
C (m) 3.38
1.84 1.00 2.15 1.91 1.98 2.28 1.14 2.30 1.09 3.92 2.44 4.13
f1 (ppm)

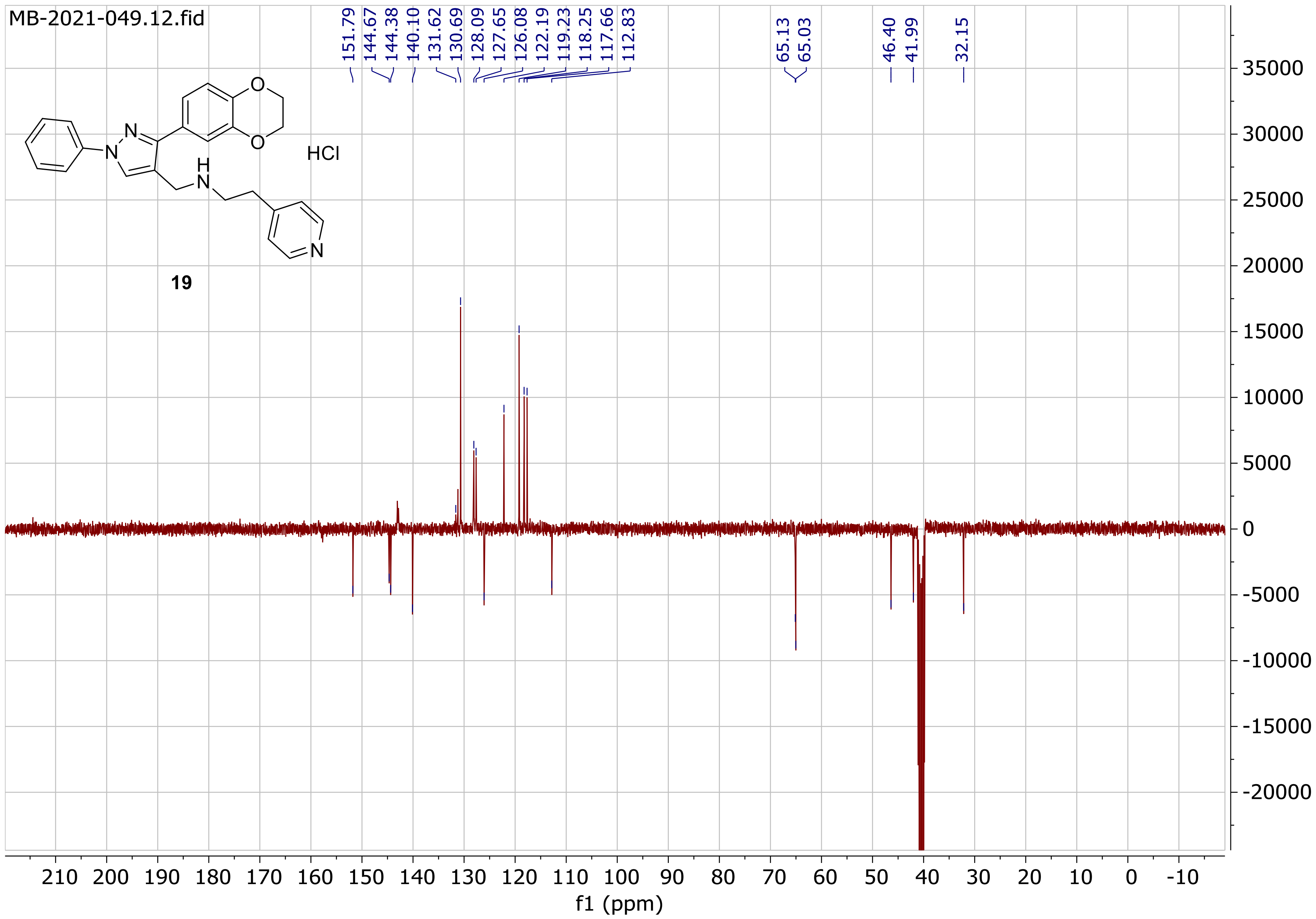

MB-2021-049.12.fid
151.79
144.67
144.38
140.10
131.62
130.69
128.09
127.65
126.08
122.19
119.23
118.25
117.66
112.83
65.13
65.03
46.40
41.99
32.15
HCl
19
f1 (ppm)

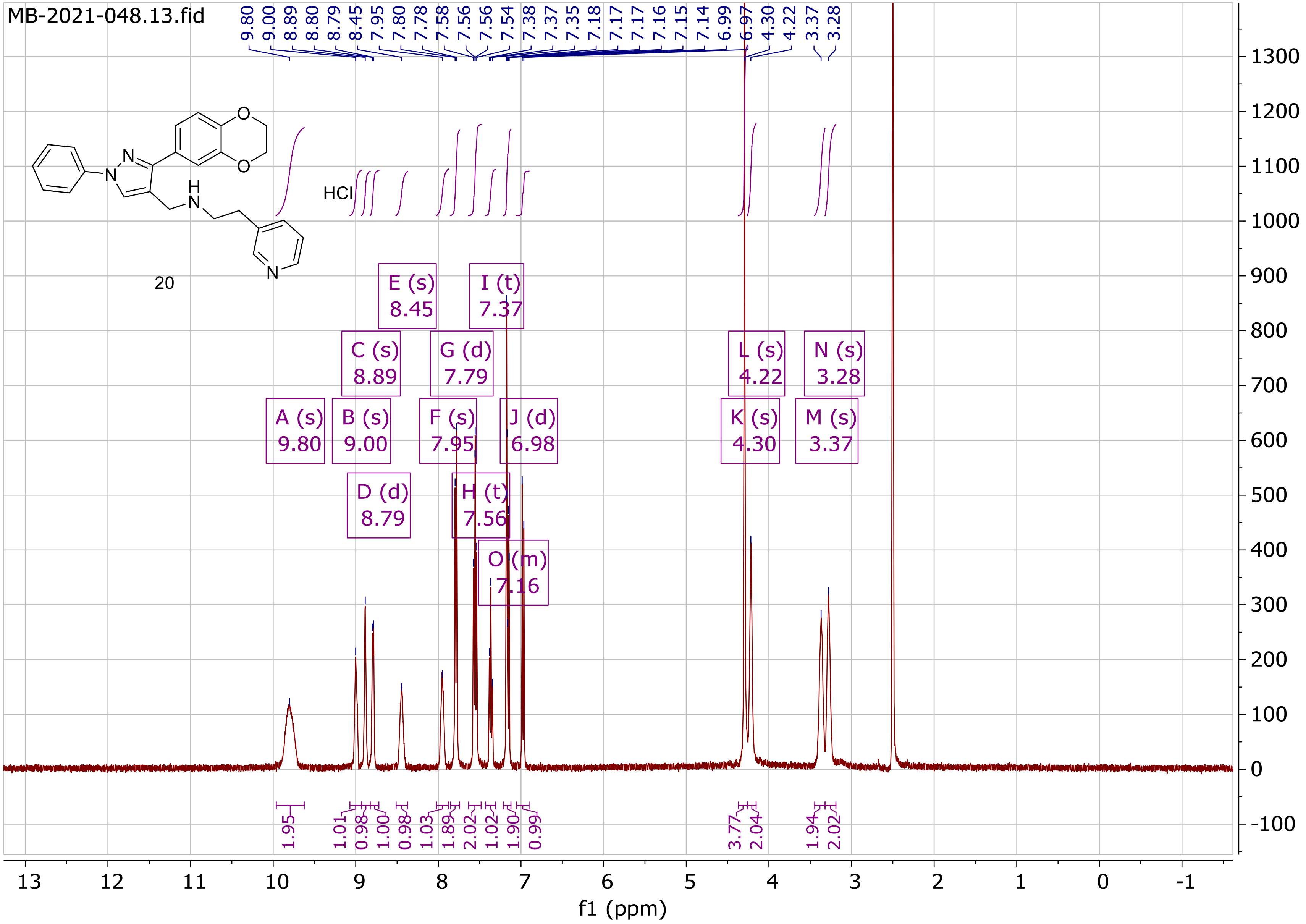

MB-2021-048.13.fid
9.80 9.00 8.89 8.80 8.79 8.45 7.95 7.80 7.78 7.58 7.56 7.56 7.54 7.38 7.37 7.35 7.18 7.17 7.17 7.16 7.15 7.14 6.99 6.97 4.30 4.22 3.37 3.28
HCl
20
A (s) 9.80
B (s) 9.00
C (s) 8.89
D (d) 8.79
E (s) 8.45
F (s) 7.95
G (d) 7.79
H (t) 7.56
I (t) 7.37
J (d) 6.98
K (s) 4.30
L (s) 4.22
M (s) 3.37
N (s) 3.28
O (m) 7.16
1.95 1.01 0.98 1.00 0.98 1.03 1.89 2.02 1.02 1.90 0.99 3.77 2.04 1.94 2.02
f1 (ppm)

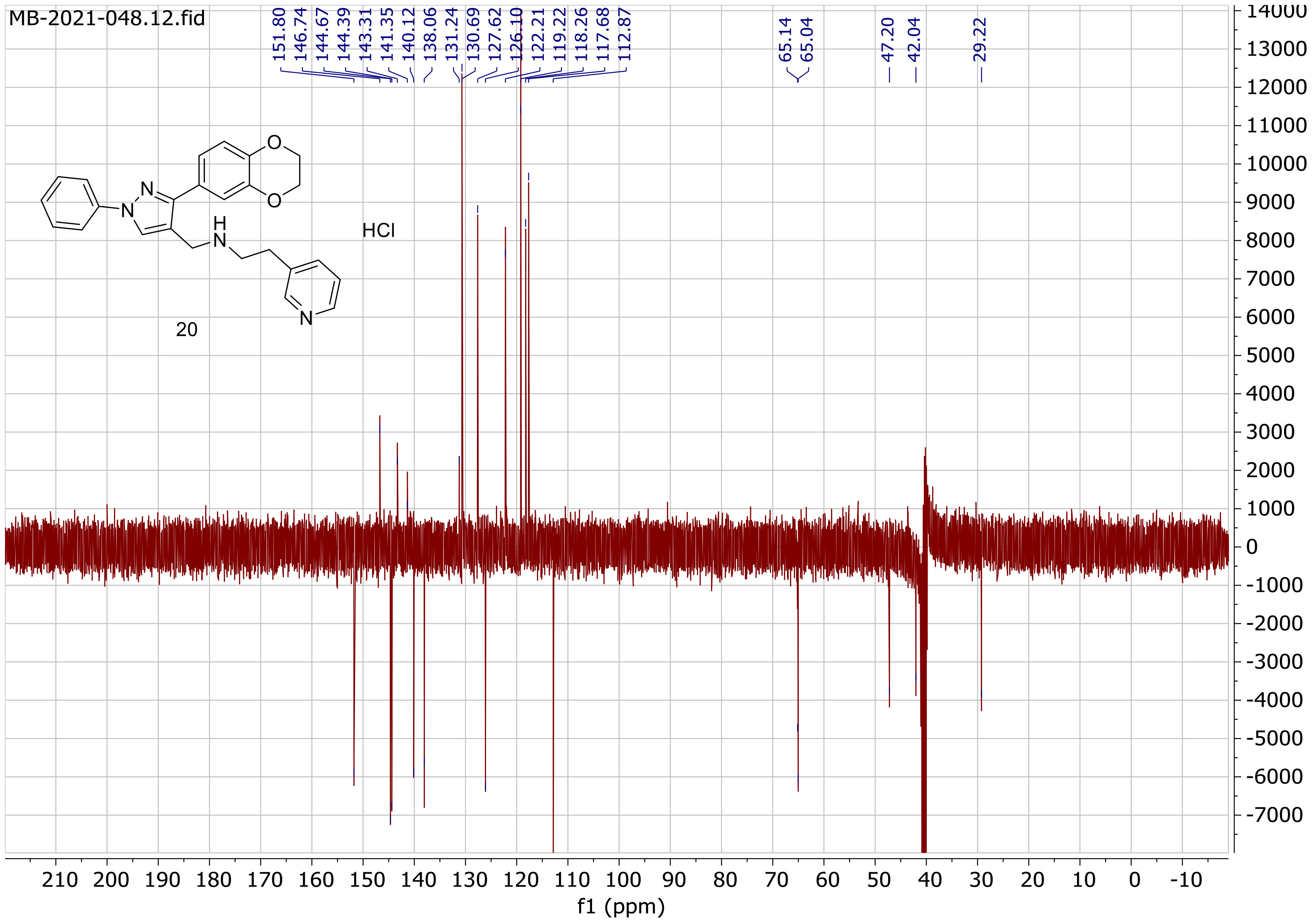
MB-2021-048.12.fid
151.80
146.74
144.67
144.39
143.31
141.35
140.12
138.06
131.24
130.69
127.62
126.10
122.21
119.22
118.26
117.68
112.87
65.14
65.04
47.20
42.04
29.22
HCl
20
f1 (ppm)

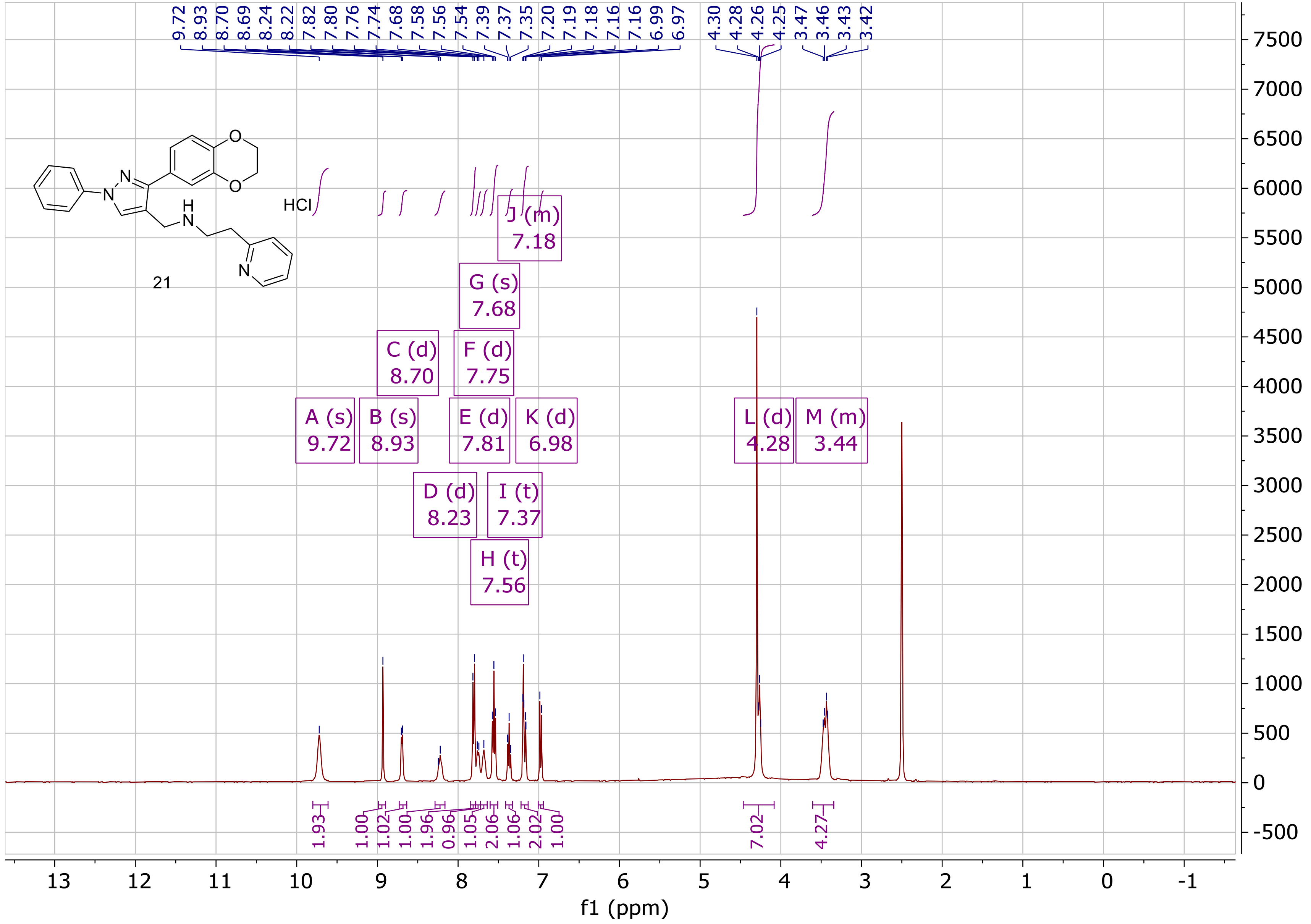
9.72 8.93 8.70 8.69 8.24 8.22 7.82 7.80 7.76 7.74 7.68 7.58 7.56 7.54 7.39 7.37 7.35 7.20 7.19 7.18 7.16 7.16 6.99 6.97
4.30 4.28 4.26 4.25 3.47 3.46 3.43 3.42
HCl
21
A (s) 9.72
B (s) 8.93
C (d) 8.70
D (d) 8.23
E (d) 7.81
F (d) 7.75
G (s) 7.68
H (t) 7.56
I (t) 7.37
J (m) 7.18
K (d) 6.98
L (d) 4.28
M (m) 3.44
1.93 1.00 1.02 1.00 1.96 0.96 1.05 2.06 1.06 2.02 1.00 7.02 4.27
f1 (ppm)

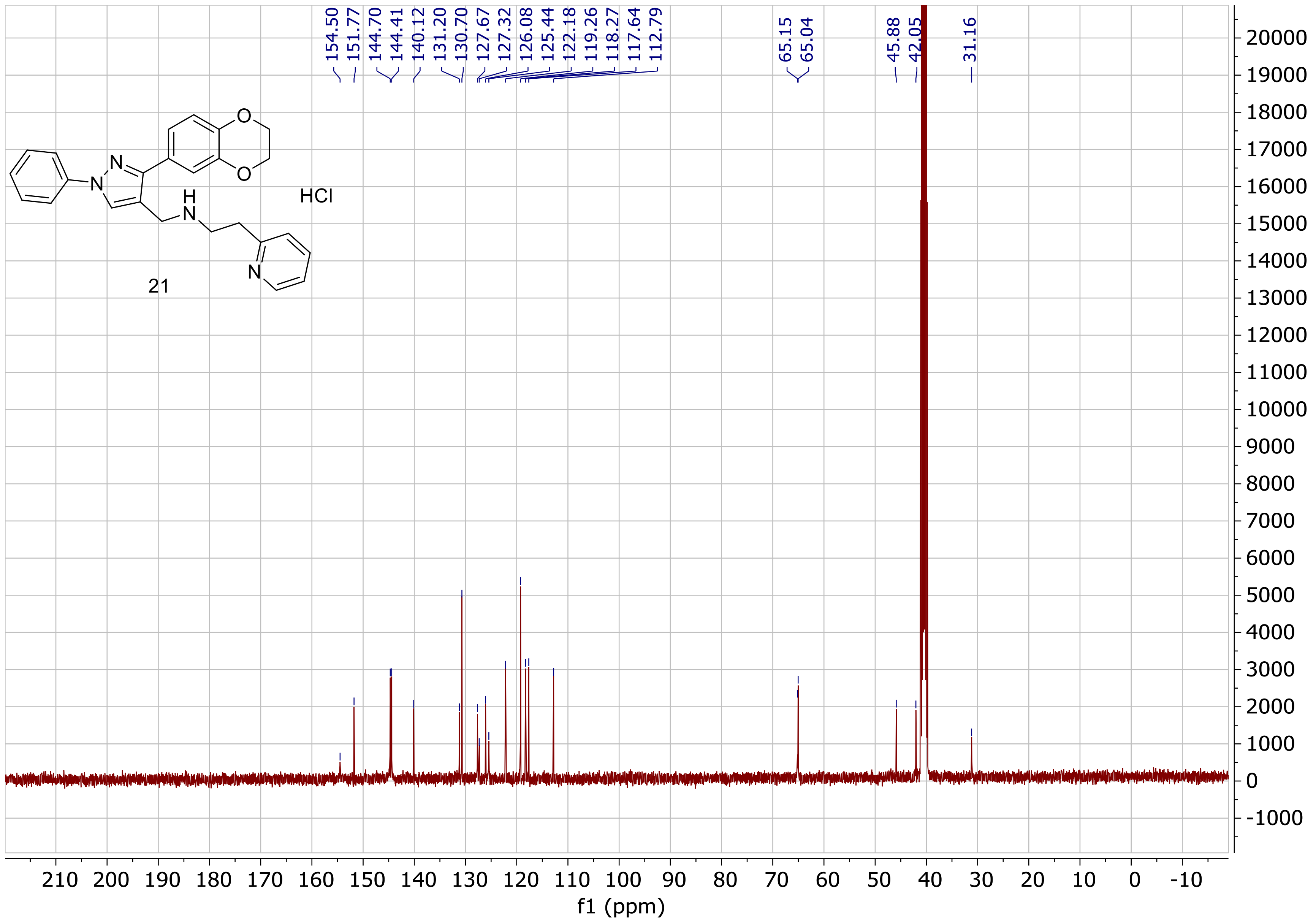
154.50
151.77
144.70
144.41
140.12
131.20
130.70
127.67
127.32
126.08
125.44
122.18
119.26
118.27
117.64
112.79
65.15
65.04
45.88
42.05
31.16
HCl
21
f1 (ppm)

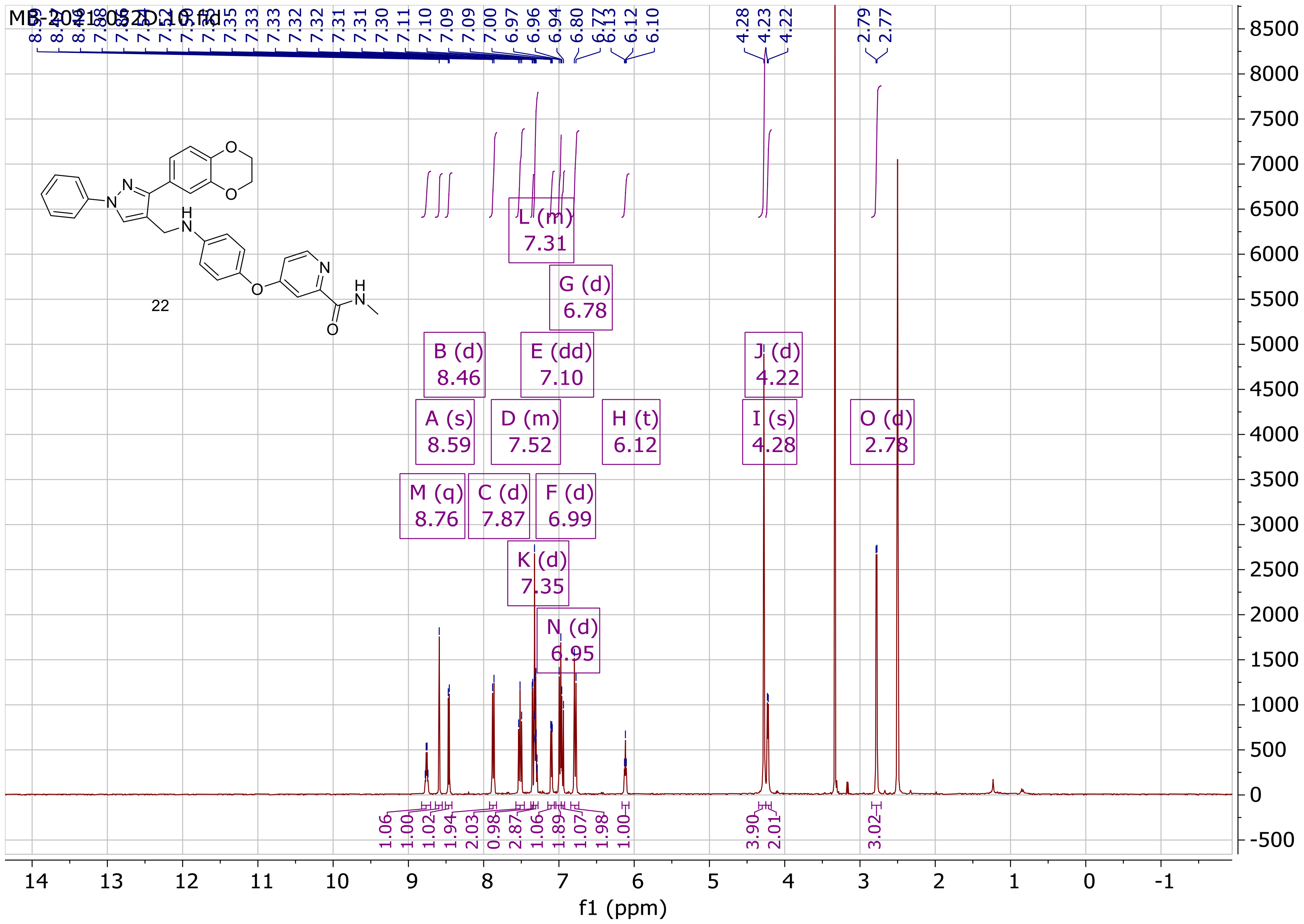
MB-20210521D.10.fid
B (d) 8.46
A (s) 8.59
M (q) 8.76
C (d) 7.87
D (m) 7.52
L (m) 7.31
K (d) 7.35
E (dd) 7.10
F (d) 6.99
N (d) 6.95
G (d) 6.78
H (t) 6.12
J (d) 4.22
I (s) 4.28
O (d) 2.78
f1 (ppm)

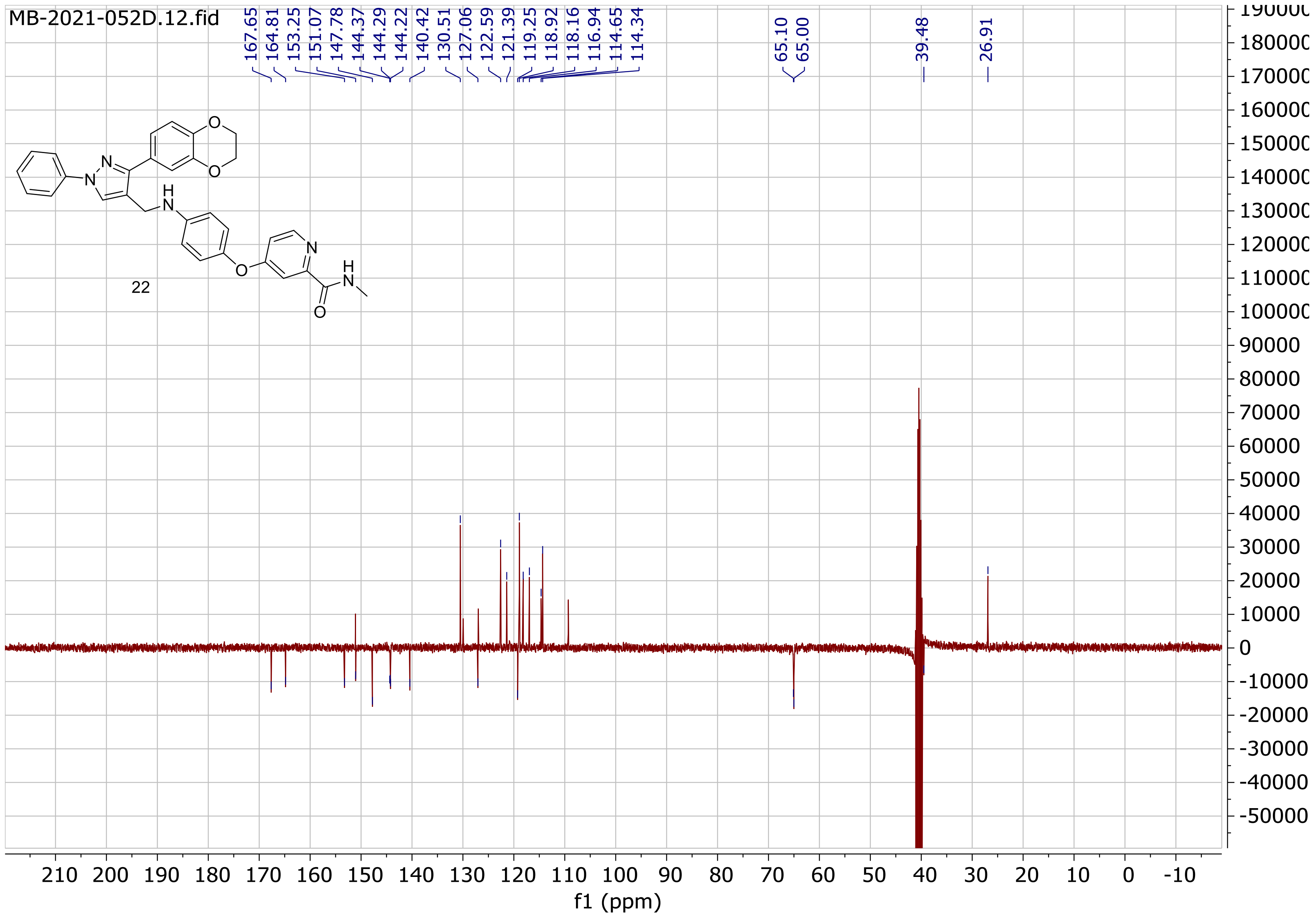
MB-2021-052D.12.fid
167.65
164.81
153.25
151.07
147.78
144.37
144.29
144.22
140.42
130.51
127.06
122.59
121.39
119.25
118.92
118.16
116.94
114.65
114.34
65.10
65.00
39.48
26.91
22
f1 (ppm)

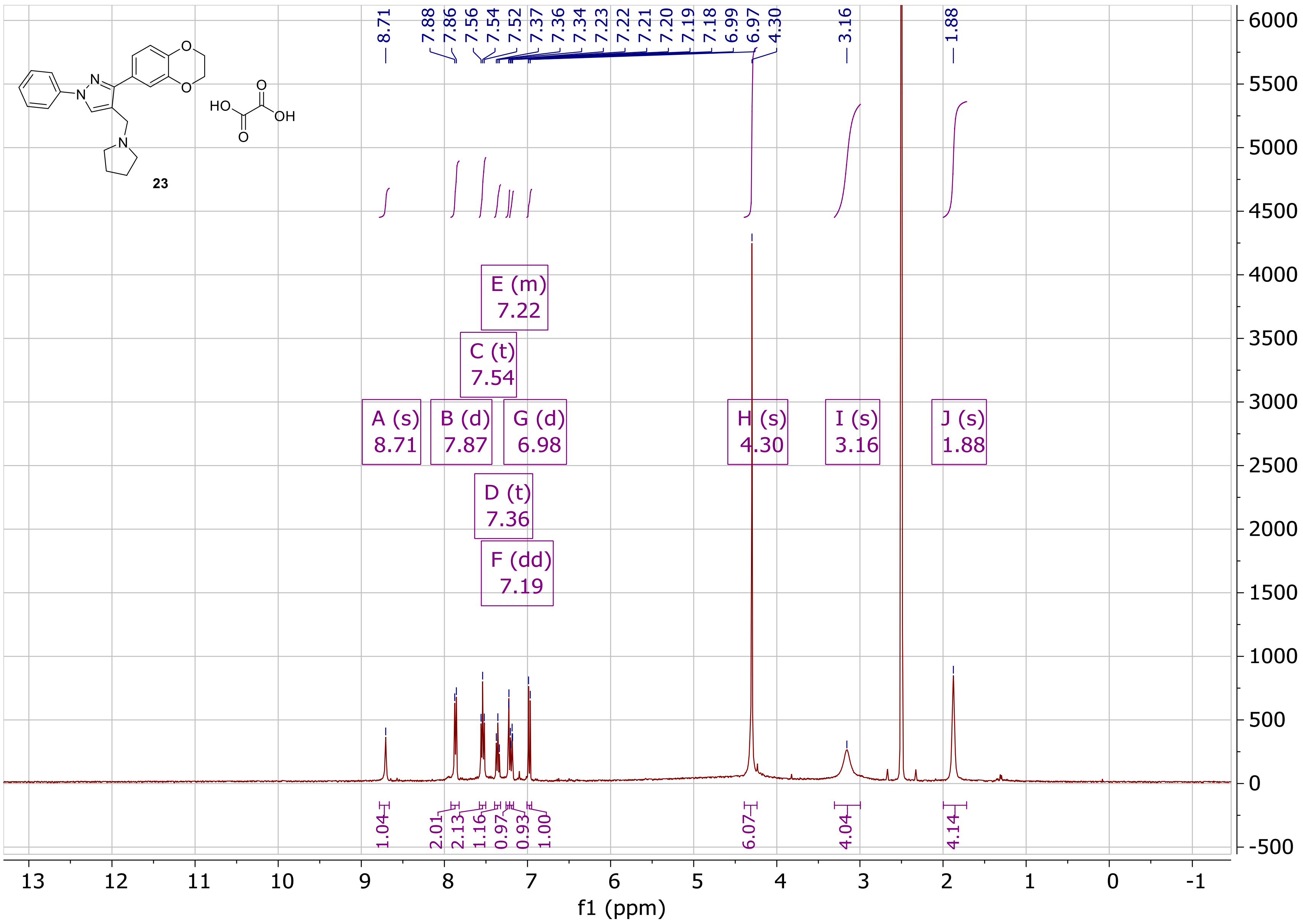

23
8.71
7.88
7.86
7.56
7.54
7.52
7.37
7.36
7.34
7.23
7.22
7.21
7.20
7.19
7.18
6.99
6.97
4.30
3.16
1.88
E (m) 7.22
C (t) 7.54
A (s) 8.71
B (d) 7.87
G (d) 6.98
D (t) 7.36
F (dd) 7.19
H (s) 4.30
I (s) 3.16
J (s) 1.88
1.04
2.01
2.13
1.16
0.97
0.93
1.00
6.07
4.04
4.14
f1 (ppm)

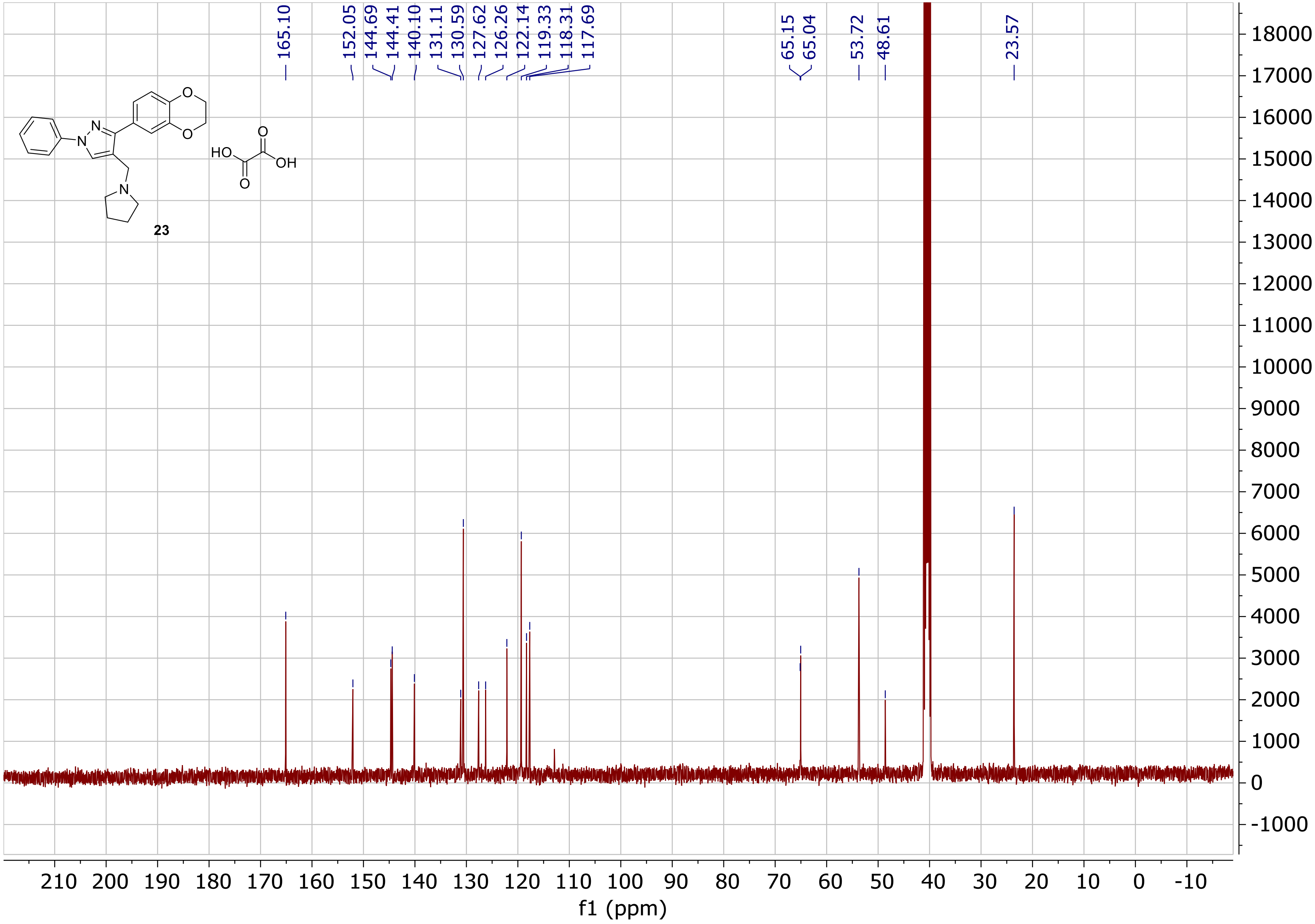
165.10
152.05
144.69
144.41
140.10
131.11
130.59
127.62
126.26
122.14
119.33
118.31
117.69
65.15
65.04
53.72
48.61
23.57
HO
O
O
OH
N
N
O
O
N
23
18000
17000
16000
15000
14000
13000
12000
11000
10000
9000
8000
7000
6000
5000
4000
3000
2000
1000
0
-1000
210 200 190 180 170 160 150 140 130 120 110 100 90 80 70 60 50 40 30 20 10 0 -10
f1 (ppm)

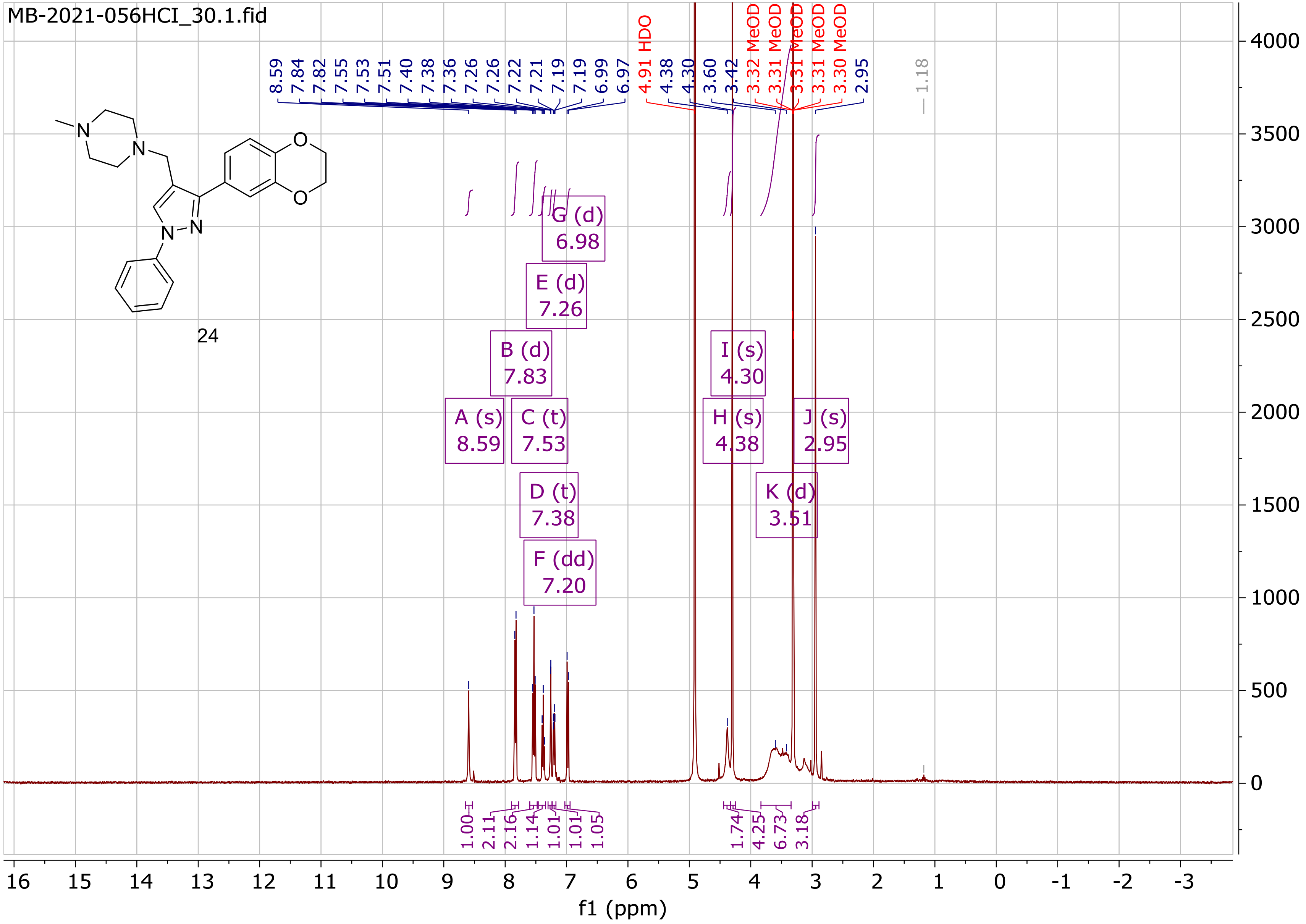
MB-2021-056HCl_30.1.fid
8.59
7.84
7.82
7.55
7.53
7.51
7.40
7.38
7.36
7.26
7.26
7.22
7.21
7.19
7.19
6.99
6.97
4.91 HDO
4.38
4.30
3.60
3.42
3.32 MeOD
3.31 MeOD
3.31 MeOD
3.31 MeOD
3.30 MeOD
2.95
1.18
24
G (d) 6.98
E (d) 7.26
B (d) 7.83
I (s) 4.30
A (s) 8.59
C (t) 7.53
H (s) 4.38
J (s) 2.95
D (t) 7.38
K (d) 3.51
F (dd) 7.20
1.00
2.11
2.16
1.14
1.01
1.01
1.05
1.74
4.25
6.73
3.18
f1 (ppm)

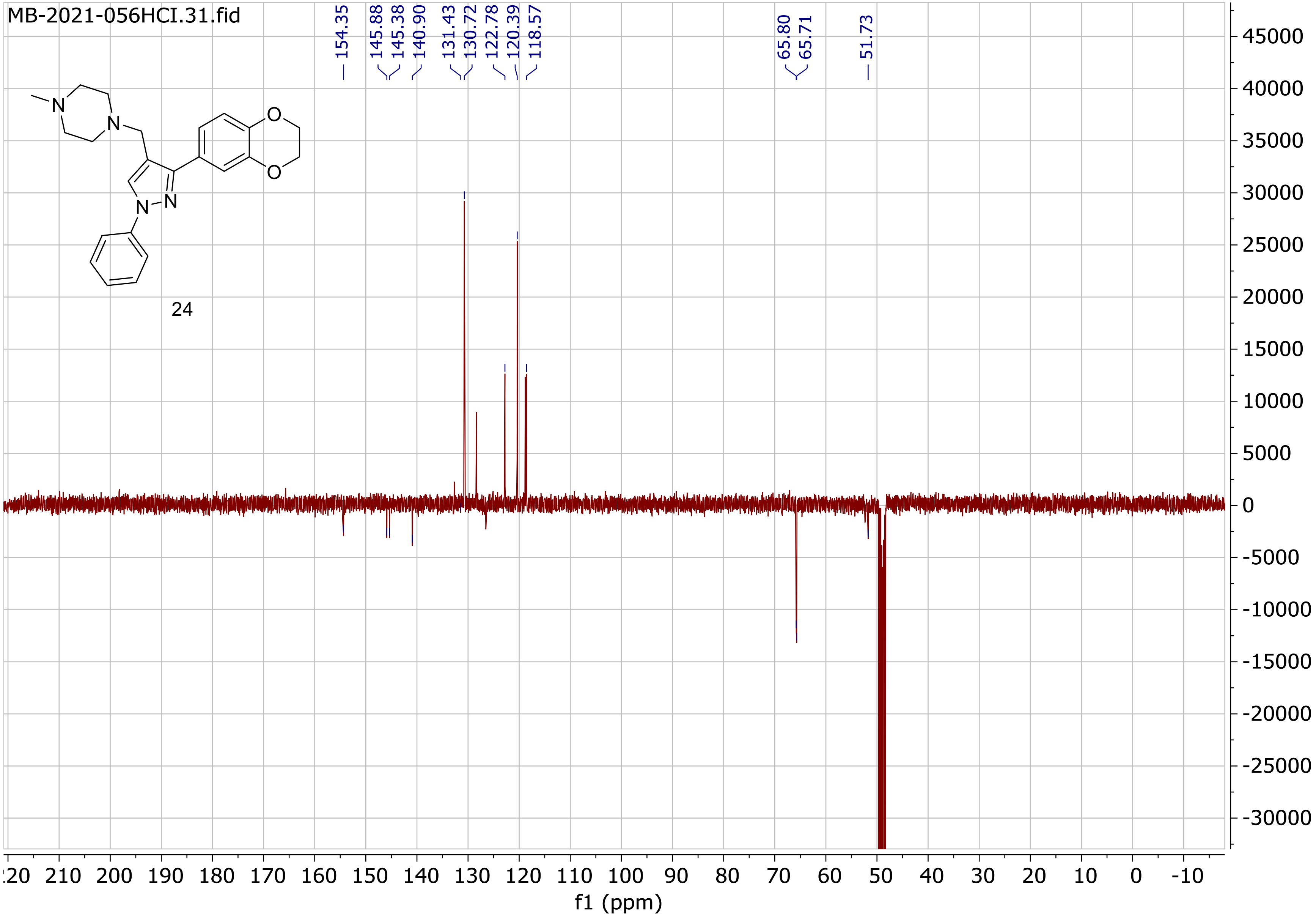
MB-2021-056HCl.31.fid
154.35
145.88
145.38
140.90
131.43
130.72
122.78
120.39
118.57
65.80
65.71
51.73
N
N
N
N
O
O
24
45000
40000
35000
30000
25000
20000
15000
10000
5000
0
-5000
-10000
-15000
-20000
-25000
-30000
210 200 190 180 170 160 150 140 130 120 110 100 90 80 70 60 50 40 30 20 10 0 -10
f1 (ppm)

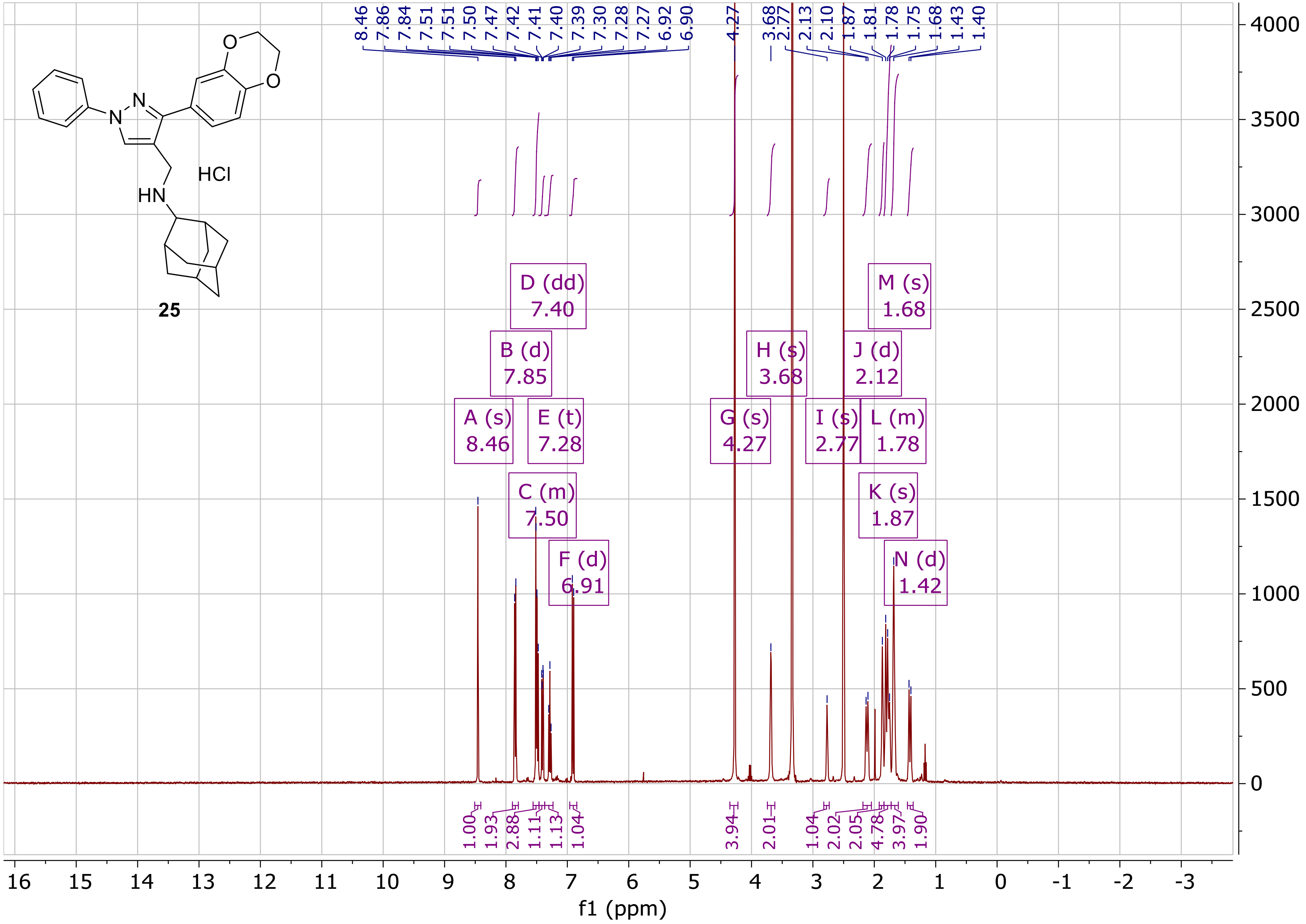
8.46 7.86 7.84 7.51 7.51 7.50 7.47 7.42 7.41 7.40 7.39 7.30 7.28 7.27 6.92 6.90
4.27 3.68 2.77 2.13 2.10 1.87 1.81 1.78 1.75 1.68 1.43 1.40
HCl
HN
N
O
25
A (s) 8.46
B (d) 7.85
C (m) 7.50
D (dd) 7.40
E (t) 7.28
F (d) 6.91
G (s) 4.27
H (s) 3.68
I (s) 2.77
J (d) 2.12
K (s) 1.87
L (m) 1.78
M (s) 1.68
N (d) 1.42
1.00 1.93 2.88 1.11 1.13 1.04 3.94 2.01 1.04 2.02 2.05 4.78 3.97 1.90
4000 3500 3000 2500 2000 1500 1000 500 0
16 15 14 13 12 11 10 9 8 7 6 5 4 3 2 1 0 -1 -2 -3
f1 (ppm)

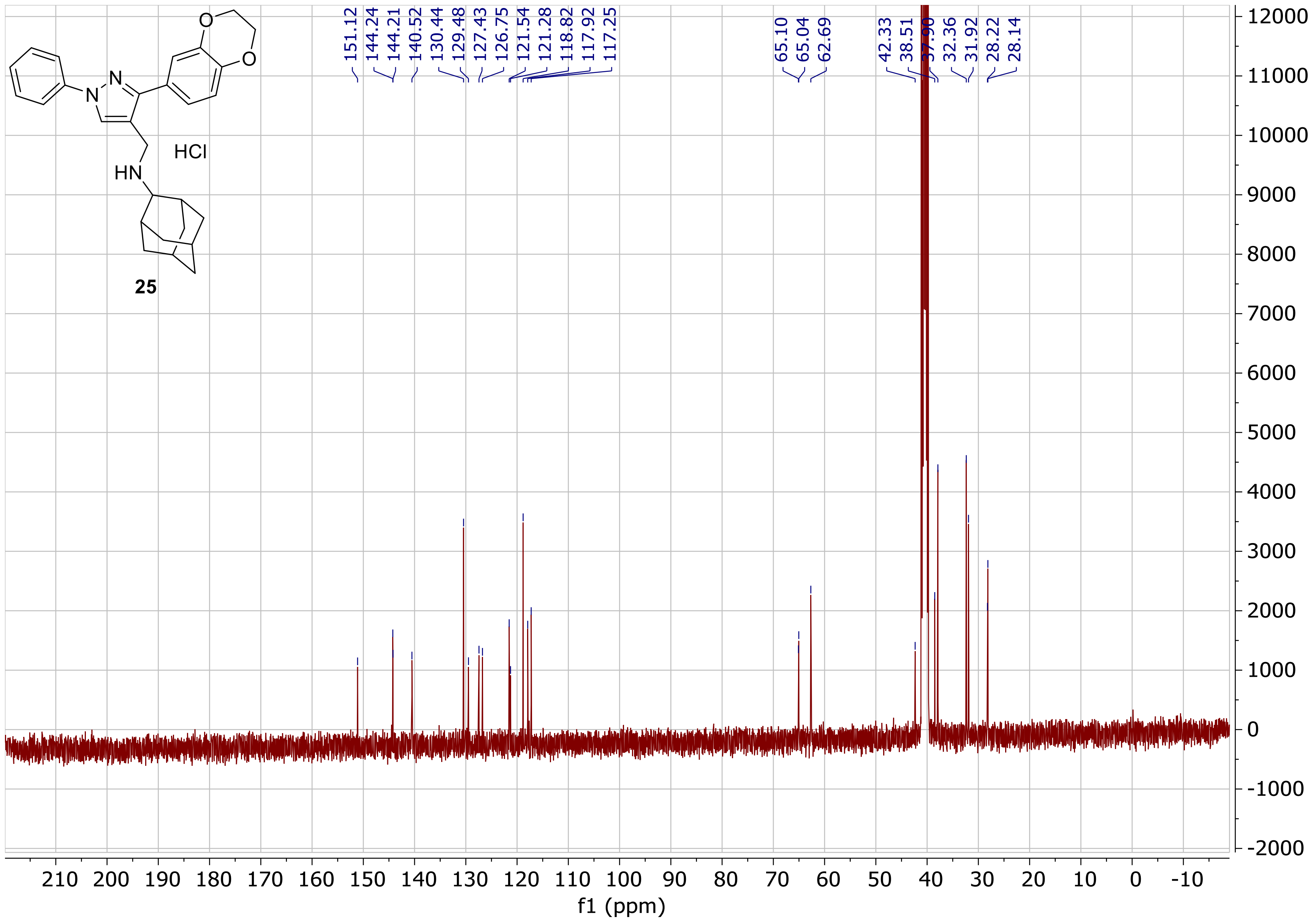

HN
HCl
25
151.12
144.24
144.21
140.52
130.44
129.48
127.43
126.75
121.54
121.28
118.82
117.92
117.25
65.10
65.04
62.69
42.33
38.51
37.90
32.36
31.92
28.22
28.14
f1 (ppm)

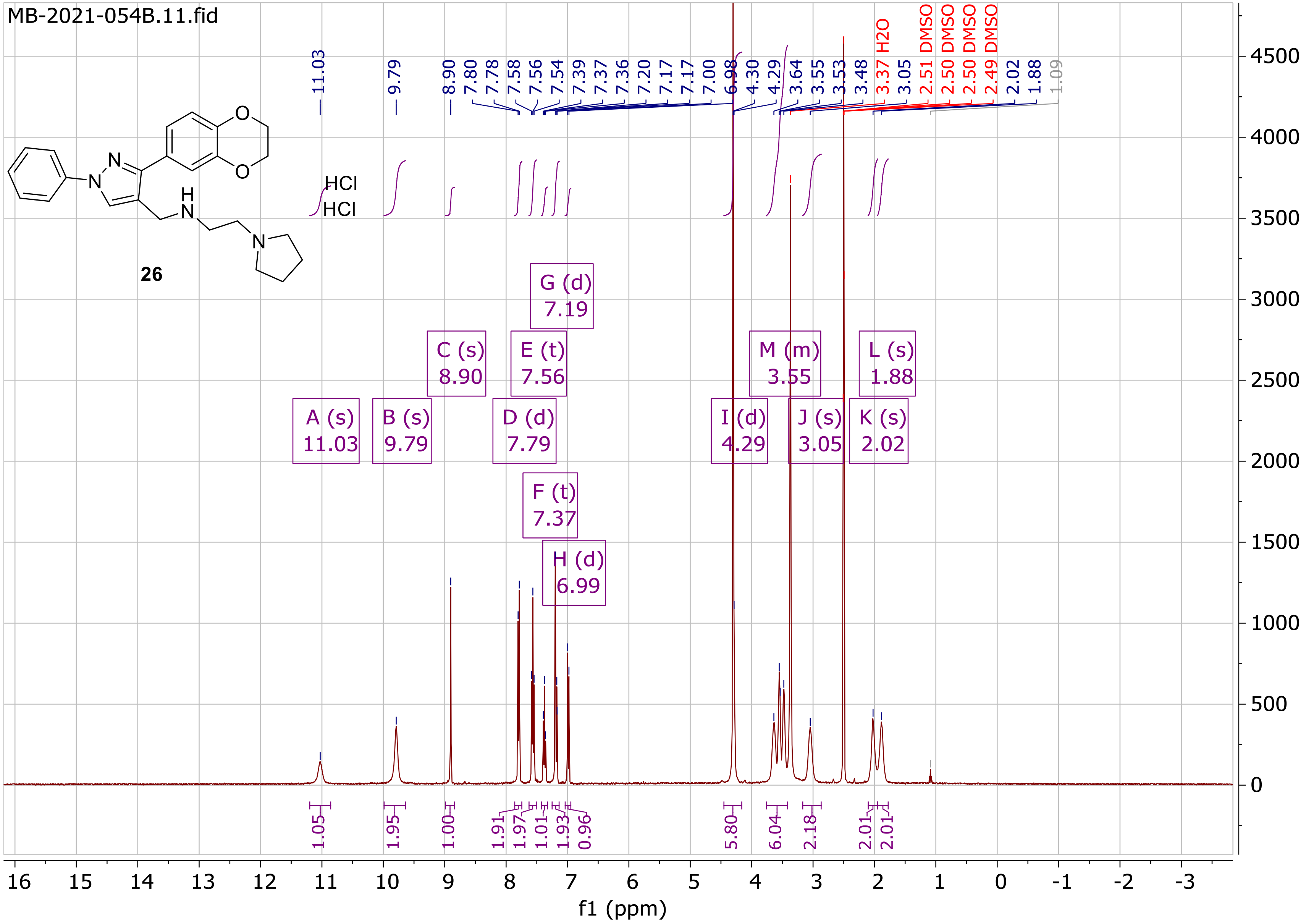
MB-2021-054B.11.fid
HCl
HCl
26
11.03
9.79
8.90
7.80
7.78
7.58
7.56
7.54
7.39
7.37
7.36
7.20
7.17
7.17
7.00
6.98
4.30
4.29
3.64
3.55
3.53
3.48
3.37 H2O
3.05
2.51 DMSO
2.50 DMSO
2.50 DMSO
2.49 DMSO
2.02
1.88
1.09
A (s) 11.03
B (s) 9.79
C (s) 8.90
D (d) 7.79
E (t) 7.56
F (t) 7.37
G (d) 7.19
H (d) 6.99
I (d) 4.29
J (s) 3.05
K (s) 2.02
L (s) 1.88
M (m) 3.55
1.05
1.95
1.00
1.91
1.97
1.01
1.93
0.96
5.80
6.04
2.18
2.01
2.01
f1 (ppm)

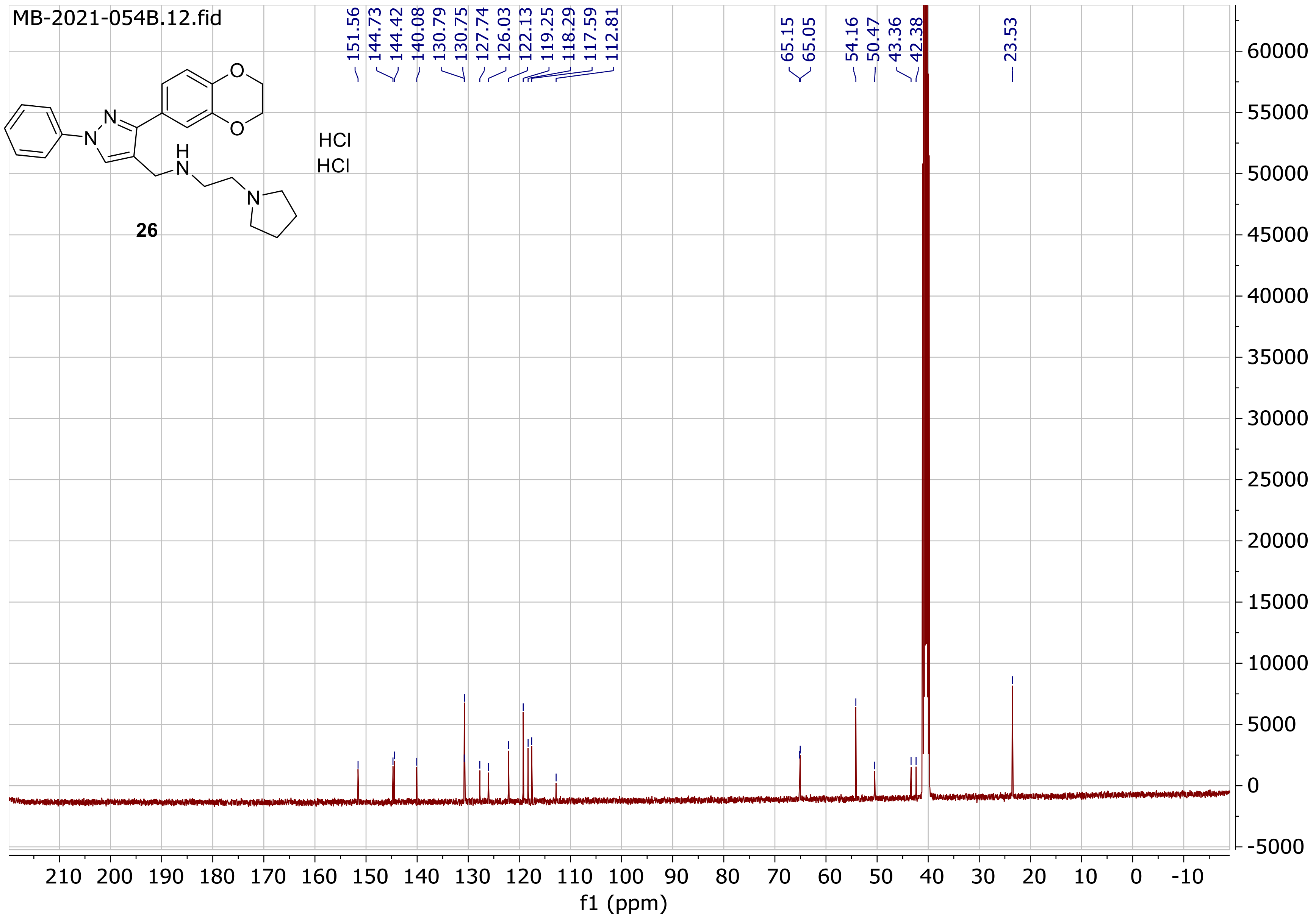
MB-2021-054B.12.fid
151.56
144.73
144.42
140.08
130.79
130.75
127.74
126.03
122.13
119.25
118.29
117.59
112.81
65.15
65.05
54.16
50.47
43.36
42.38
23.53
HCl
HCl
26
f1 (ppm)

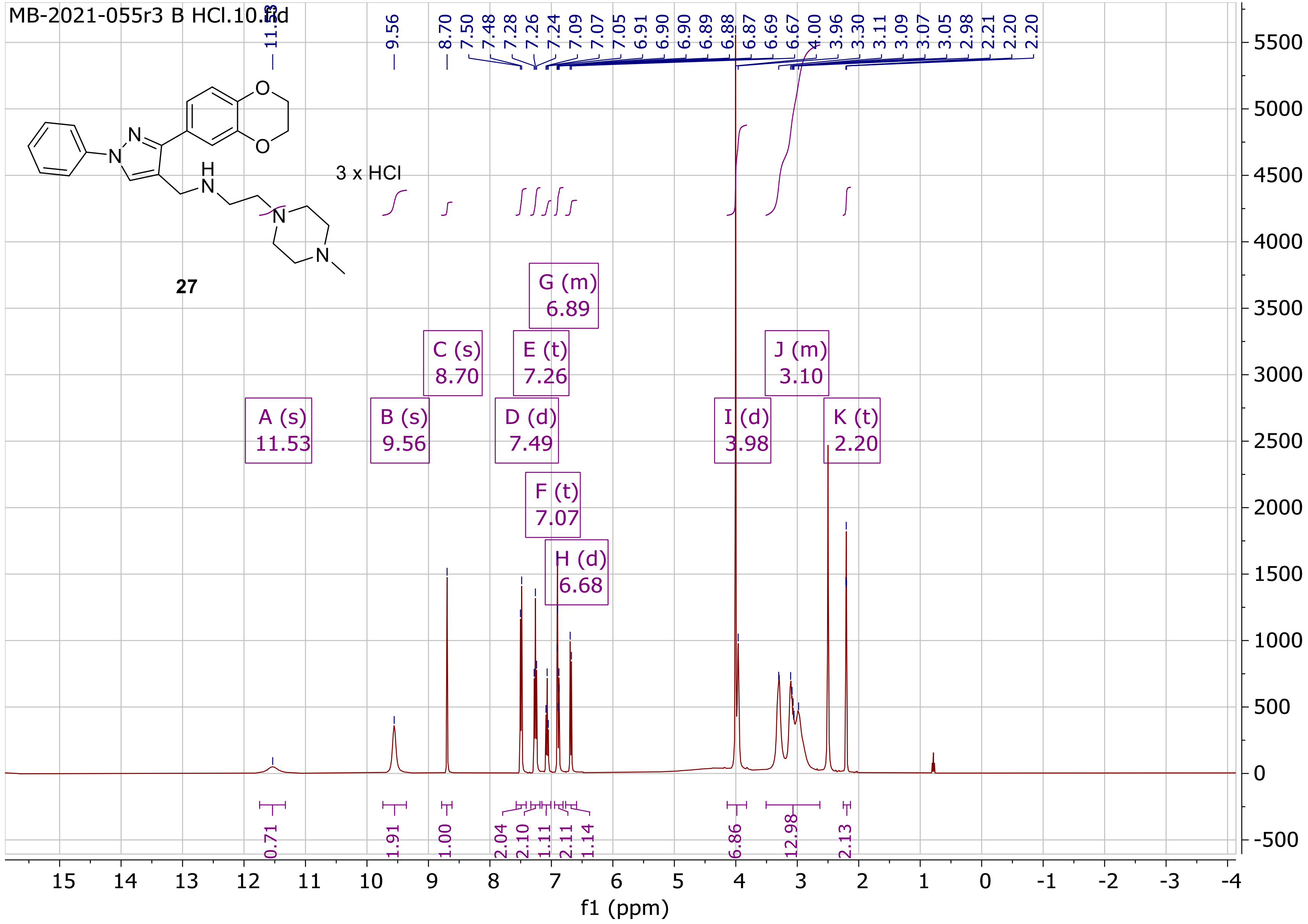

MB-2021-055r3 B HCl.10.fid
3 x HCl
27
A (s) 11.53
B (s) 9.56
C (s) 8.70
D (d) 7.49
E (t) 7.26
F (t) 7.07
G (m) 6.89
H (d) 6.68
I (d) 3.98
J (m) 3.10
K (t) 2.20
0.71
1.91
1.00
2.04
2.10
1.11
2.11
1.14
6.86
12.98
2.13
f1 (ppm)

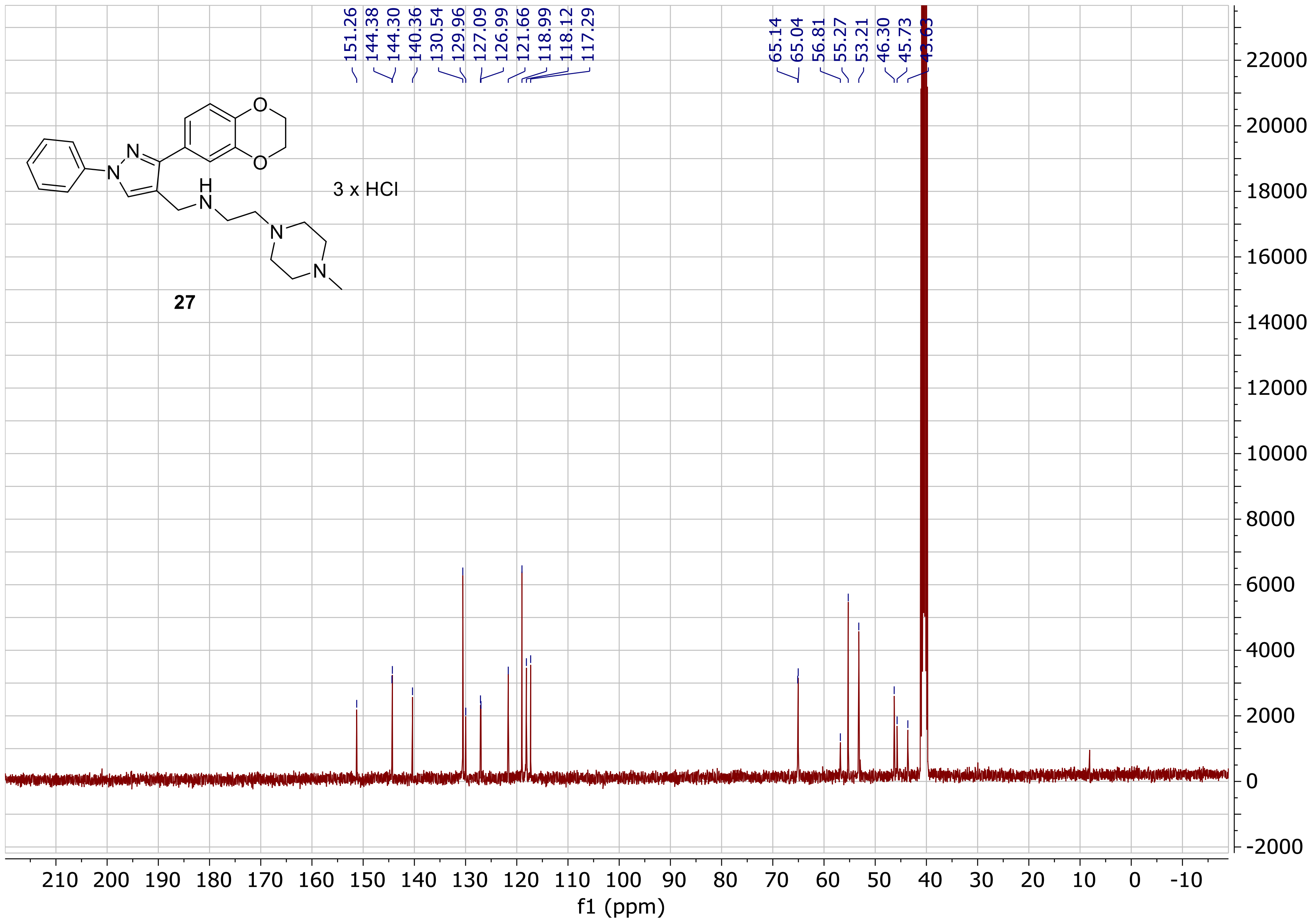

151.26
144.38
144.30
140.36
130.54
129.96
127.09
126.99
121.66
118.99
118.12
117.29
65.14
65.04
56.81
55.27
53.21
46.30
45.73
3 x HCl
27
210 200 190 180 170 160 150 140 130 120 110 100 90 80 70 60 50 40 30 20 10 0 -10
f1 (ppm)
22000
20000
18000
16000
14000
12000
10000
8000
6000
4000
2000
0
-2000

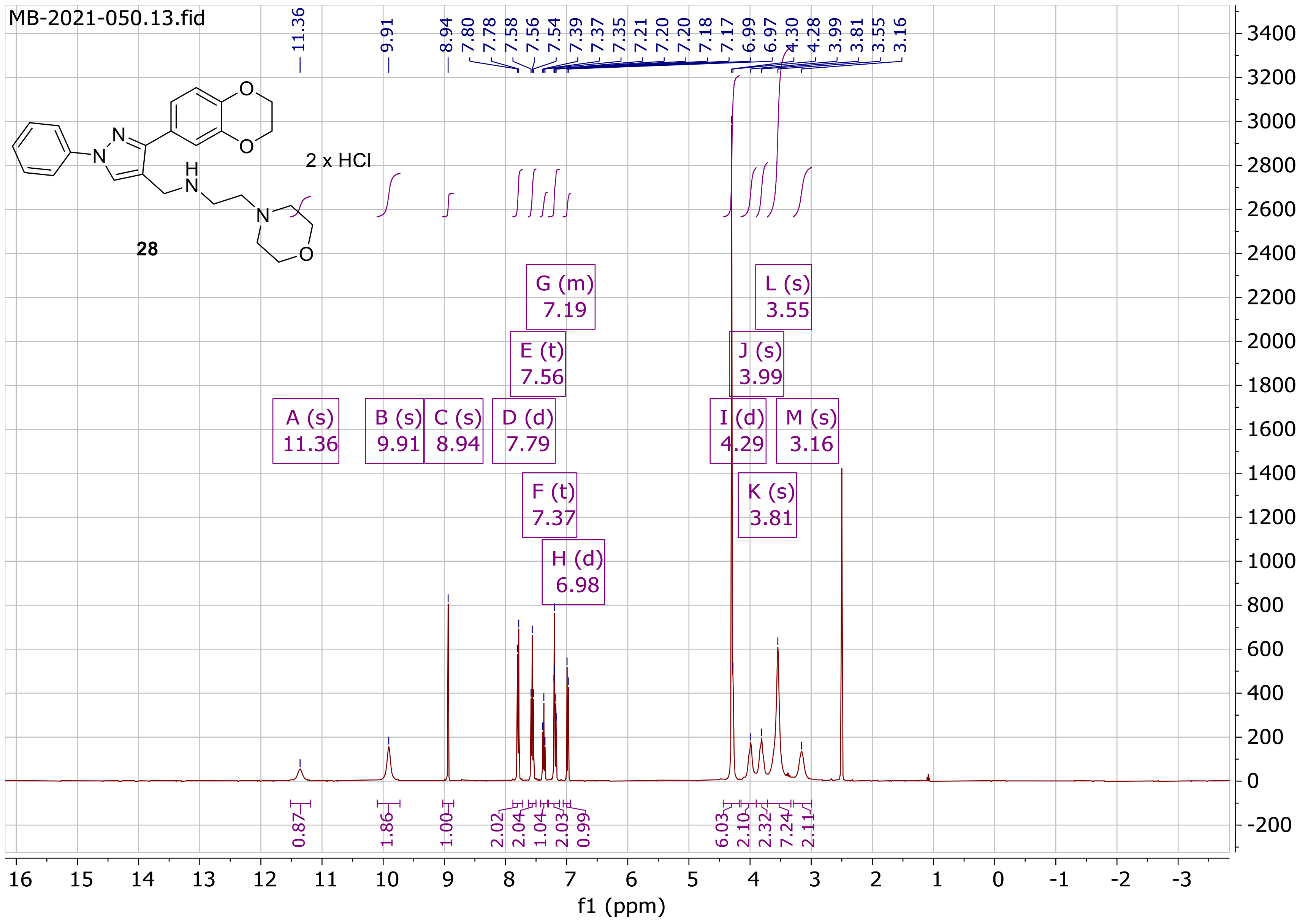
MB-2021-050.13.fid
11.36
9.91
8.94
7.80
7.78
7.58
7.56
7.54
7.39
7.37
7.35
7.21
7.20
7.20
7.18
7.17
6.99
6.97
4.30
4.28
3.99
3.81
3.55
3.16
2 x HCl
28
A (s) 11.36
B (s) 9.91
C (s) 8.94
D (d) 7.79
E (t) 7.56
F (t) 7.37
G (m) 7.19
H (d) 6.98
I (d) 4.29
J (s) 3.99
K (s) 3.81
L (s) 3.55
M (s) 3.16
0.87
1.86
1.00
2.02
2.04
1.04
2.03
0.99
6.03
2.10
2.32
7.24
2.11
f1 (ppm)

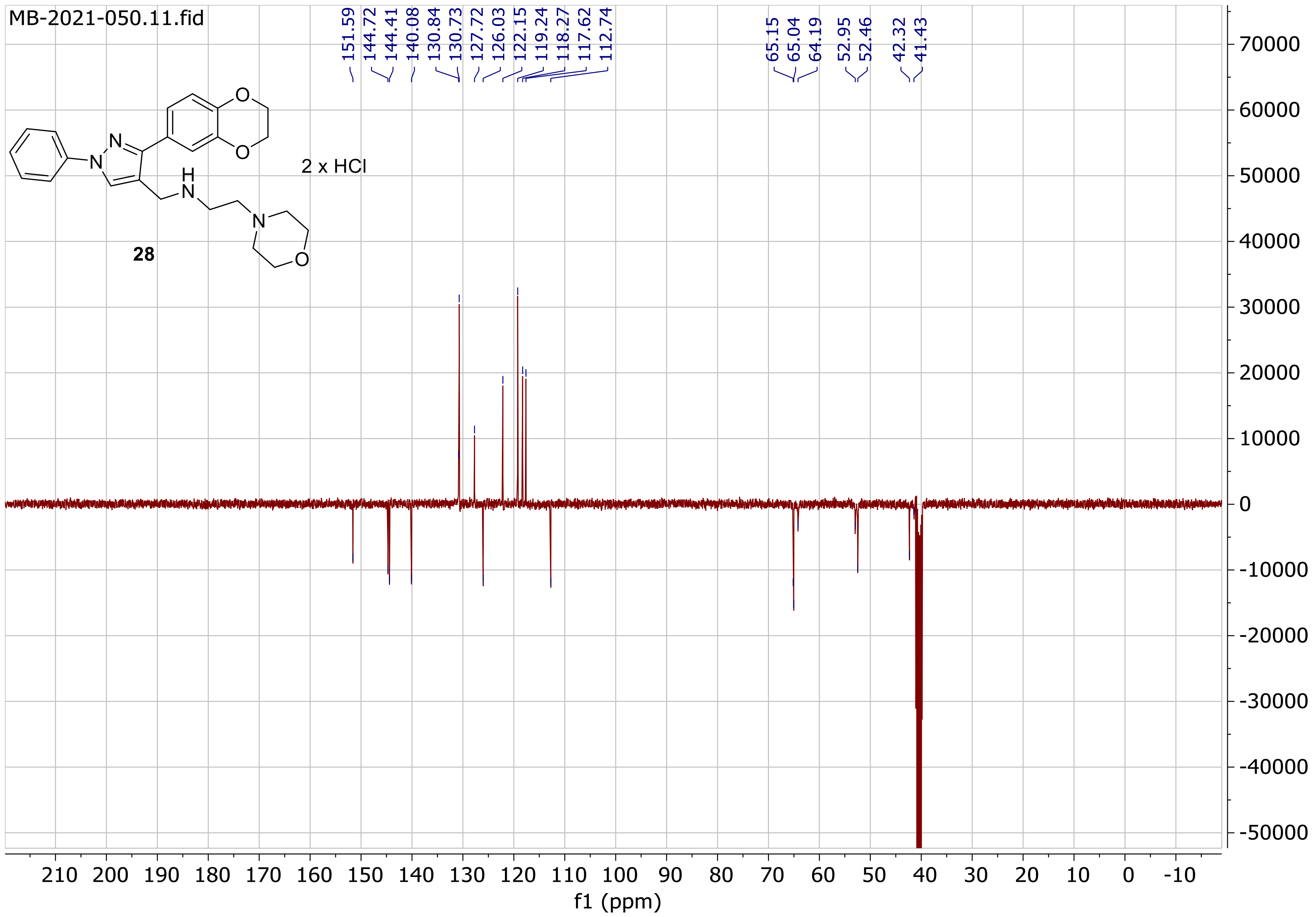
MB-2021-050.11.fid
151.59
144.72
144.41
140.08
130.84
130.73
127.72
126.03
122.15
119.24
118.27
117.62
112.74
65.15
65.04
64.19
52.95
52.46
42.32
41.43
2 x HCl
28
f1 (ppm)

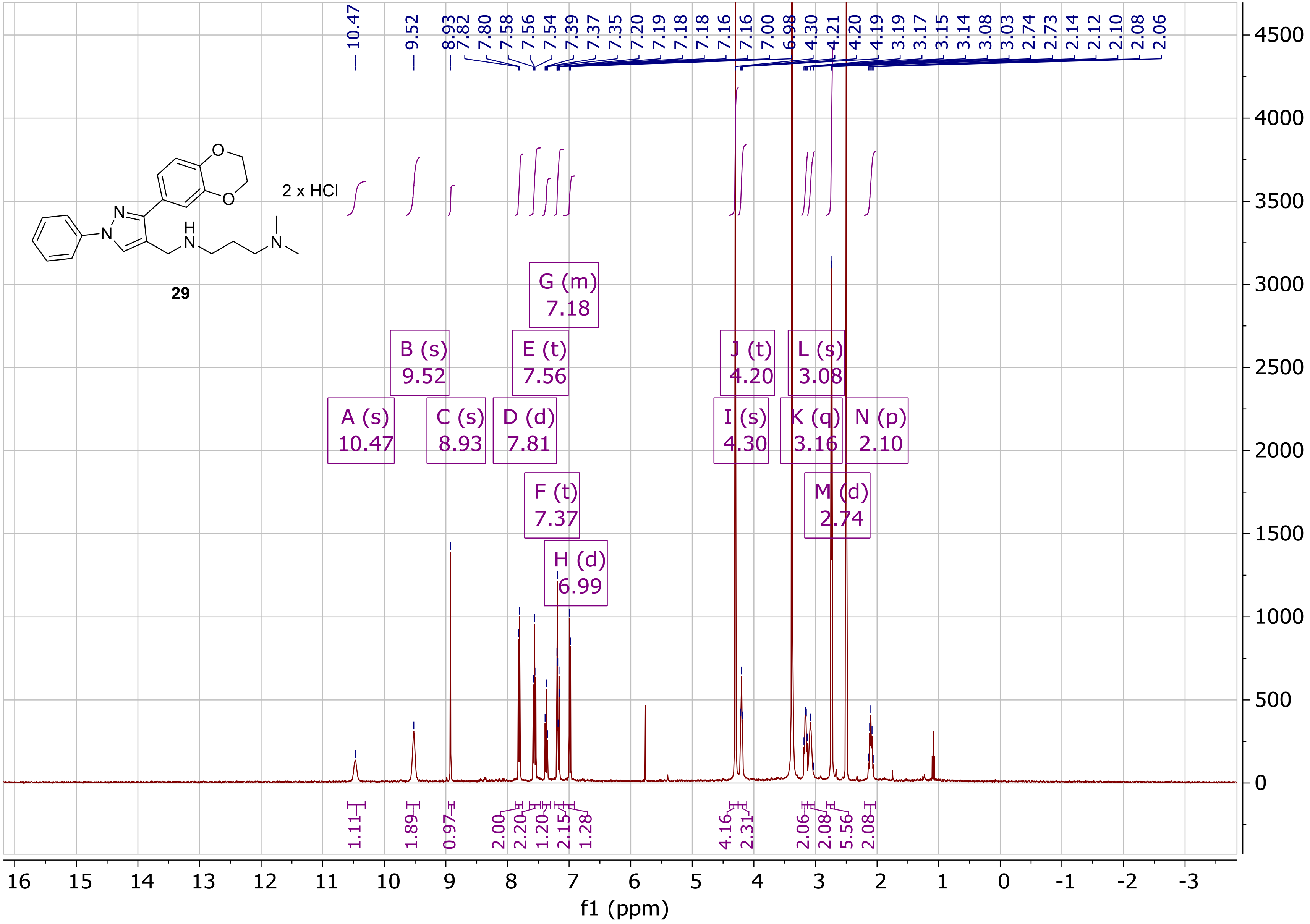

2 x HCl
29
10.47
9.52
8.93
7.82
7.80
7.58
7.56
7.54
7.39
7.37
7.35
7.20
7.19
7.18
7.18
7.16
7.16
7.00
6.98
4.30
4.21
4.20
4.19
3.19
3.17
3.15
3.14
3.08
3.03
2.74
2.73
2.14
2.12
2.10
2.08
2.06
A (s) 10.47
B (s) 9.52
C (s) 8.93
D (d) 7.81
E (t) 7.56
F (t) 7.37
G (m) 7.18
H (d) 6.99
I (s) 4.30
J (t) 4.20
K (q) 3.16
L (s) 3.08
M (d) 2.74
N (p) 2.10
1.11
1.89
0.97
2.00
2.20
1.20
2.15
1.28
4.16
2.31
2.06
2.08
5.56
2.08
f1 (ppm)

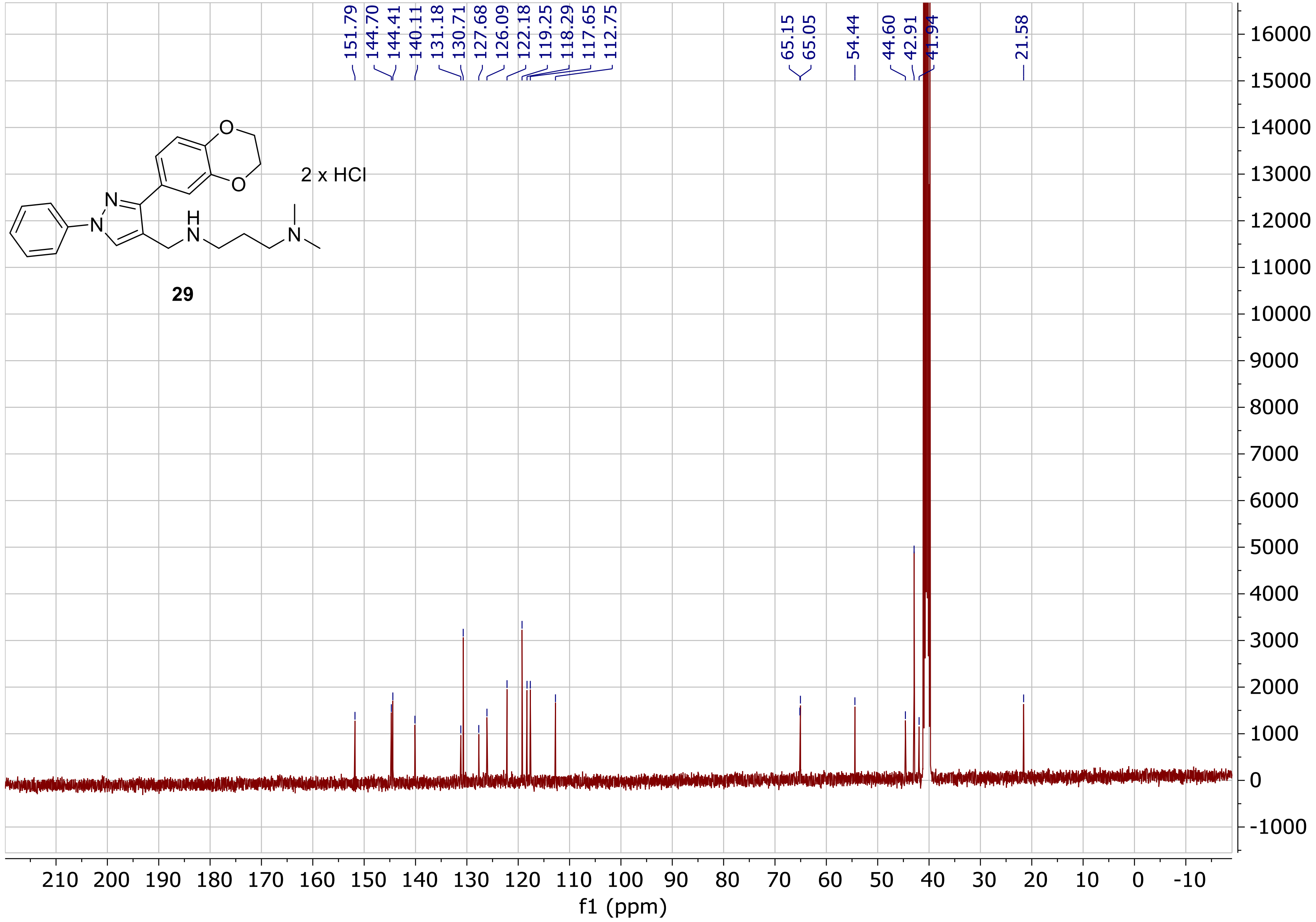
151.79
144.70
144.41
140.11
131.18
130.71
127.68
126.09
122.18
119.25
118.29
117.65
112.75
65.15
65.05
54.44
44.60
42.91
41.94
21.58
2 x HCl
29
16000
15000
14000
13000
12000
11000
10000
9000
8000
7000
6000
5000
4000
3000
2000
1000
0
-1000
210 200 190 180 170 160 150 140 130 120 110 100 90 80 70 60 50 40 30 20 10 0 -10
f1 (ppm)

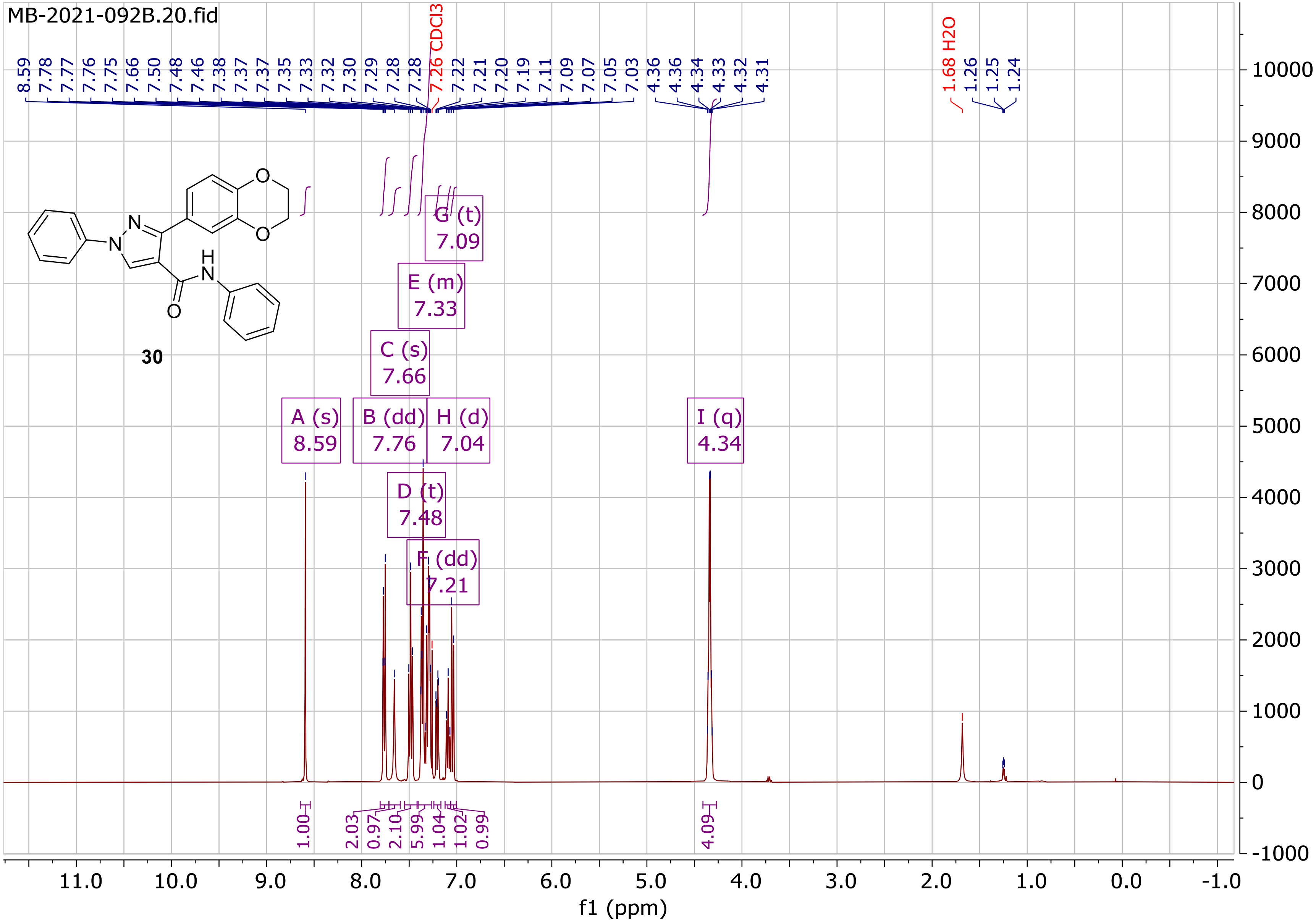
MB-2021-092B.20.fid
8.59
7.78
7.77
7.76
7.75
7.66
7.50
7.48
7.46
7.38
7.37
7.37
7.35
7.33
7.32
7.30
7.29
7.28
7.28
7.26 CDCl3
7.22
7.21
7.20
7.19
7.11
7.09
7.07
7.05
7.03
4.36
4.36
4.34
4.33
4.32
4.31
1.68 H2O
1.26
1.25
1.24
30
G (t) 7.09
E (m) 7.33
C (s) 7.66
A (s) 8.59
B (dd) 7.76
H (d) 7.04
I (q) 4.34
D (t) 7.48
F (dd) 7.21
1.00
2.03
0.97
2.10
5.99
1.04
1.02
0.99
4.09
10000
9000
8000
7000
6000
5000
4000
3000
2000
1000
0
-1000
11.0
10.0
9.0
8.0
7.0
6.0
5.0
4.0
3.0
2.0
1.0
0.0
-1.0
f1 (ppm)

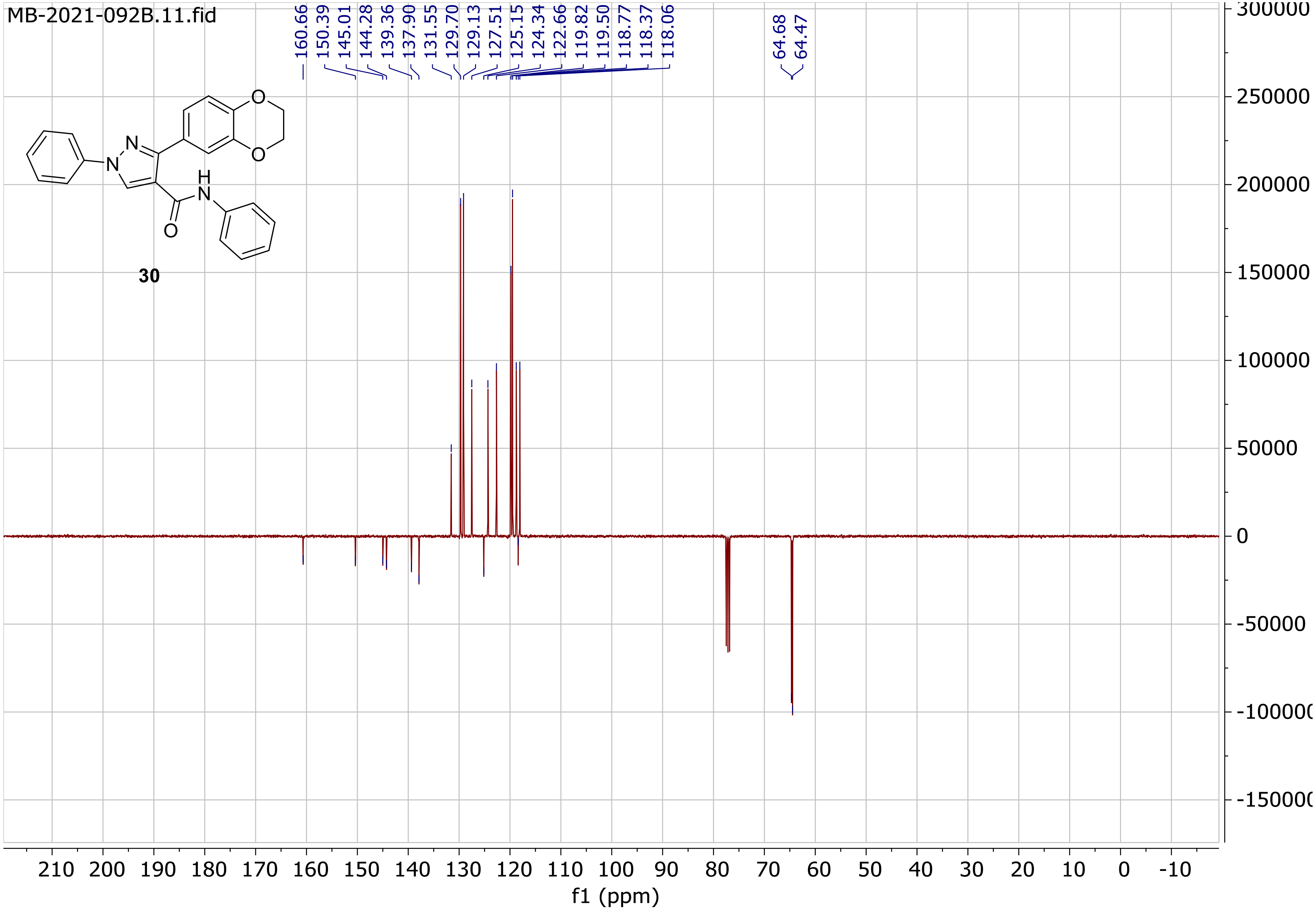
MB-2021-092B.11.fid
160.66
150.39
145.01
144.28
139.36
137.90
131.55
129.70
129.13
127.51
125.15
124.34
122.66
119.82
119.50
118.77
118.37
118.06
64.68
64.47
30
f1 (ppm)

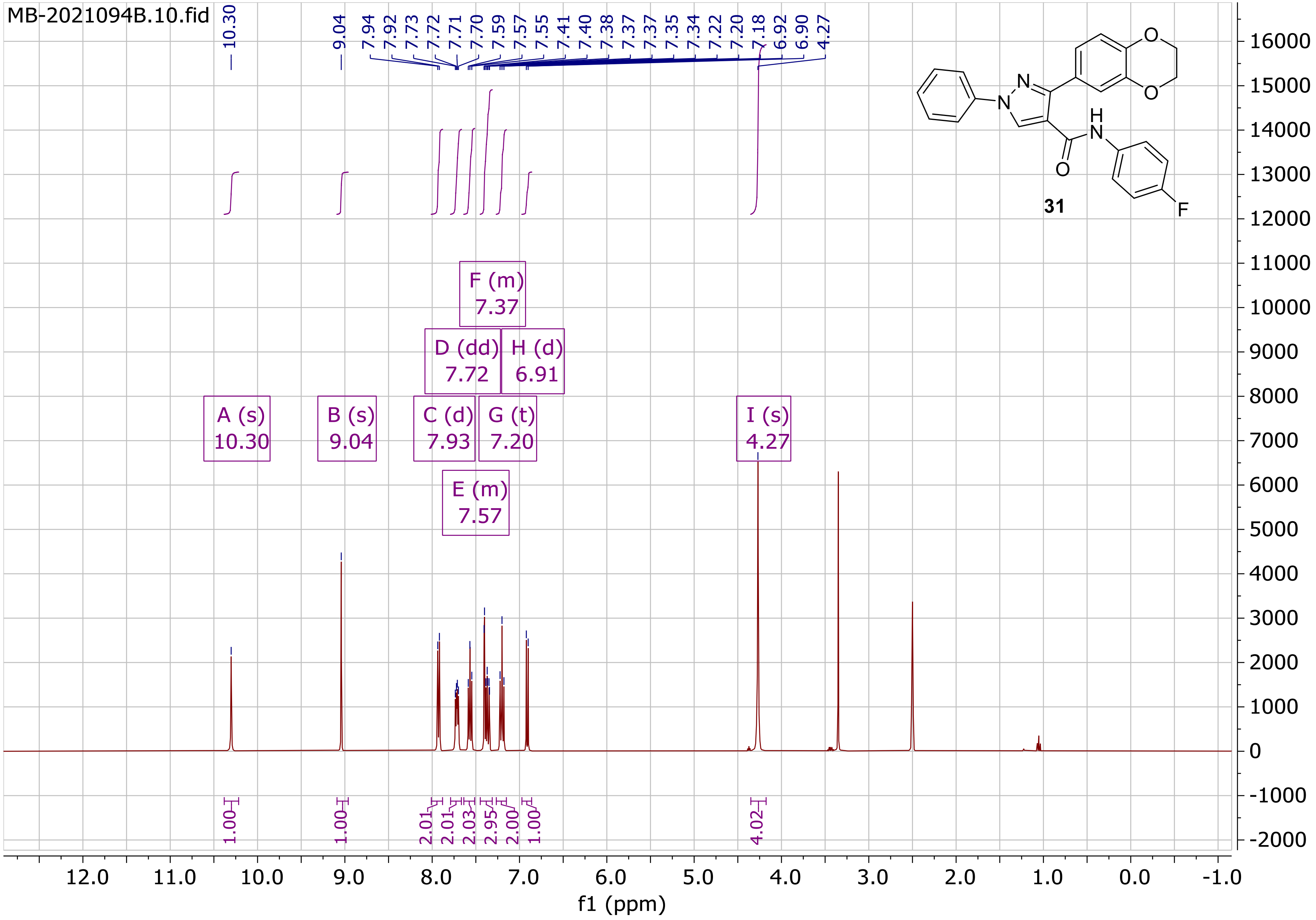
MB-2021094B.10.fid
10.30
9.04
7.94
7.92
7.73
7.72
7.71
7.70
7.59
7.57
7.55
7.41
7.40
7.38
7.37
7.37
7.35
7.34
7.22
7.20
7.18
6.92
6.90
4.27
A (s) 10.30
B (s) 9.04
C (d) 7.93
D (dd) 7.72
E (m) 7.57
F (m) 7.37
G (t) 7.20
H (d) 6.91
I (s) 4.27
1.00
1.00
2.01
2.01
2.03
2.95
2.00
1.00
4.02
31
f1 (ppm)

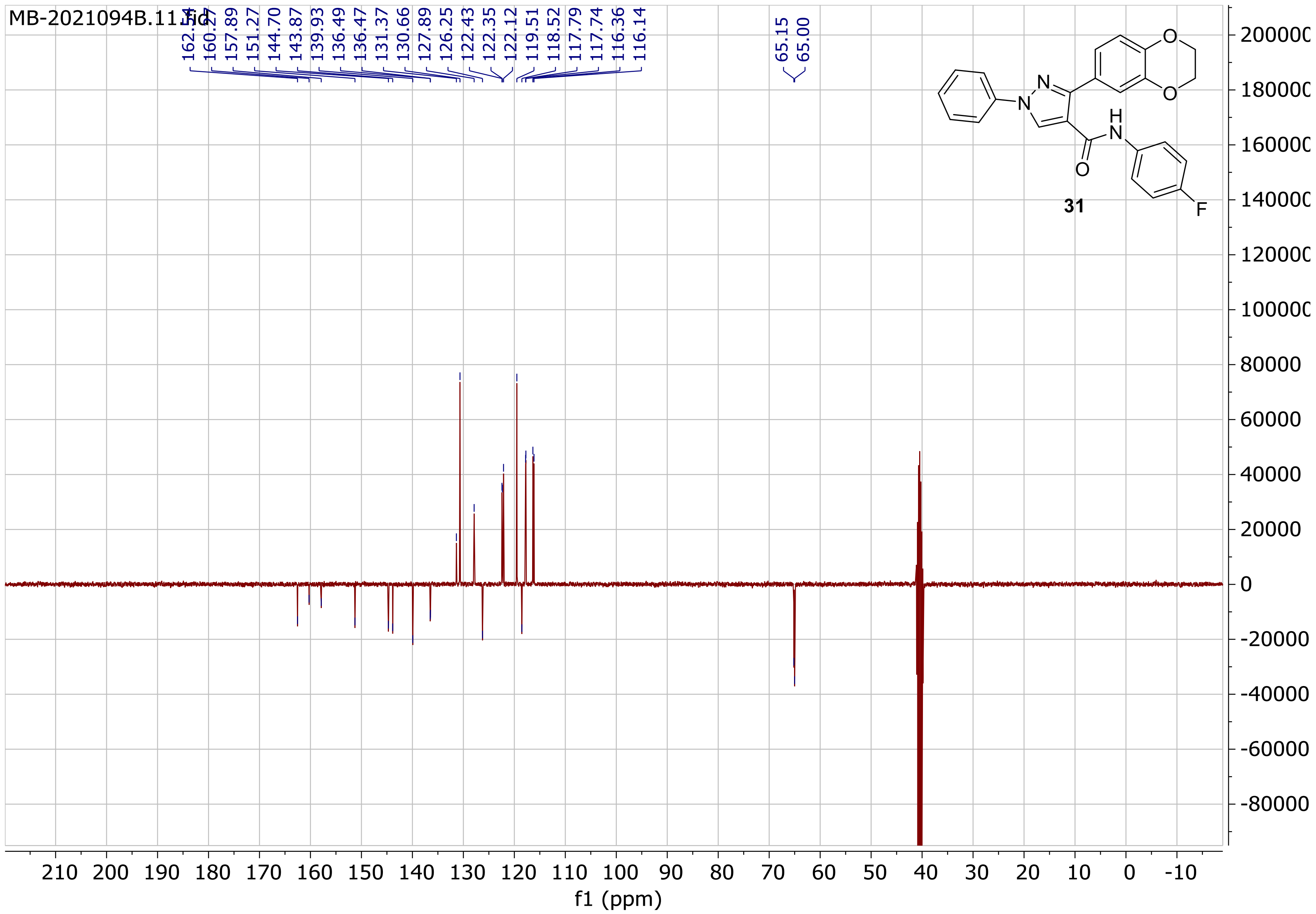
MB-2021094B.11.fid
162.54
160.27
157.89
151.27
144.70
143.87
139.93
136.49
136.47
131.37
130.66
127.89
126.25
122.43
122.35
122.12
119.51
118.52
117.79
117.74
116.36
116.14
65.15
65.00
N
N
O
O
H
N
O
F
31
200000
180000
160000
140000
120000
100000
80000
60000
40000
20000
0
-20000
-40000
-60000
-80000
210
200
190
180
170
160
150
140
130
120
110
100
90
80
70
60
50
40
30
20
10
0
-10
f1 (ppm)

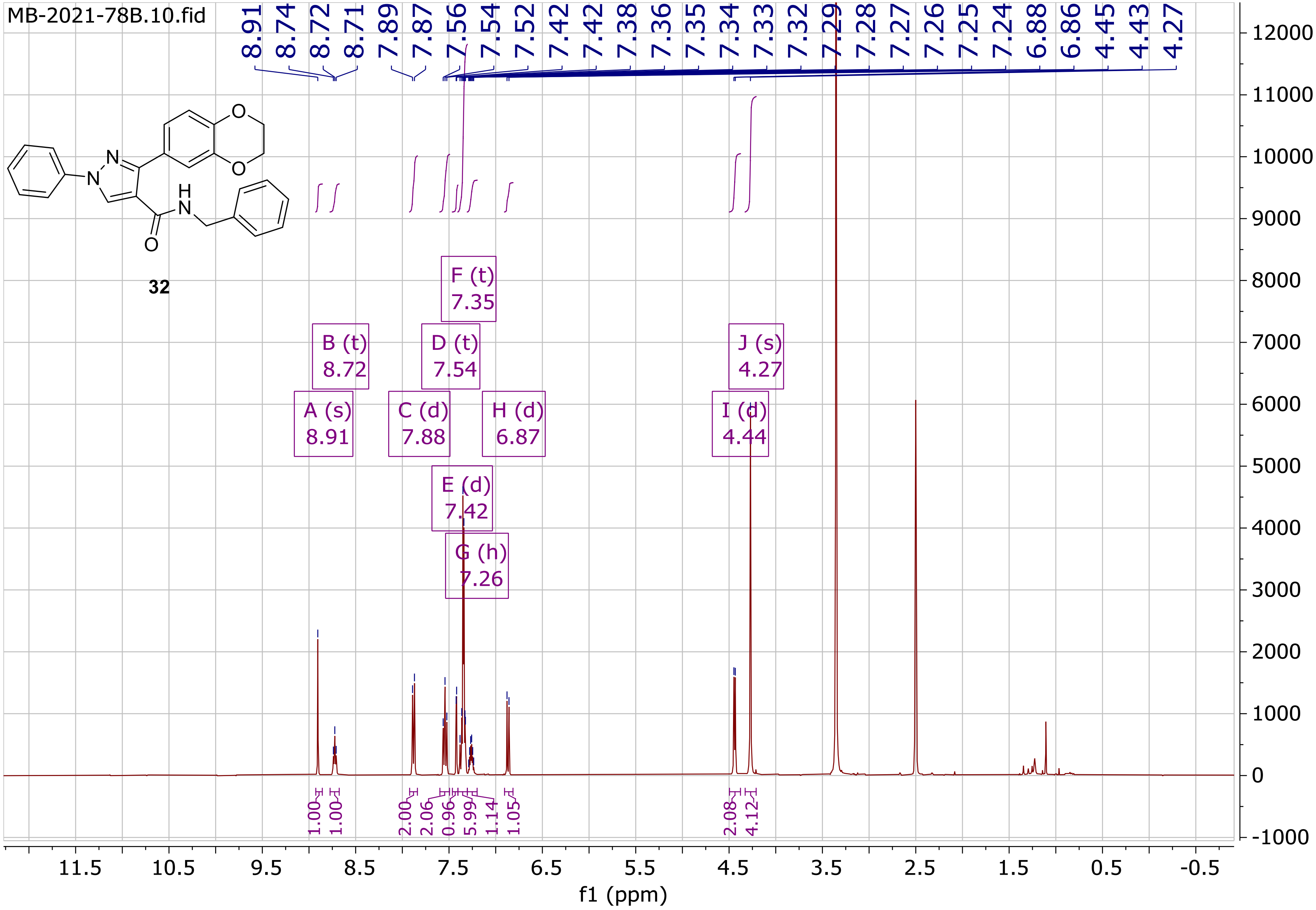
MB-2021-78B.10.fid
8.91 8.74 8.72 8.71 7.89 7.87 7.56 7.54 7.52 7.42 7.42 7.38 7.36 7.35 7.34 7.33 7.32 7.29 7.28 7.27 7.26 7.25 7.24 6.88 6.86 4.45 4.43 4.27
A (s) 8.91
B (t) 8.72
C (d) 7.88
D (t) 7.54
E (d) 7.42
F (t) 7.35
G (h) 7.26
H (d) 6.87
I (d) 4.44
J (s) 4.27
1.00 1.00 2.00 2.06 0.96 5.99 1.14 1.05 2.08 4.12
f1 (ppm)
11.5 10.5 9.5 8.5 7.5 6.5 5.5 4.5 3.5 2.5 1.5 0.5 -0.5
12000 11000 10000 9000 8000 7000 6000 5000 4000 3000 2000 1000 0 -1000

32

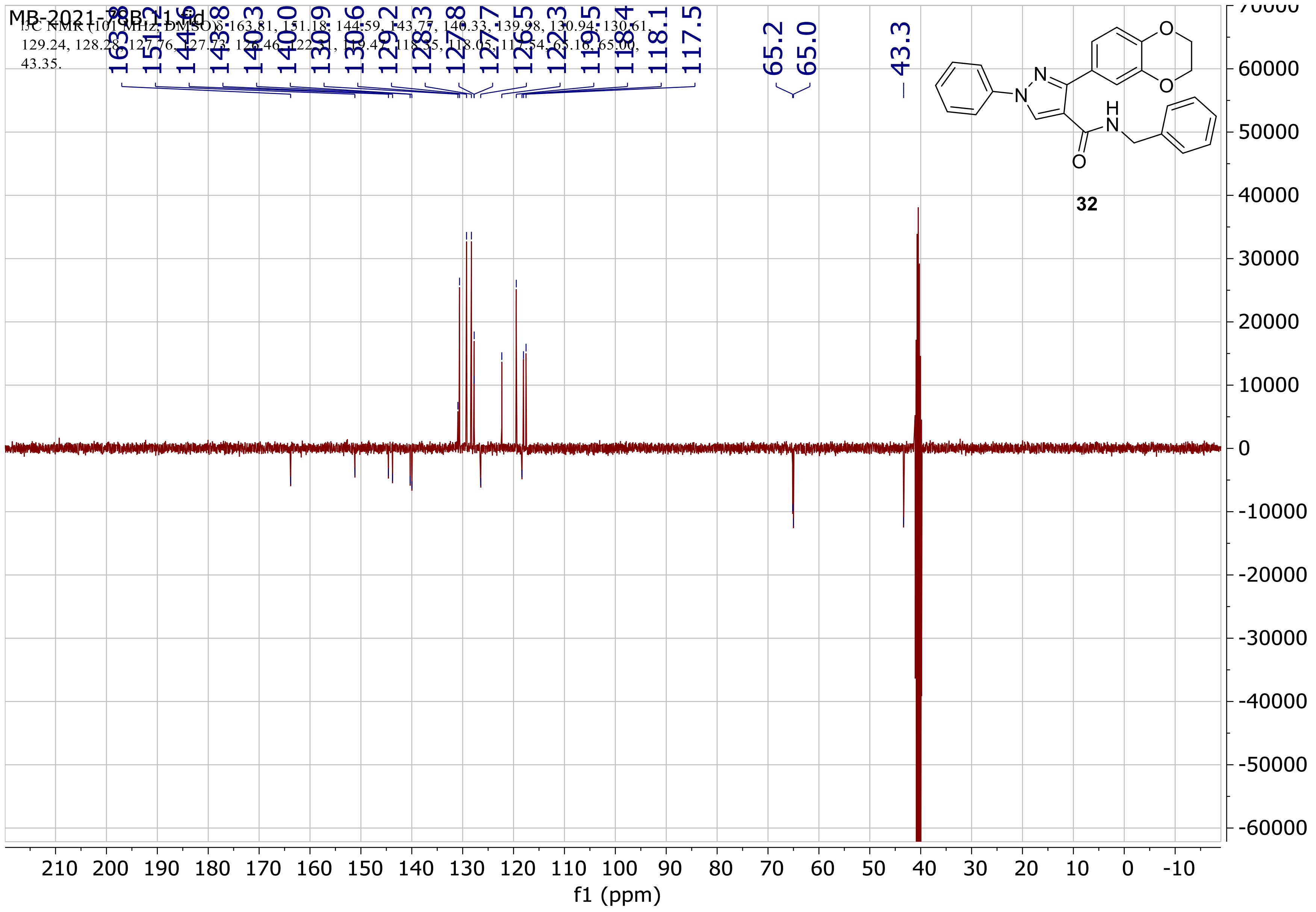

MB-2021-78B.11.fid
13C NMR (101 MHz, DMSO) δ 163.81, 151.18, 144.59, 143.77, 140.33, 139.98, 130.94, 130.61, 129.24, 128.28, 127.76, 127.73, 126.46, 122.31, 119.47, 118.35, 118.05, 117.54, 65.16, 65.00, 43.35.
163.8
151.2
144.6
143.8
140.3
140.0
130.9
130.6
129.2
128.3
127.8
127.7
126.5
122.3
119.5
118.4
118.1
117.5
65.2
65.0
43.3
32
70000
60000
50000
40000
30000
20000
10000
0
-10000
-20000
-30000
-40000
-50000
-60000
210 200 190 180 170 160 150 140 130 120 110 100 90 80 70 60 50 40 30 20 10 0 -10
f1 (ppm)

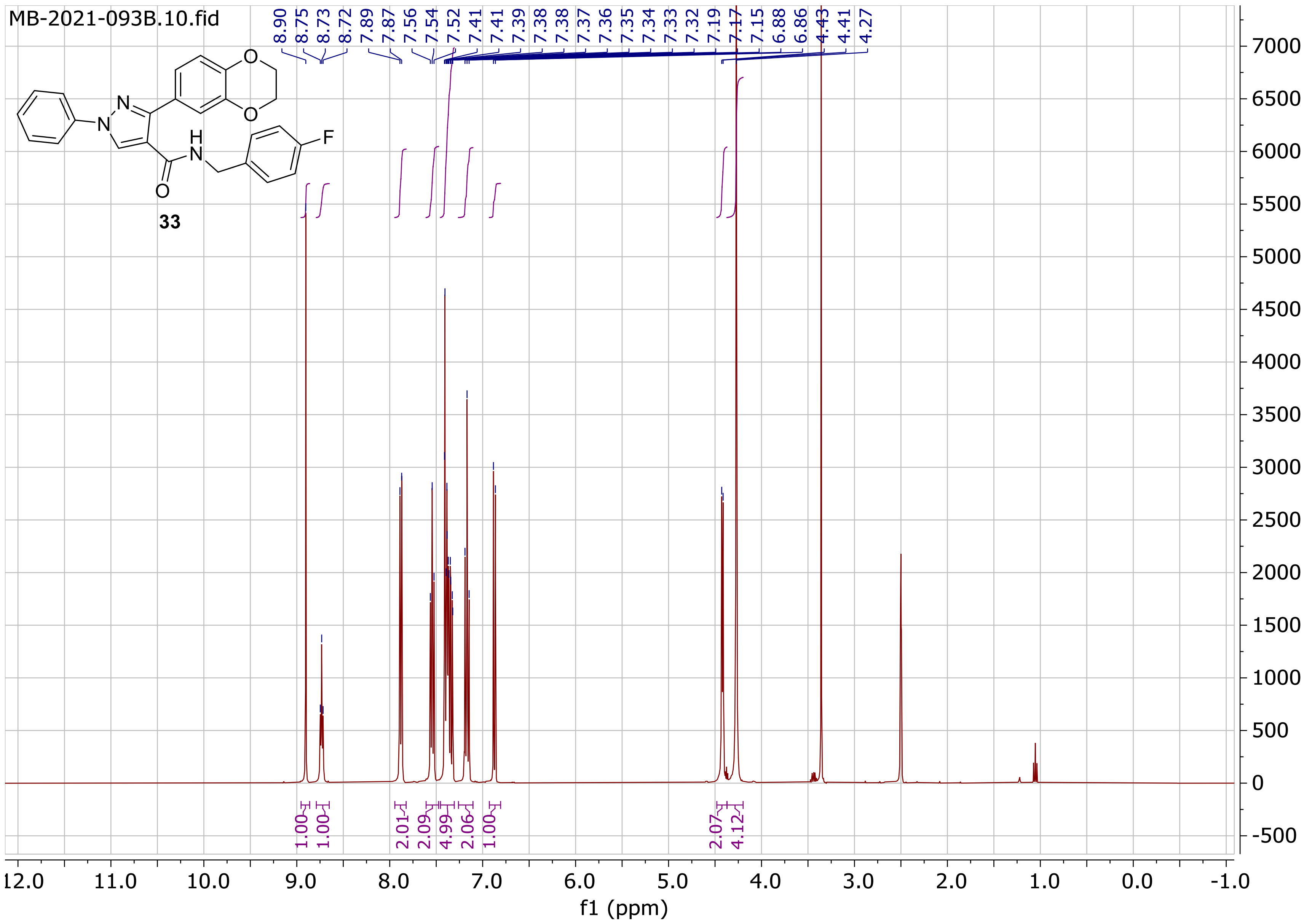
MB-2021-093B.10.fid
8.90
8.75
8.73
8.72
7.89
7.87
7.56
7.54
7.52
7.41
7.41
7.39
7.38
7.38
7.37
7.36
7.35
7.34
7.33
7.32
7.19
7.17
7.15
6.88
6.86
4.43
4.41
4.27
33
1.00
1.00
2.01
2.09
4.99
2.06
1.00
2.07
4.12
f1 (ppm)

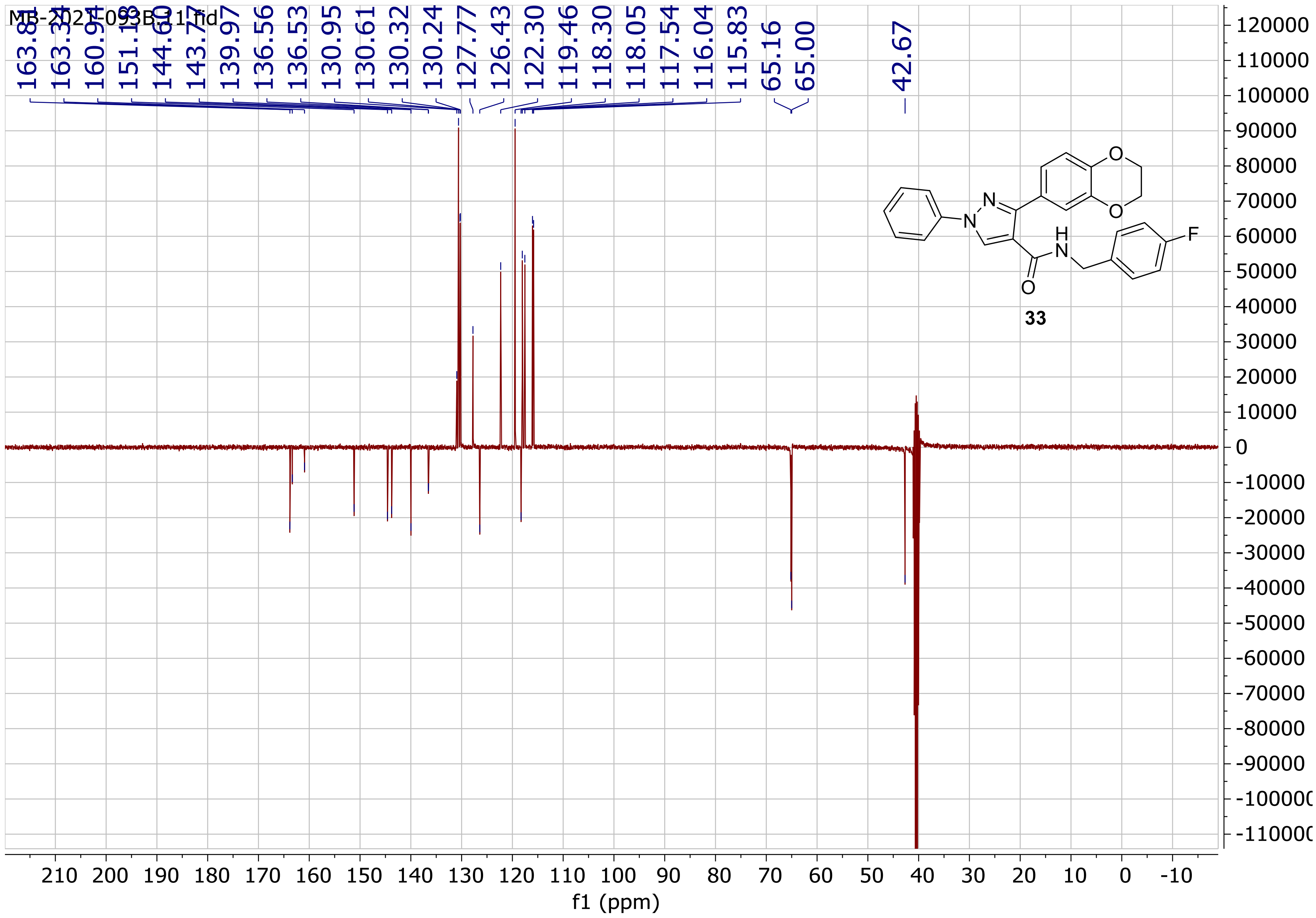

163.81
163.34
160.94
151.18
144.60
143.77
139.97
136.56
136.53
130.95
130.61
130.32
130.24
127.77
126.43
122.30
119.46
118.30
118.05
117.54
116.04
115.83
65.16
65.00
42.67
N
N
O
O
H
N
F
O
33
f1 (ppm)

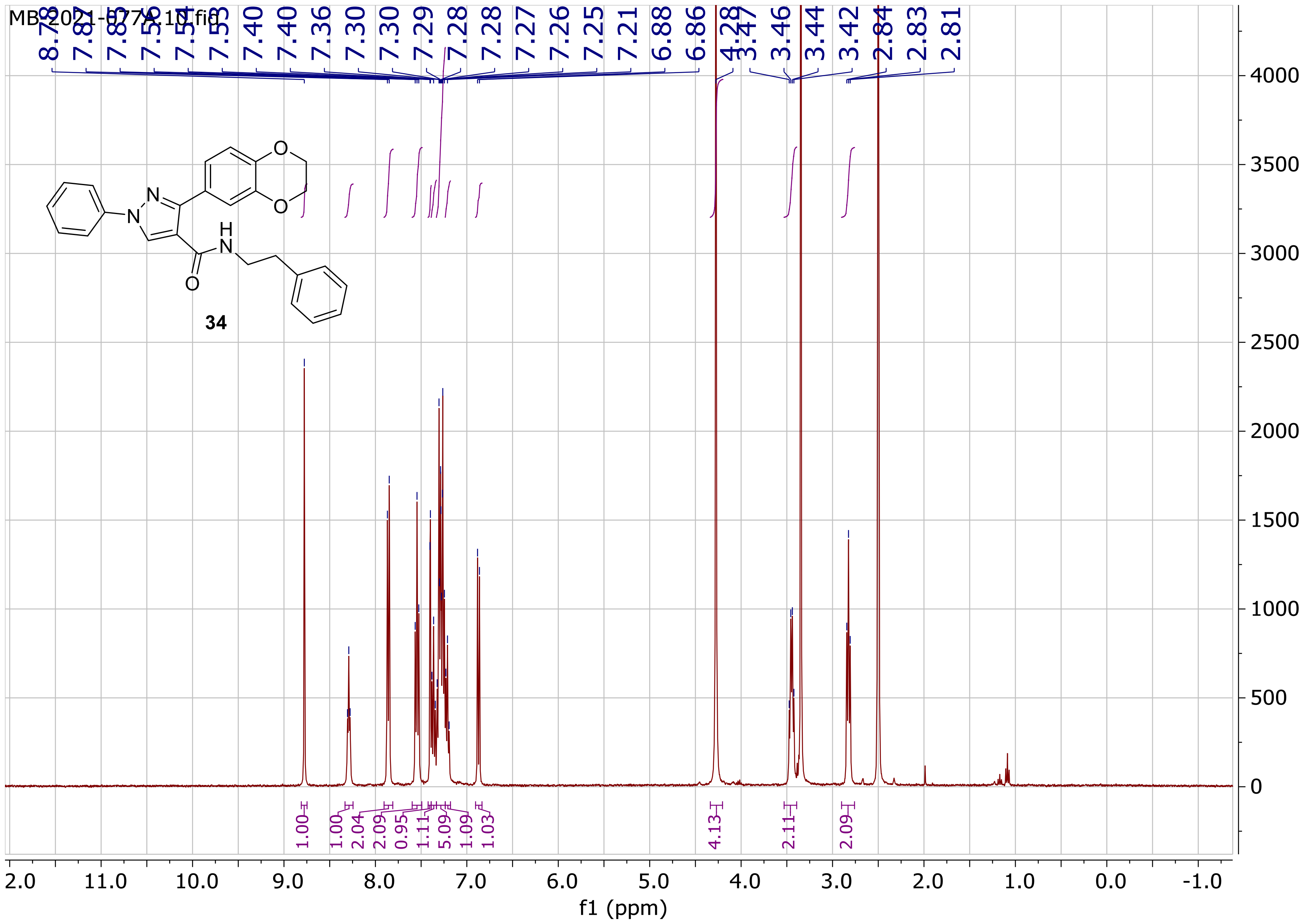
MB-2021-077A.10.fid
8.78
7.87
7.85
7.56
7.54
7.53
7.40
7.40
7.36
7.30
7.30
7.29
7.28
7.28
7.27
7.26
7.25
7.21
6.88
6.86
4.28
3.47
3.46
3.44
3.42
2.84
2.83
2.81
34
1.00
1.00
2.04
2.09
0.95
1.11
5.09
1.09
1.03
4.13
2.11
2.09
f1 (ppm)

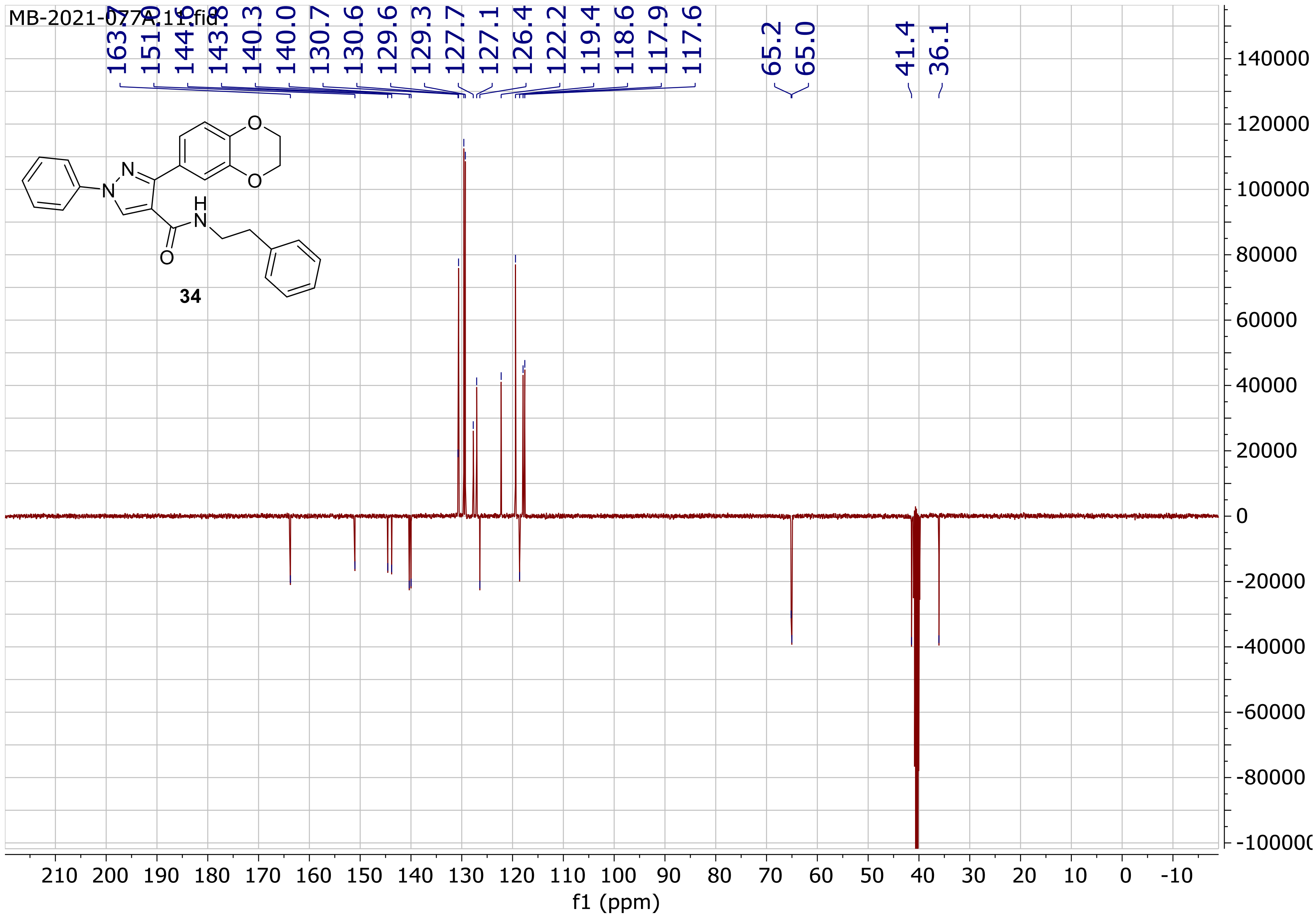
MB-2021-027A.11.fid
163.7
151.0
144.6
143.8
140.3
140.0
130.7
130.6
129.6
129.3
127.7
127.1
126.4
122.2
119.4
118.6
117.9
117.6
65.2
65.0
41.4
36.1
34
f1 (ppm)

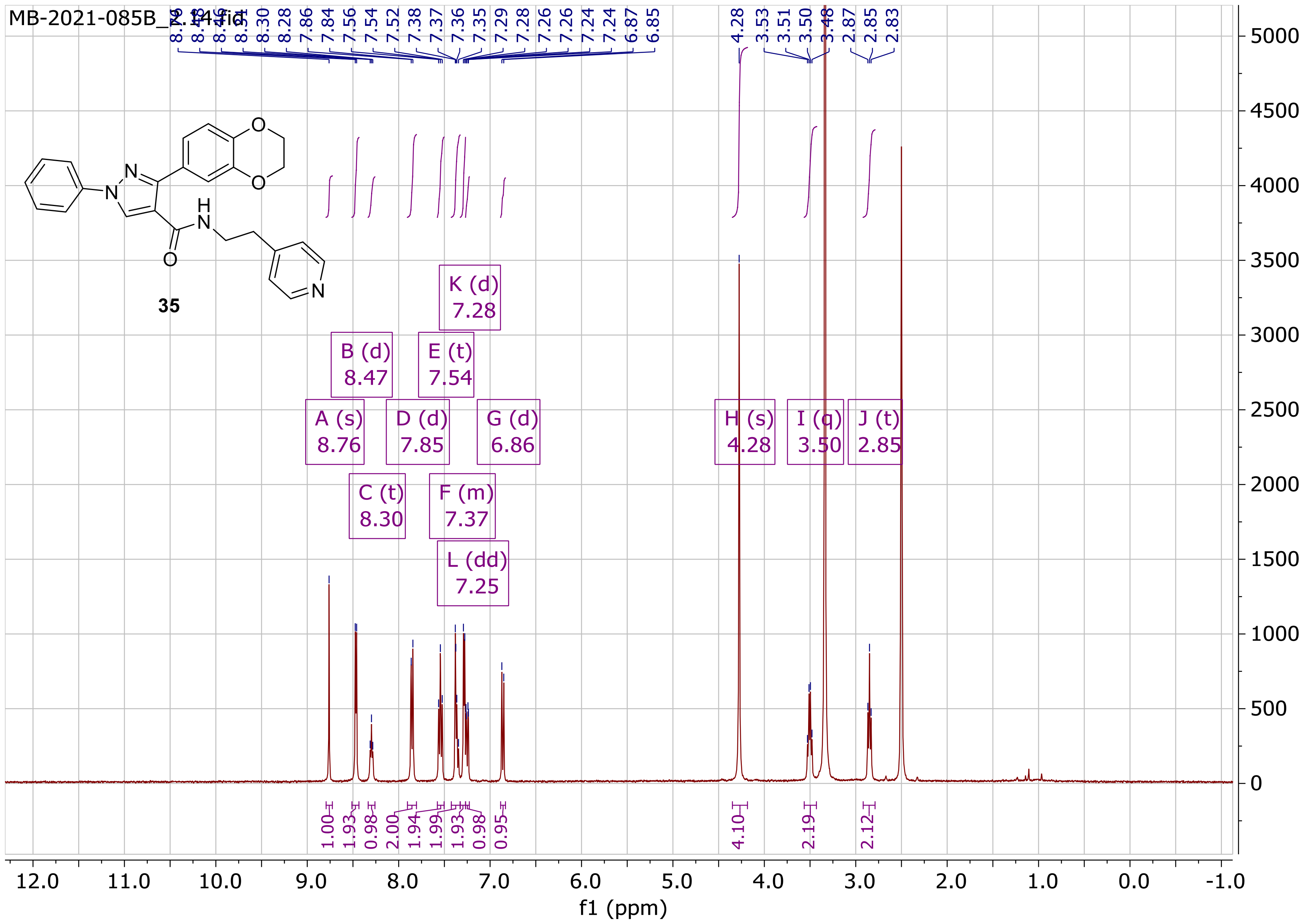

4.28
3.53
3.51
3.50
3.48
2.87
2.85
2.83
8.30
8.28
7.86
7.84
7.56
7.54
7.52
7.38
7.37
7.36
7.35
7.29
7.28
7.26
7.26
7.24
7.24
6.87
6.85
35
A (s) 8.76
B (d) 8.47
C (t) 8.30
D (d) 7.85
E (t) 7.54
F (m) 7.37
K (d) 7.28
L (dd) 7.25
G (d) 6.86
H (s) 4.28
I (q) 3.50
J (t) 2.85
1.00
1.93
0.98
2.00
1.94
1.99
1.93
0.98
0.95
4.10
2.19
2.12
f1 (ppm)

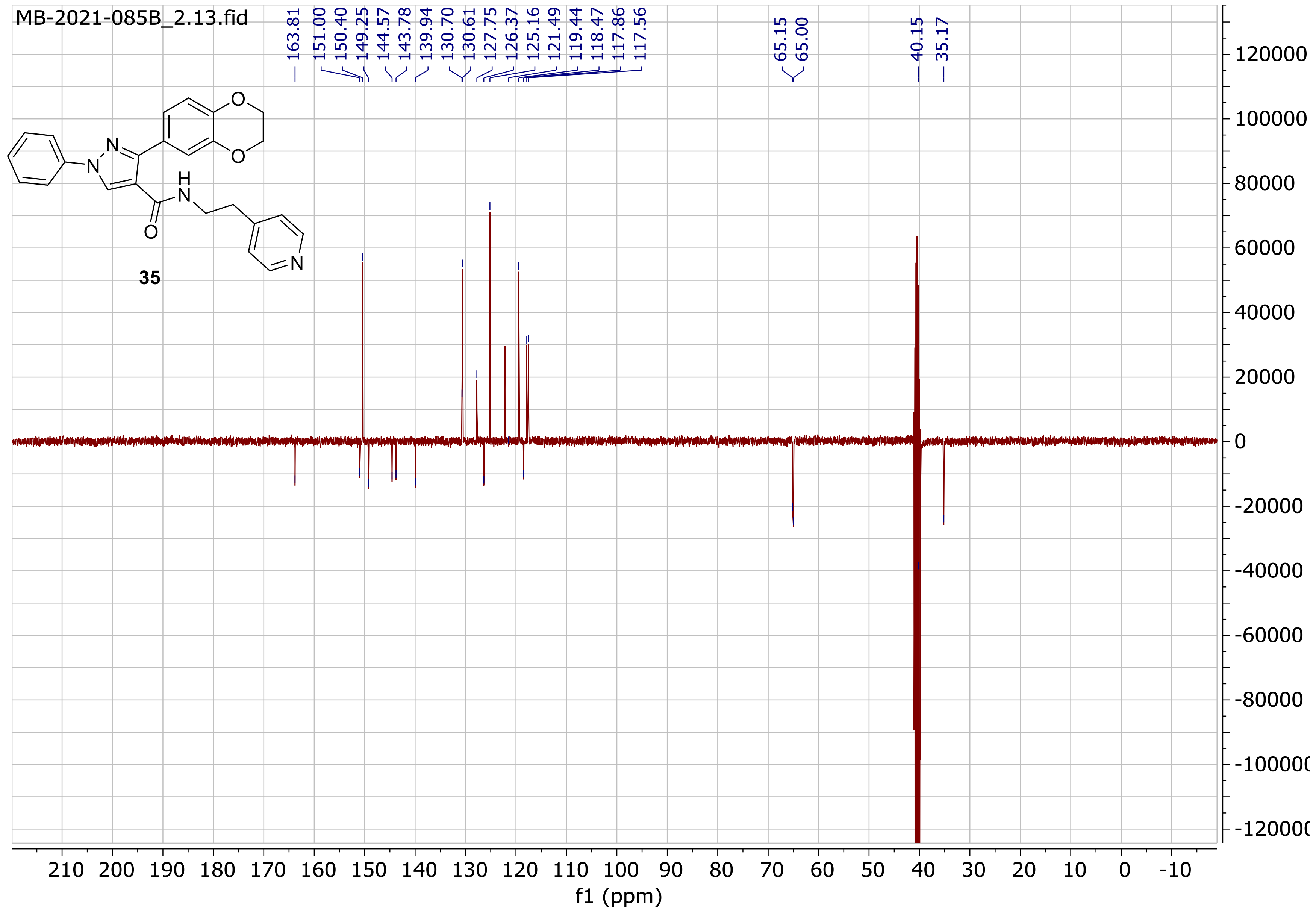

MB-2021-085B_2.13.fid
163.81
151.00
150.40
149.25
144.57
143.78
139.94
130.70
130.61
127.75
126.37
125.16
121.49
119.44
118.47
117.86
117.56
65.15
65.00
40.15
35.17
35
f1 (ppm)

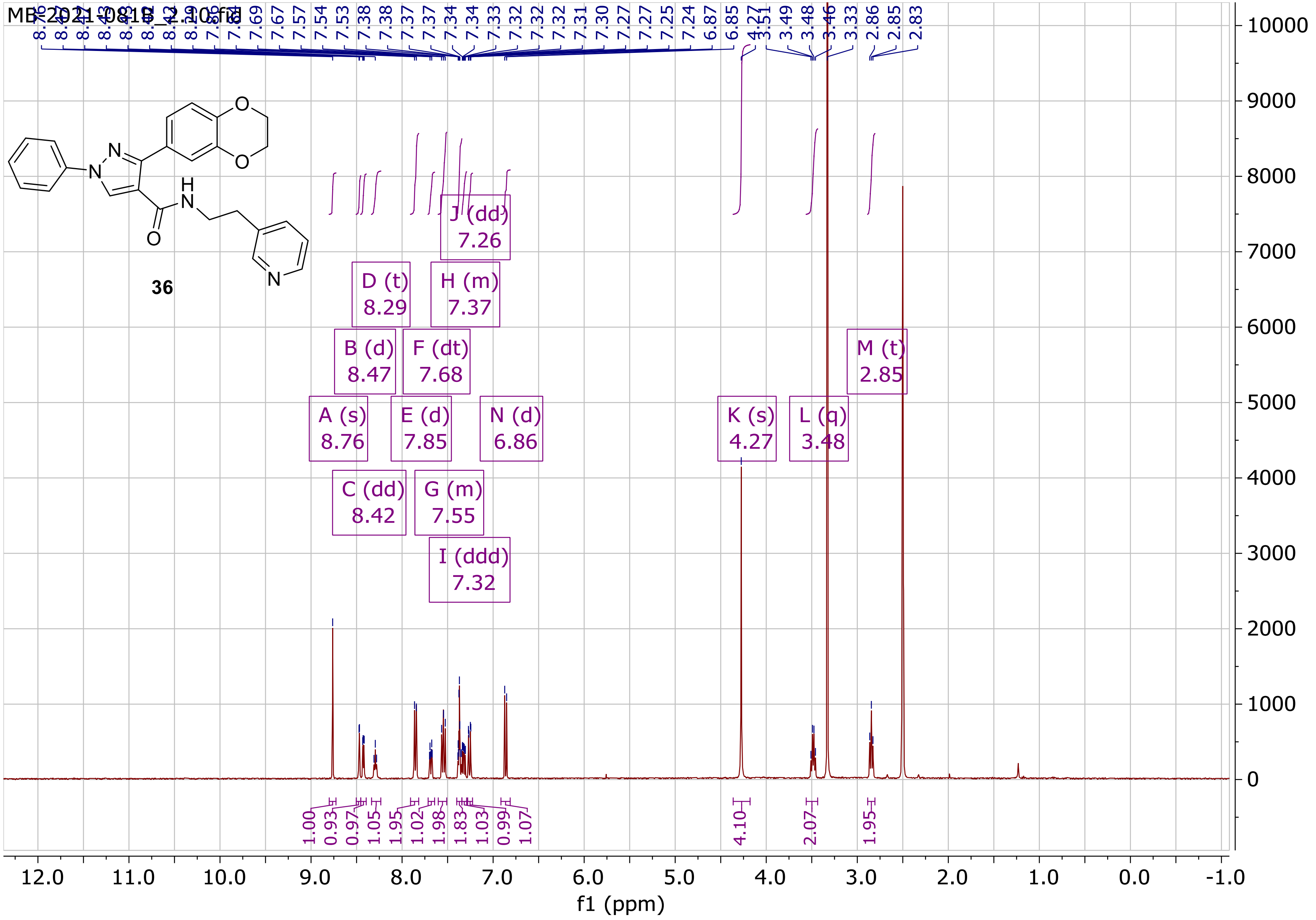

36
A (s) 8.76
B (d) 8.47
C (dd) 8.42
D (t) 8.29
E (d) 7.85
F (dt) 7.68
G (m) 7.55
H (m) 7.37
I (ddd) 7.32
J (dd) 7.26
N (d) 6.86
K (s) 4.27
L (q) 3.48
M (t) 2.85
1.00
0.93
0.97
1.05
1.95
1.02
1.98
1.83
1.03
0.99
1.07
4.10
2.07
1.95
f1 (ppm)

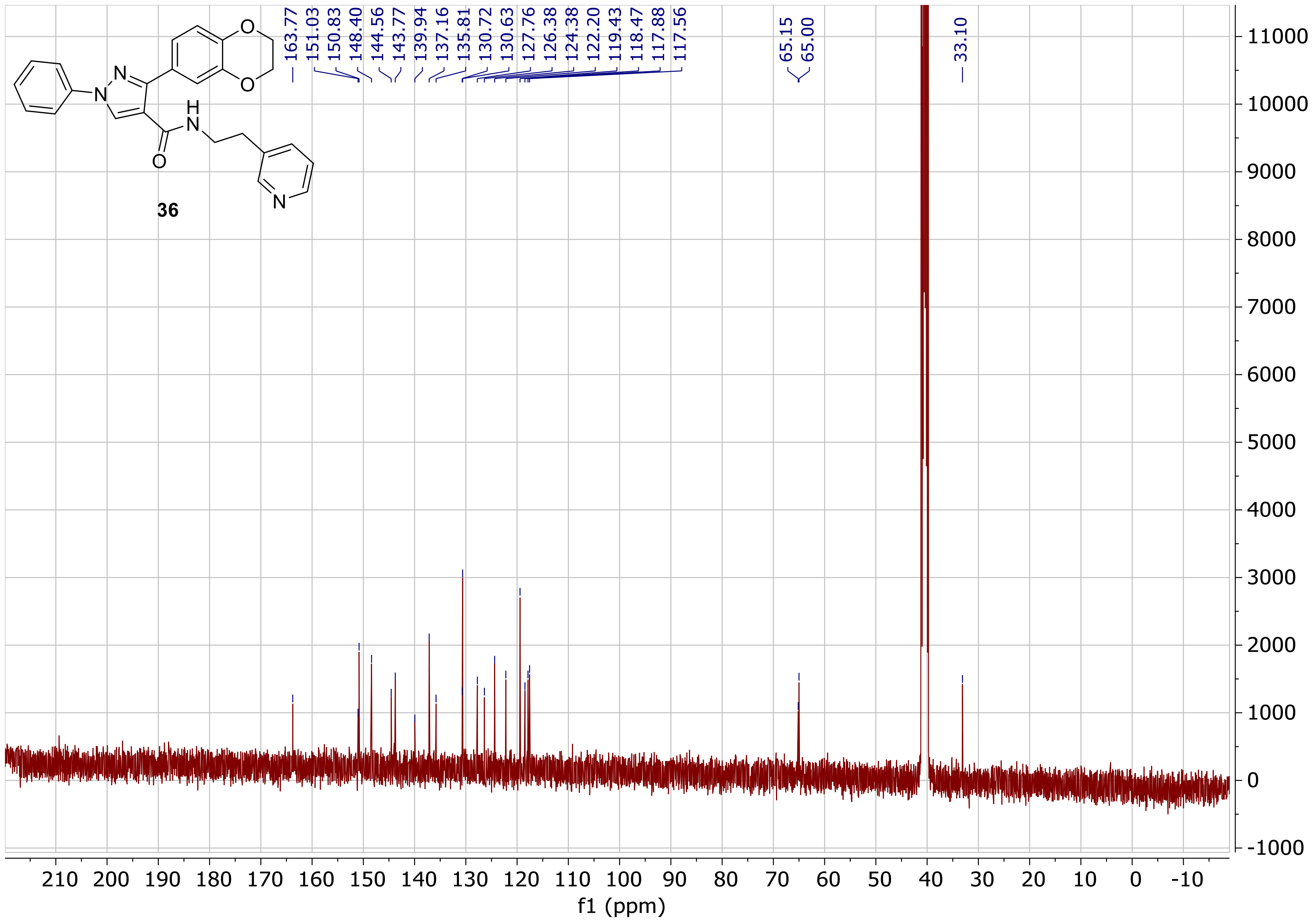
36
163.77
151.03
150.83
148.40
144.56
143.77
139.94
137.16
135.81
130.72
130.63
127.76
126.38
124.38
122.20
119.43
118.47
117.88
117.56
65.15
65.00
33.10
11000
10000
9000
8000
7000
6000
5000
4000
3000
2000
1000
0
-1000
210 200 190 180 170 160 150 140 130 120 110 100 90 80 70 60 50 40 30 20 10 0 -10
f1 (ppm)

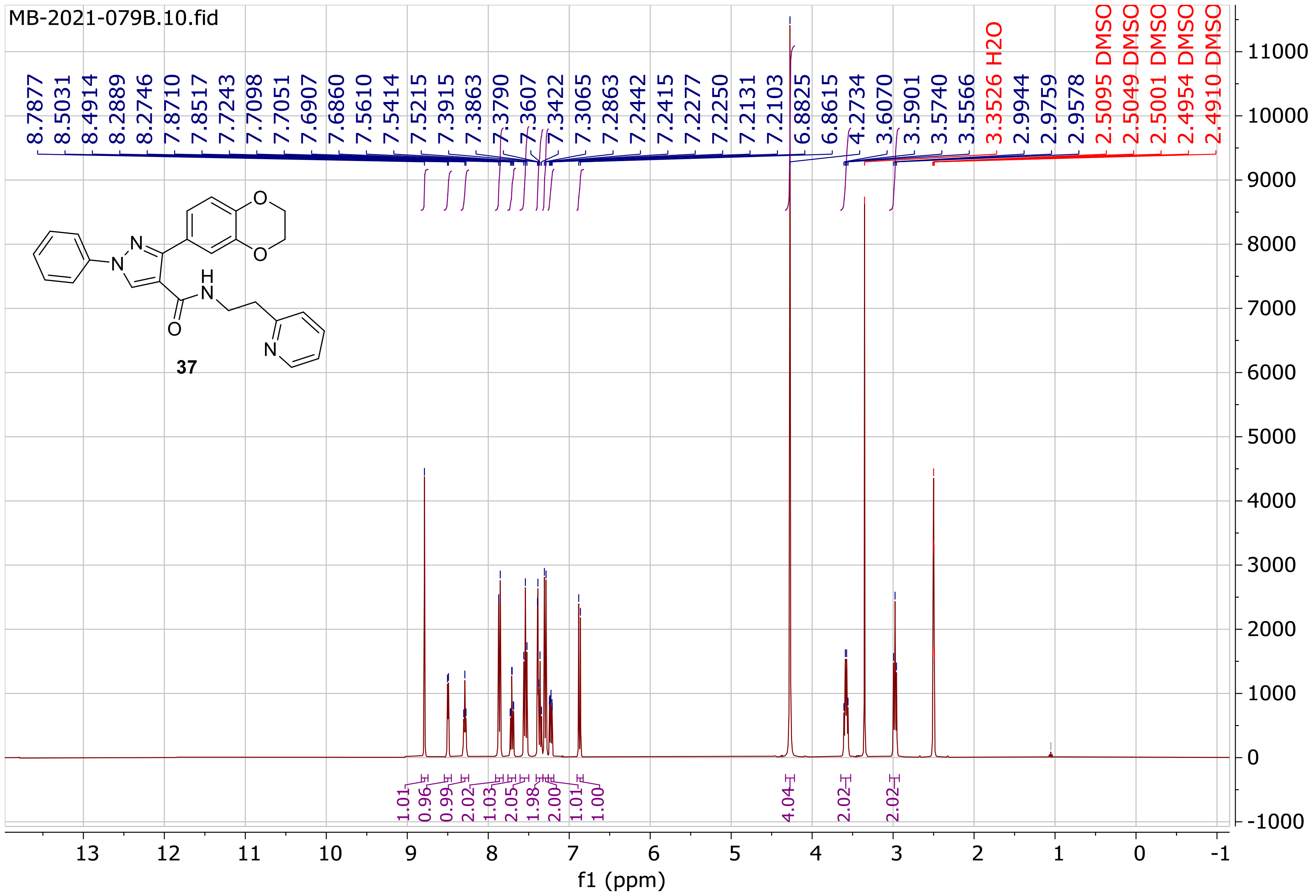

MB-2021-079B.10.fid
8.7877
8.5031
8.4914
8.2889
8.2746
7.8710
7.8517
7.7243
7.7098
7.7051
7.6907
7.6860
7.5610
7.5414
7.5215
7.3915
7.3863
7.3790
7.3607
7.3422
7.3065
7.2863
7.2442
7.2415
7.2277
7.2250
7.2131
7.2103
6.8825
6.8615
4.2734
3.6070
3.5901
3.5740
3.5566
3.3526 H2O
2.9944
2.9759
2.9578
2.5095 DMSO
2.5049 DMSO
2.5001 DMSO
2.4954 DMSO
2.4910 DMSO
N
N
O
O
H
N
O
N
37
1.01
0.96
0.99
2.02
1.03
2.05
1.98
2.00
1.01
1.00
4.04
2.02
2.02
f1 (ppm)

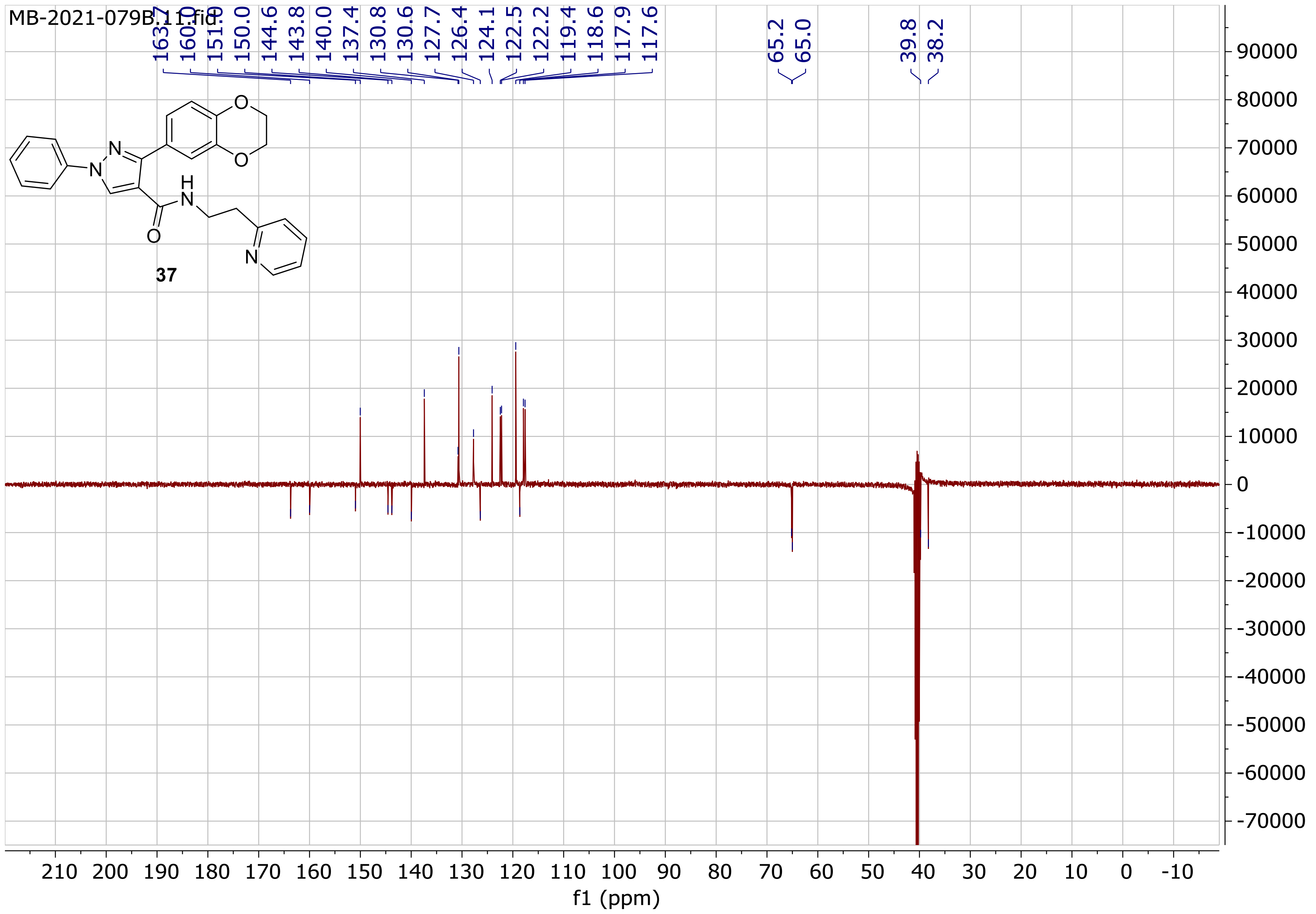
MB-2021-079B.11.fid
163.7
160.0
151.0
150.0
144.6
143.8
140.0
137.4
130.8
130.6
127.7
126.4
124.1
122.5
122.2
119.4
118.6
117.9
117.6
65.2
65.0
39.8
38.2
37
90000
80000
70000
60000
50000
40000
30000
20000
10000
0
-10000
-20000
-30000
-40000
-50000
-60000
-70000
210
200
190
180
170
160
150
140
130
120
110
100
90
80
70
60
50
40
30
20
10
0
-10
f1 (ppm)

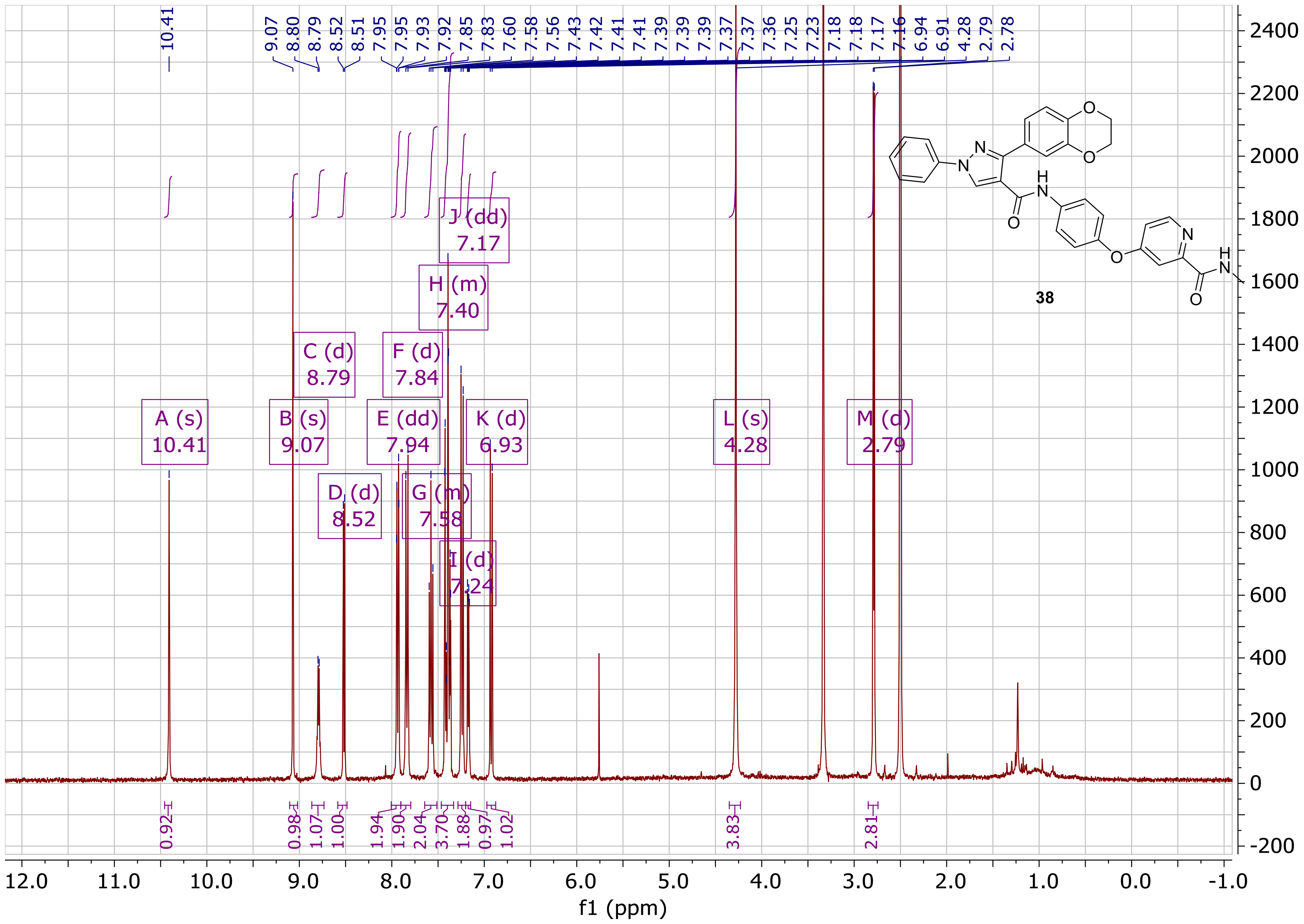

A (s) 10.41
B (s) 9.07
C (d) 8.79
D (d) 8.52
E (dd) 7.94
F (d) 7.84
G (m) 7.58
H (m) 7.40
I (d) 7.24
J (dd) 7.17
K (d) 6.93
L (s) 4.28
M (d) 2.79
38
f1 (ppm)

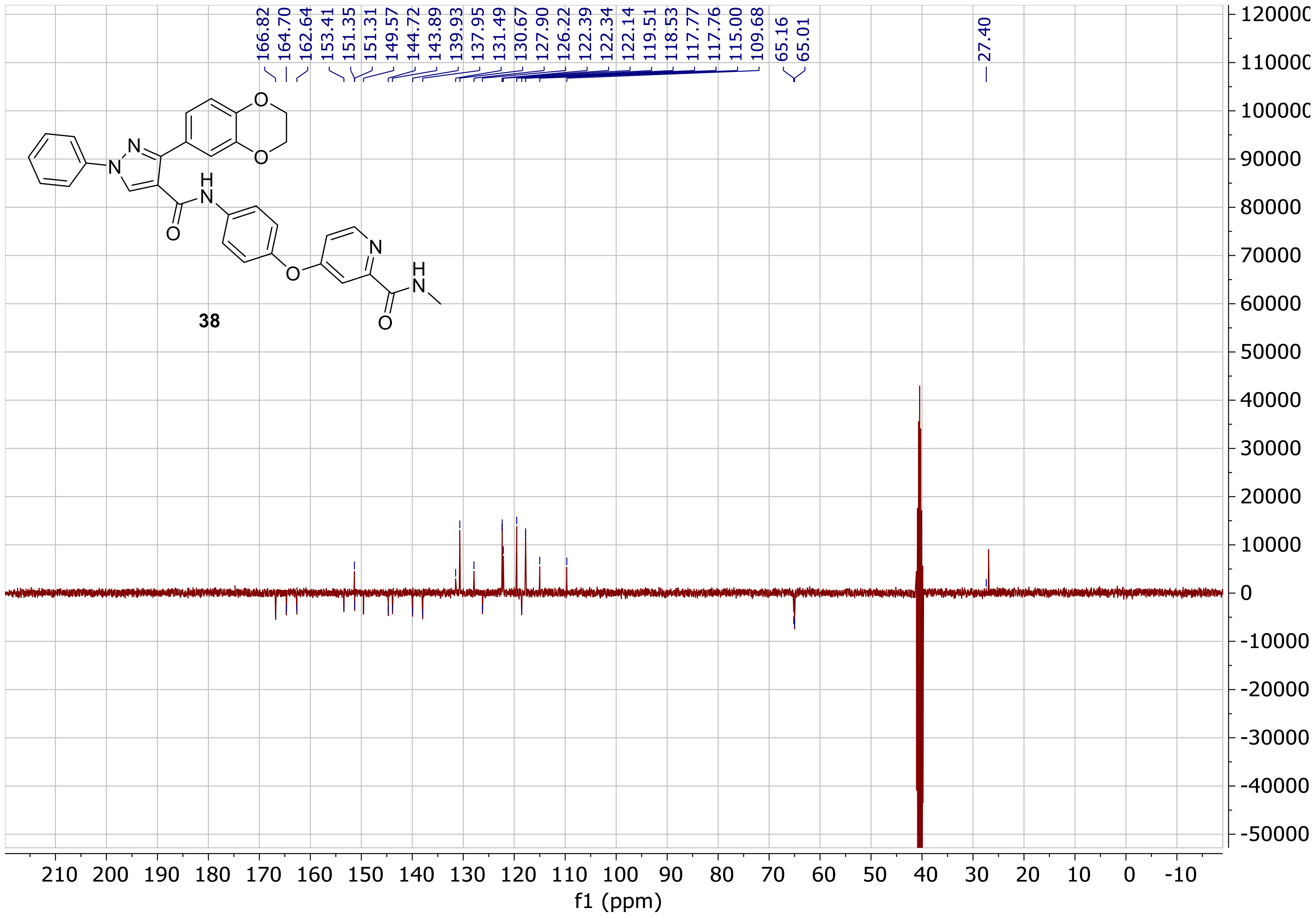

166.82
164.70
162.64
153.41
151.35
151.31
149.57
144.72
143.89
139.93
137.95
131.49
130.67
127.90
126.22
122.39
122.34
122.14
119.51
118.53
117.77
117.76
115.00
109.68
65.16
65.01
27.40
f1 (ppm)
N
N
O
O
H
N
O
N
O
H
N
O

38

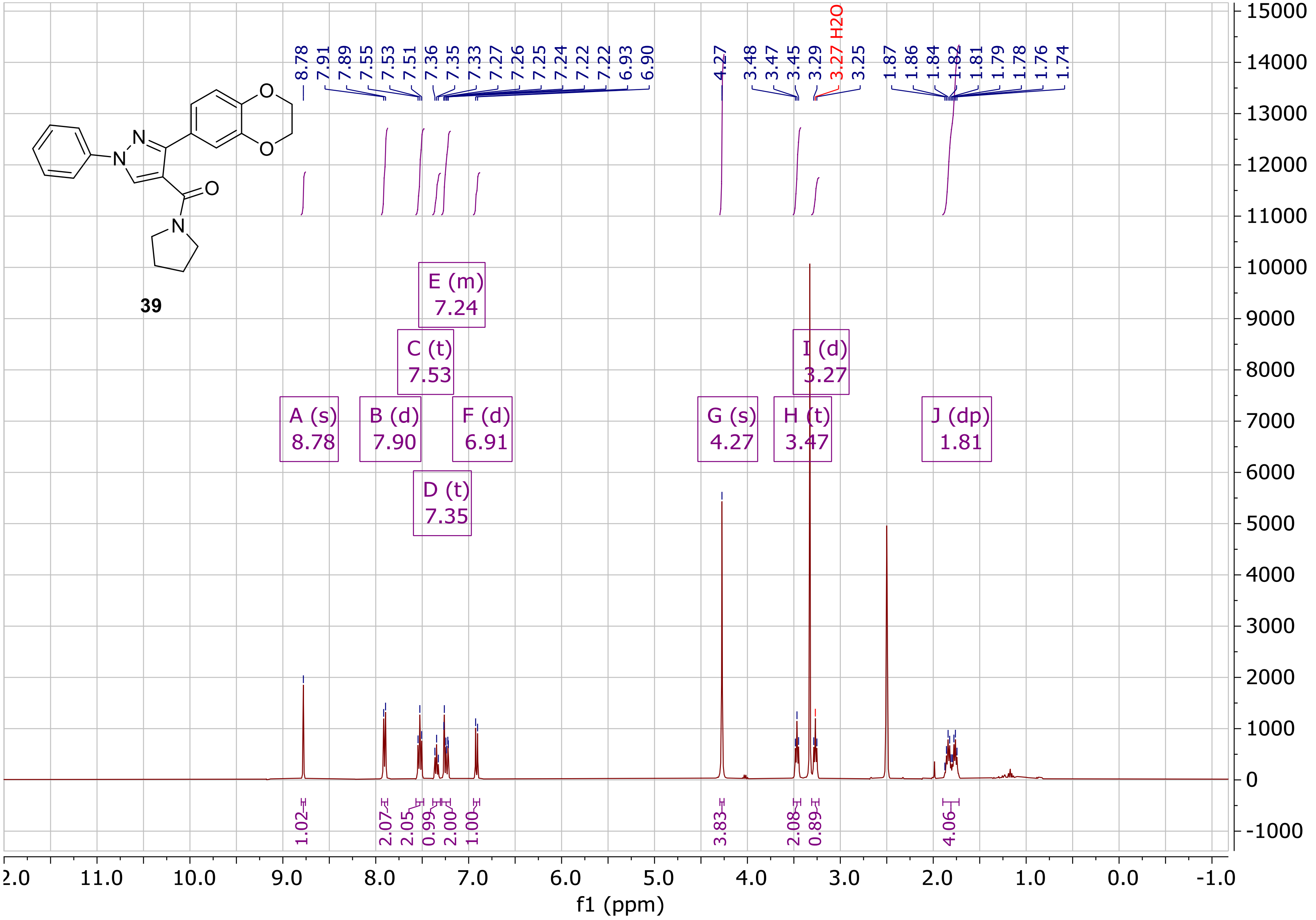
8.78
7.91
7.89
7.55
7.53
7.51
7.36
7.35
7.33
7.27
7.26
7.25
7.24
7.22
7.22
6.93
6.90
4.27
3.48
3.47
3.45
3.29
3.27 H2O
3.25
1.87
1.86
1.84
1.82
1.81
1.79
1.78
1.76
1.74
39
E (m) 7.24
C (t) 7.53
I (d) 3.27
A (s) 8.78
B (d) 7.90
F (d) 6.91
G (s) 4.27
H (t) 3.47
J (dp) 1.81
D (t) 7.35
1.02
2.07
2.05
0.99
2.00
1.00
3.83
2.08
0.89
4.06
f1 (ppm)

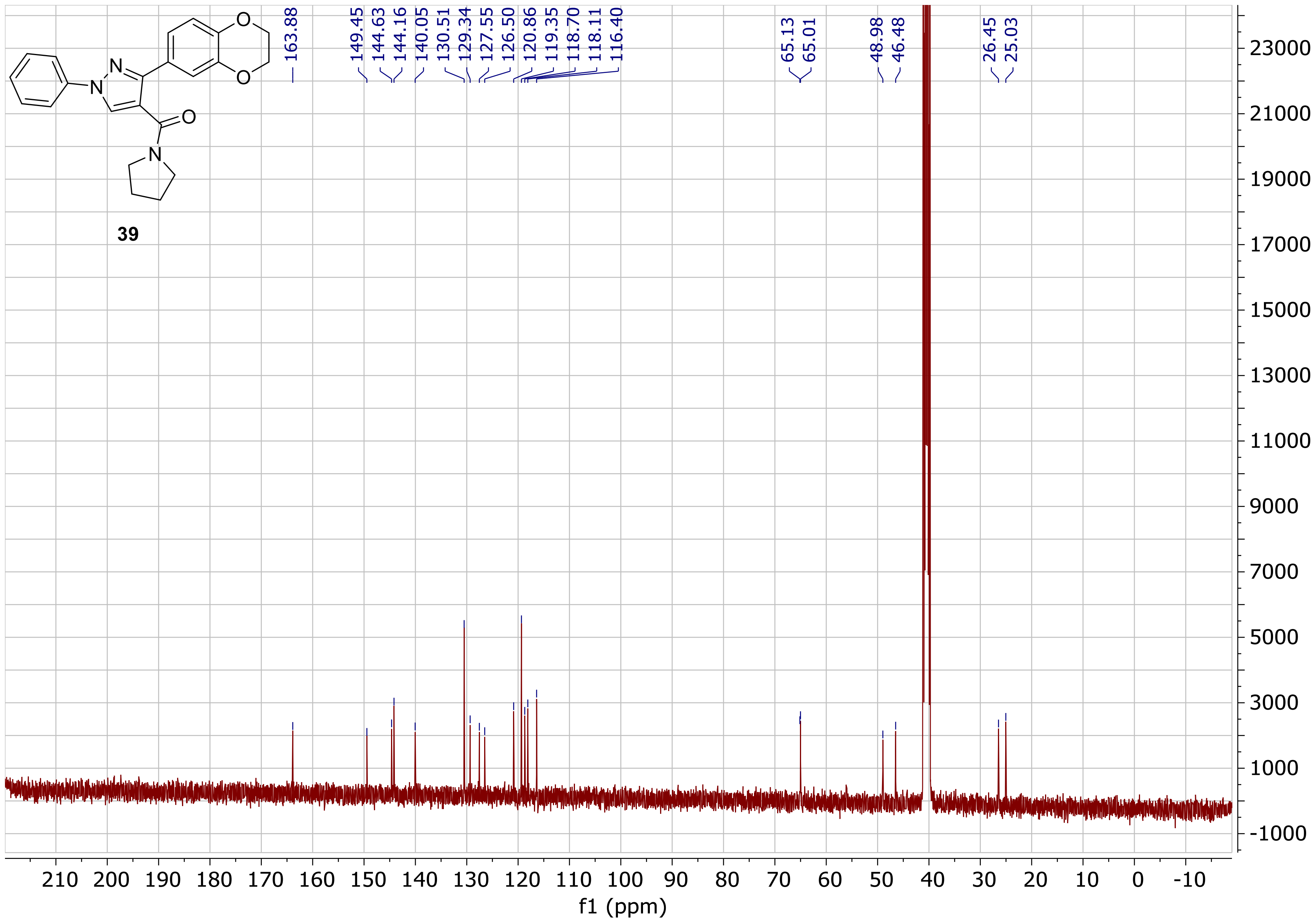

39
163.88
149.45
144.63
144.16
140.05
130.51
129.34
127.55
126.50
120.86
119.35
118.70
118.11
116.40
65.13
65.01
48.98
46.48
26.45
25.03
f1 (ppm)

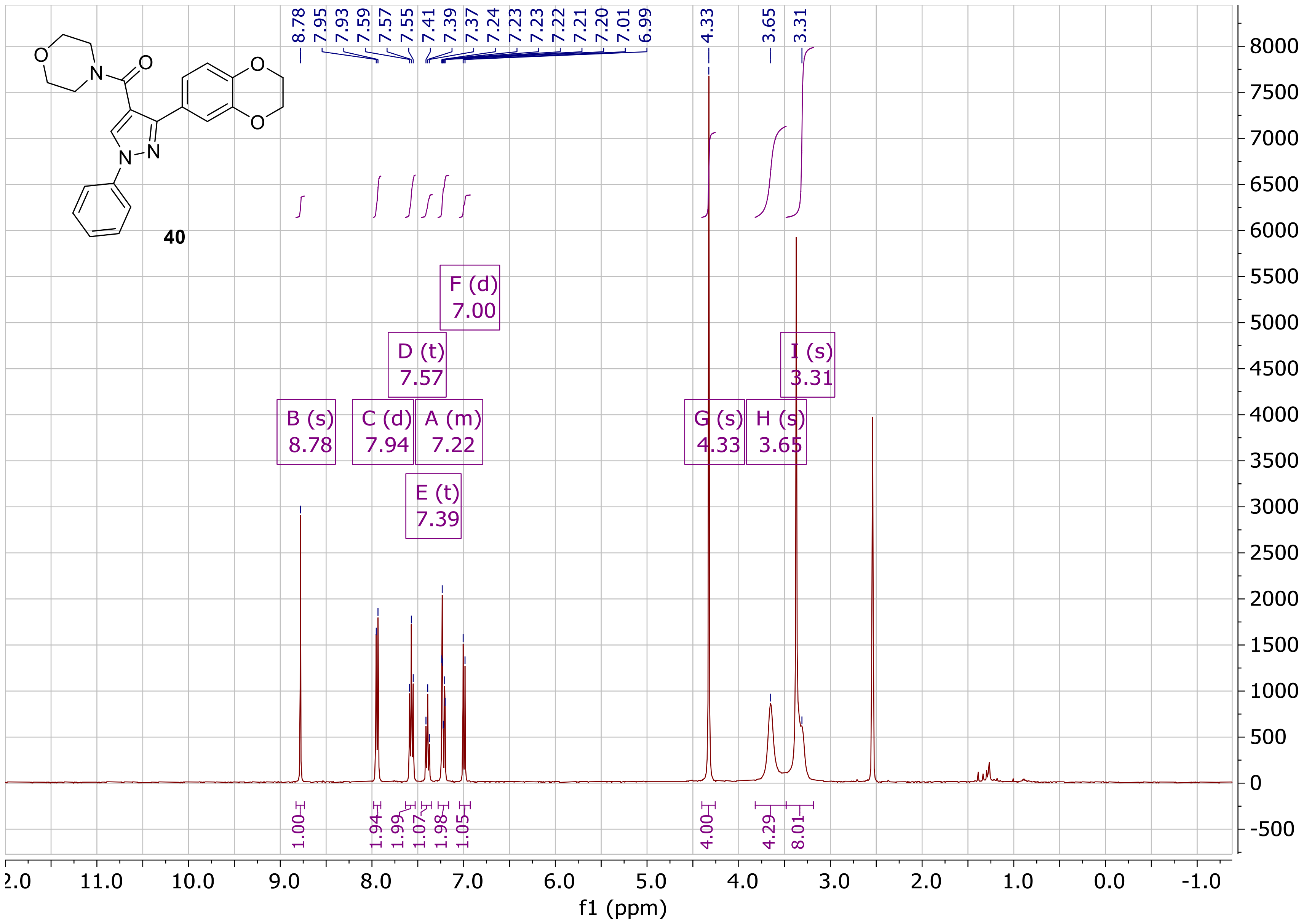

40
8.78
7.95
7.93
7.59
7.57
7.55
7.41
7.39
7.37
7.24
7.23
7.23
7.22
7.21
7.20
7.01
6.99
4.33
3.65
3.31
F (d) 7.00
D (t) 7.57
I (s) 3.31
B (s) 8.78
C (d) 7.94
A (m) 7.22
G (s) 4.33
H (s) 3.65
E (t) 7.39
1.00
1.94
1.99
1.07
1.98
1.05
4.00
4.29
8.01
f1 (ppm)

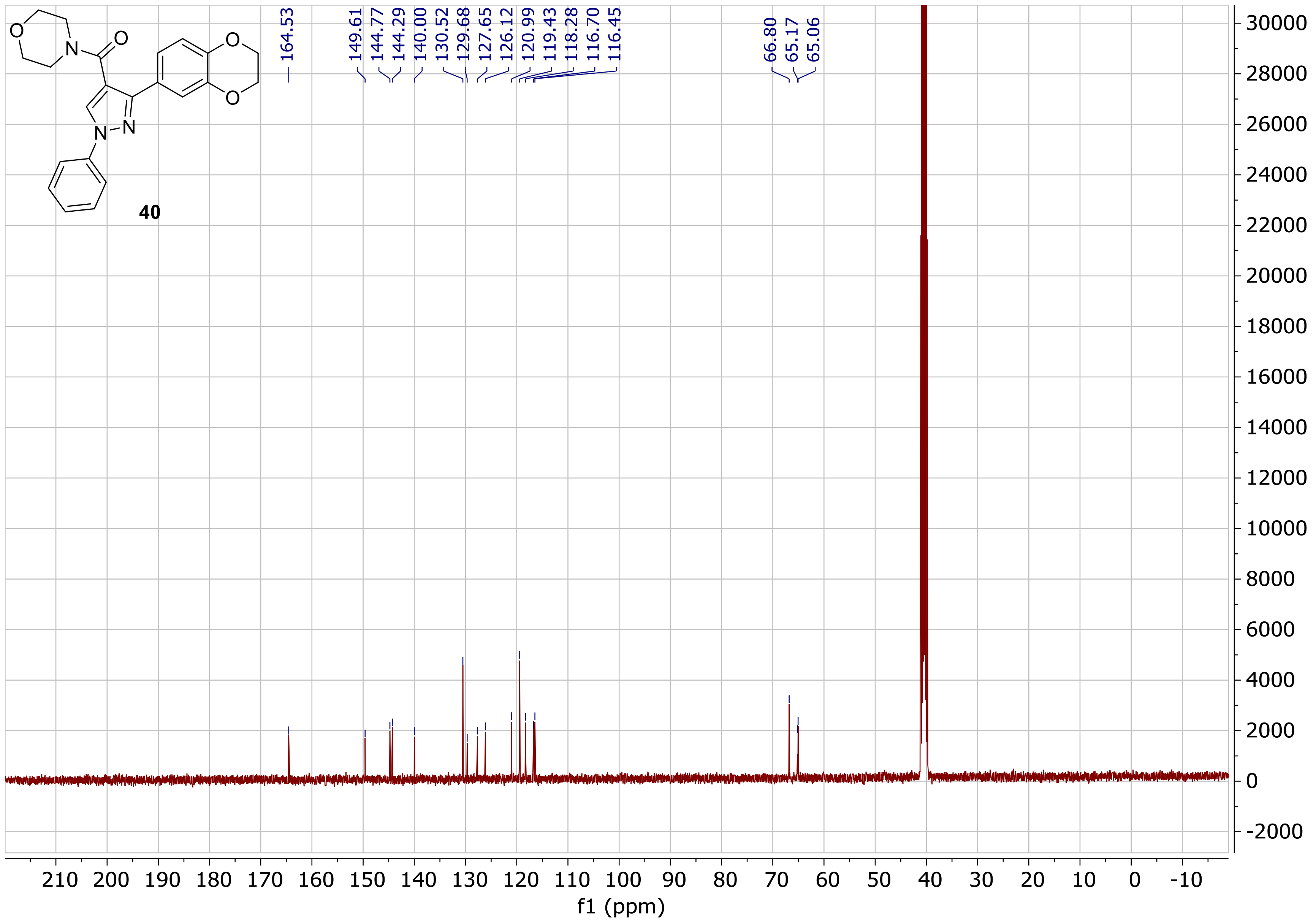
40
164.53
149.61
144.77
144.29
140.00
130.52
129.68
127.65
126.12
120.99
119.43
118.28
116.70
116.45
66.80
65.17
65.06
30000
28000
26000
24000
22000
20000
18000
16000
14000
12000
10000
8000
6000
4000
2000
0
-2000
210 200 190 180 170 160 150 140 130 120 110 100 90 80 70 60 50 40 30 20 10 0 -10
f1 (ppm)

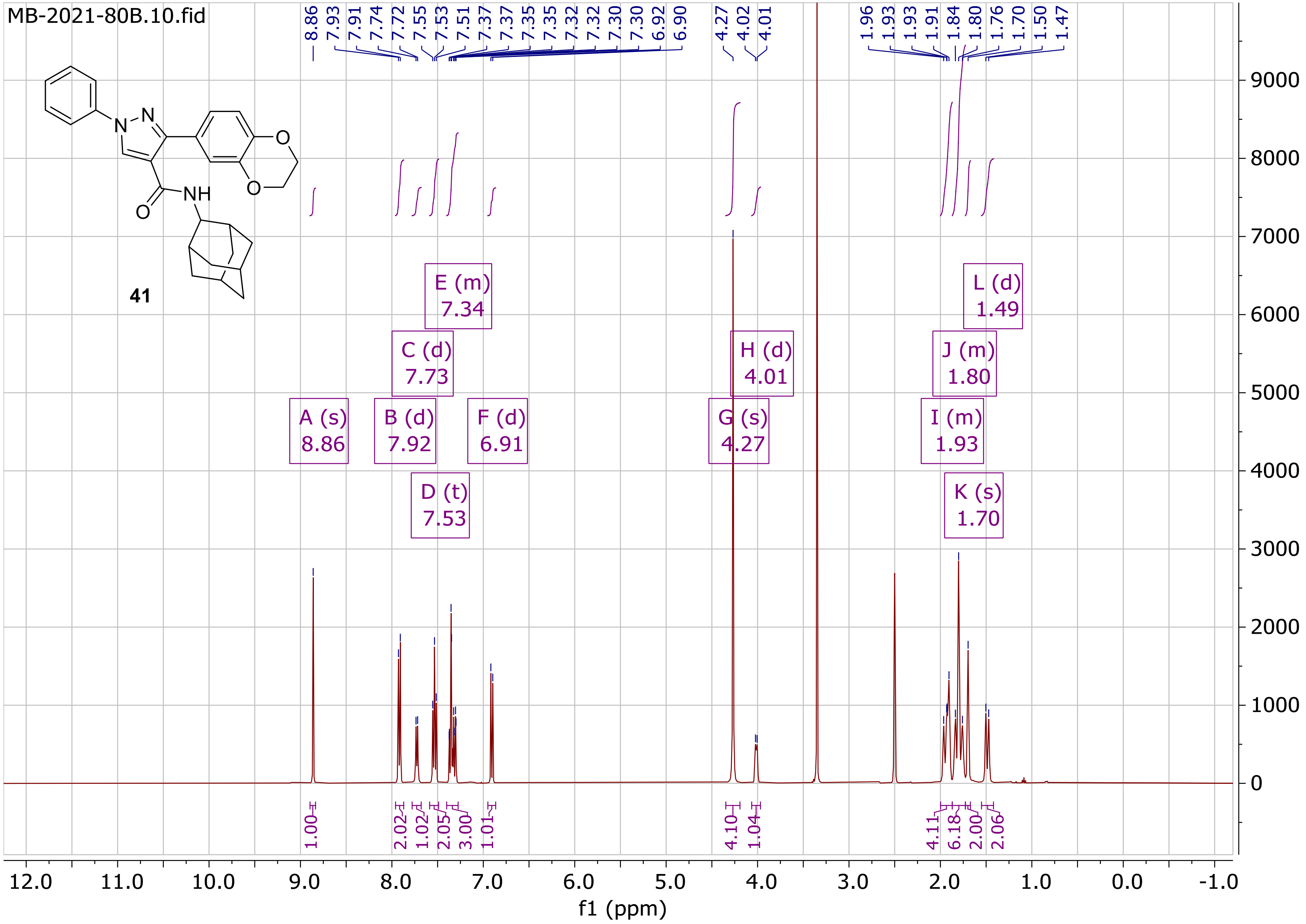
MB-2021-80B.10.fid
8.86 7.93 7.91 7.74 7.72 7.55 7.53 7.51 7.37 7.37 7.35 7.35 7.32 7.32 7.30 7.30 6.92 6.90
4.27 4.02 4.01
1.96 1.93 1.93 1.91 1.84 1.80 1.76 1.70 1.50 1.47
41
A (s) 8.86
B (d) 7.92
C (d) 7.73
D (t) 7.53
E (m) 7.34
F (d) 6.91
G (s) 4.27
H (d) 4.01
I (m) 1.93
J (m) 1.80
K (s) 1.70
L (d) 1.49
1.00 2.02 1.02 2.05 3.00 1.01 4.10 1.04 4.11 6.18 2.00 2.06
f1 (ppm)

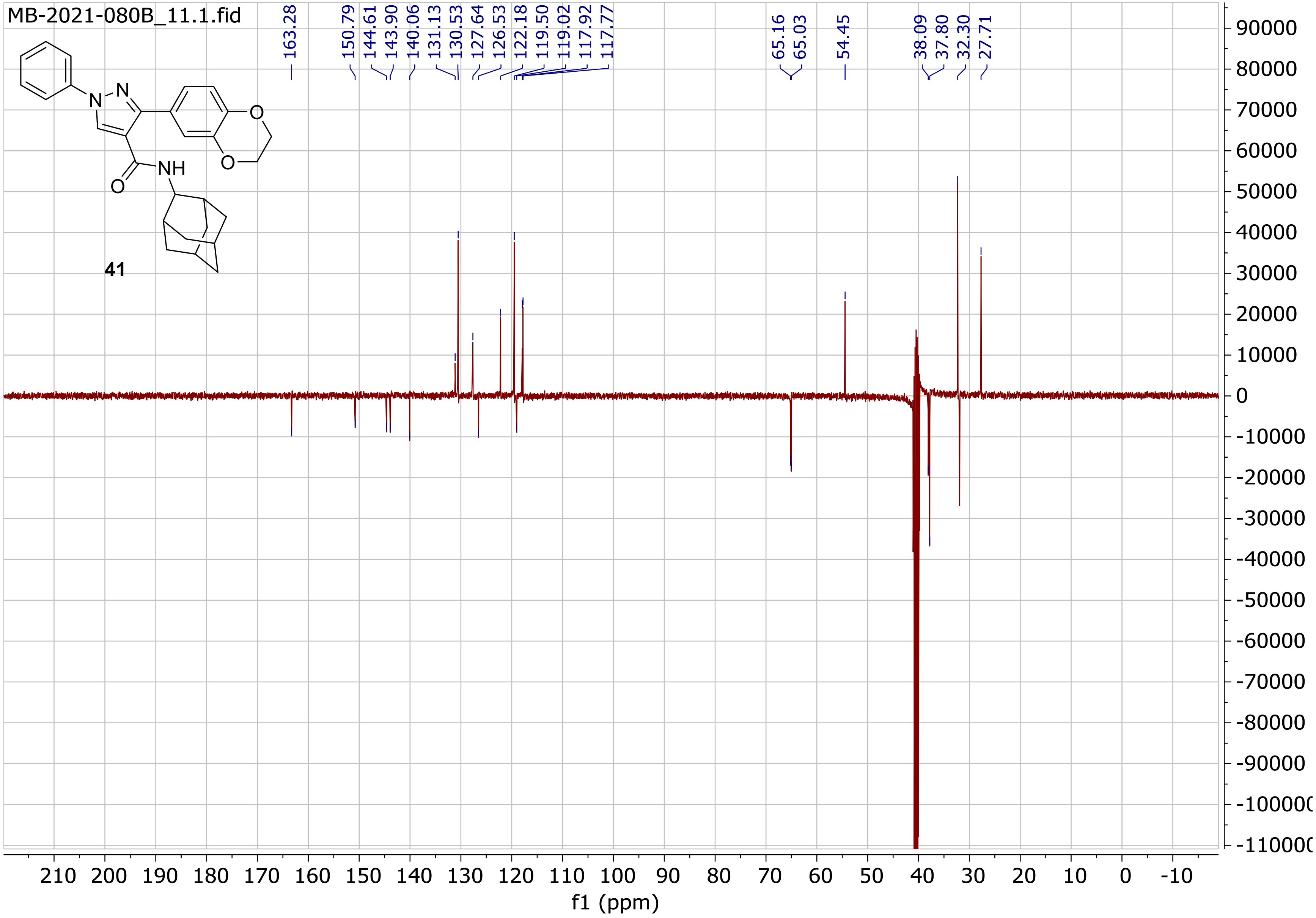
MB-2021-080B_11.1.fid
41
163.28
150.79
144.61
143.90
140.06
131.13
130.53
127.64
126.53
122.18
119.50
119.02
117.92
117.77
65.16
65.03
54.45
38.09
37.80
32.30
27.71
f1 (ppm)

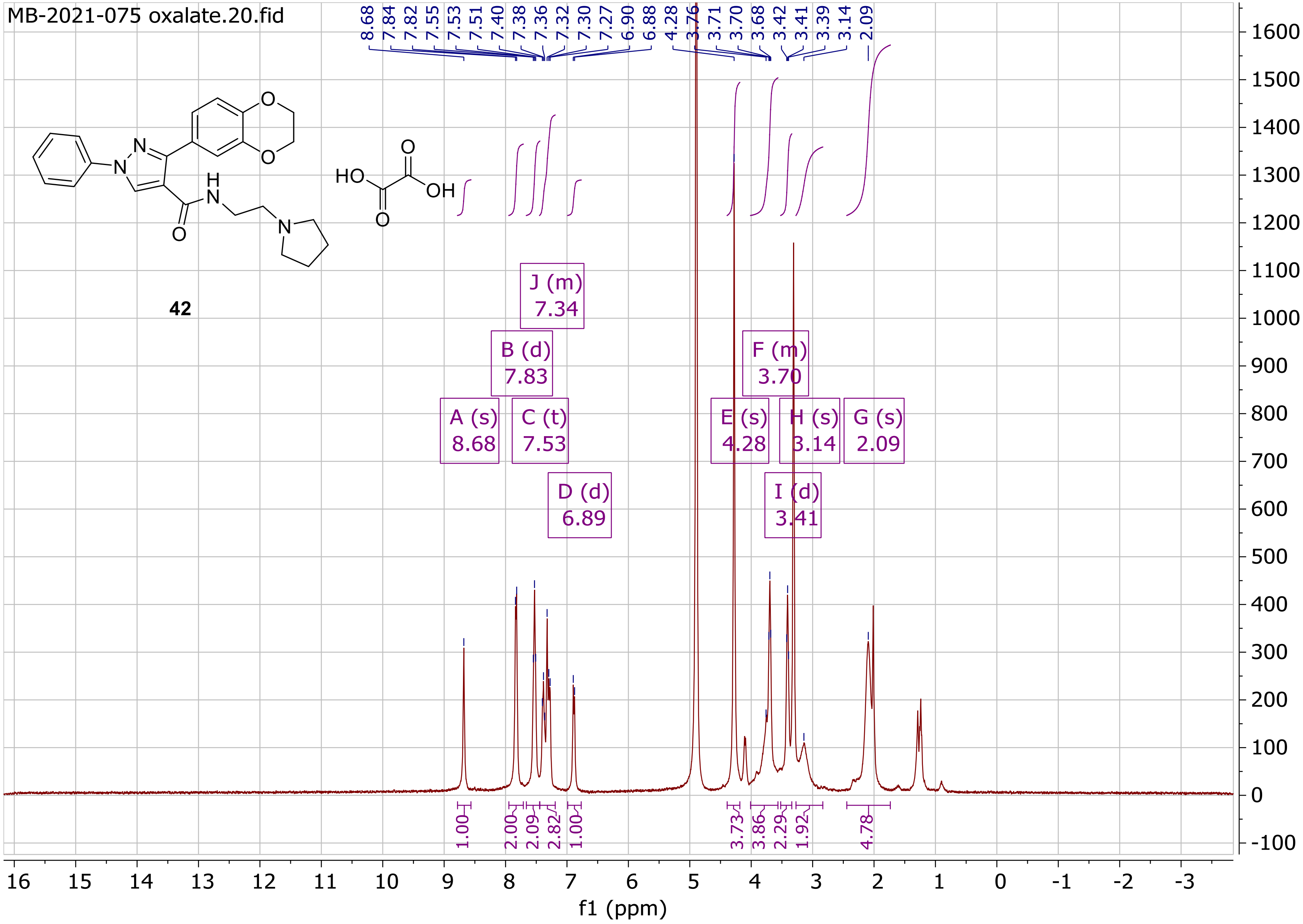
MB-2021-075 oxalate.20.fid
8.68 7.84 7.82 7.55 7.53 7.51 7.40 7.38 7.36 7.32 7.30 7.27 6.90 6.88 4.28 3.76 3.71 3.70 3.68 3.42 3.41 3.39 3.14 2.09
42
HO
OH
O
N
H
J (m) 7.34
B (d) 7.83
A (s) 8.68
C (t) 7.53
D (d) 6.89
F (m) 3.70
E (s) 4.28
H (s) 3.14
G (s) 2.09
I (d) 3.41
1.00 2.00 2.09 2.82 1.00 3.73 3.86 2.29 1.92 4.78
f1 (ppm)

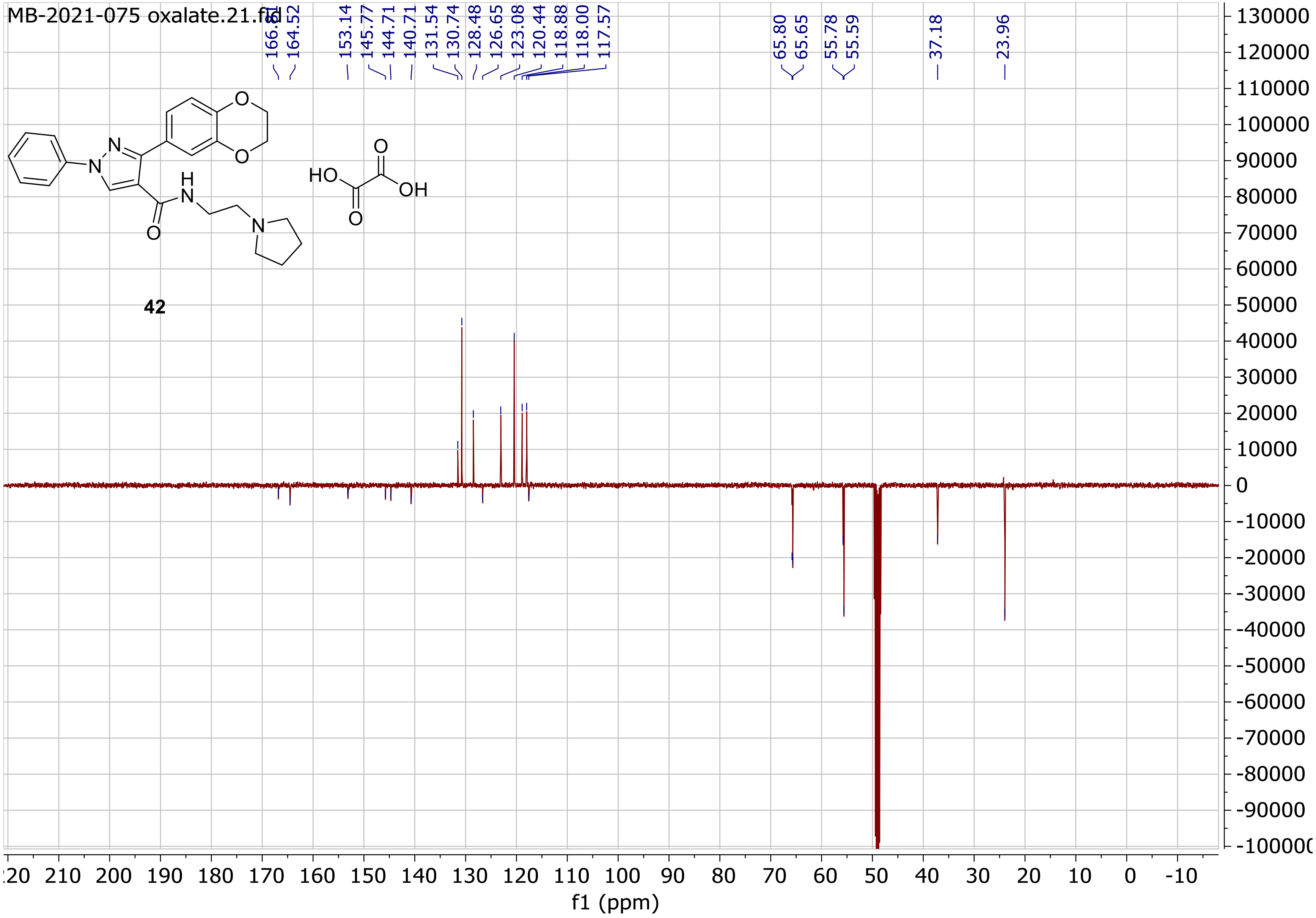
MB-2021-075 oxalate.21.fid
164.52
153.14
145.77
144.71
140.71
131.54
130.74
128.48
126.65
123.08
120.44
118.88
118.00
117.57
65.80
65.65
55.78
55.59
37.18
23.96
42
f1 (ppm)

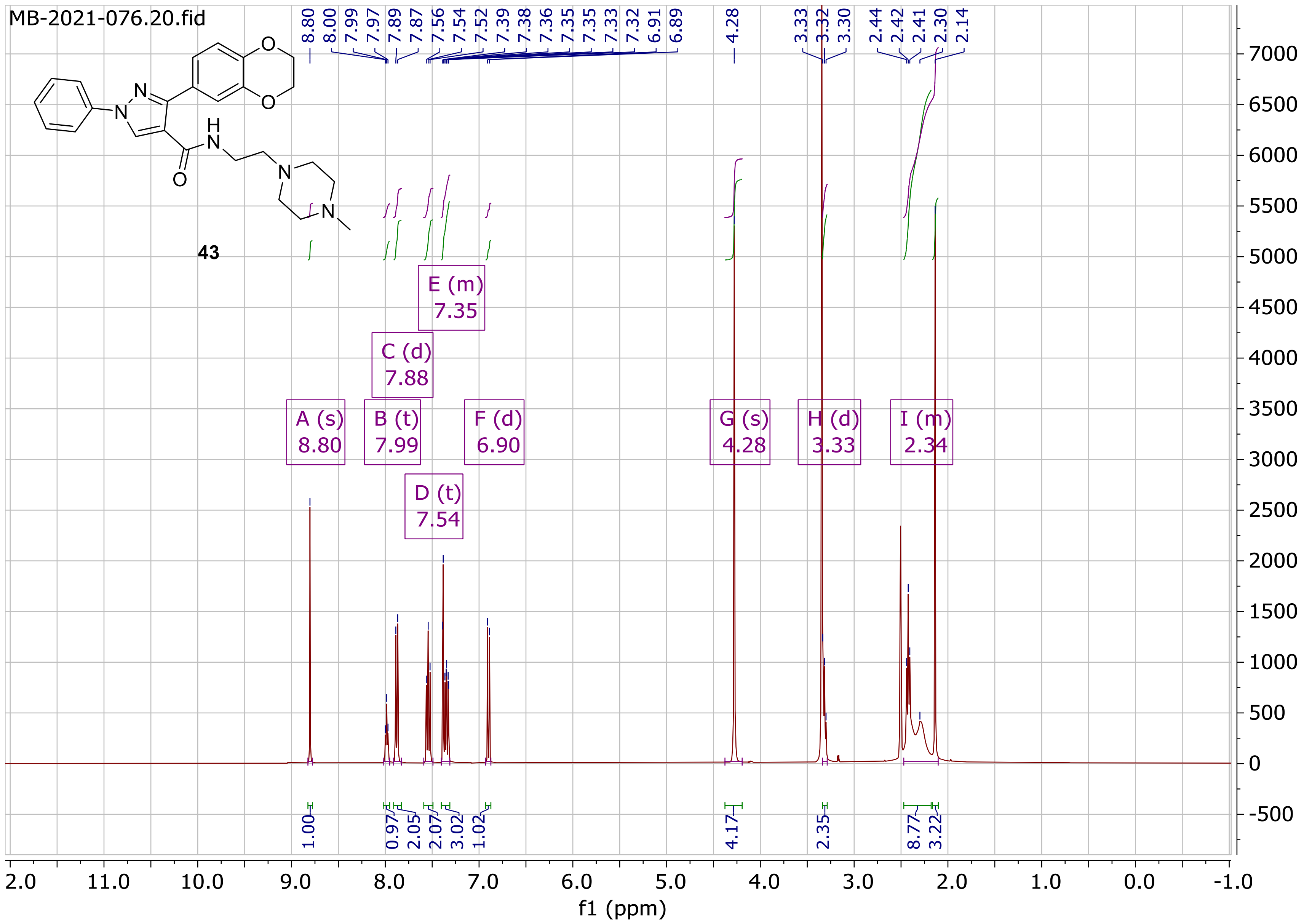

MB-2021-076.20.fid
43
8.80
8.00
7.99
7.97
7.89
7.87
7.56
7.54
7.52
7.39
7.38
7.36
7.35
7.35
7.33
7.32
6.91
6.89
4.28
3.33
3.32
3.30
2.44
2.42
2.41
2.30
2.14
A (s) 8.80
B (t) 7.99
C (d) 7.88
D (t) 7.54
E (m) 7.35
F (d) 6.90
G (s) 4.28
H (d) 3.33
I (m) 2.34
1.00
0.97
2.05
2.07
3.02
1.02
4.17
2.35
8.77
3.22
f1 (ppm)

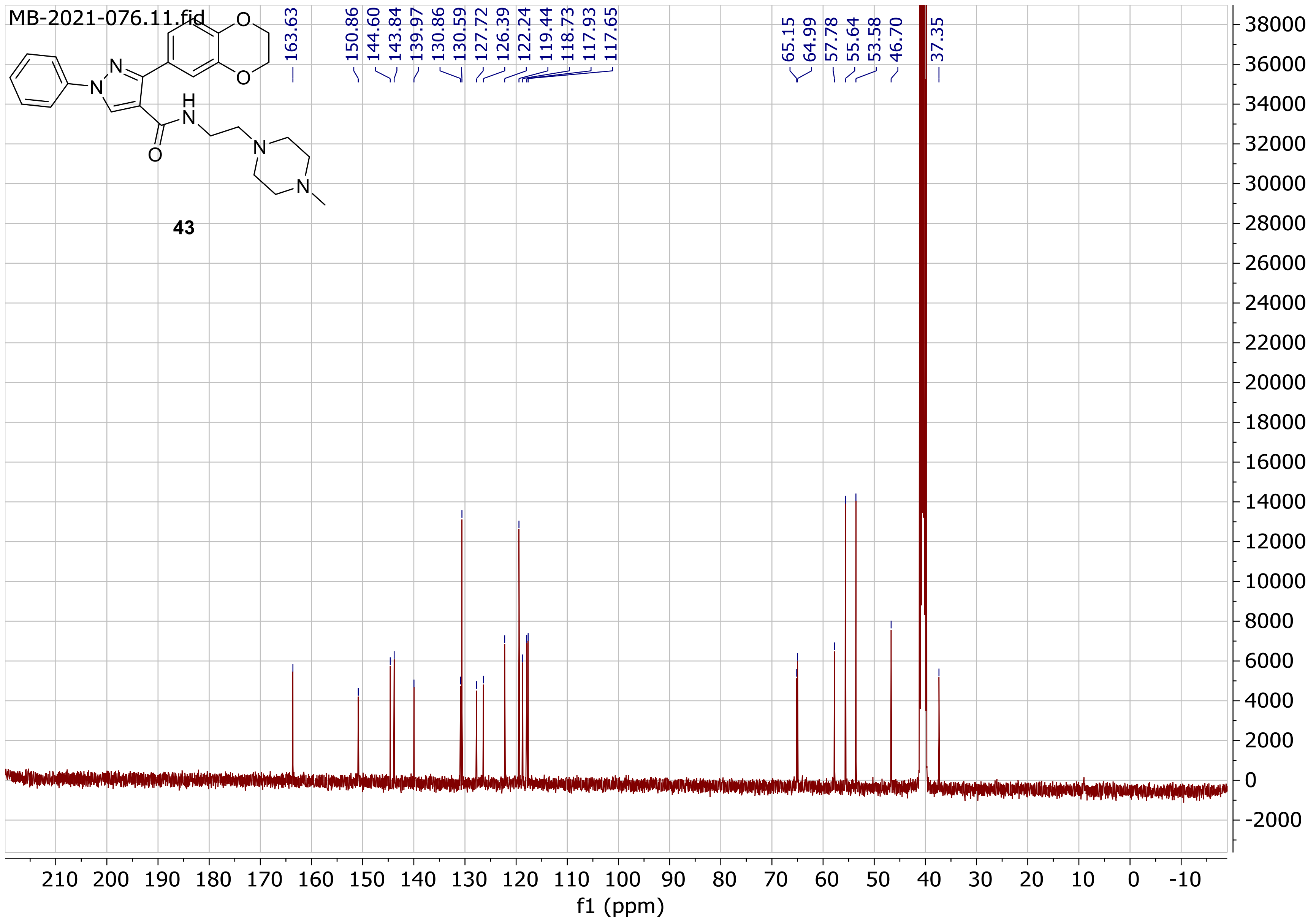
MB-2021-076.11.fid
43
163.63
150.86
144.60
143.84
139.97
130.86
130.59
127.72
126.39
122.24
119.44
118.73
117.93
117.65
65.15
64.99
57.78
55.64
53.58
46.70
37.35
f1 (ppm)

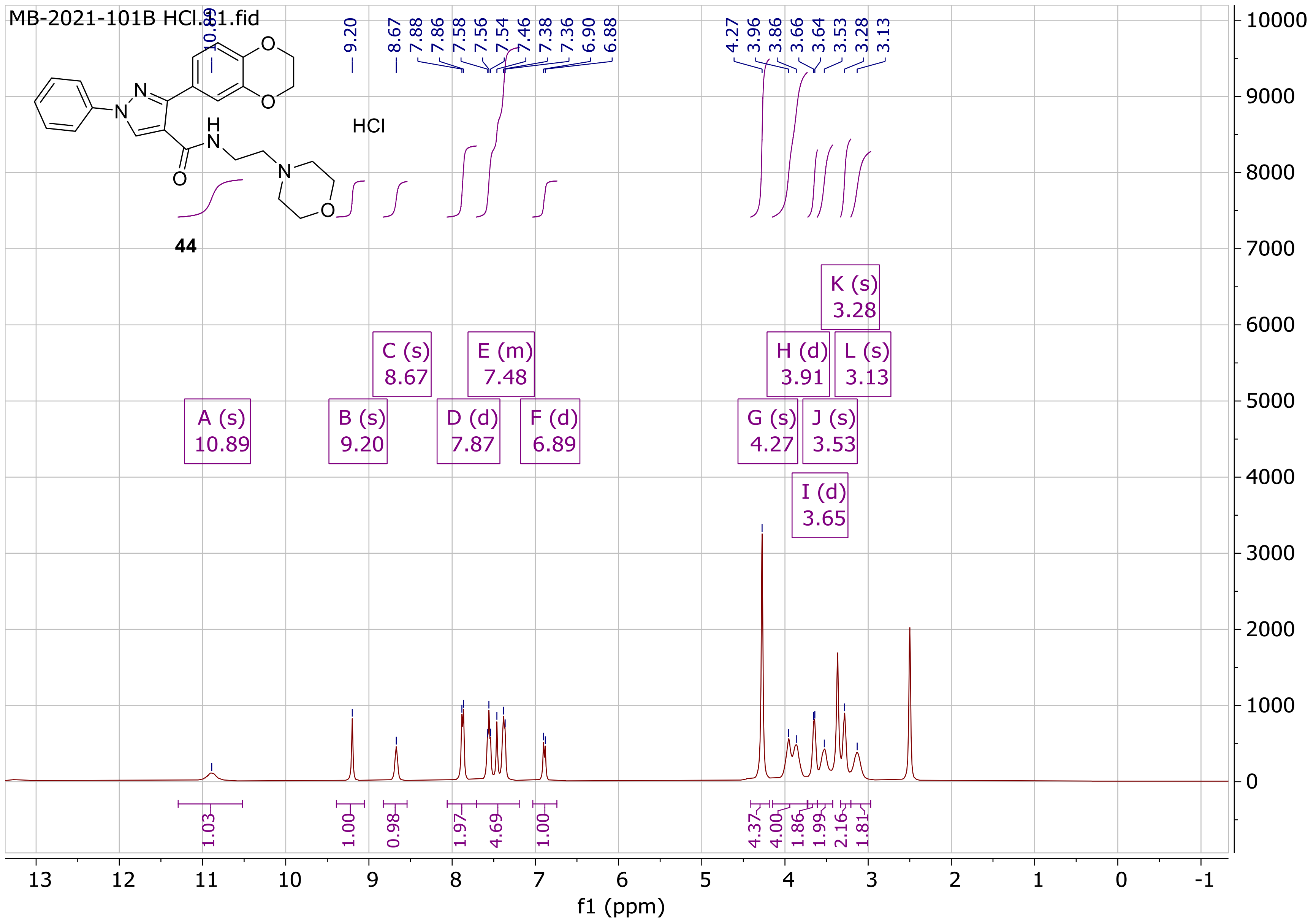
MB-2021-101B HCl.11.fid
HCl
44
10.89
9.20
8.67
7.88
7.86
7.58
7.56
7.54
7.46
7.38
7.36
6.90
6.88
4.27
3.96
3.86
3.66
3.64
3.53
3.28
3.13
A (s) 10.89
B (s) 9.20
C (s) 8.67
D (d) 7.87
E (m) 7.48
F (d) 6.89
G (s) 4.27
H (d) 3.91
I (d) 3.65
J (s) 3.53
K (s) 3.28
L (s) 3.13
1.03
1.00
0.98
1.97
4.69
1.00
4.37
4.00
1.86
1.99
2.16
1.81
f1 (ppm)

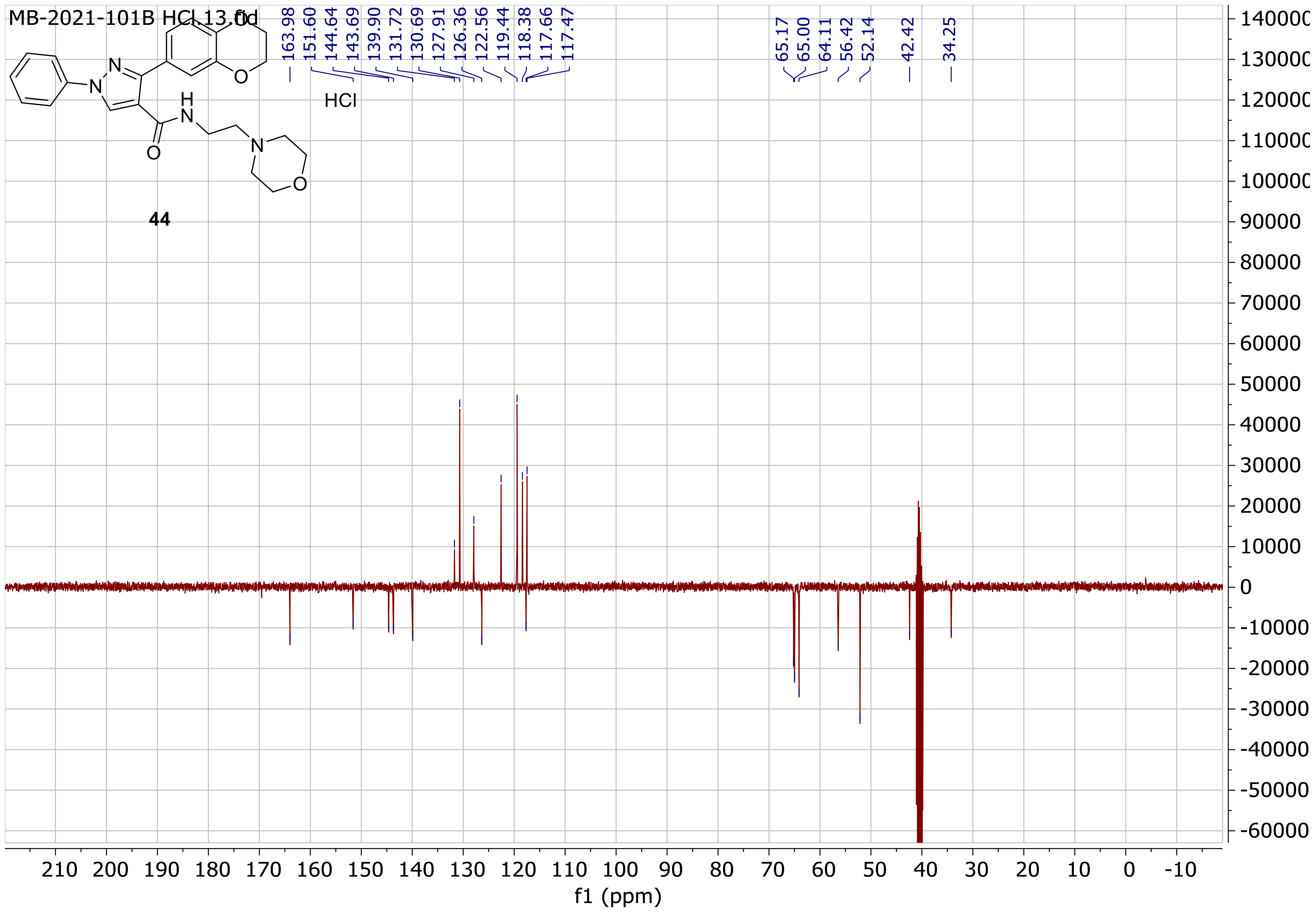

MB-2021-101B HCl 13.fid
HCl
44
163.98
151.60
144.64
143.69
139.90
131.72
130.69
127.91
126.36
122.56
119.44
118.38
117.66
117.47
65.17
65.00
64.11
56.42
52.14
42.42
34.25
f1 (ppm)

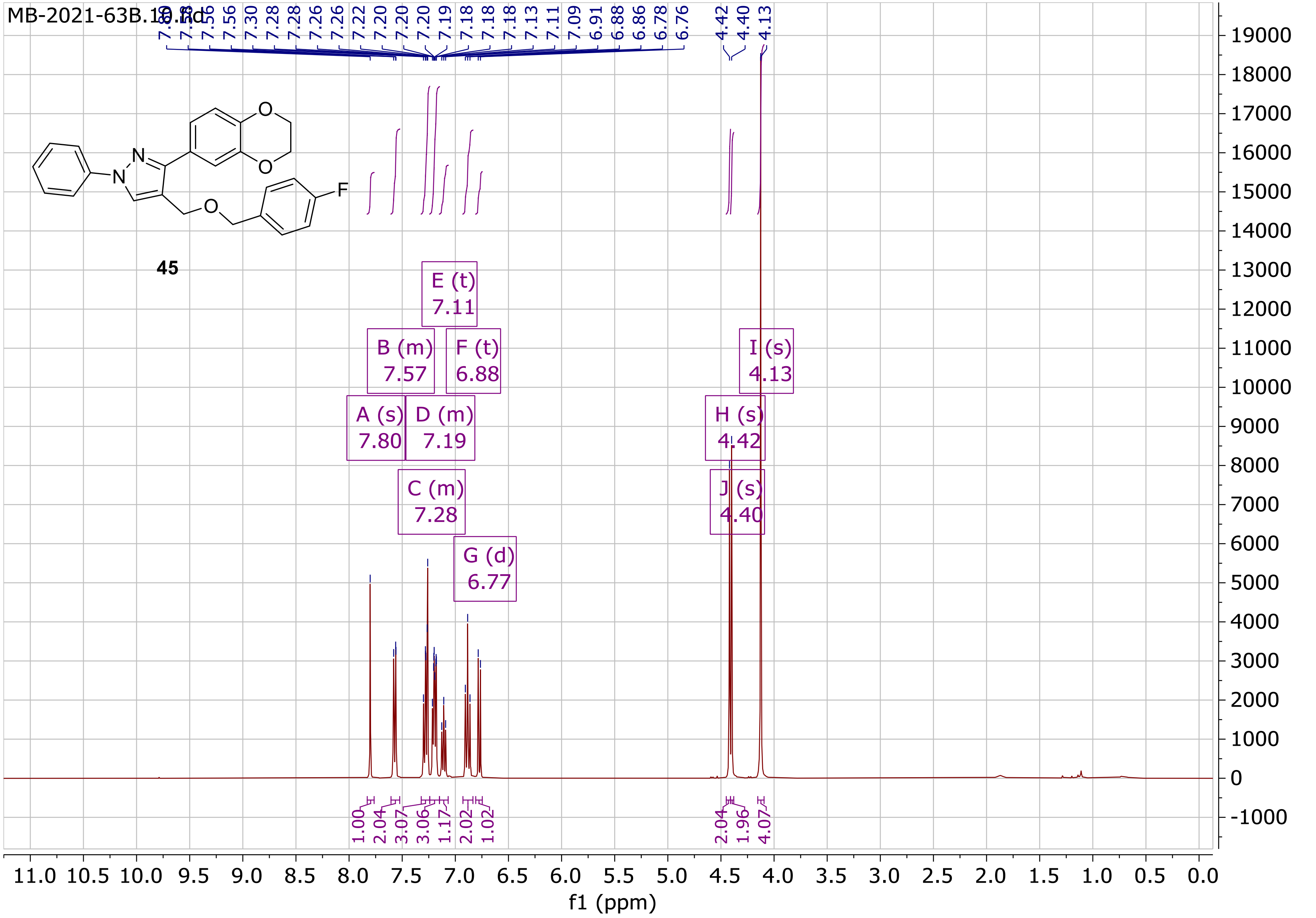

7.56
7.30
7.28
7.28
7.26
7.26
7.22
7.20
7.20
7.20
7.19
7.18
7.18
7.18
7.13
7.11
7.09
6.91
6.88
6.86
6.78
6.76
4.42
4.40
4.13
O
O
N
N
F
O
45
A (s) 7.80
B (m) 7.57
C (m) 7.28
D (m) 7.19
E (t) 7.11
F (t) 6.88
G (d) 6.77
H (s) 4.42
I (s) 4.13
J (s) 4.40
1.00
2.04
3.07
3.06
1.17
2.02
1.02
2.04
1.96
4.07
f1 (ppm)

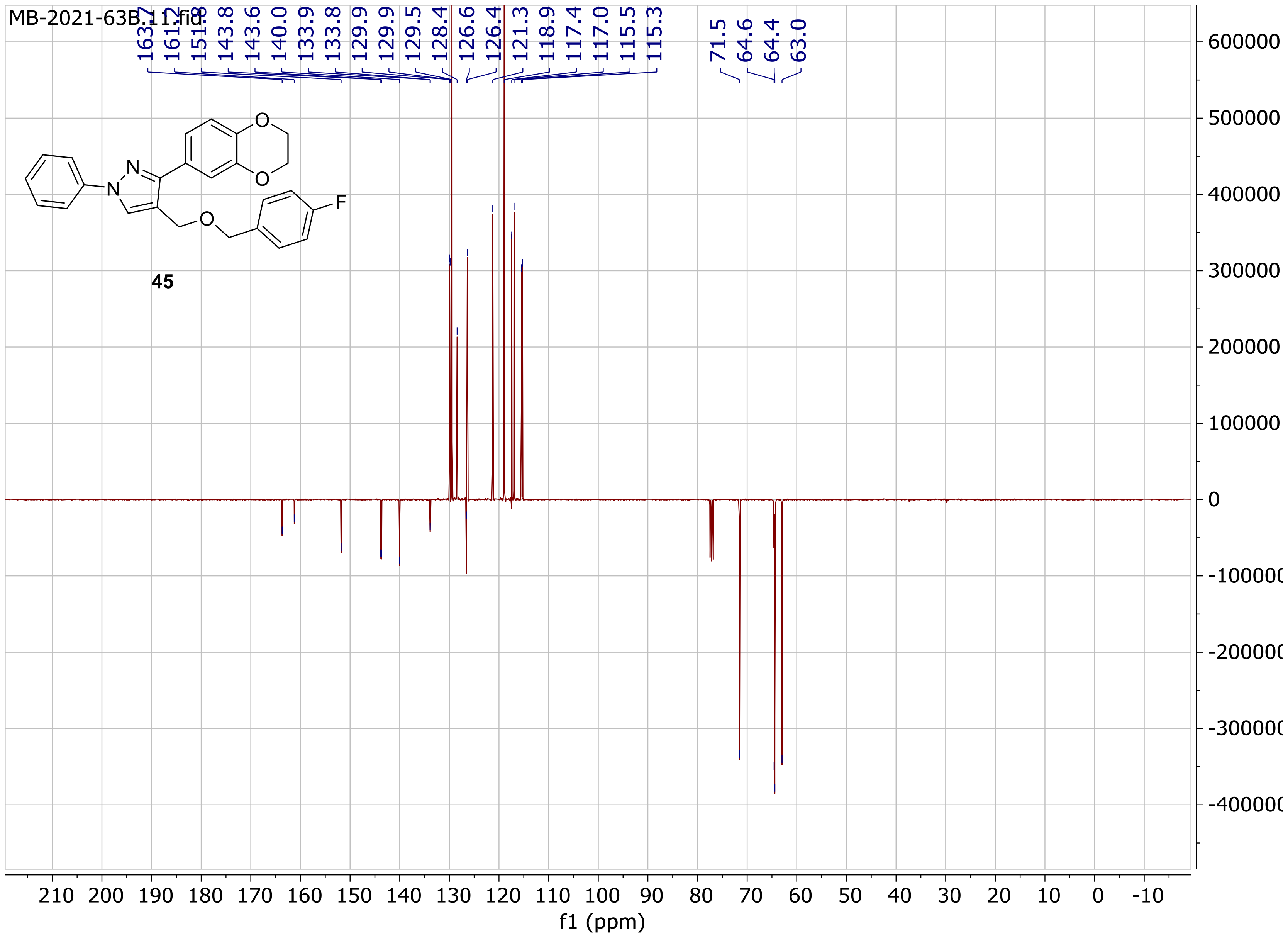
MB-2021-63B.11.fid
163.7
161.2
151.8
143.8
143.6
140.0
133.9
133.8
129.9
129.9
129.5
128.4
126.6
126.4
121.3
118.9
117.4
117.0
115.5
115.3
71.5
64.6
64.4
63.0
N
N
O
O
O
F
45
600000
500000
400000
300000
200000
100000
0
210 200 190 180 170 160 150 140 130 120 110 100 90 80 70 60 50 40 30 20 10 0 -10
f1 (ppm)

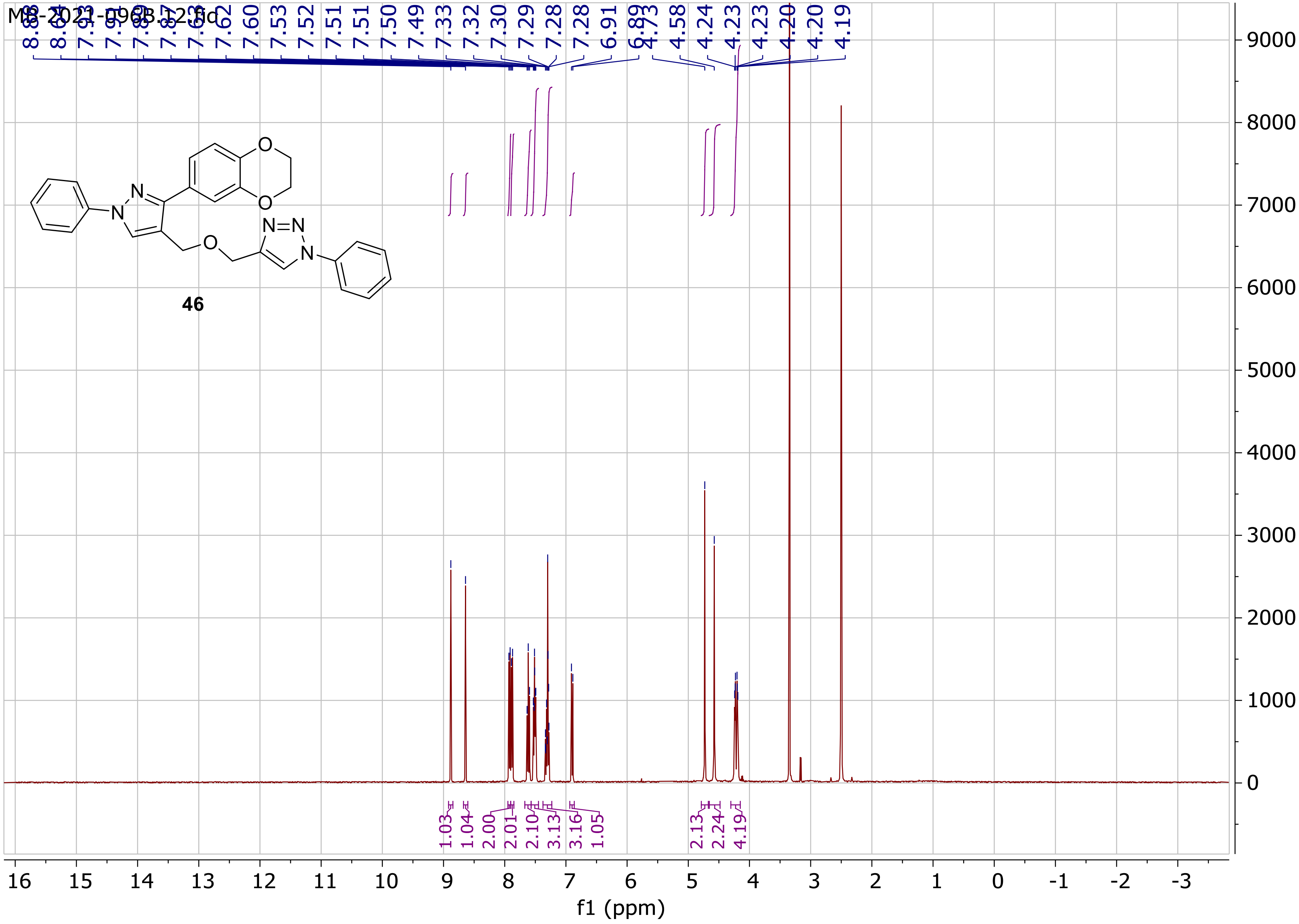
7.62
7.60
7.53
7.52
7.51
7.51
7.50
7.49
7.33
7.32
7.30
7.29
7.28
7.28
6.91
4.58
4.24
4.23
4.23
4.20
4.20
4.19
46
1.03
1.04
2.00
2.01
2.10
3.13
3.16
1.05
2.13
2.24
4.19
16 15 14 13 12 11 10 9 8 7 6 5 4 3 2 1 0 -1 -2 -3
f1 (ppm)
9000 8000 7000 6000 5000 4000 3000 2000 1000 0

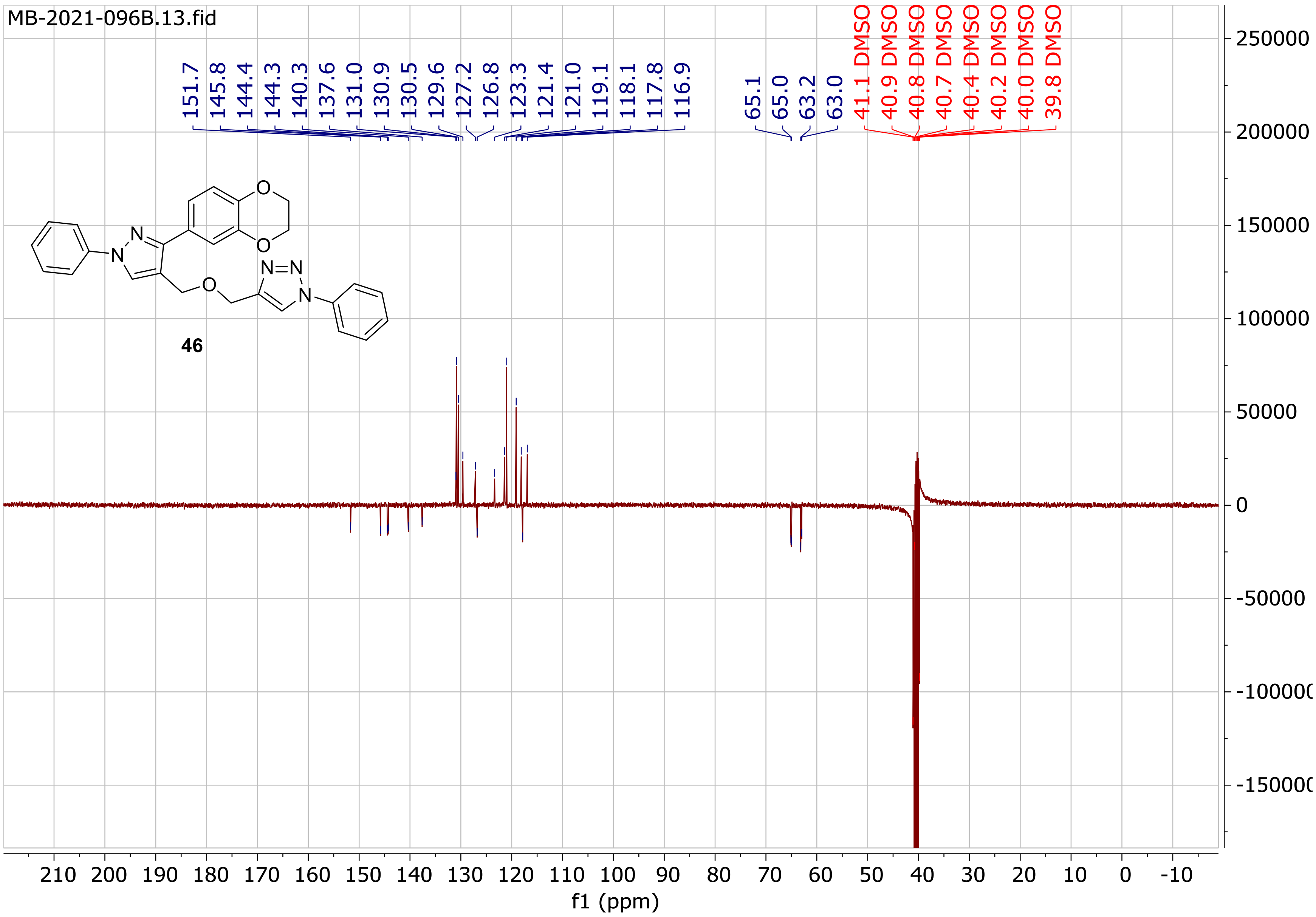
MB-2021-096B.13.fid
151.7
145.8
144.4
144.3
140.3
137.6
131.0
130.9
130.5
129.6
127.2
126.8
123.3
121.4
121.0
119.1
118.1
117.8
116.9
65.1
65.0
63.2
63.0
41.1 DMSO
40.9 DMSO
40.8 DMSO
40.7 DMSO
40.4 DMSO
40.2 DMSO
40.0 DMSO
39.8 DMSO
46
250000
200000
150000
100000
50000
0
-50000
210 200 190 180 170 160 150 140 130 120 110 100 90 80 70 60 50 40 30 20 10 0 -10
f1 (ppm)

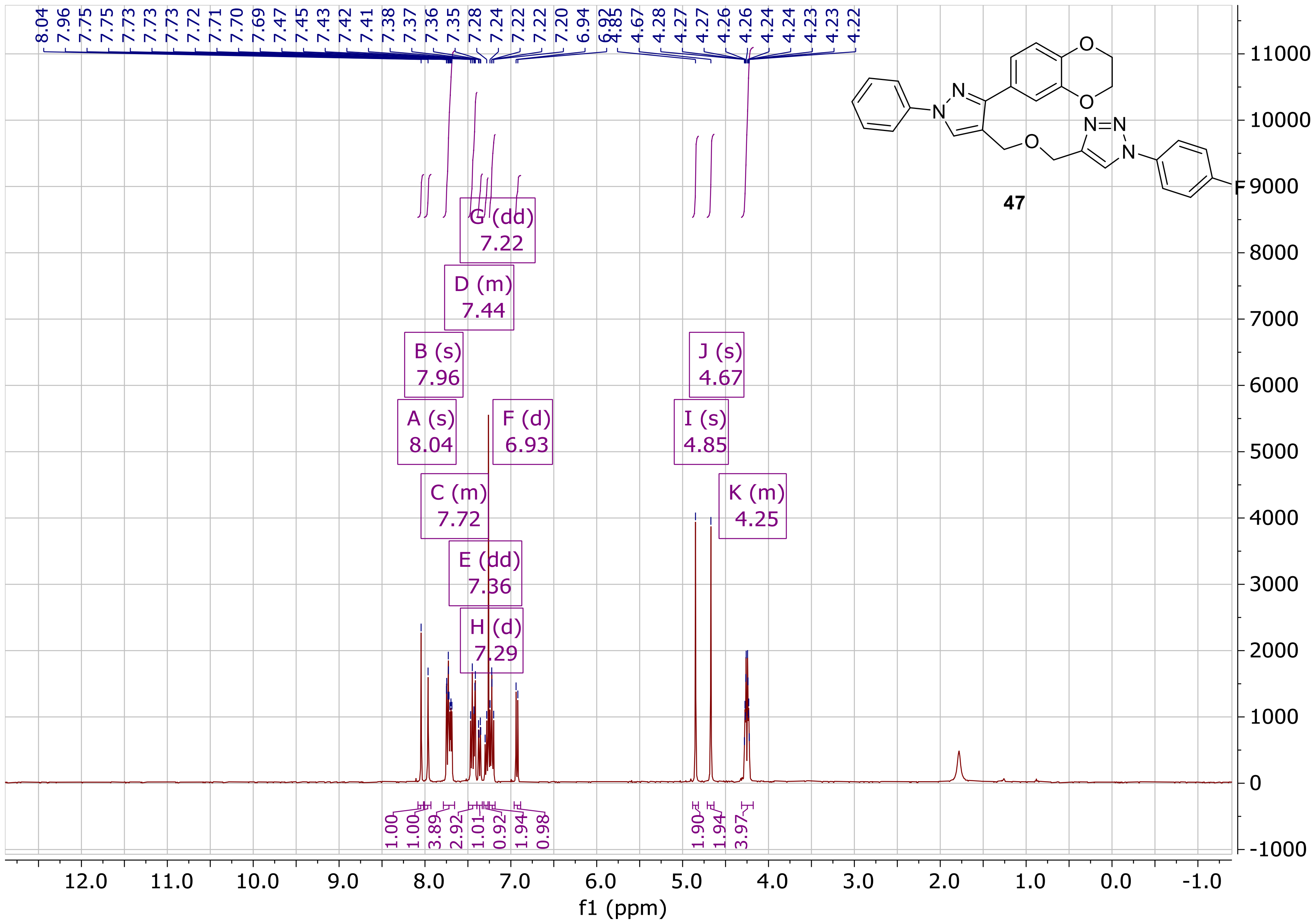
8.04 7.96 7.75 7.75 7.73 7.73 7.73 7.72 7.71 7.70 7.69 7.47 7.45 7.43 7.42 7.41 7.38 7.37 7.36 7.35 7.28 7.24 7.22 7.22 7.20 6.94 6.92 4.85 4.67 4.28 4.27 4.27 4.26 4.26 4.24 4.24 4.23 4.23 4.22
G (dd) 7.22
D (m) 7.44
B (s) 7.96
A (s) 8.04
C (m) 7.72
E (dd) 7.36
H (d) 7.29
F (d) 6.93
J (s) 4.67
I (s) 4.85
K (m) 4.25
1.00 1.00 3.89 2.92 1.01 0.92 1.94 0.98 1.90 1.94 3.97
47
f1 (ppm)

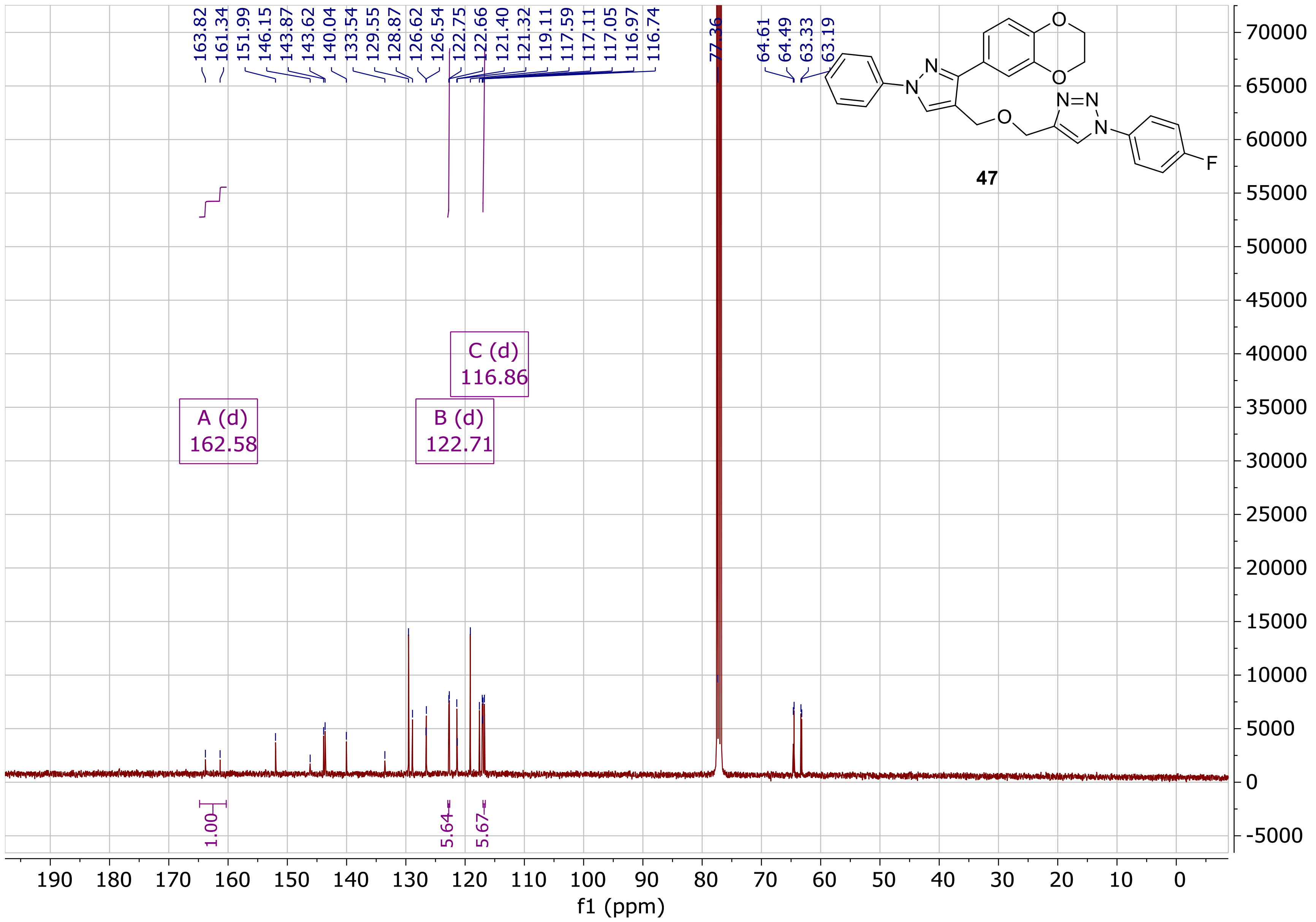

163.82
161.34
151.99
146.15
143.87
143.62
140.04
133.54
129.55
128.87
126.62
126.54
122.75
122.66
121.40
121.32
119.11
117.59
117.11
117.05
116.97
116.74
77.36
64.61
64.49
63.33
63.19
A (d)
162.58
B (d)
122.71
C (d)
116.86
1.00
5.64
5.67
47
f1 (ppm)

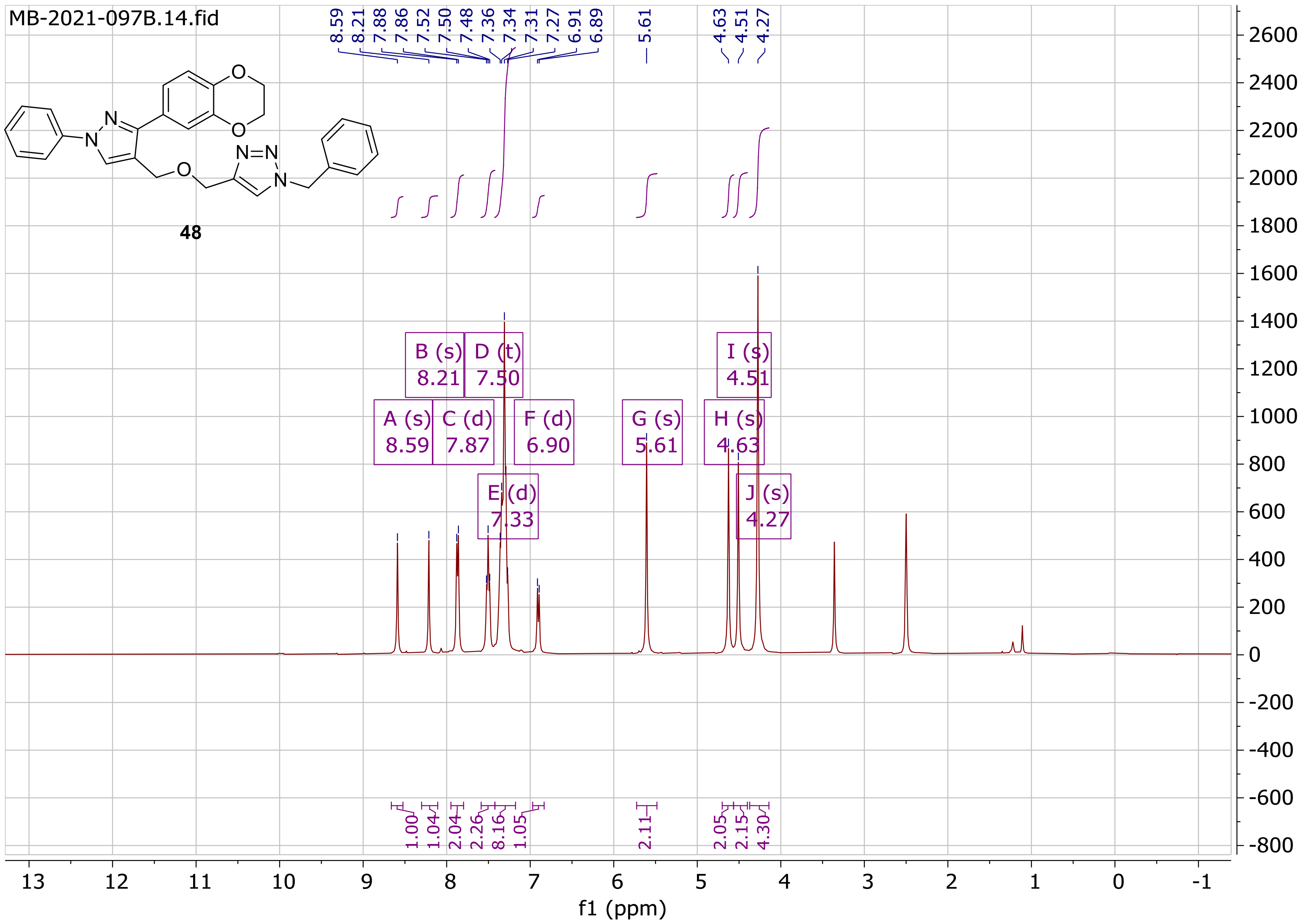
MB-2021-097B.14.fid
8.59
8.21
7.88
7.86
7.52
7.50
7.48
7.36
7.34
7.31
7.27
6.91
6.89
5.61
4.63
4.51
4.27
48
A (s) 8.59
B (s) 8.21
C (d) 7.87
D (t) 7.50
E (d) 7.33
F (d) 6.90
G (s) 5.61
H (s) 4.63
I (s) 4.51
J (s) 4.27
1.00
1.04
2.04
2.26
8.16
1.05
2.11
2.05
2.15
4.30
f1 (ppm)

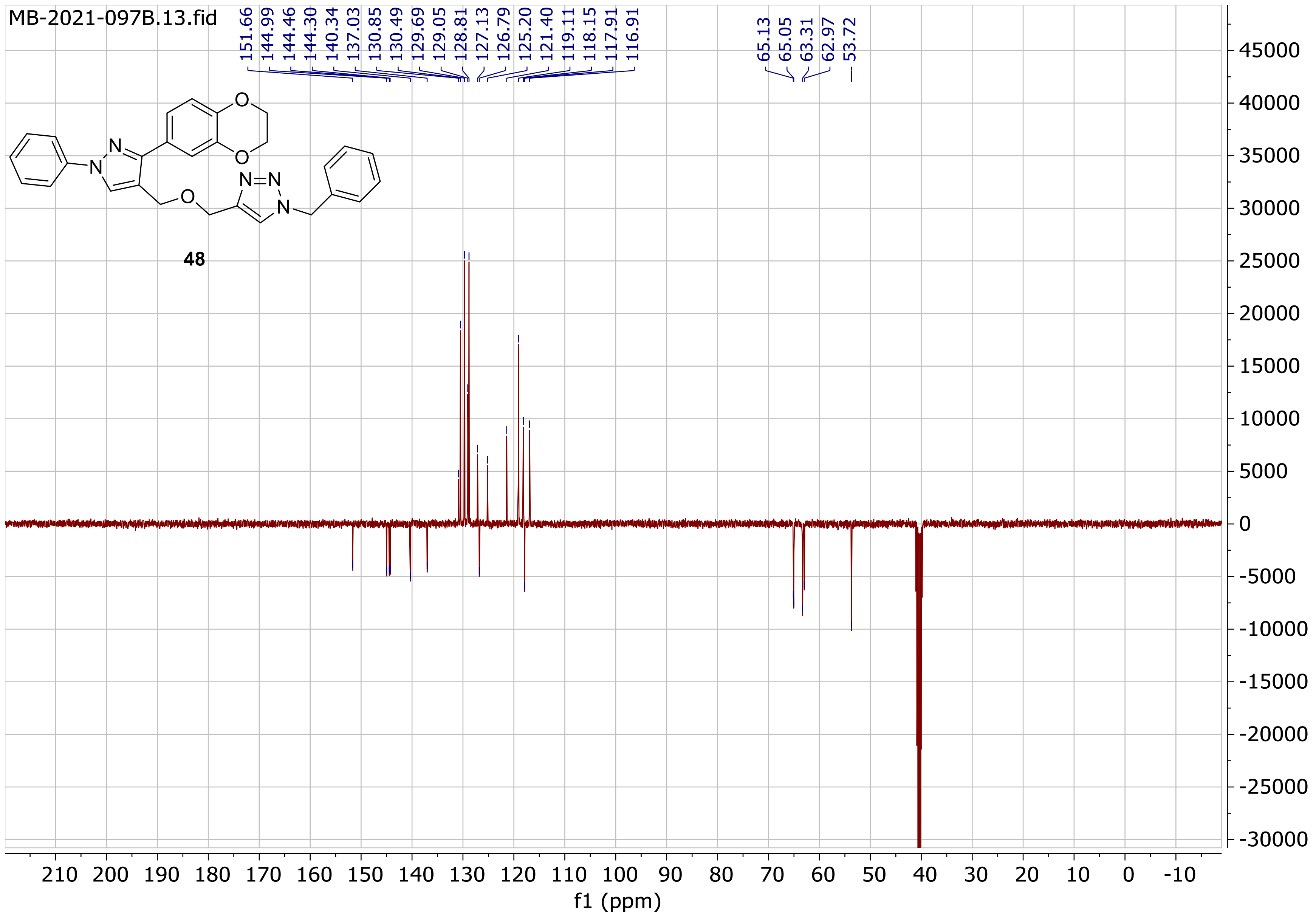
MB-2021-097B.13.fid
151.66
144.99
144.46
144.30
140.34
137.03
130.85
130.49
129.69
129.05
128.81
127.13
126.79
125.20
121.40
119.11
118.15
117.91
116.91
65.13
65.05
63.31
62.97
53.72
N
N
O
O
O
N=N
N
48
f1 (ppm)

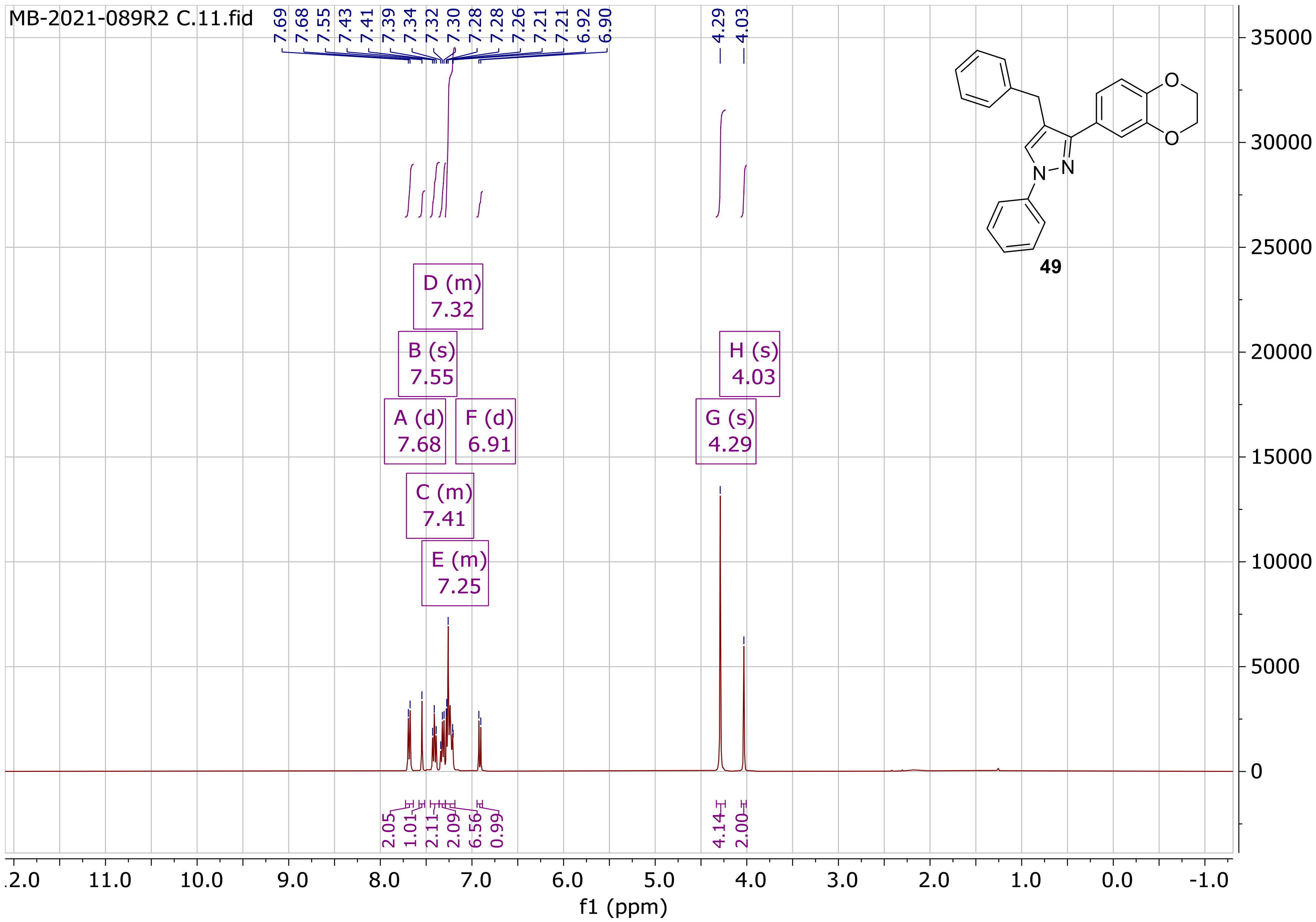
MB-2021-089R2 C.11.fid
7.69 7.68 7.55 7.43 7.41 7.39 7.34 7.32 7.30 7.28 7.28 7.26 7.21 7.21 6.92 6.90
4.29 4.03
D (m) 7.32
B (s) 7.55
A (d) 7.68
F (d) 6.91
C (m) 7.41
E (m) 7.25
H (s) 4.03
G (s) 4.29
2.05 1.01 2.11 2.09 6.56 0.99
4.14 2.00
49
f1 (ppm)

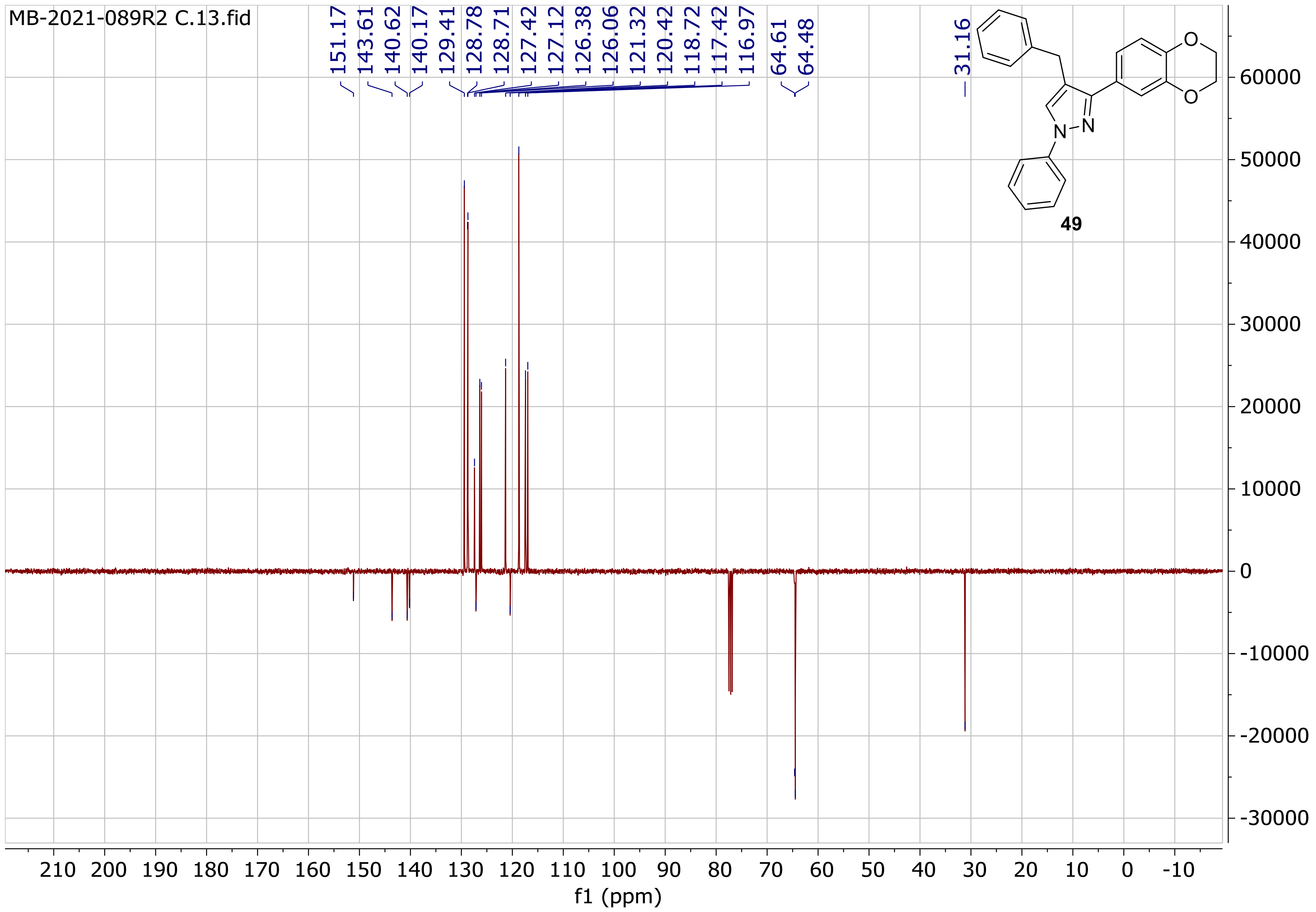
MB-2021-089R2 C.13.fid
151.17
143.61
140.62
140.17
129.41
128.78
128.71
127.42
127.12
126.38
126.06
121.32
120.42
118.72
117.42
116.97
64.61
64.48
31.16
O
O
N
N
49
60000
50000
40000
30000
20000
10000
0
-10000
-20000
-30000
210 200 190 180 170 160 150 140 130 120 110 100 90 80 70 60 50 40 30 20 10 0 -10
f1 (ppm)

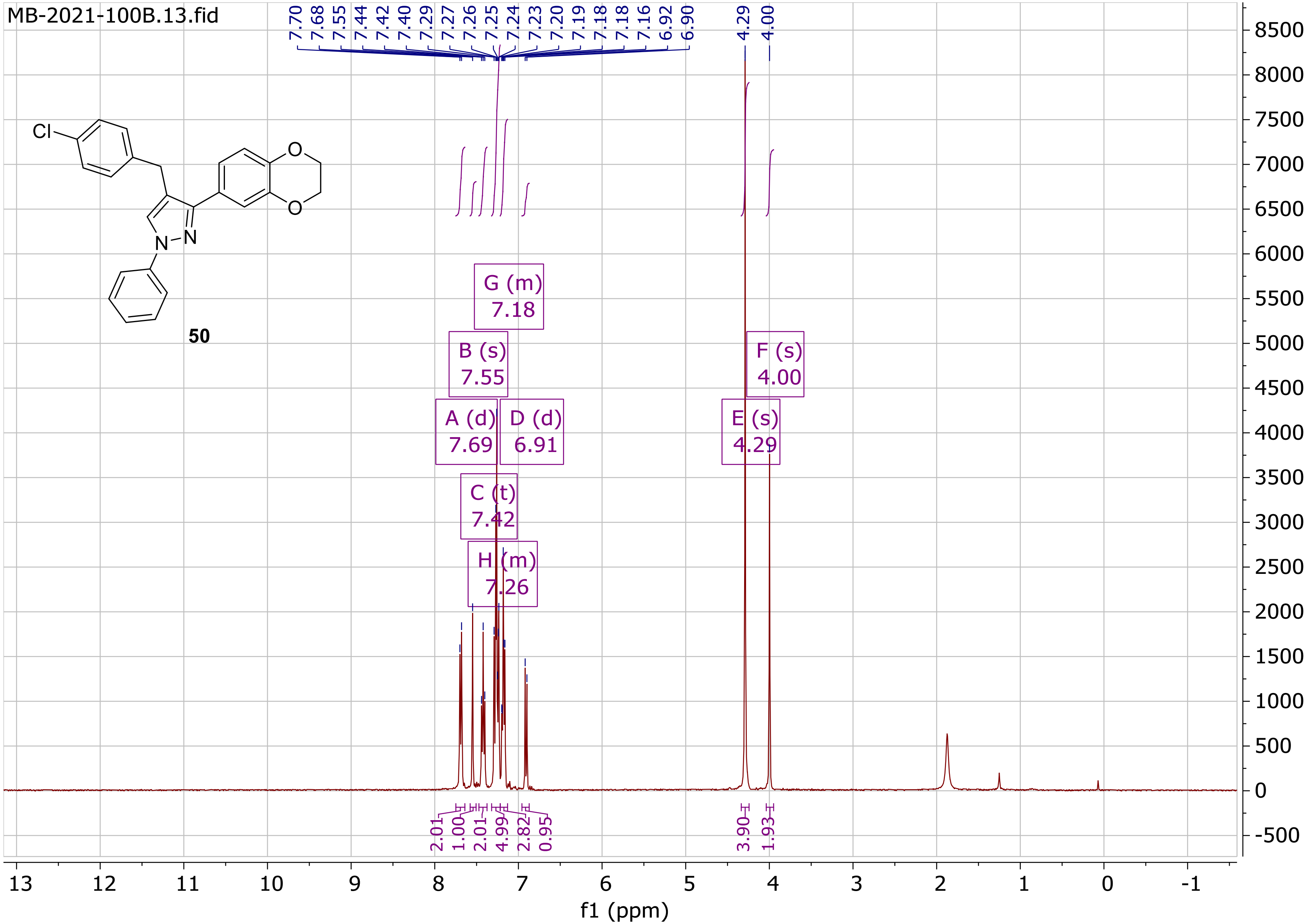

MB-2021-100B.13.fid
7.70 7.68 7.55 7.44 7.42 7.40 7.29 7.27 7.26 7.25 7.24 7.23 7.20 7.19 7.18 7.18 7.16 6.92 6.90
4.29 4.00
Cl
O
O
N
N
50
G (m) 7.18
B (s) 7.55
A (d) 7.69
D (d) 6.91
C (t) 7.42
H (m) 7.26
E (s) 4.29
F (s) 4.00
2.01 1.00 2.01 4.99 2.82 0.95
3.90 1.93
8500 8000 7500 7000 6500 6000 5500 5000 4500 4000 3500 3000 2500 2000 1500 1000 500 0 -500
13 12 11 10 9 8 7 6 5 4 3 2 1 0 -1
f1 (ppm)

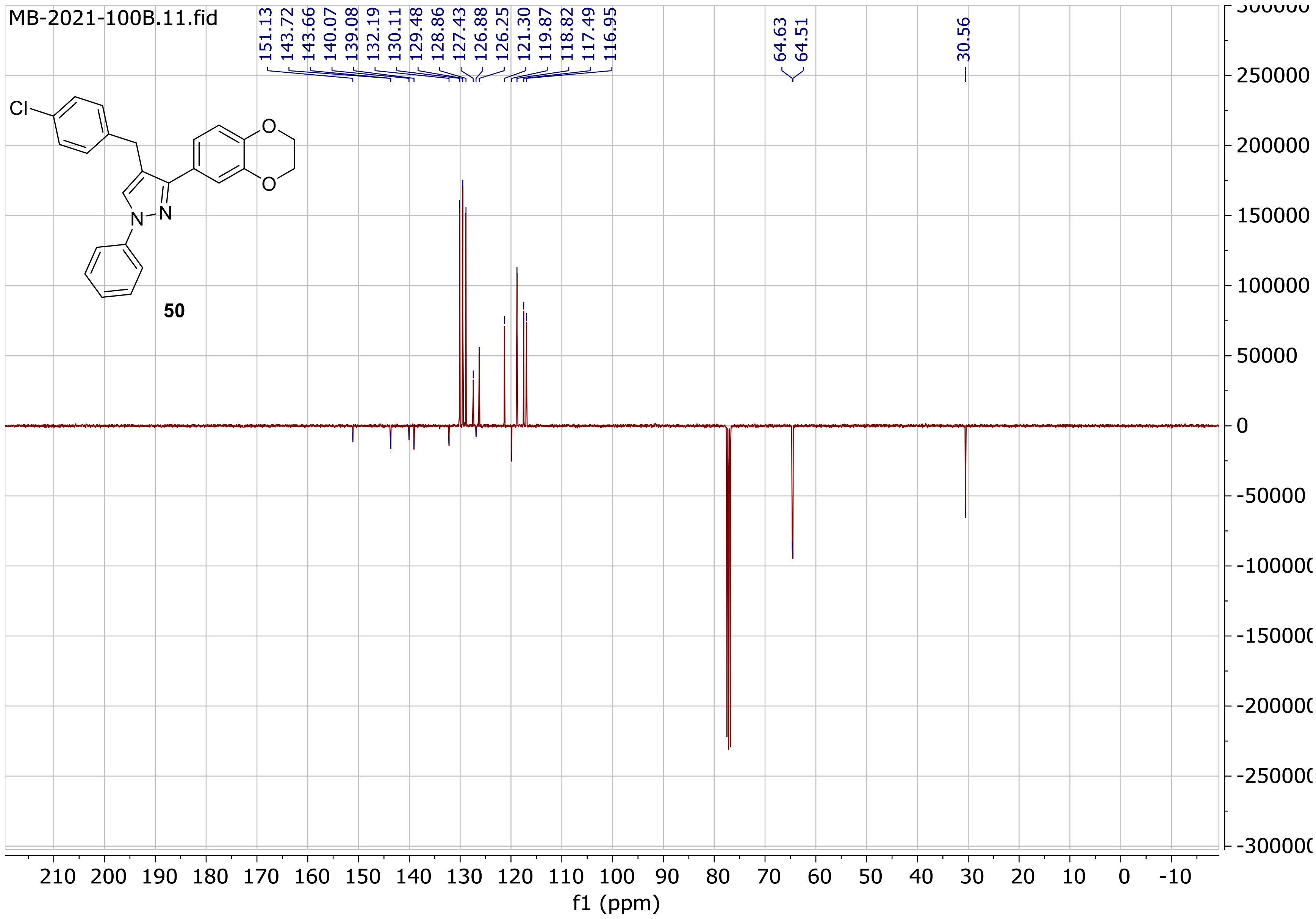
MB-2021-100B.11.fid
151.13
143.72
143.66
140.07
139.08
132.19
130.11
129.48
128.86
127.43
126.88
126.25
121.30
119.87
118.82
117.49
116.95
64.63
64.51
30.56
Cl
O
O
N
N
50
f1 (ppm)

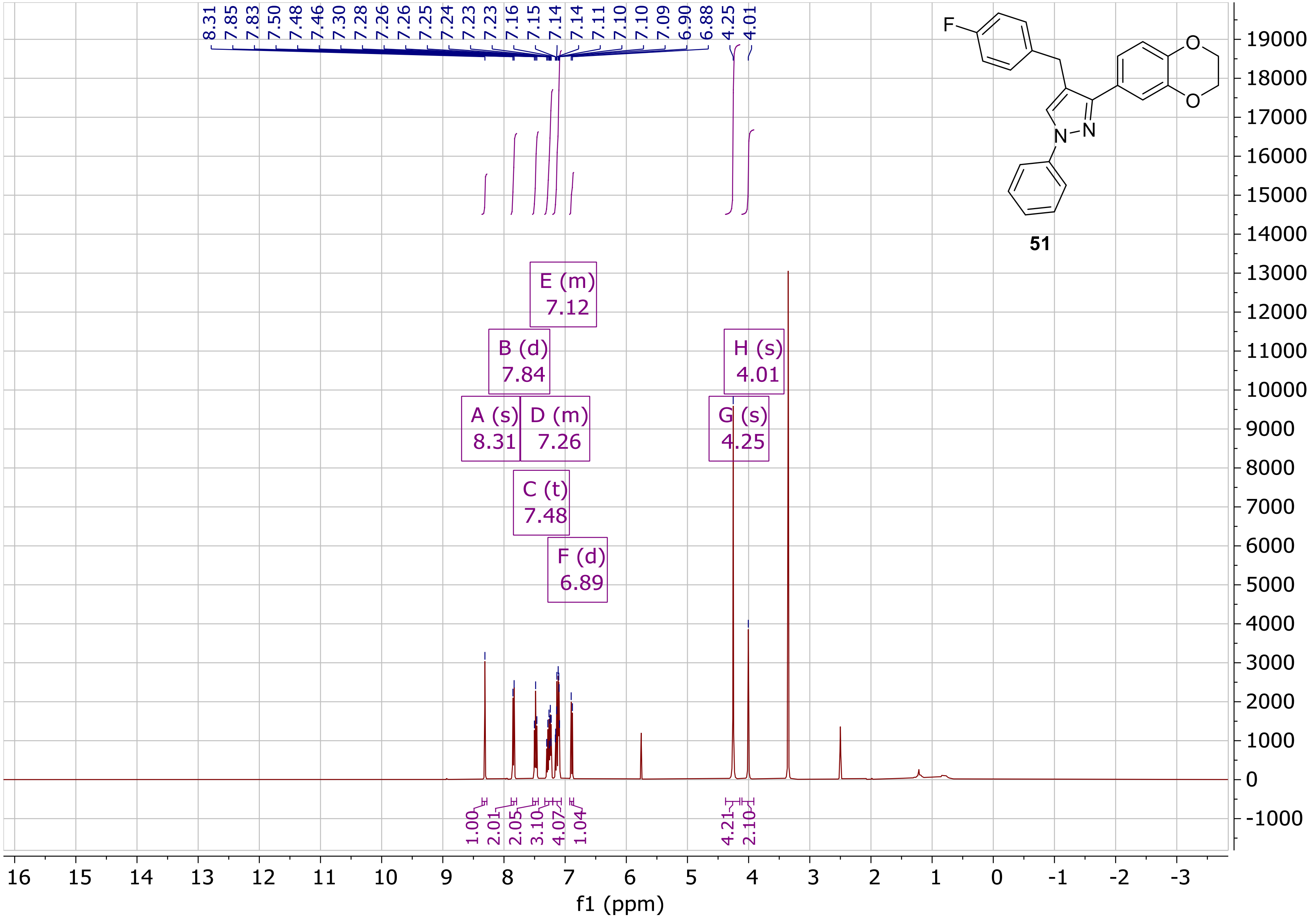

8.31 7.85 7.83 7.50 7.48 7.46 7.30 7.28 7.26 7.26 7.25 7.24 7.23 7.23 7.16 7.15 7.14 7.14 7.11 7.10 7.10 7.09 6.90 6.88 4.25 4.01
E (m) 7.12
B (d) 7.84
H (s) 4.01
A (s) 8.31
D (m) 7.26
G (s) 4.25
C (t) 7.48
F (d) 6.89
1.00 2.01 2.05 3.10 4.07 1.04 4.21 2.10
F
O
O
N
N
51
f1 (ppm)

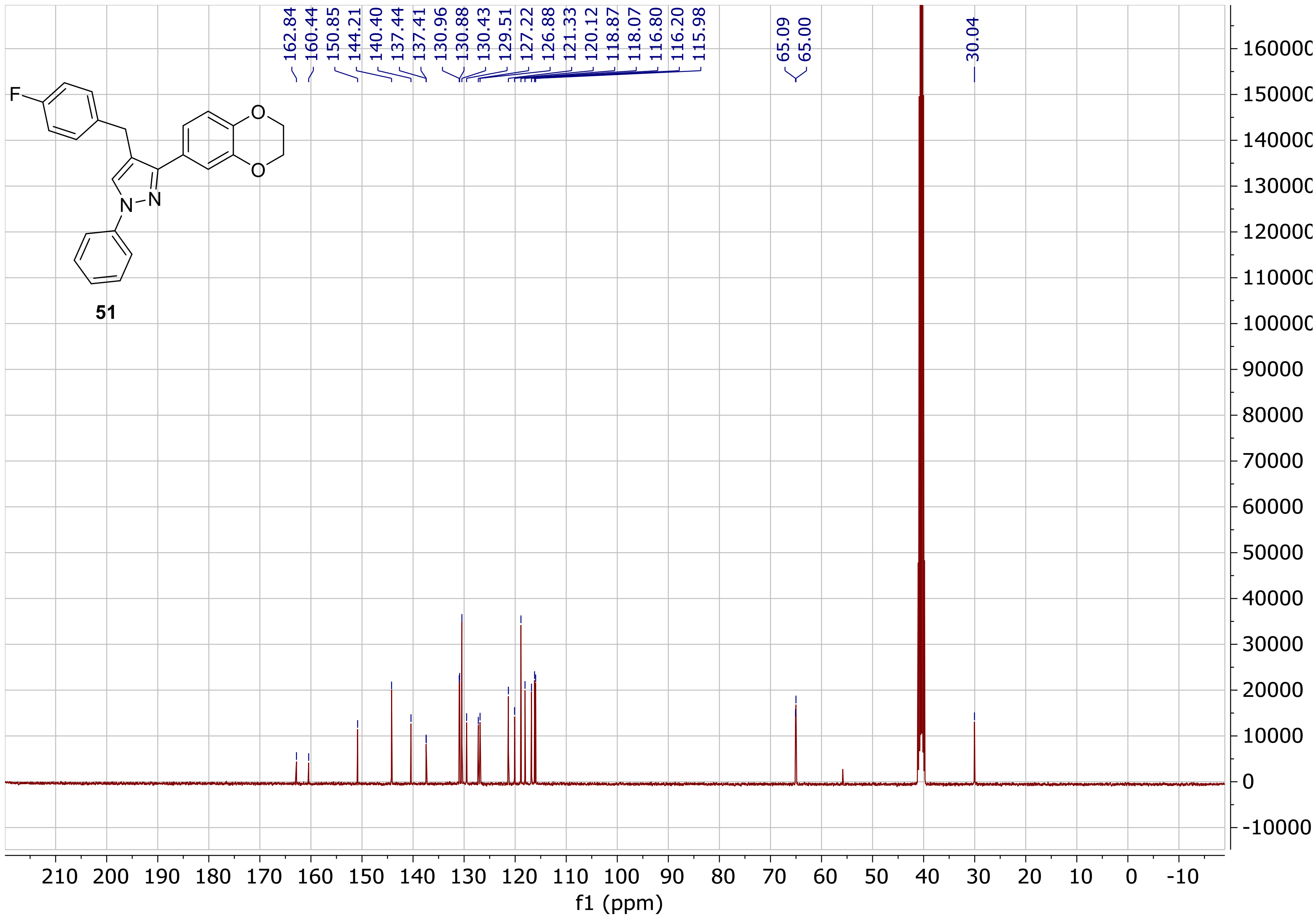

162.84
160.44
150.85
144.21
140.40
137.44
137.41
130.96
130.88
130.43
129.51
127.22
126.88
121.33
120.12
118.87
118.07
116.80
116.20
115.98
65.09
65.00
30.04
F
O
O
N
N
51
210 200 190 180 170 160 150 140 130 120 110 100 90 80 70 60 50 40 30 20 10 0 -10
f1 (ppm)
160000
150000
140000
130000
120000
110000
100000
90000
80000
70000
60000
50000
40000
30000
20000
10000
0
-10000

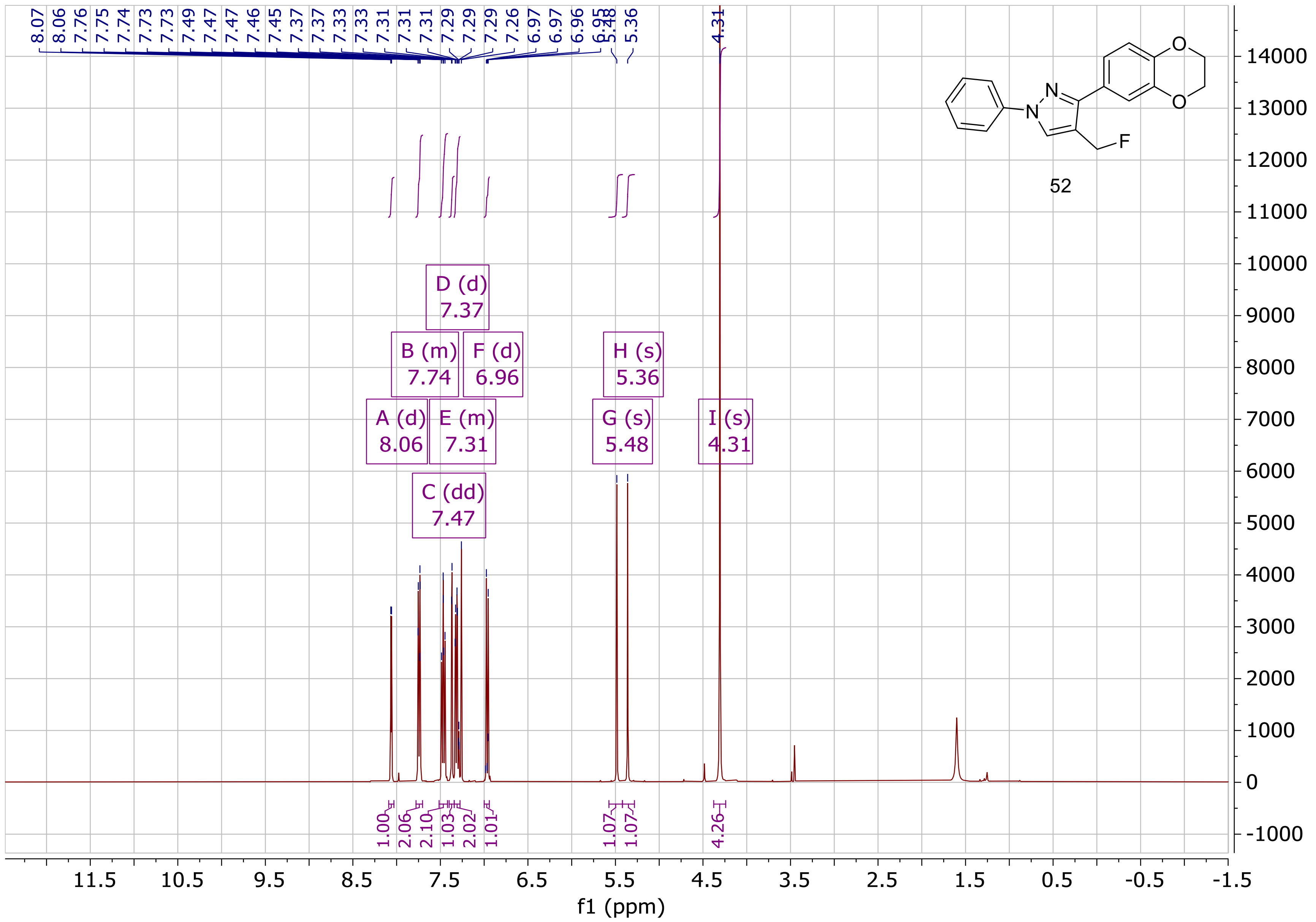
8.07 8.06 7.76 7.75 7.74 7.73 7.73 7.49 7.47 7.47 7.46 7.45 7.37 7.37 7.33 7.33 7.31 7.31 7.31 7.29 7.29 7.29 7.26 6.97 6.97 6.96 6.95 5.48 5.36
4.31
A (d) 8.06
B (m) 7.74
C (dd) 7.47
D (d) 7.37
E (m) 7.31
F (d) 6.96
G (s) 5.48
H (s) 5.36
I (s) 4.31
1.00 2.06 2.10 1.03 2.02 1.01 1.07 1.07 4.26
f1 (ppm)
N
N
O
O
F
52

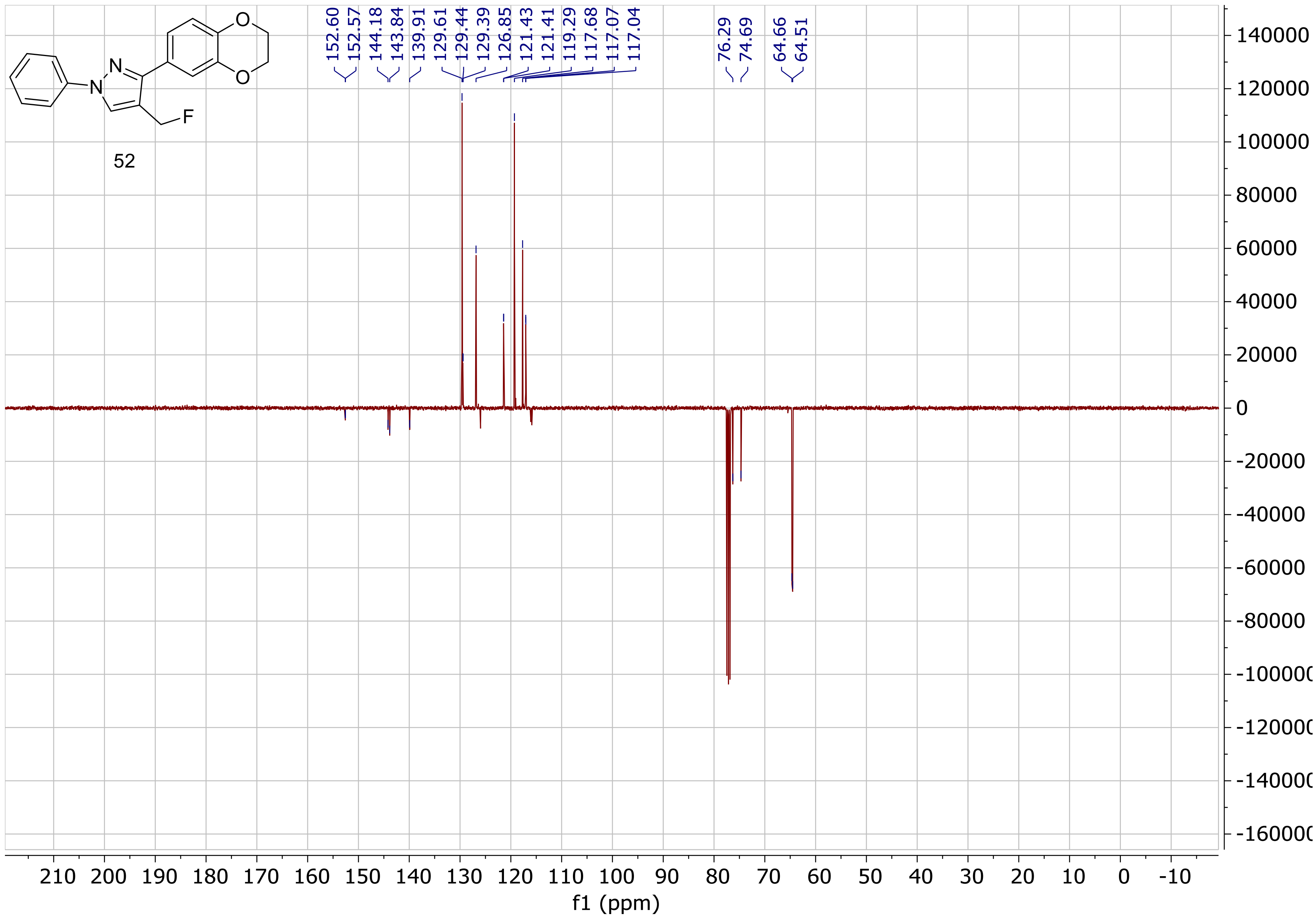
52
152.60
152.57
144.18
143.84
139.91
129.61
129.44
129.39
126.85
121.43
121.41
119.29
117.68
117.07
117.04
76.29
74.69
64.66
64.51
f1 (ppm)

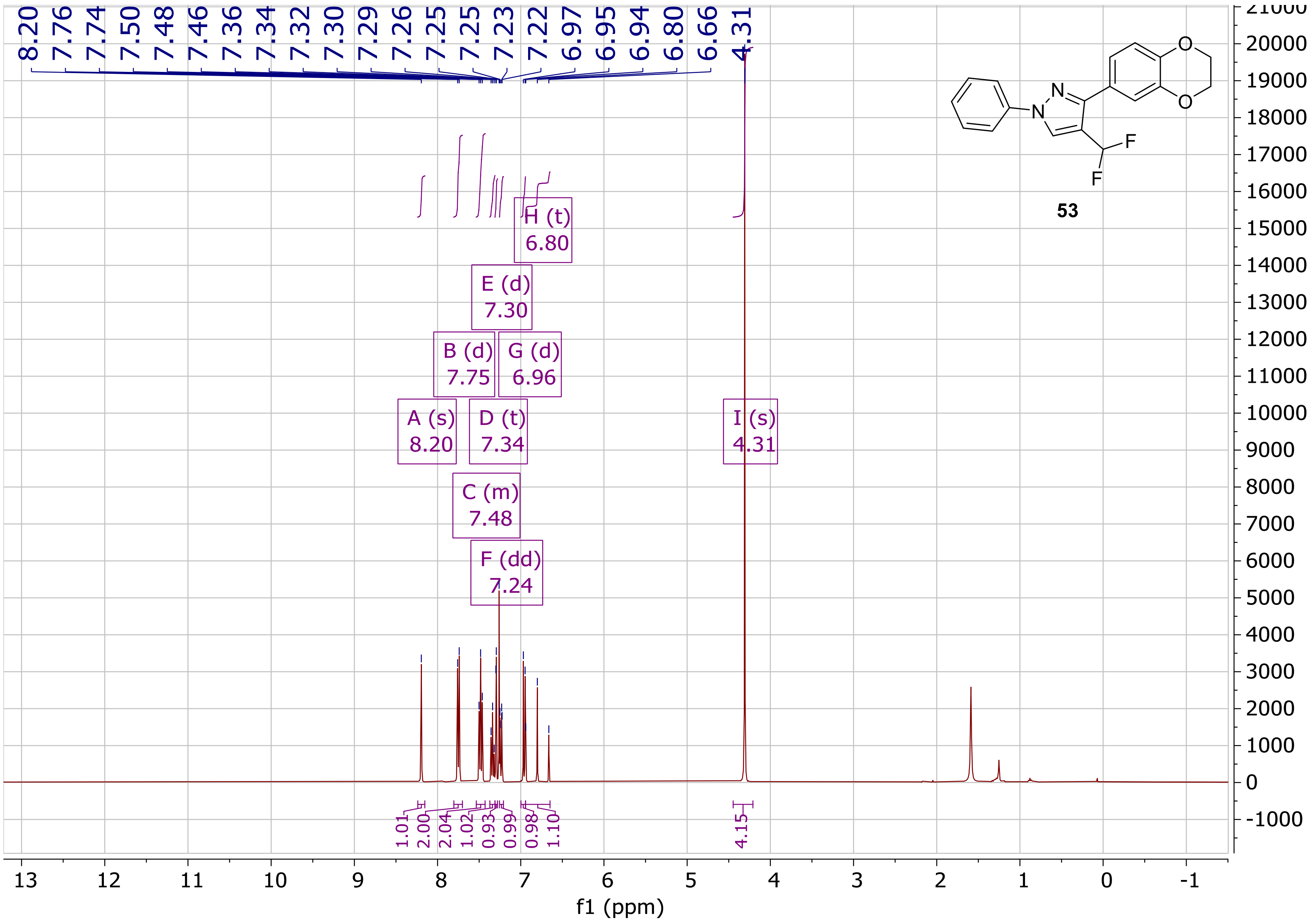

8.20
7.76
7.74
7.50
7.48
7.46
7.36
7.34
7.32
7.30
7.29
7.26
7.25
7.25
7.23
7.22
6.97
6.95
6.94
6.80
6.66
4.31
A (s) 8.20
B (d) 7.75
C (m) 7.48
D (t) 7.34
E (d) 7.30
F (dd) 7.24
G (d) 6.96
H (t) 6.80
I (s) 4.31
1.01
2.00
2.04
1.02
0.93
0.99
0.98
1.10
4.15
f1 (ppm)

53

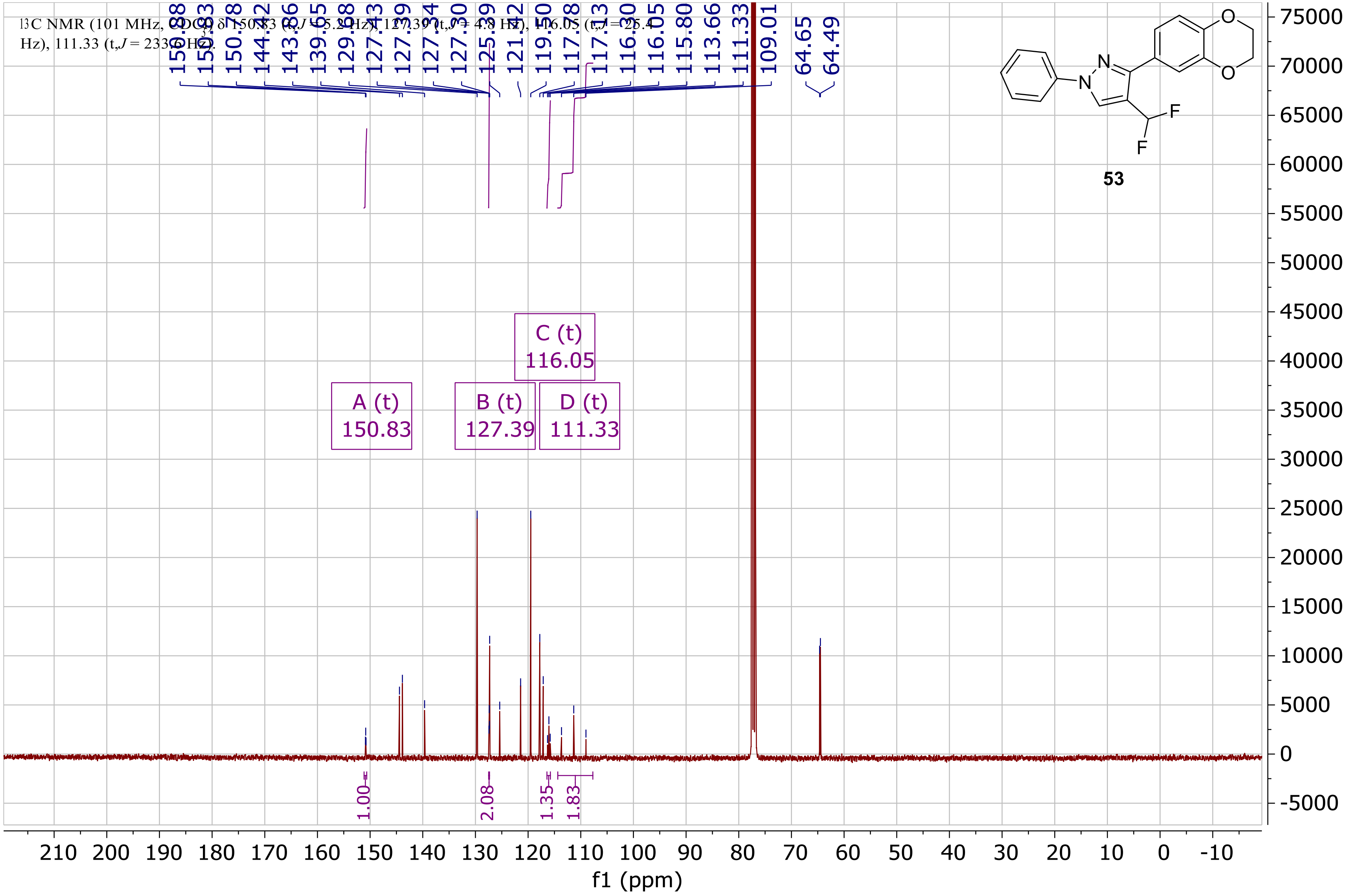
13C NMR (101 MHz, CDCl3) δ 150.83 (t, J = 5.2 Hz), 127.39 (t, J = 4.8 Hz), 116.05 (t, J = 25.4 Hz), 111.33 (t, J = 233.6 Hz).
150.88
150.83
150.78
144.42
143.86
139.55
129.58
127.43
127.39
127.34
127.30
125.39
121.42
119.50
117.78
117.13
116.30
116.05
115.80
113.66
111.33
109.01
64.65
64.49
A (t) 150.83
B (t) 127.39
C (t) 116.05
D (t) 111.33
1.00
2.08
1.35
1.83
53
f1 (ppm)

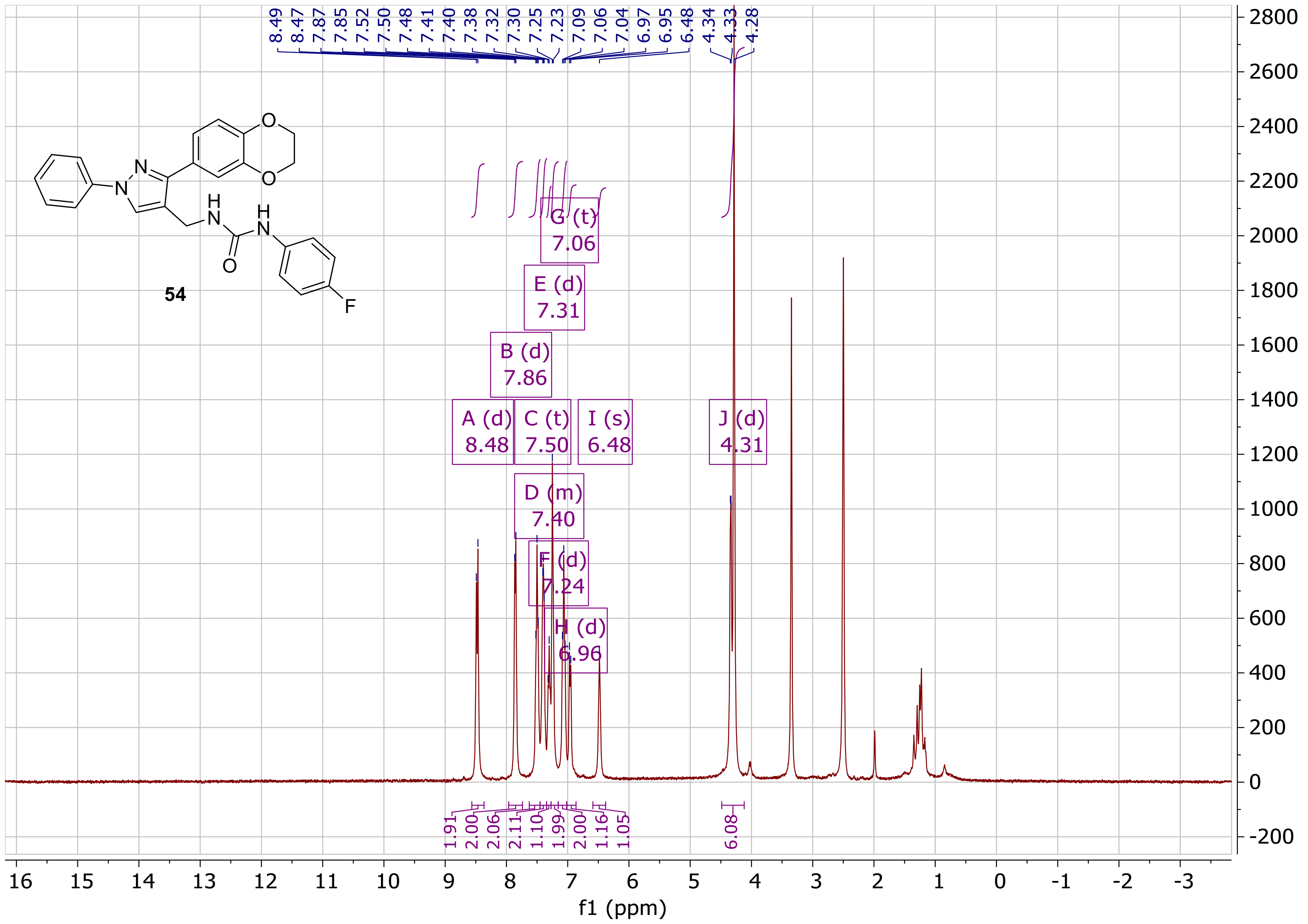

54
8.49
8.47
7.87
7.85
7.52
7.50
7.48
7.41
7.40
7.38
7.32
7.30
7.25
7.23
7.09
7.06
7.04
6.97
6.95
6.48
4.34
4.33
4.28
A (d) 8.48
B (d) 7.86
C (t) 7.50
D (m) 7.40
E (d) 7.31
F (d) 7.24
G (t) 7.06
H (d) 6.96
I (s) 6.48
J (d) 4.31
1.91
2.00
2.06
2.11
1.10
1.99
2.00
1.16
1.05
6.08
f1 (ppm)

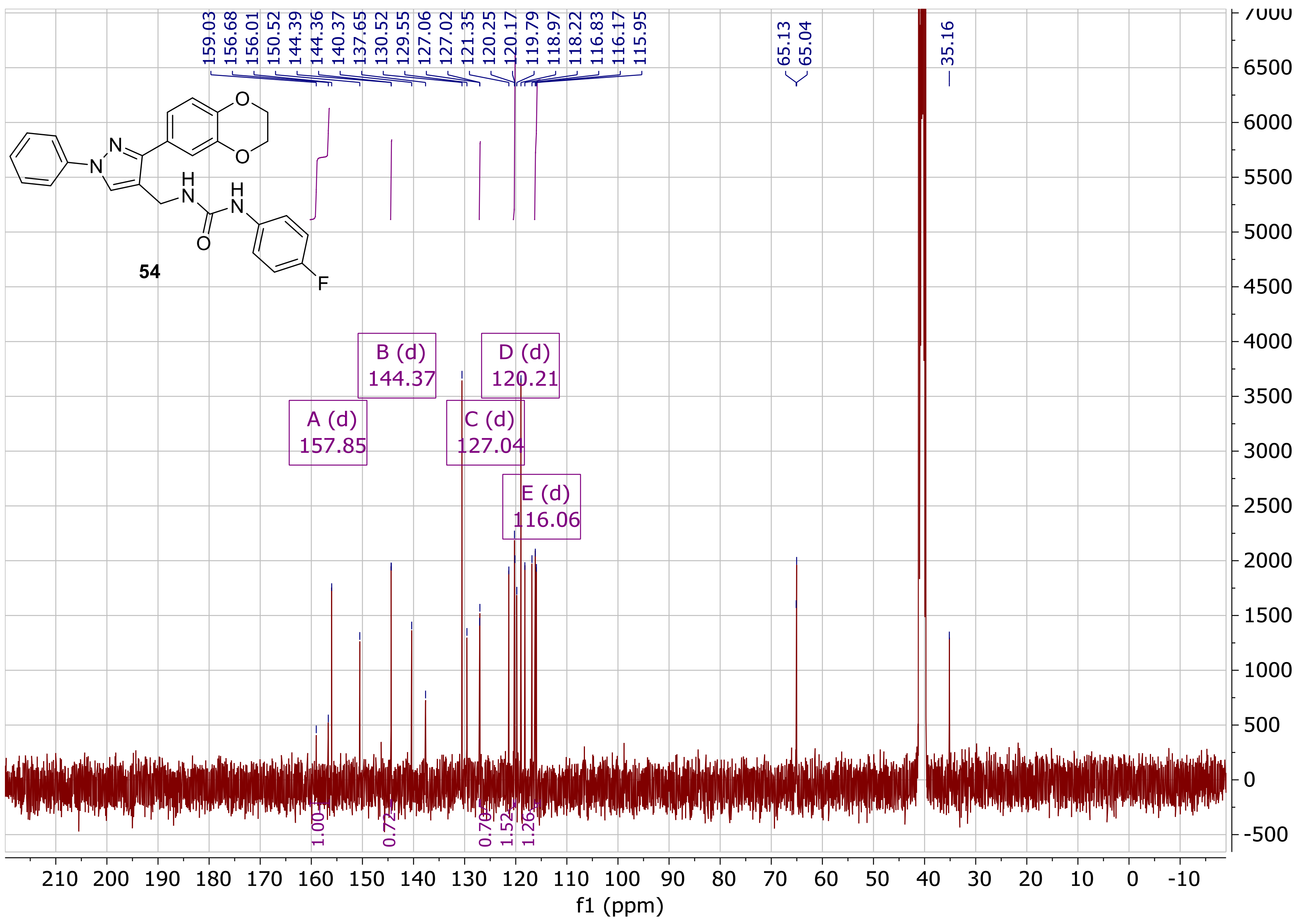
159.03
156.68
156.01
150.52
144.39
144.36
140.37
137.65
130.52
129.55
127.06
127.02
121.35
120.25
120.17
119.79
118.97
118.22
116.83
116.17
115.95
65.13
65.04
35.16
54
A (d)
157.85
B (d)
144.37
C (d)
127.04
D (d)
120.21
E (d)
116.06
1.00
0.72
0.70
1.52
1.26
f1 (ppm)

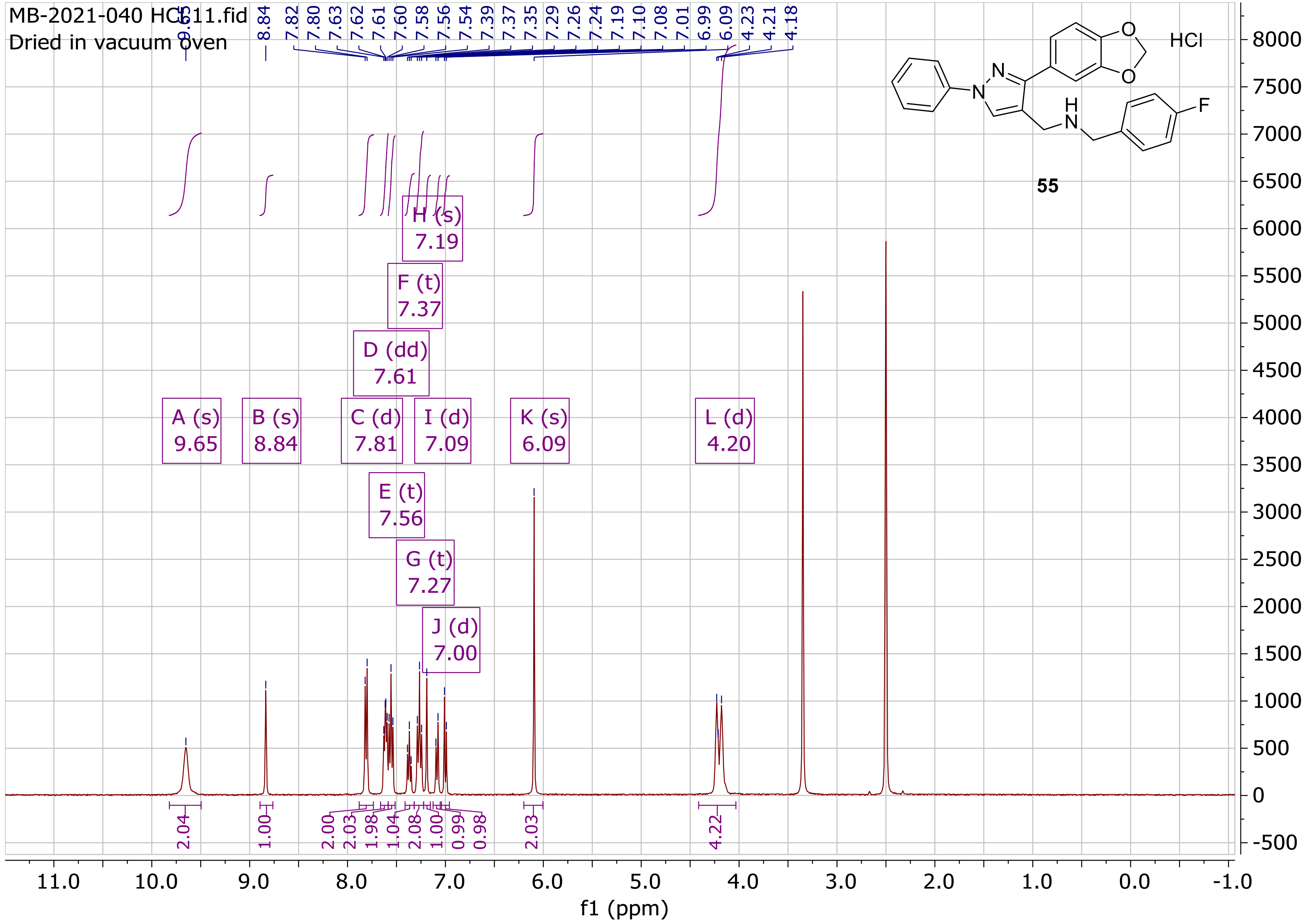
MB-2021-040 HCl11.fid
Dried in vacuum oven
9.65
8.84
7.82
7.80
7.63
7.62
7.61
7.60
7.58
7.56
7.54
7.39
7.37
7.35
7.29
7.26
7.24
7.19
7.10
7.08
7.01
6.99
6.09
4.23
4.21
4.18
HCl
55
A (s) 9.65
B (s) 8.84
C (d) 7.81
D (dd) 7.61
E (t) 7.56
F (t) 7.37
G (t) 7.27
H (s) 7.19
I (d) 7.09
J (d) 7.00
K (s) 6.09
L (d) 4.20
2.04
1.00
2.00
2.03
1.98
1.04
2.08
1.00
0.99
0.98
2.03
4.22
f1 (ppm)

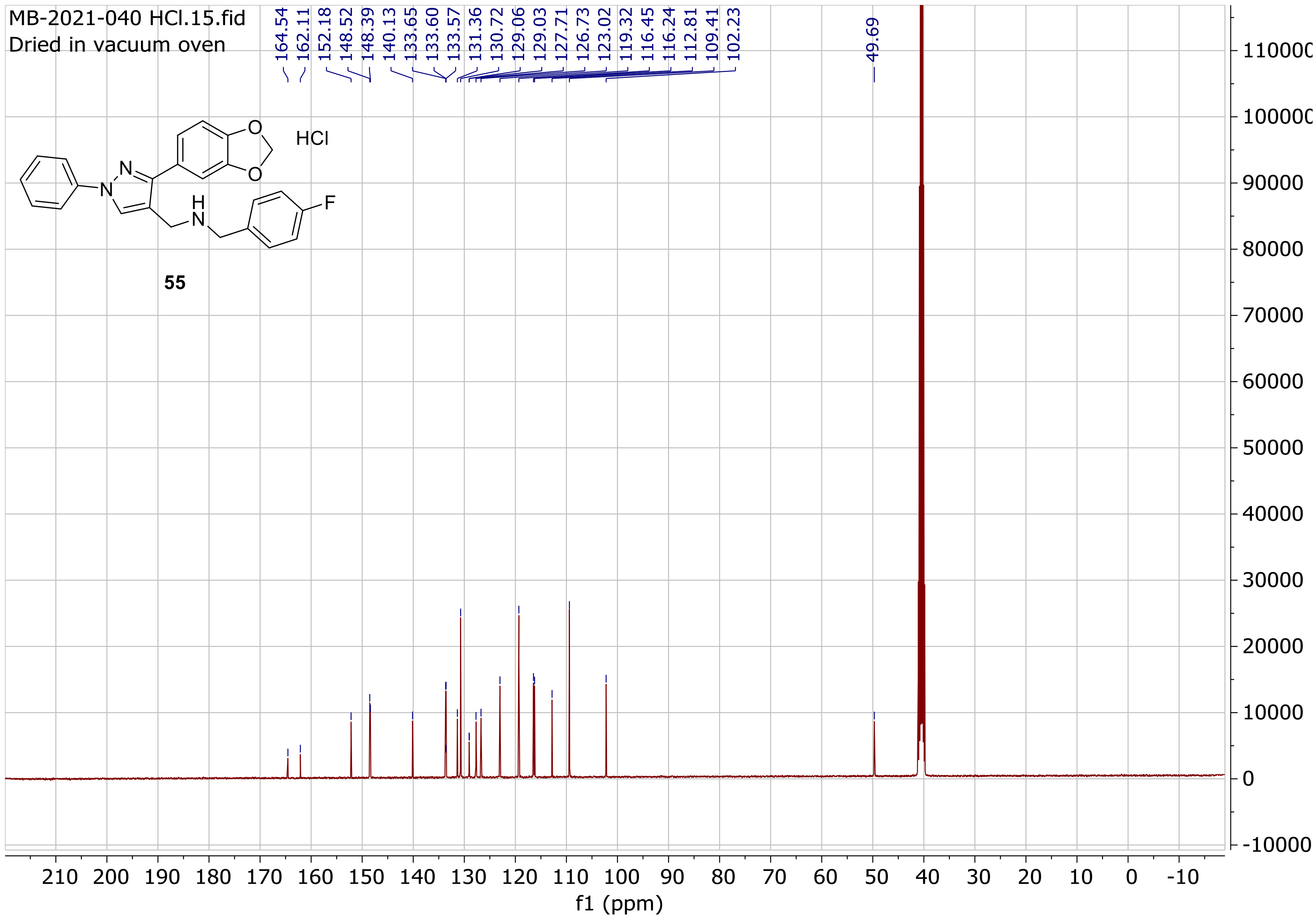

MB-2021-040 HCl.15.fid
Dried in vacuum oven
164.54
162.11
152.18
148.52
148.39
140.13
133.65
133.60
133.57
131.36
130.72
129.06
129.03
127.71
126.73
123.02
119.32
116.45
116.24
112.81
109.41
102.23
49.69
HCl
55
f1 (ppm)

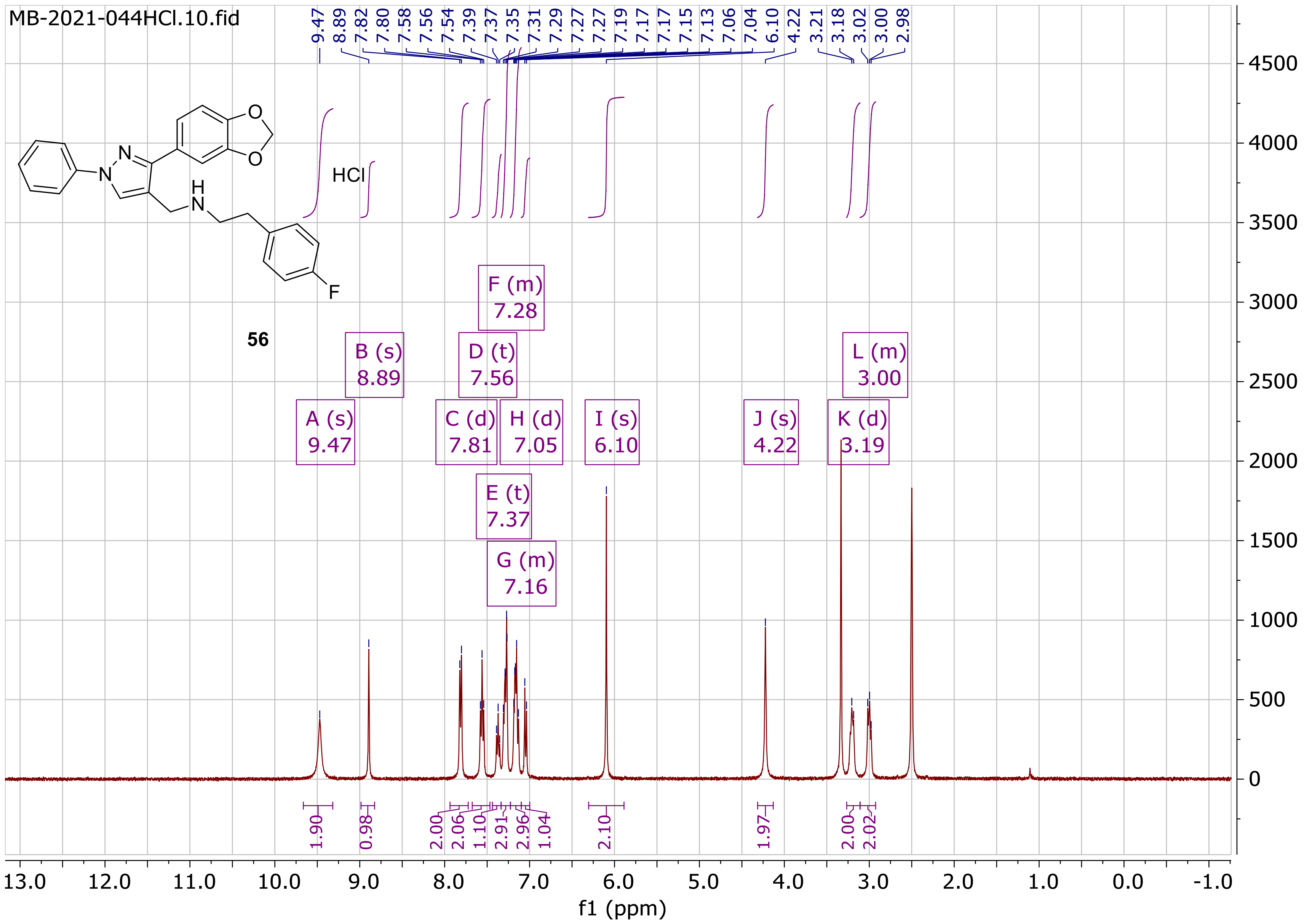

MB-2021-044HCl.10.fid
9.47
8.89
7.82
7.80
7.58
7.56
7.54
7.39
7.37
7.35
7.31
7.29
7.27
7.27
7.19
7.17
7.17
7.15
7.13
7.06
7.04
6.10
4.22
3.21
3.18
3.02
3.00
2.98
HCl
56
F (m) 7.28
B (s) 8.89
D (t) 7.56
L (m) 3.00
A (s) 9.47
C (d) 7.81
H (d) 7.05
I (s) 6.10
J (s) 4.22
K (d) 3.19
E (t) 7.37
G (m) 7.16
1.90
0.98
2.00
2.06
1.10
2.91
2.96
1.04
2.10
1.97
2.00
2.02
f1 (ppm)

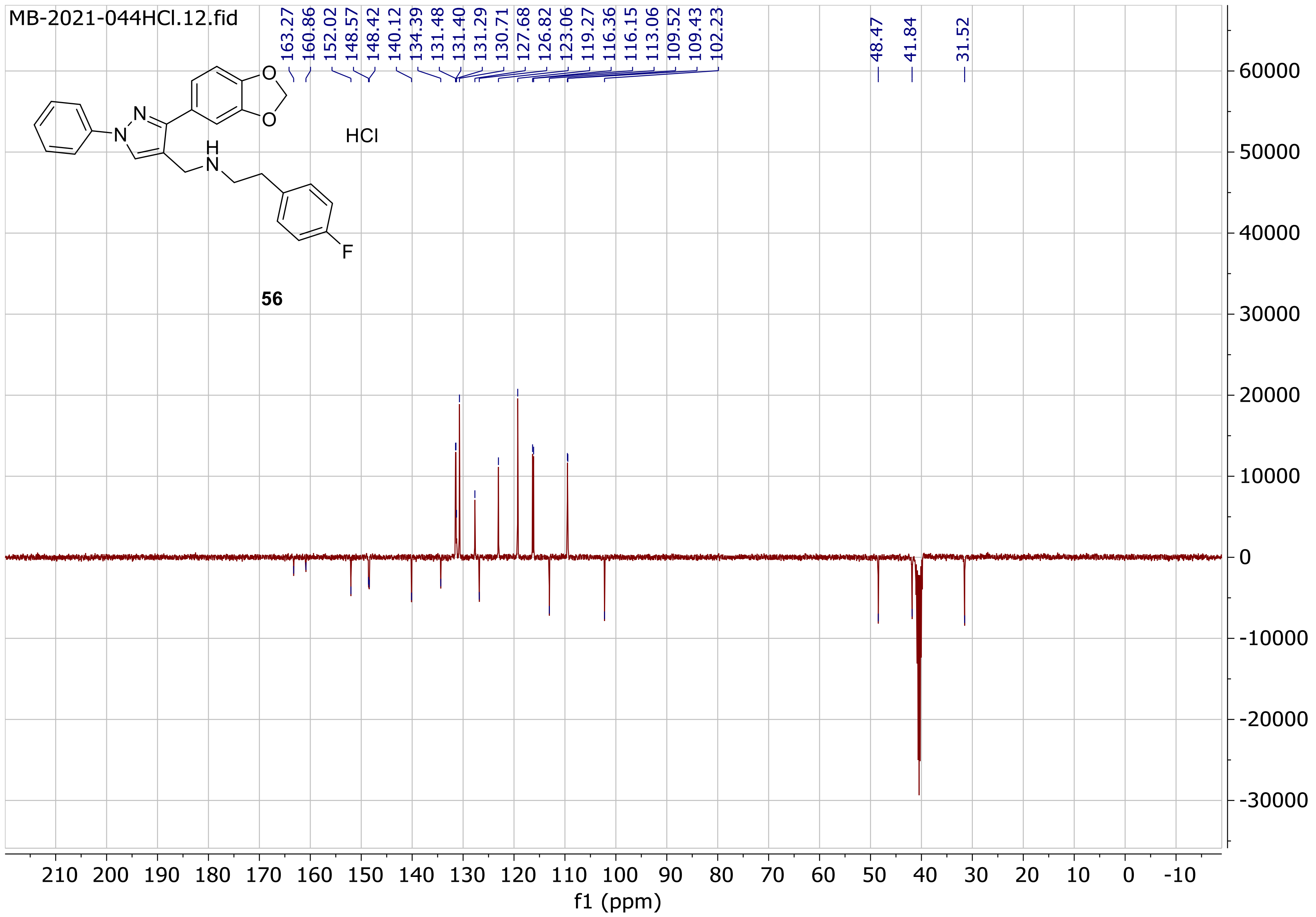
MB-2021-044HCl.12.fid
163.27
160.86
152.02
148.57
148.42
140.12
134.39
131.48
131.40
131.29
130.71
127.68
126.82
123.06
119.27
116.36
116.15
113.06
109.52
109.43
102.23
48.47
41.84
31.52
HCl
56
f1 (ppm)

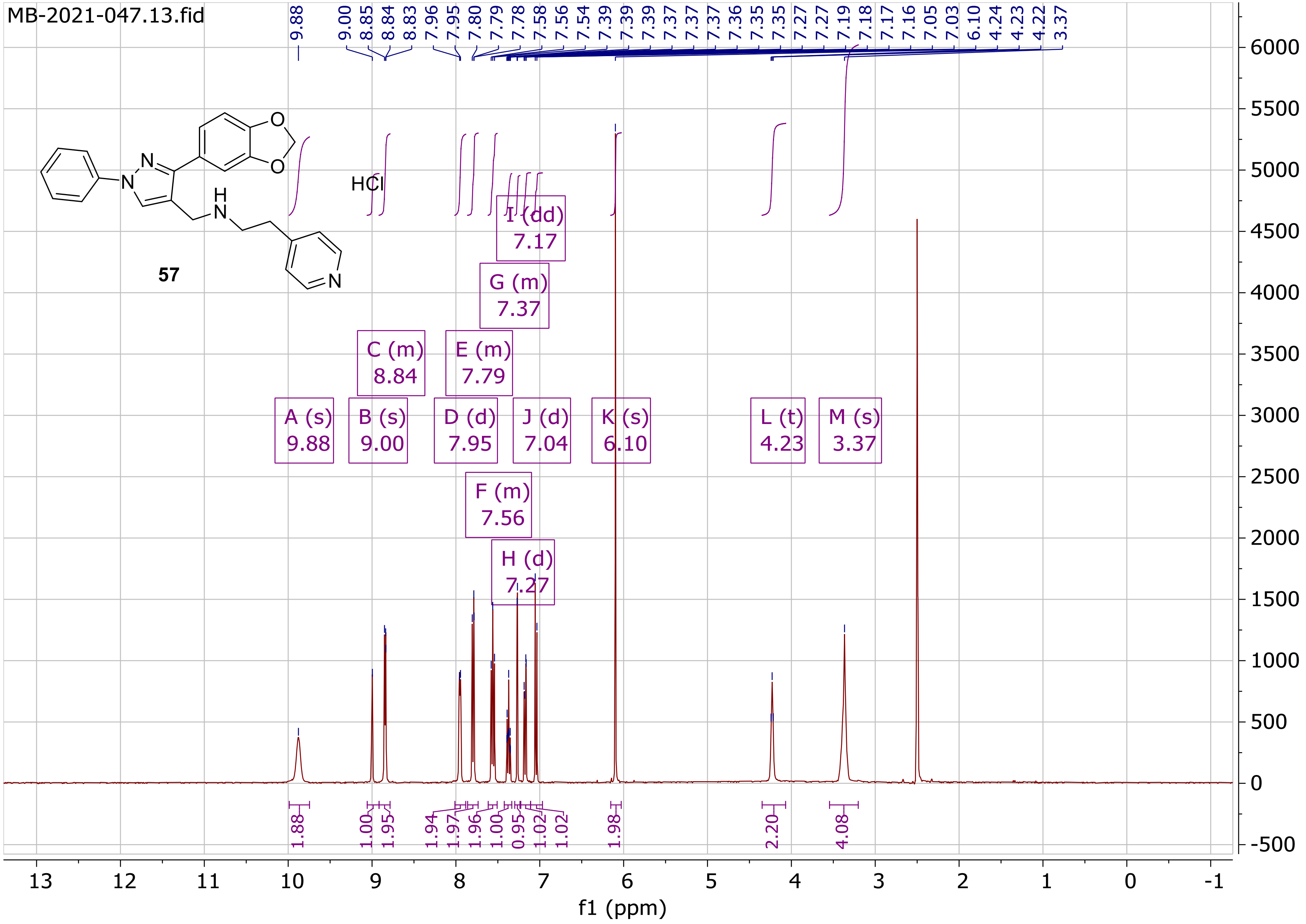

MB-2021-047.13.fid
9.88
9.00
8.85
8.84
8.83
7.96
7.95
7.80
7.79
7.78
7.58
7.56
7.54
7.39
7.39
7.39
7.37
7.37
7.37
7.36
7.35
7.35
7.27
7.27
7.19
7.18
7.17
7.16
7.05
7.03
6.10
4.24
4.23
4.22
3.37
HCl
57
A (s) 9.88
B (s) 9.00
C (m) 8.84
D (d) 7.95
E (m) 7.79
F (m) 7.56
G (m) 7.37
H (d) 7.27
I (dd) 7.17
J (d) 7.04
K (s) 6.10
L (t) 4.23
M (s) 3.37
1.88
1.00
1.95
1.94
1.97
1.96
1.00
0.95
1.02
1.02
1.98
2.20
4.08
f1 (ppm)

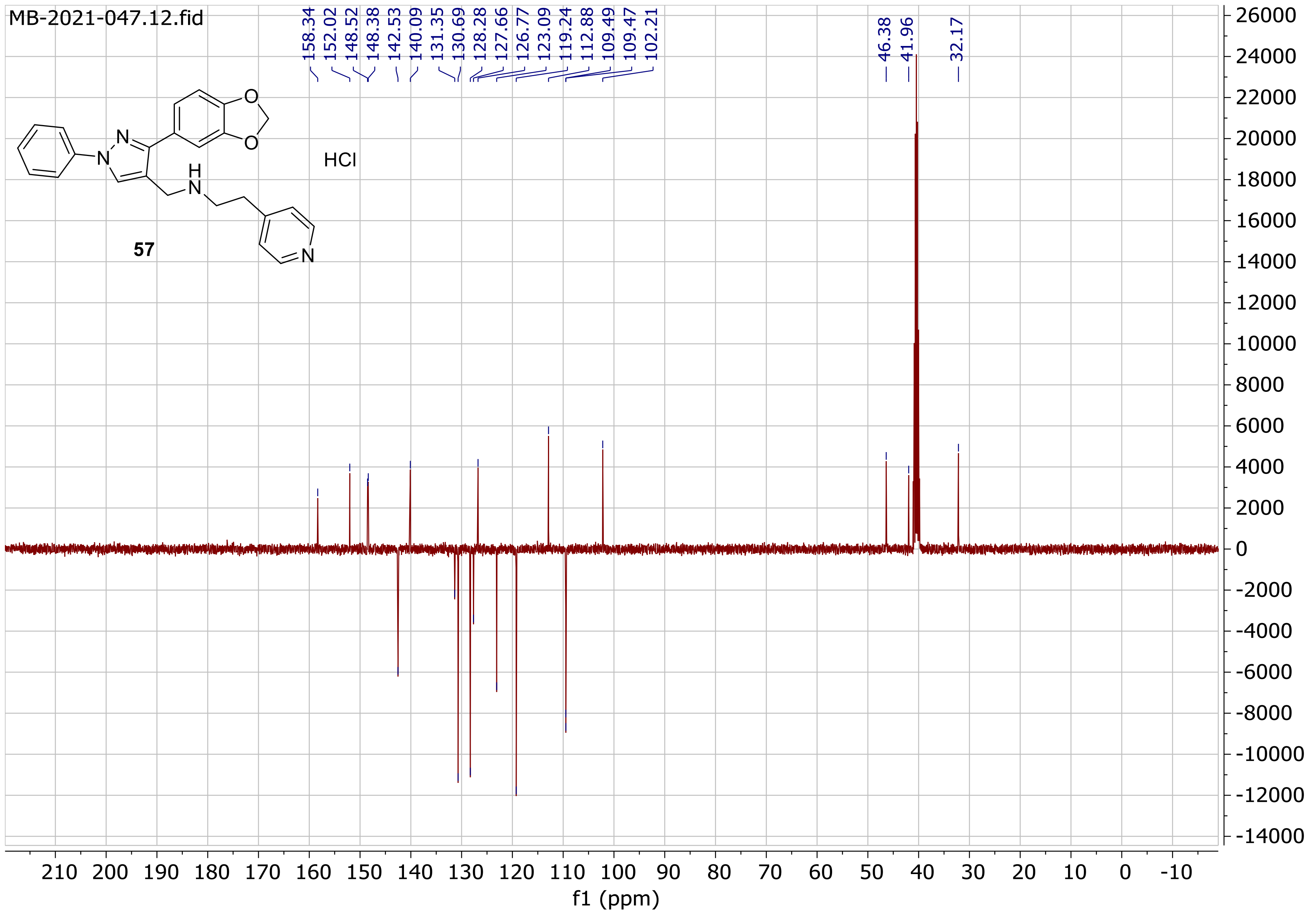

MB-2021-047.12.fid
158.34
152.02
148.52
148.38
142.53
140.09
131.35
130.69
128.28
127.66
126.77
123.09
119.24
112.88
109.49
109.47
102.21
46.38
41.96
32.17
HCl
57
f1 (ppm)

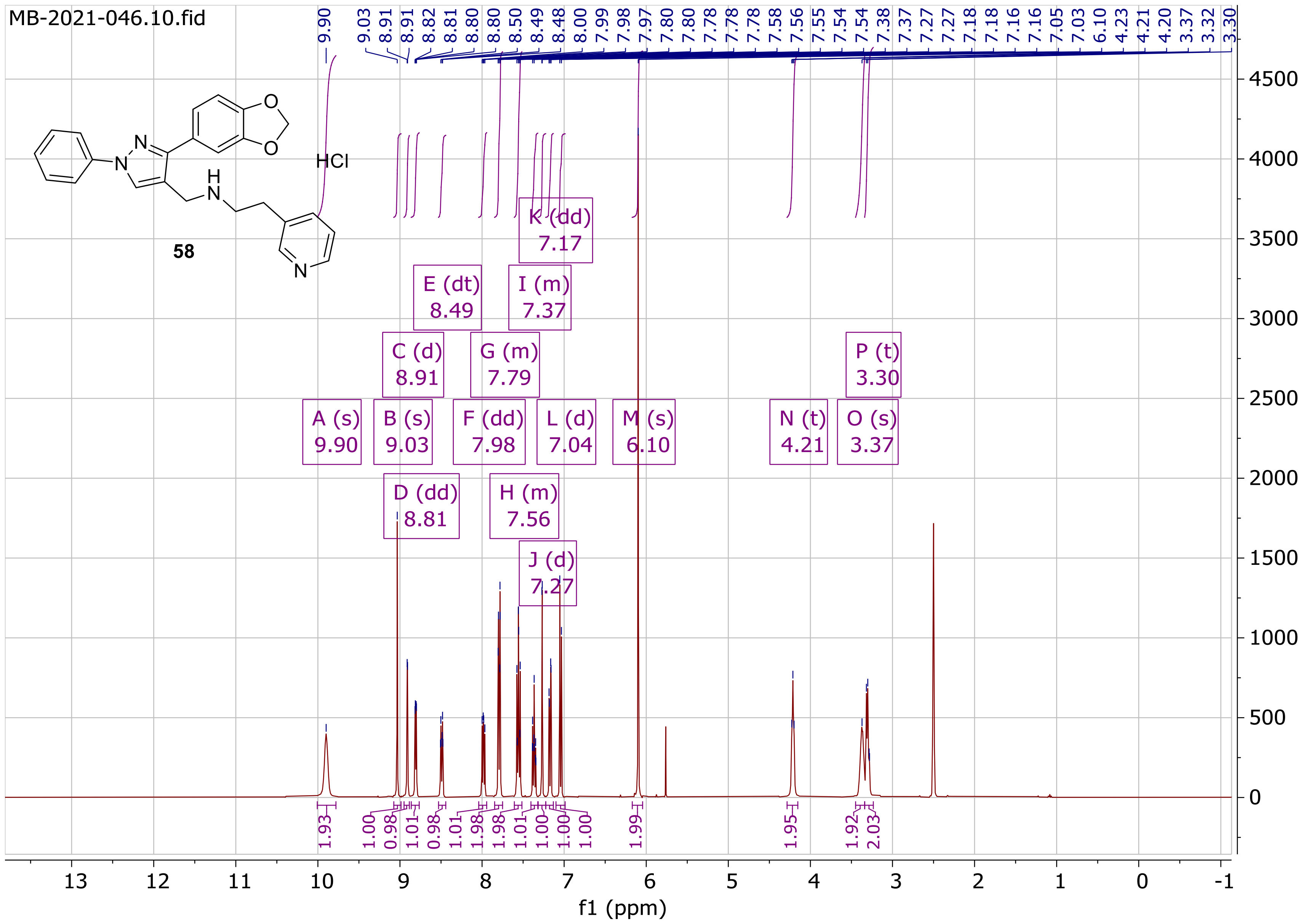
MB-2021-046.10.fid
HCl
58
A (s) 9.90
B (s) 9.03
C (d) 8.91
D (dd) 8.81
E (dt) 8.49
F (dd) 7.98
G (m) 7.79
H (m) 7.56
I (m) 7.37
J (d) 7.27
K (dd) 7.17
L (d) 7.04
M (s) 6.10
N (t) 4.21
O (s) 3.37
P (t) 3.30
9.90 9.03 8.91 8.91 8.82 8.81 8.80 8.80 8.50 8.49 8.48 8.00 7.99 7.98 7.97 7.80 7.80 7.78 7.78 7.78 7.58 7.56 7.55 7.54 7.54 7.38 7.37 7.27 7.27 7.18 7.18 7.16 7.16 7.05 7.03 6.10 4.23 4.21 4.20 3.37 3.32 3.30
1.93 1.00 0.98 1.01 0.98 1.01 1.98 1.98 1.01 1.00 1.00 1.00 1.99 1.95 1.92 2.03
f1 (ppm)

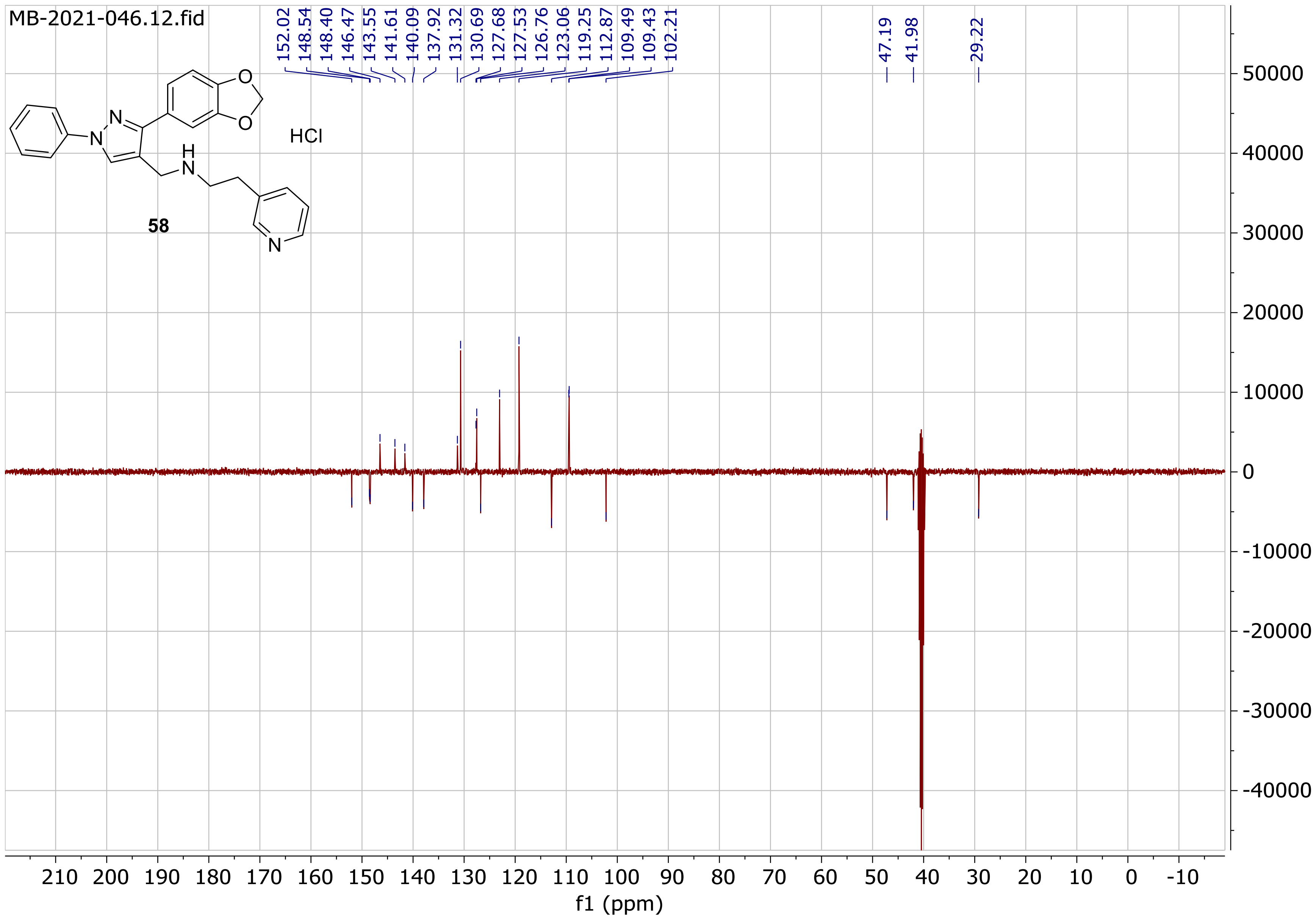
MB-2021-046.12.fid
152.02
148.54
148.40
146.47
143.55
141.61
140.09
137.92
131.32
130.69
127.68
127.53
126.76
123.06
119.25
112.87
109.49
109.43
102.21
47.19
41.98
29.22
HCl
58
50000
40000
30000
20000
10000
0
-10000
-20000
-30000
-40000
210 200 190 180 170 160 150 140 130 120 110 100 90 80 70 60 50 40 30 20 10 0 -10
f1 (ppm)

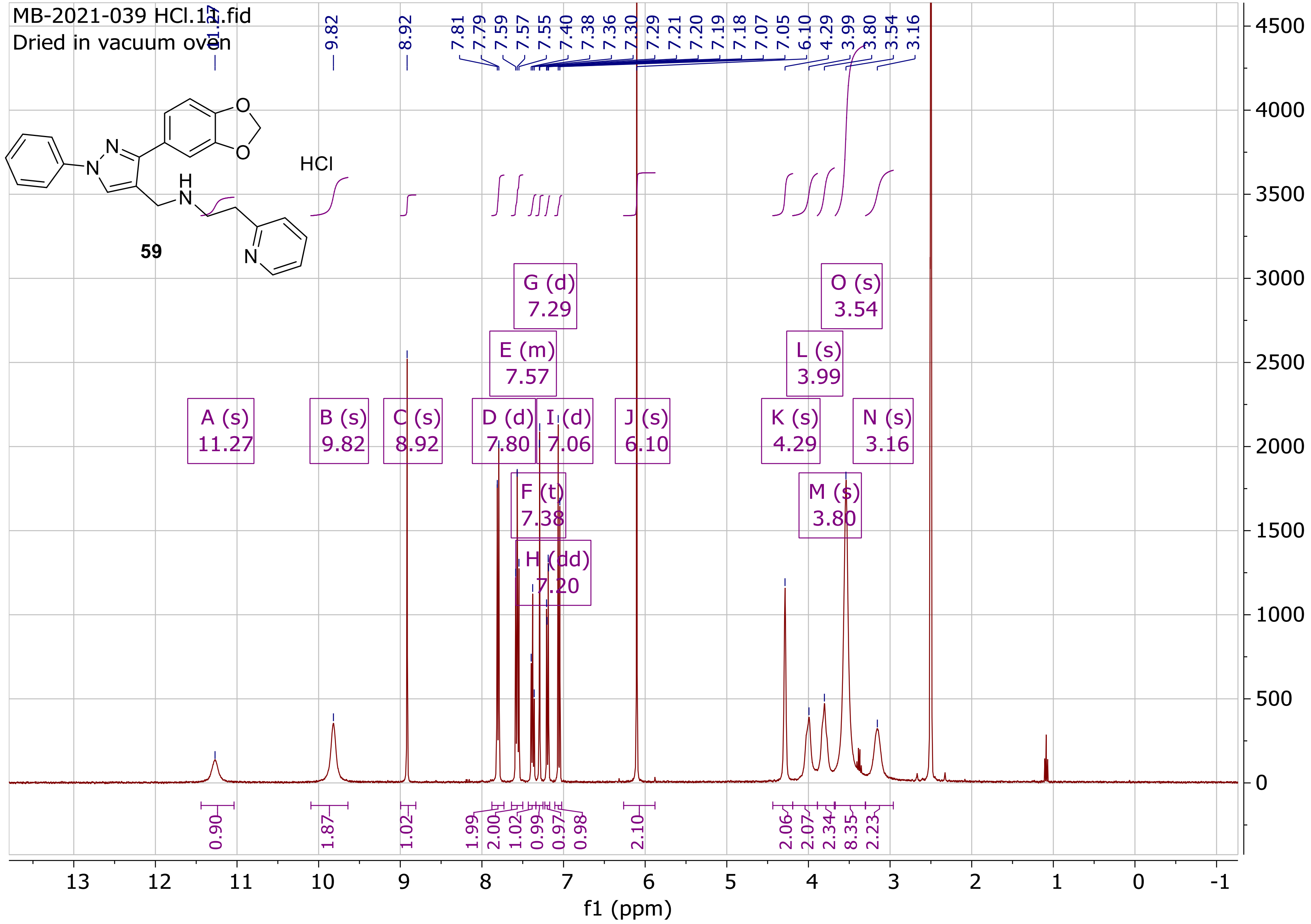
MB-2021-039 HCl.11.fid
Dried in vacuum oven
HCl
59
A (s) 11.27
B (s) 9.82
C (s) 8.92
D (d) 7.80
E (m) 7.57
F (t) 7.38
G (d) 7.29
H (dd) 7.20
I (d) 7.06
J (s) 6.10
K (s) 4.29
L (s) 3.99
M (s) 3.80
N (s) 3.16
O (s) 3.54
0.90
1.87
1.02
1.99
2.00
1.02
0.99
0.97
0.98
2.10
2.06
2.07
2.34
8.35
2.23
f1 (ppm)

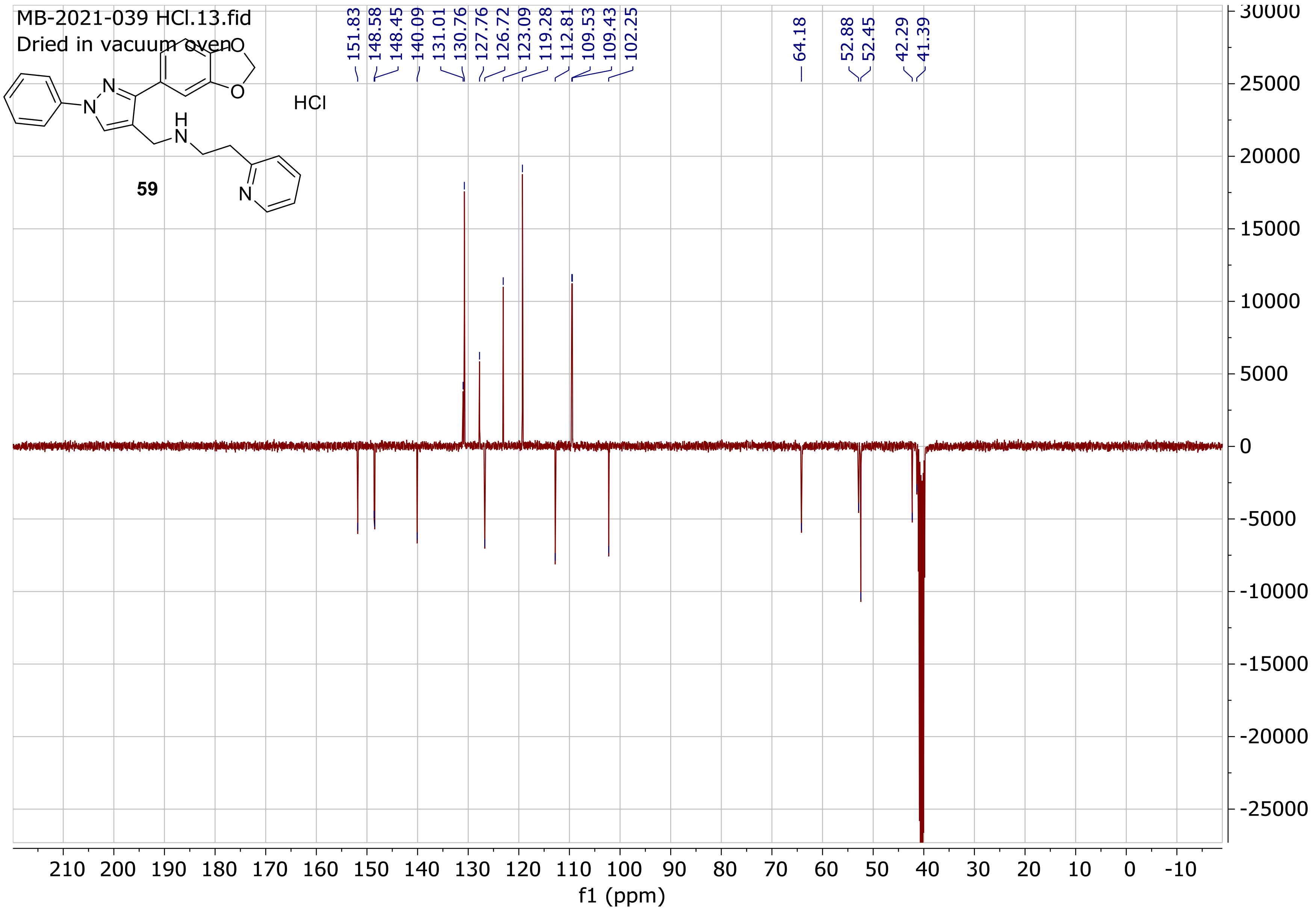
MB-2021-039 HCl.13.fid
Dried in vacuum oven
HCl
59
151.83
148.58
148.45
140.09
131.01
130.76
127.76
126.72
123.09
119.28
112.81
109.53
109.43
102.25
64.18
52.88
52.45
42.29
41.39
f1 (ppm)

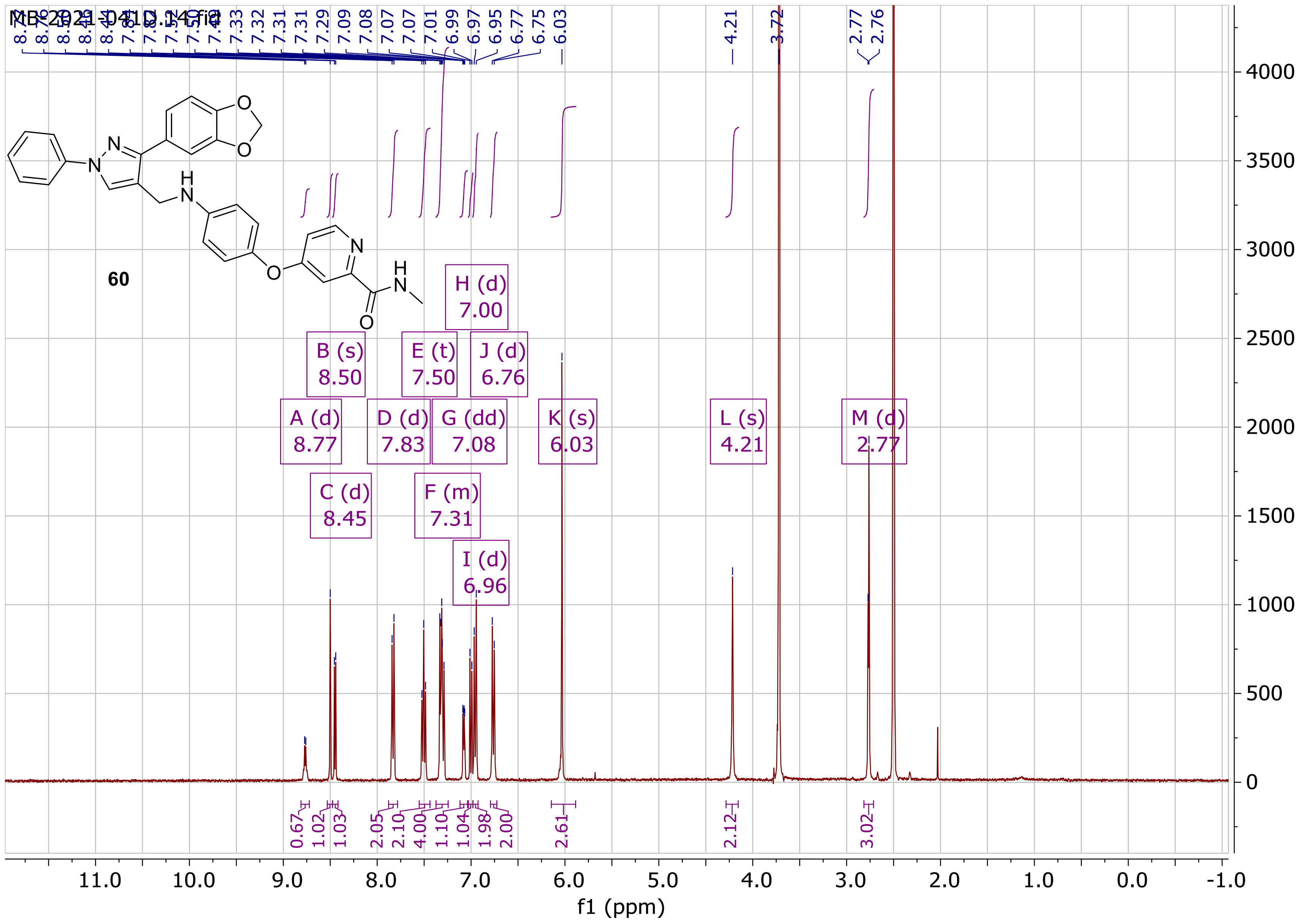
MB-2021-041D.14.fid
60
H (d) 7.00
B (s) 8.50
E (t) 7.50
J (d) 6.76
A (d) 8.77
D (d) 7.83
G (dd) 7.08
K (s) 6.03
L (s) 4.21
M (d) 2.77
C (d) 8.45
F (m) 7.31
I (d) 6.96
0.67 1.02 1.03 2.05 2.10 4.00 1.10 1.04 1.98 2.00 2.61 2.12 3.02
f1 (ppm)

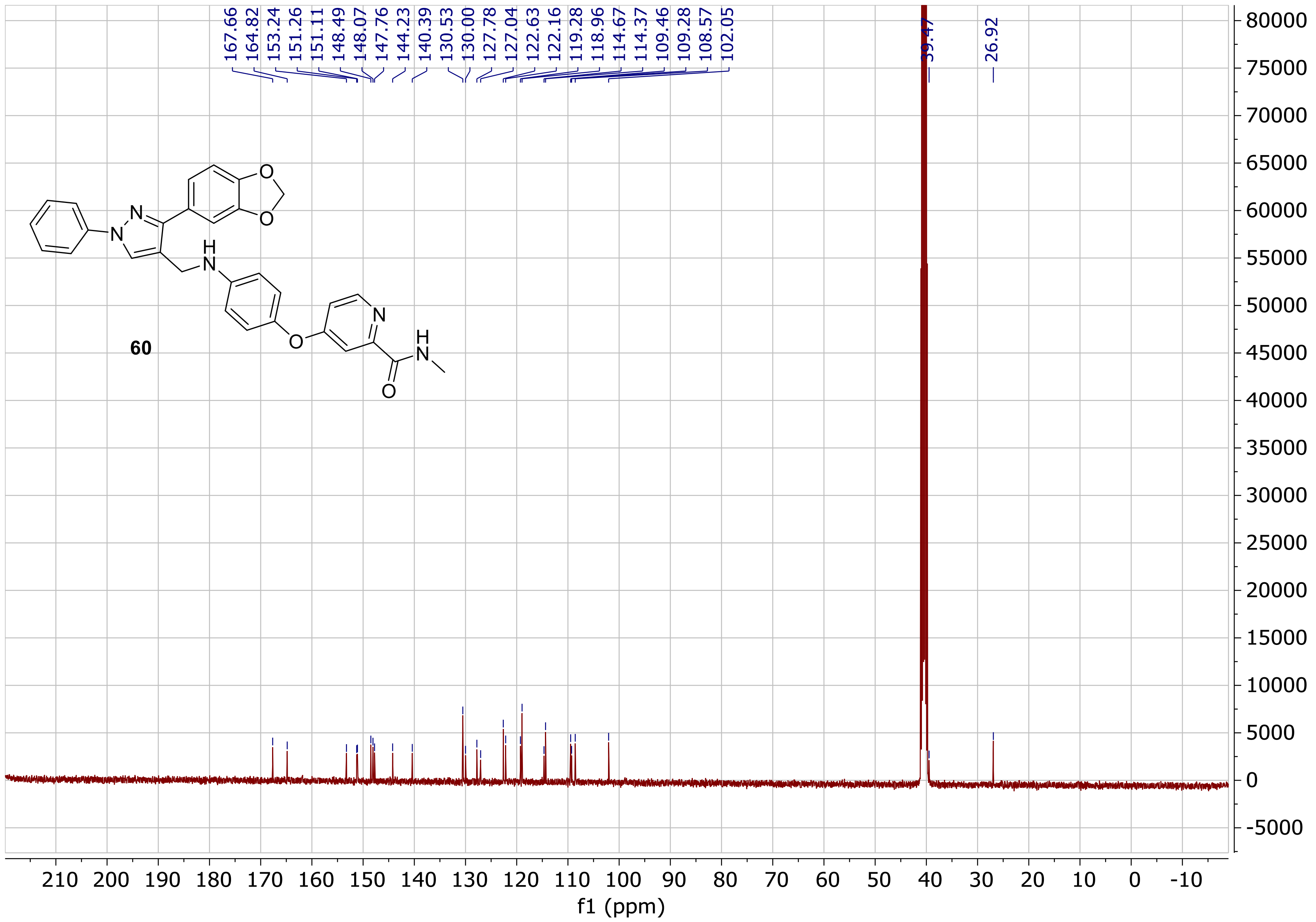

167.66
164.82
153.24
151.26
151.11
148.49
148.07
147.76
144.23
140.39
130.53
130.00
127.78
127.04
122.63
122.16
119.28
118.96
114.67
114.37
109.46
109.28
108.57
102.05
39.47
26.92
60
f1 (ppm)

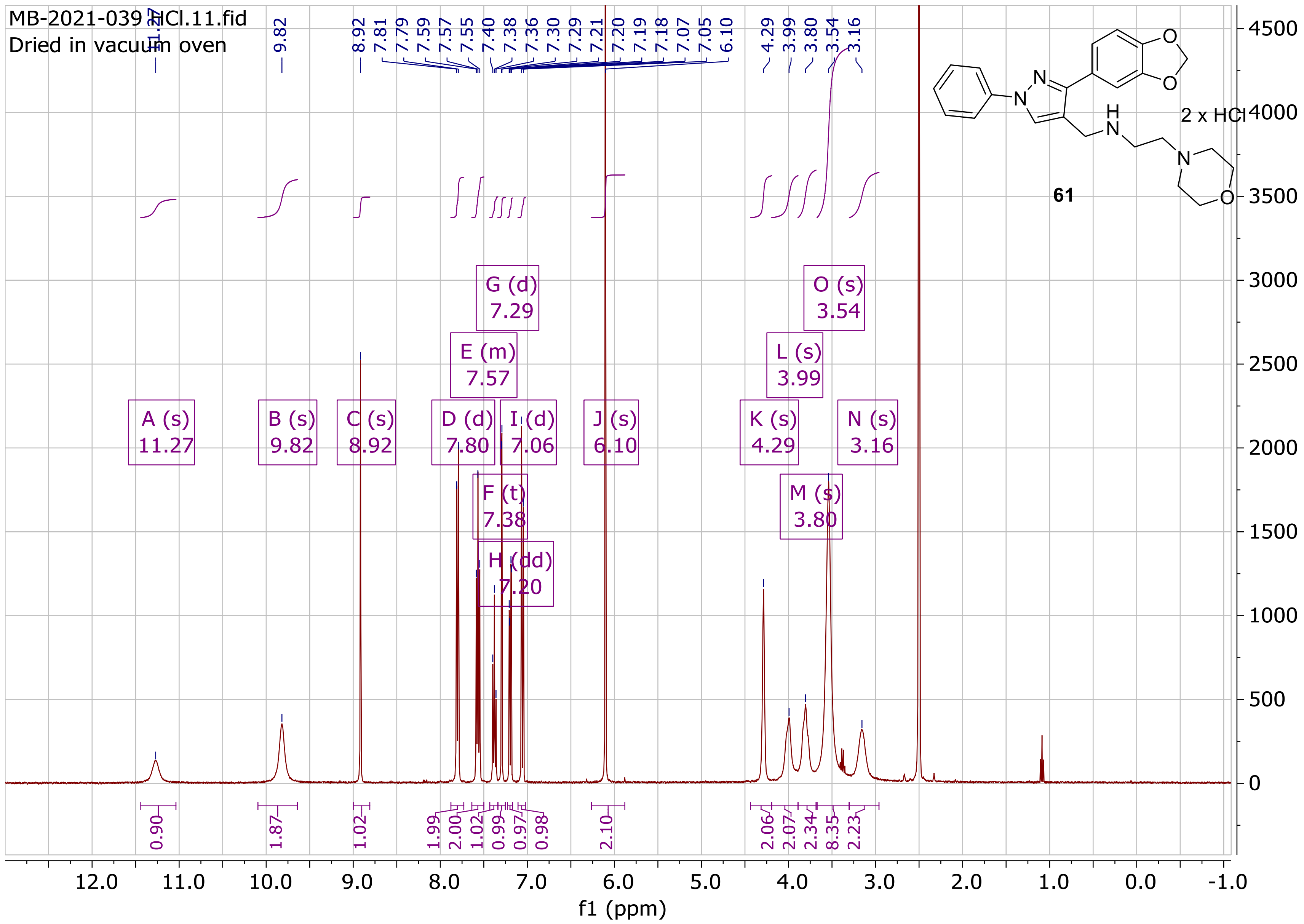
MB-2021-039 HCl.11.fid
Dried in vacuum oven
2 x HCl
61
A (s) 11.27
B (s) 9.82
C (s) 8.92
D (d) 7.80
E (m) 7.57
F (t) 7.38
G (d) 7.29
H (dd) 7.20
I (d) 7.06
J (s) 6.10
K (s) 4.29
L (s) 3.99
M (s) 3.80
N (s) 3.16
O (s) 3.54
f1 (ppm)

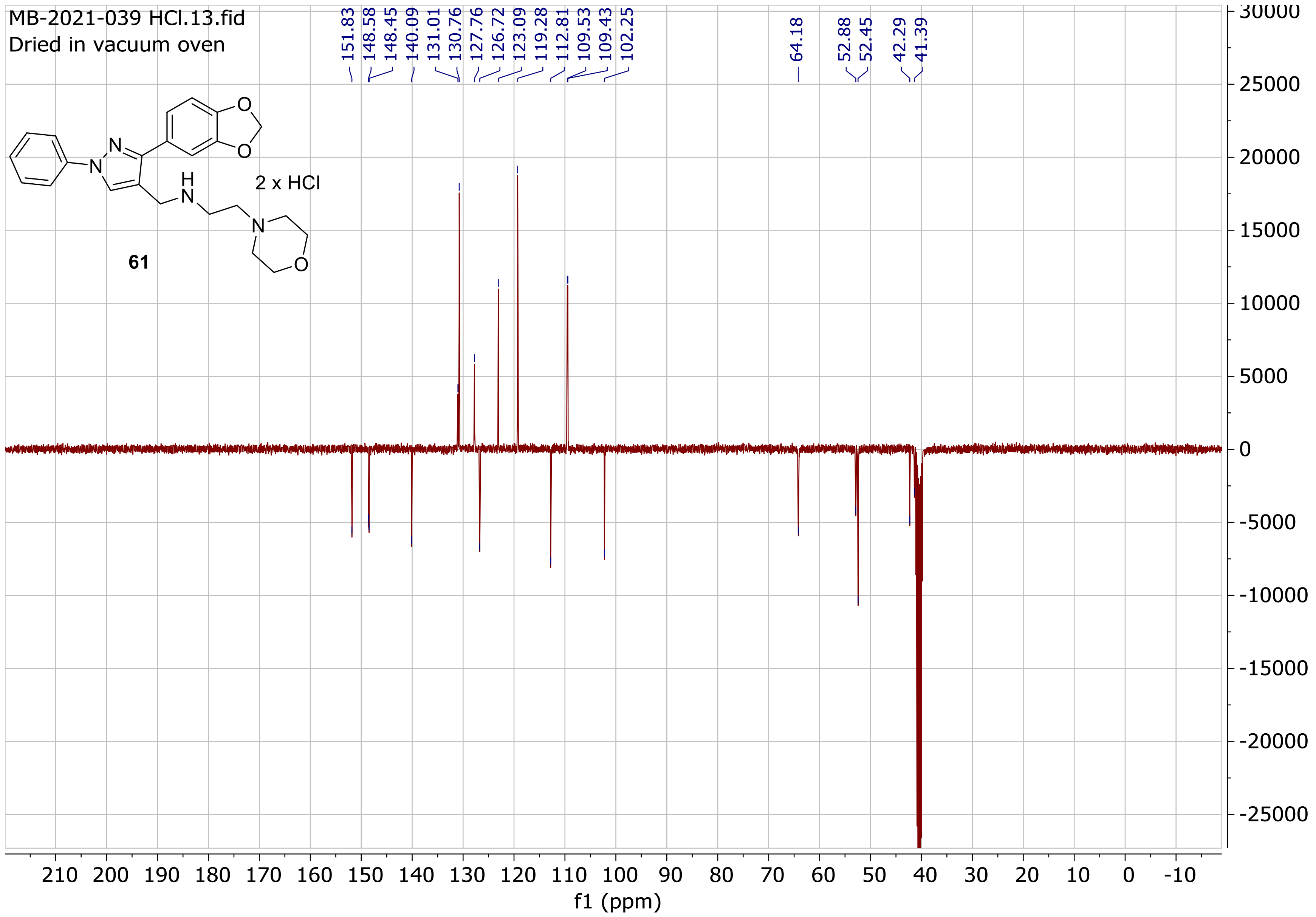
MB-2021-039 HCl.13.fid
Dried in vacuum oven
151.83
148.58
148.45
140.09
131.01
130.76
127.76
126.72
123.09
119.28
112.81
109.53
109.43
102.25
64.18
52.88
52.45
42.29
41.39
2 x HCl
61
f1 (ppm)

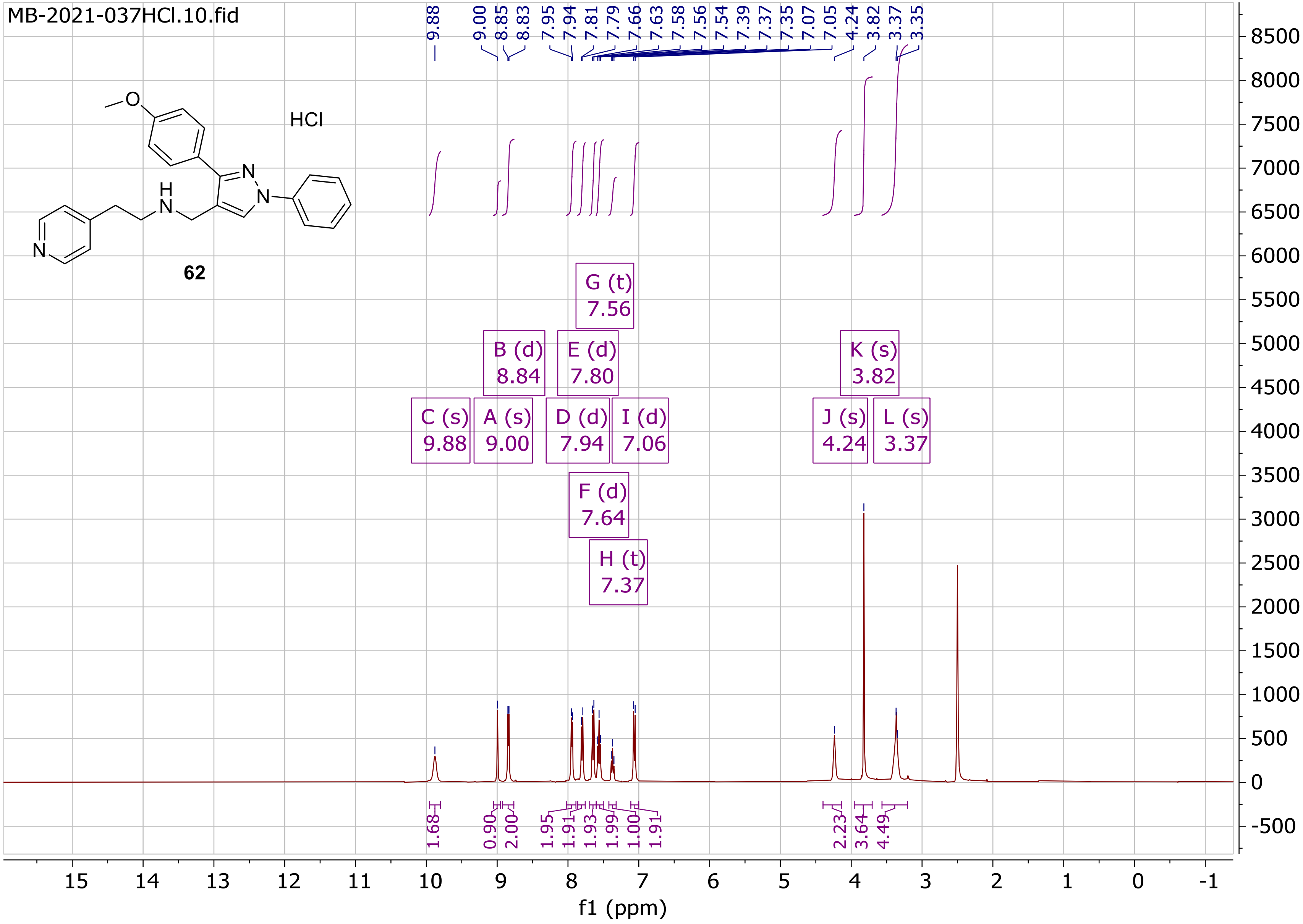
MB-2021-037HCl.10.fid
9.88
9.00
8.85
8.83
7.95
7.94
7.81
7.79
7.66
7.63
7.58
7.56
7.54
7.39
7.37
7.35
7.07
7.05
4.24
3.82
3.37
3.35
HCl
62
G (t) 7.56
B (d) 8.84
E (d) 7.80
K (s) 3.82
C (s) 9.88
A (s) 9.00
D (d) 7.94
I (d) 7.06
J (s) 4.24
L (s) 3.37
F (d) 7.64
H (t) 7.37
1.68
0.90
2.00
1.95
1.91
1.93
1.99
1.00
1.91
2.23
3.64
4.49
f1 (ppm)

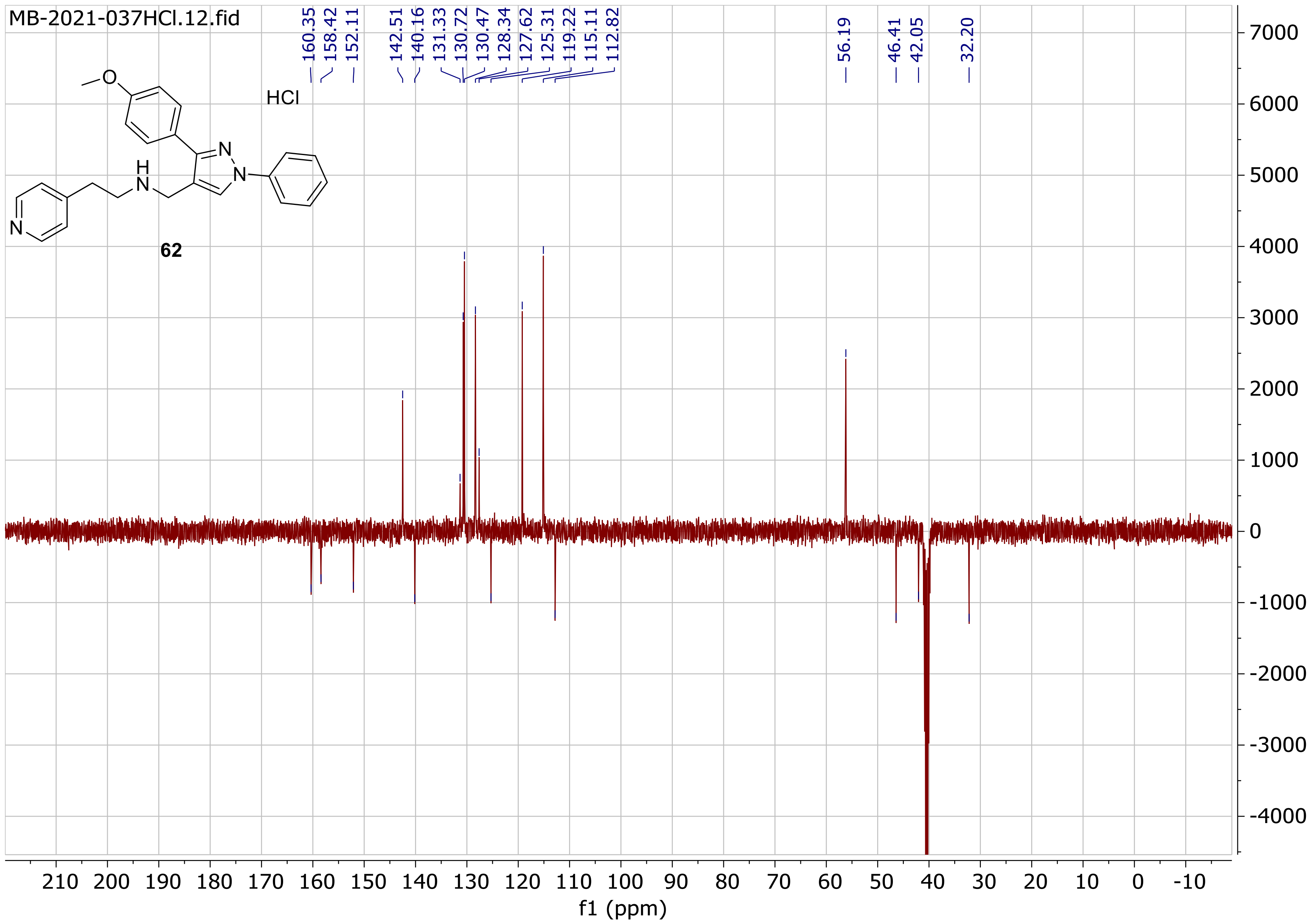

MB-2021-037HCl.12.fid
160.35
158.42
152.11
142.51
140.16
131.33
130.72
130.47
128.34
127.62
125.31
119.22
115.11
112.82
56.19
46.41
42.05
32.20
HCl
62
7000
6000
5000
4000
3000
2000
1000
0
-1000
-2000
-3000
-4000
210 200 190 180 170 160 150 140 130 120 110 100 90 80 70 60 50 40 30 20 10 0 -10
f1 (ppm)

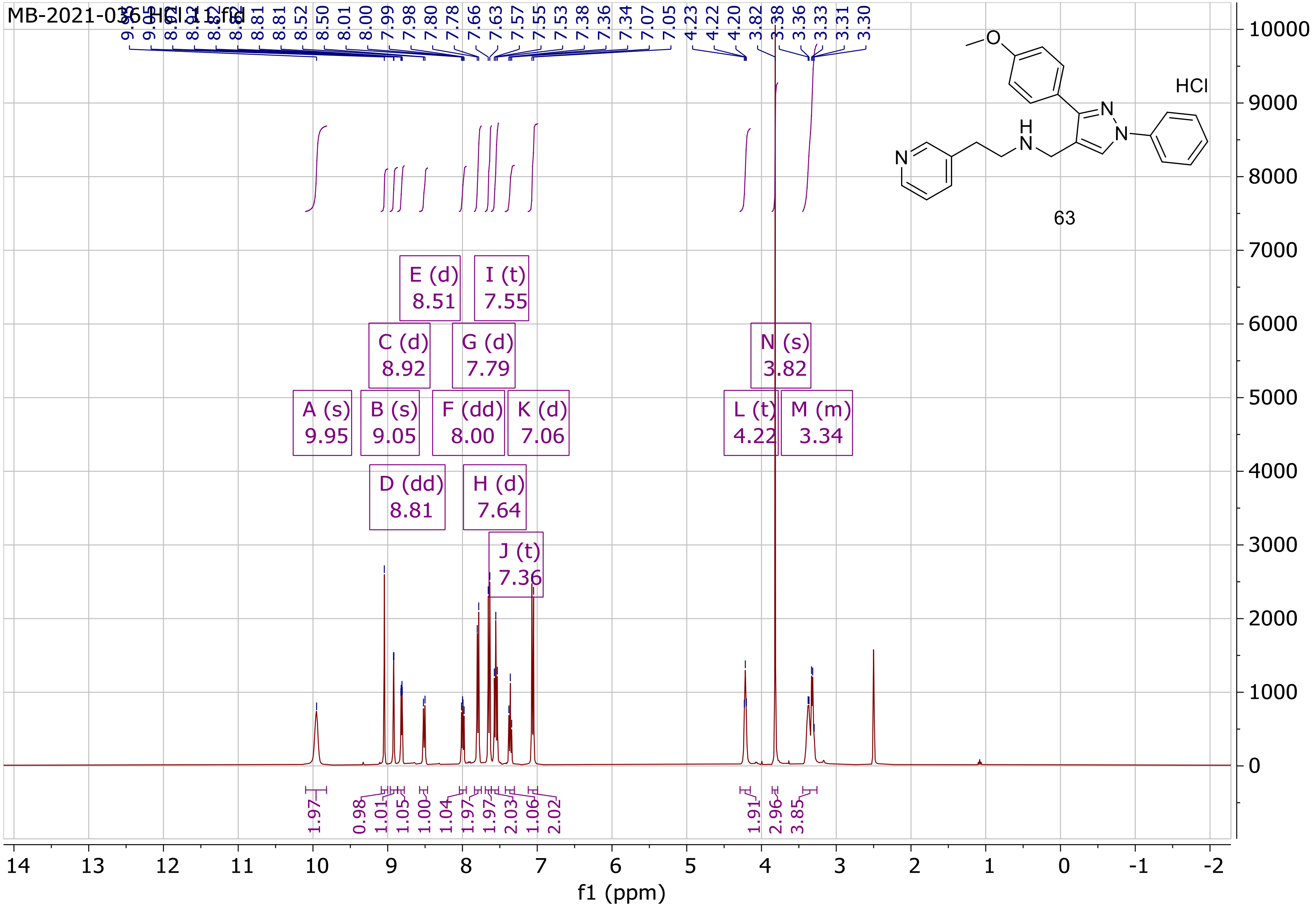
MB-2021-036 HCl.11.fid
8.81
8.81
8.52
8.50
8.01
8.00
7.99
7.98
7.80
7.78
7.66
7.63
7.57
7.55
7.53
7.38
7.36
7.34
7.07
7.05
4.23
4.22
4.20
3.82
3.38
3.36
3.33
3.31
3.30
A (s)
9.95
B (s)
9.05
C (d)
8.92
D (dd)
8.81
E (d)
8.51
F (dd)
8.00
G (d)
7.79
H (d)
7.64
I (t)
7.55
J (t)
7.36
K (d)
7.06
L (t)
4.22
M (m)
3.34
N (s)
3.82
1.97
0.98
1.01
1.05
1.00
1.04
1.97
1.97
2.03
1.06
2.02
1.91
2.96
3.85
HCl
N
H
N
N
N
O
63
f1 (ppm)

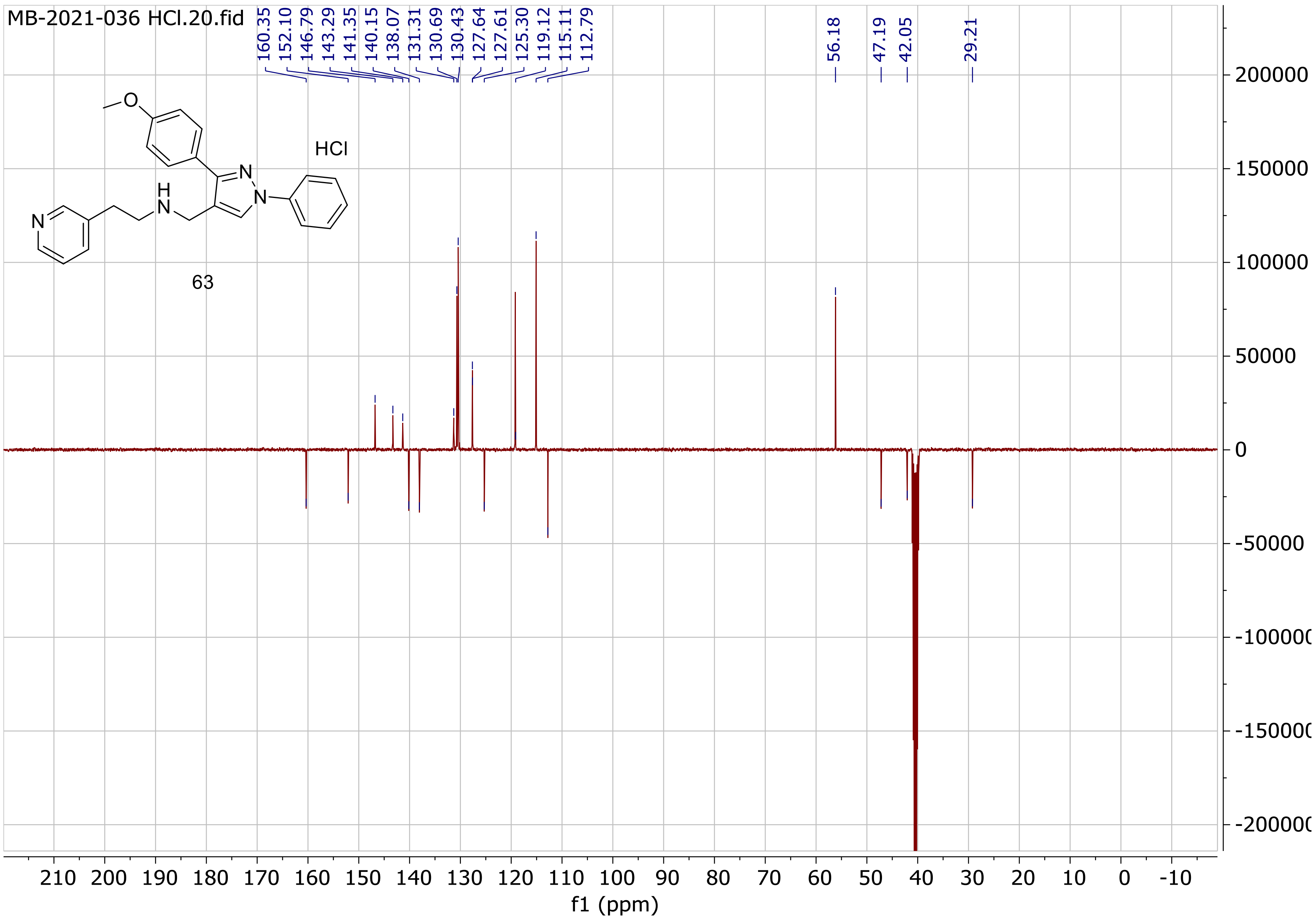
MB-2021-036 HCl.20.fid
160.35
152.10
146.79
143.29
141.35
140.15
138.07
131.31
130.69
130.43
127.64
127.61
125.30
119.12
115.11
112.79
56.18
47.19
42.05
29.21
HCl
63
f1 (ppm)

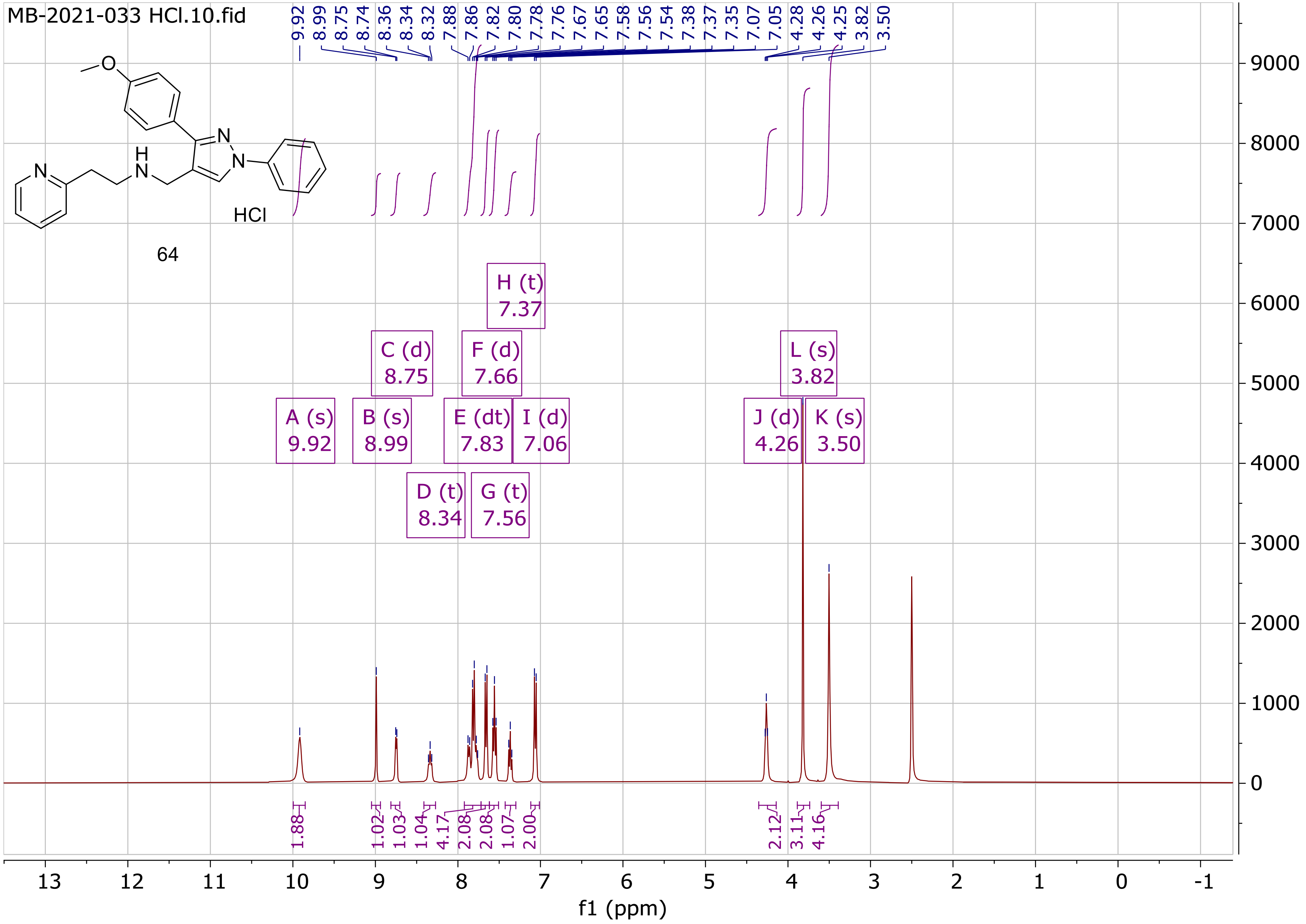

MB-2021-033 HCl.10.fid
9.92
8.99
8.75
8.74
8.36
8.34
8.32
7.88
7.86
7.82
7.80
7.78
7.76
7.67
7.65
7.58
7.56
7.54
7.38
7.37
7.35
7.07
7.05
4.28
4.26
4.25
3.82
3.50
HCl
64
A (s) 9.92
B (s) 8.99
C (d) 8.75
D (t) 8.34
E (dt) 7.83
F (d) 7.66
G (t) 7.56
H (t) 7.37
I (d) 7.06
J (d) 4.26
K (s) 3.50
L (s) 3.82
1.88
1.02
1.03
1.04
4.17
2.08
2.08
1.07
2.00
2.12
3.11
4.16
f1 (ppm)

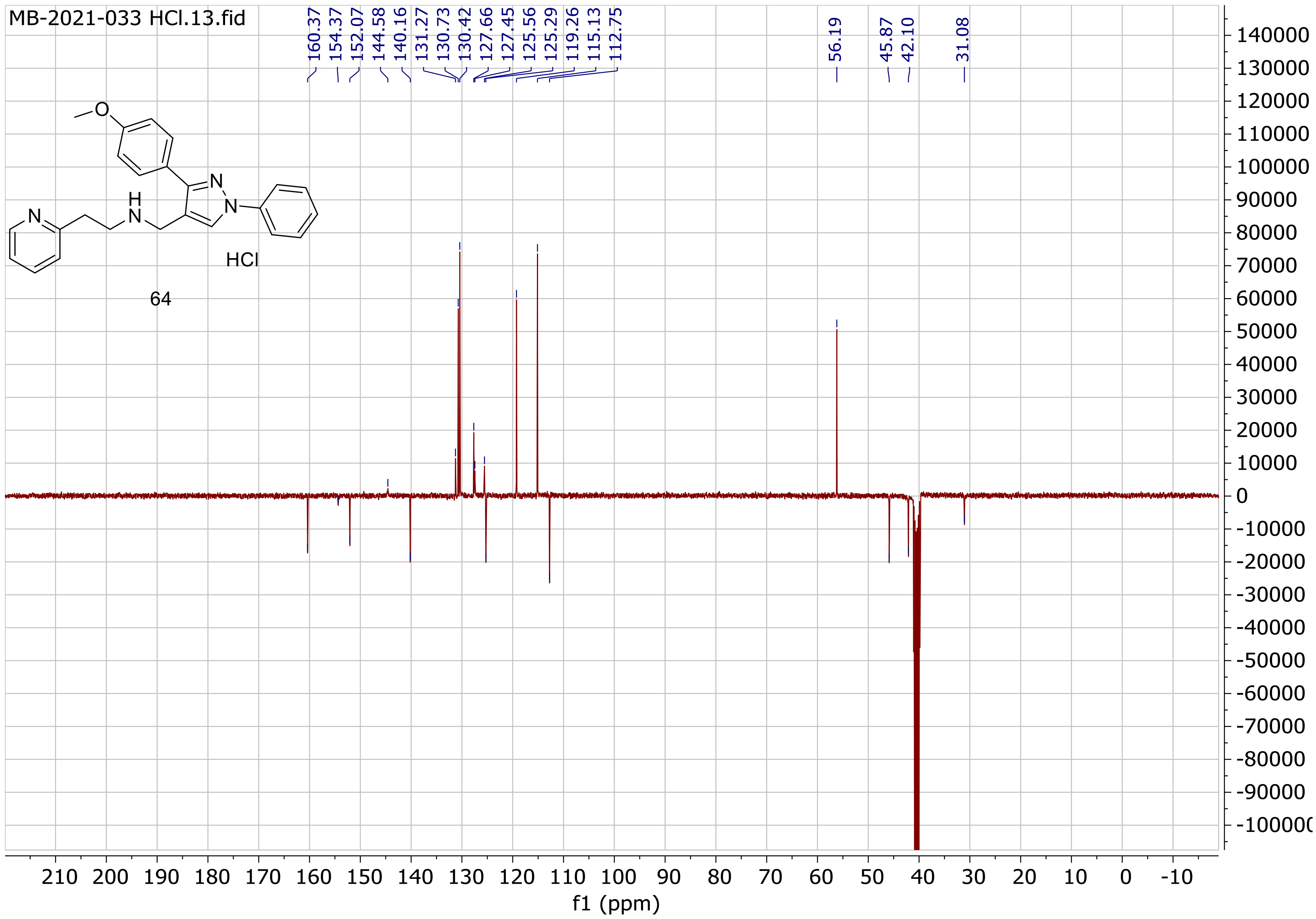
MB-2021-033 HCl.13.fid
160.37
154.37
152.07
144.58
140.16
131.27
130.73
130.42
127.66
127.45
125.56
125.29
119.26
115.13
112.75
56.19
45.87
42.10
31.08
HCl
64
f1 (ppm)

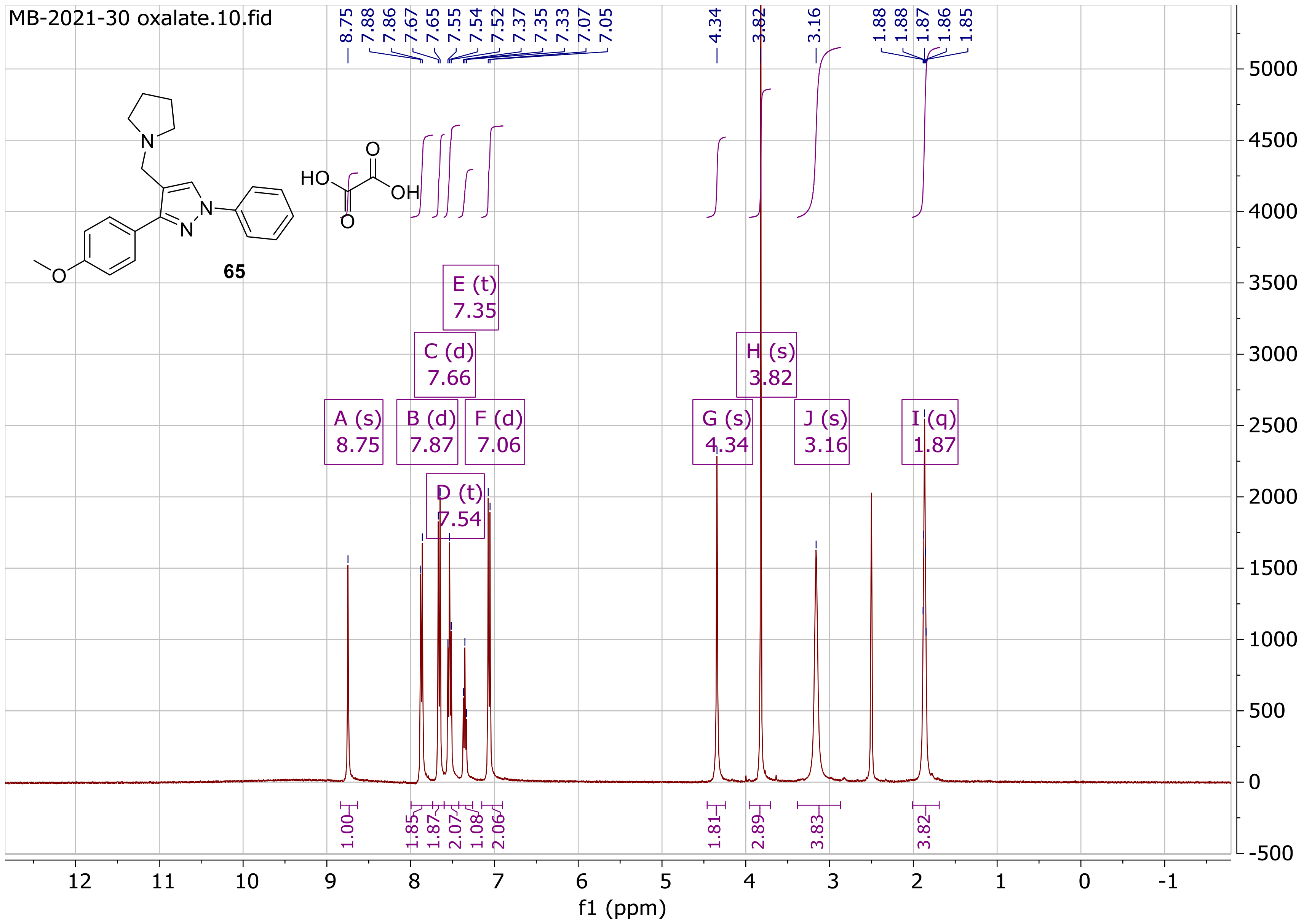

MB-2021-30 oxalate.10.fid
8.75
7.88
7.86
7.67
7.65
7.55
7.54
7.52
7.37
7.35
7.33
7.07
7.05
4.34
3.82
3.16
1.88
1.88
1.87
1.86
1.85
HO
O
OH
O
N
N
N
O
65
A (s) 8.75
B (d) 7.87
C (d) 7.66
D (t) 7.54
E (t) 7.35
F (d) 7.06
G (s) 4.34
H (s) 3.82
J (s) 3.16
I (q) 1.87
1.00
1.85
1.87
2.07
1.08
2.06
1.81
2.89
3.83
3.82
12
11
10
9
8
7
6
5
4
3
2
1
0
-1
f1 (ppm)
5000
4500
4000
3500
3000
2500
2000
1500
1000
500
0
-500

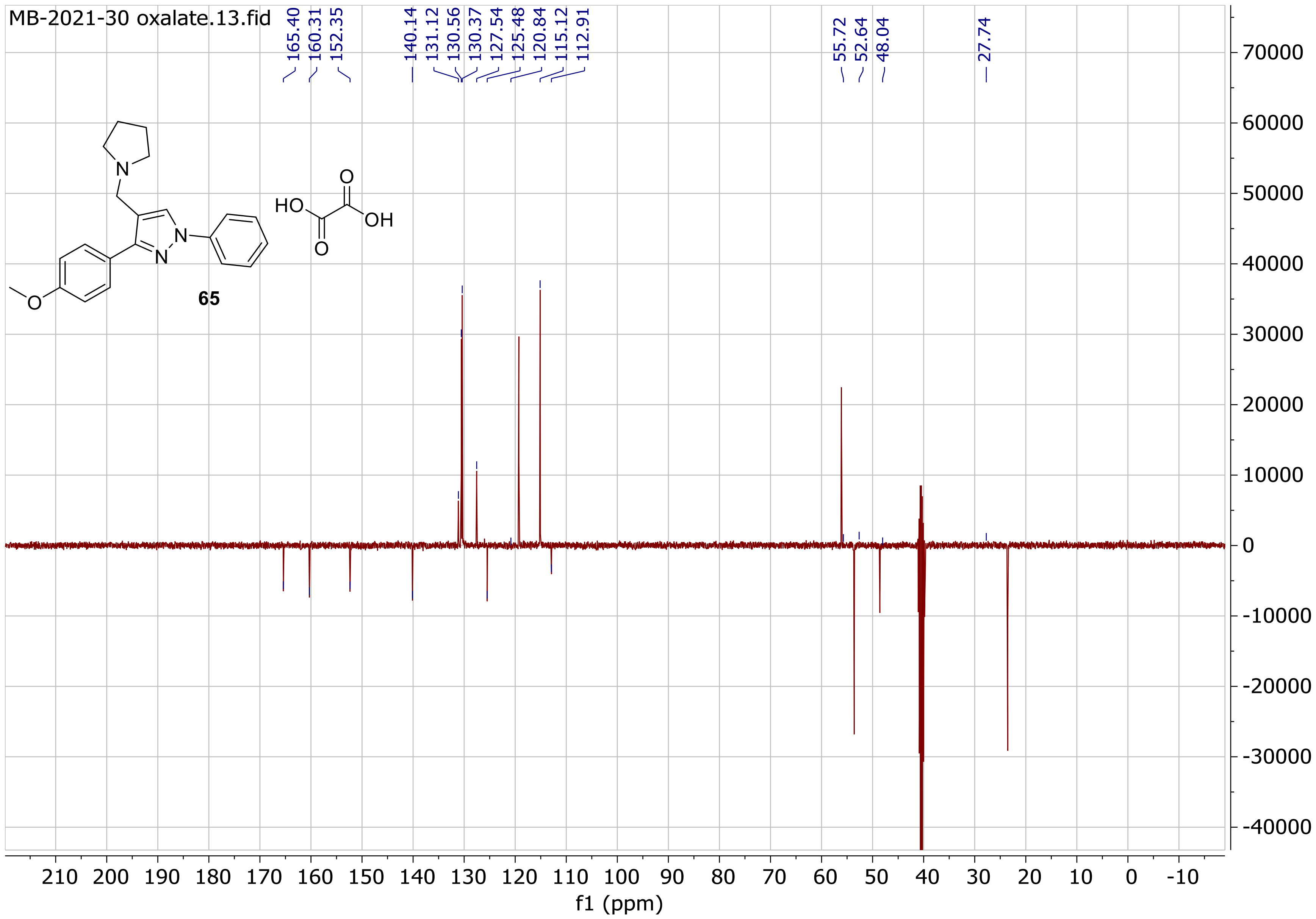
MB-2021-30 oxalate.13.fid
165.40
160.31
152.35
140.14
131.12
130.56
130.37
127.54
125.48
120.84
115.12
112.91
55.72
52.64
48.04
27.74
N
N
N
O
HO
OH
O
O
65
f1 (ppm)

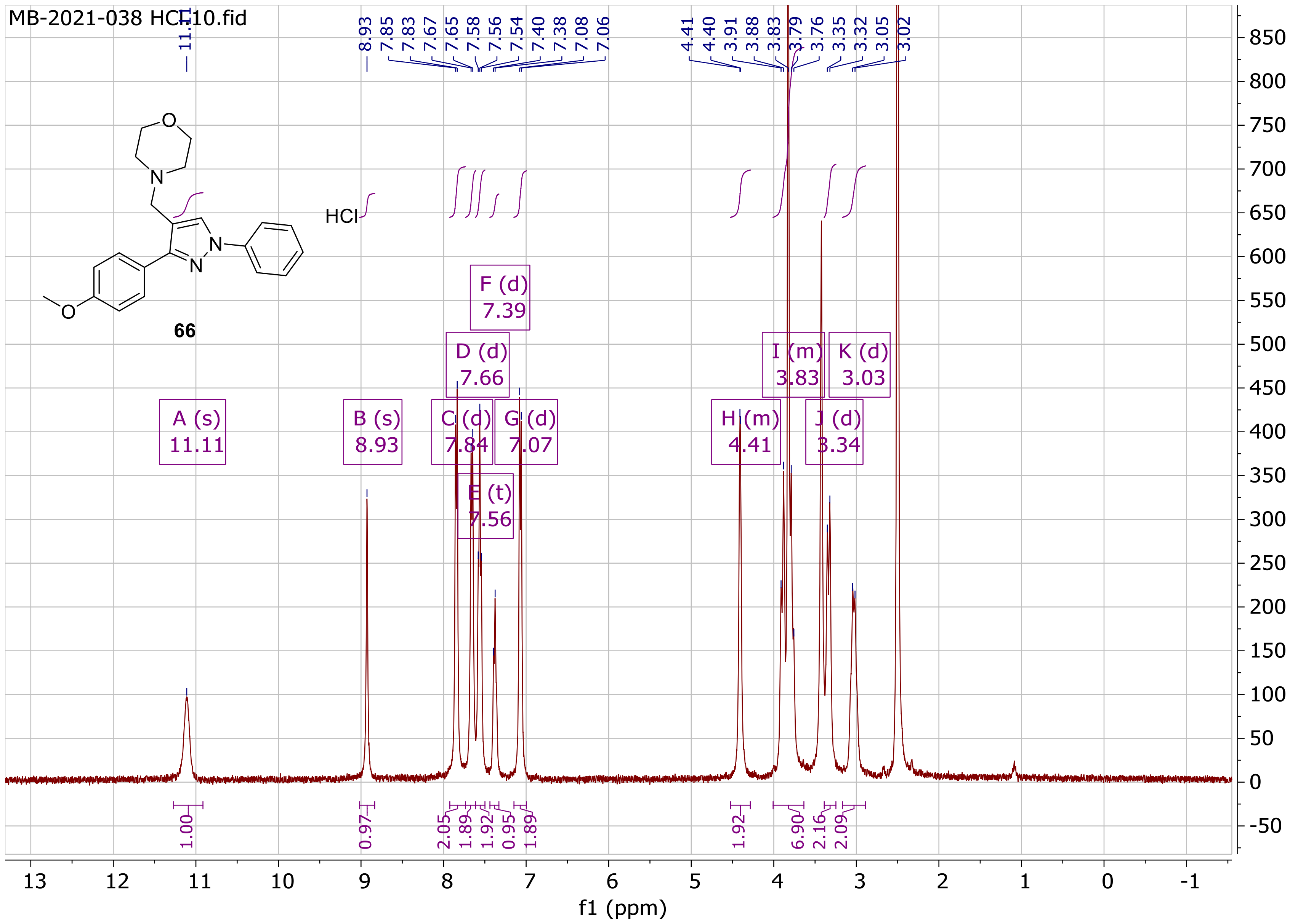

MB-2021-038 HCl 10.fid
11.11
8.93 7.85 7.83 7.67 7.65 7.58 7.56 7.54 7.40 7.38 7.08 7.06
4.41 4.40 3.91 3.88 3.83 3.79 3.76 3.35 3.32 3.05 3.02
HCl
66
A (s) 11.11
B (s) 8.93
C (d) 7.84
D (d) 7.66
E (t) 7.56
F (d) 7.39
G (d) 7.07
H (m) 4.41
I (m) 3.83
J (d) 3.34
K (d) 3.03
1.00
0.97
2.05
1.89
1.92
0.95
1.89
1.92
6.90
2.16
2.09
f1 (ppm)

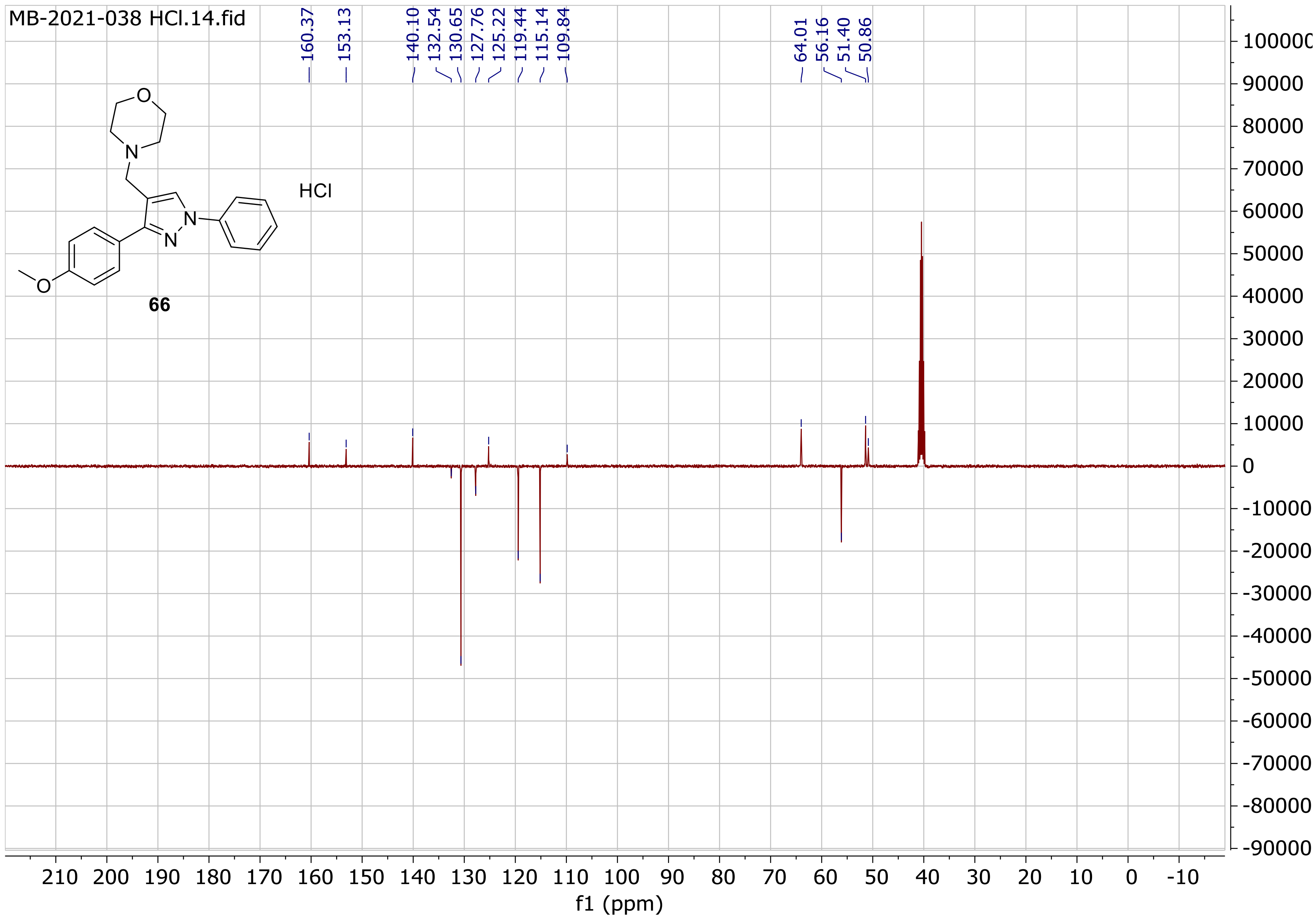

MB-2021-038 HCl.14.fid
160.37
153.13
140.10
132.54
130.65
127.76
125.22
119.44
115.14
109.84
64.01
56.16
51.40
50.86
HCl
66
f1 (ppm)

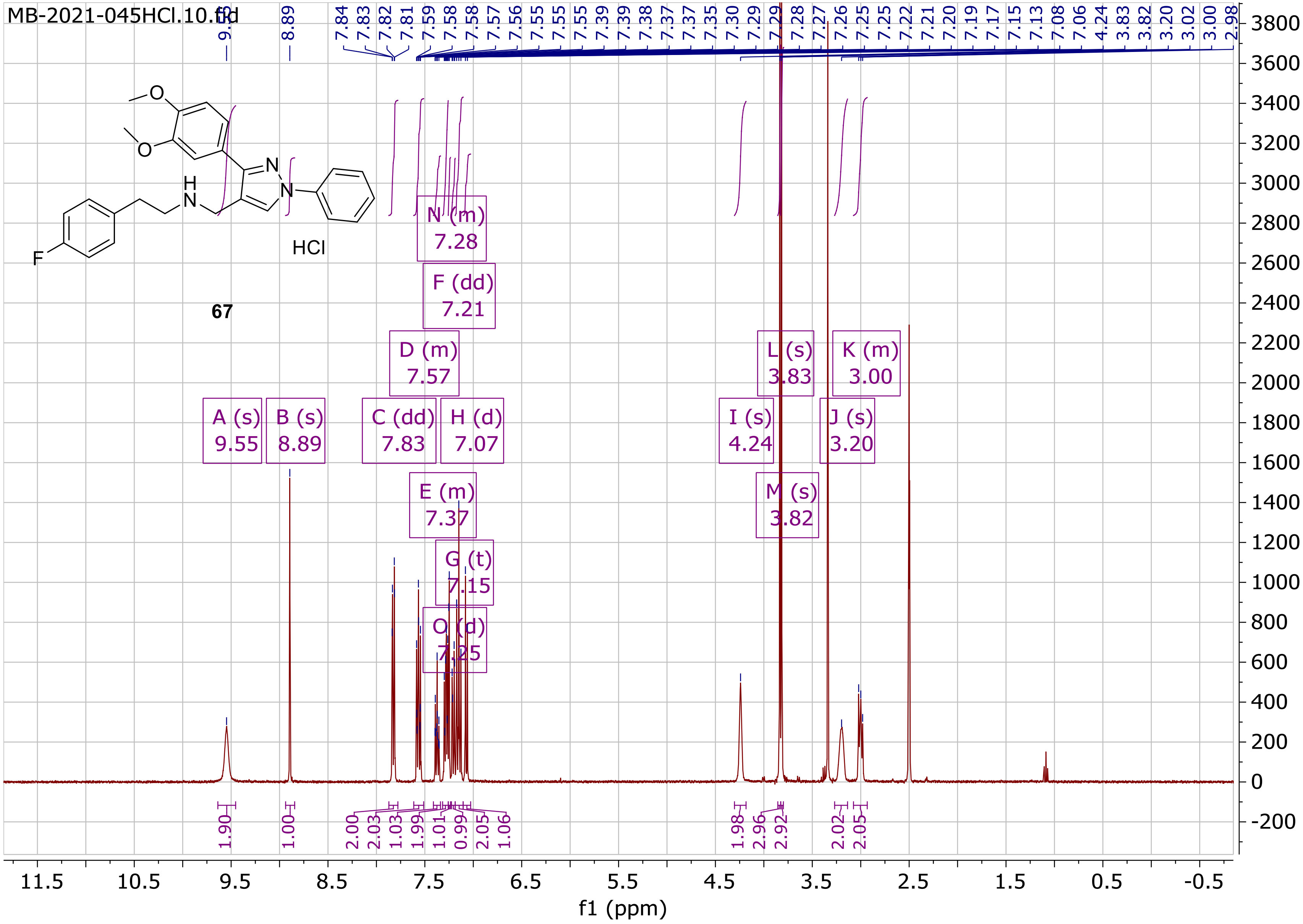

MB-2021-045HCl.10.fid
HCl
67
A (s) 9.55
B (s) 8.89
C (dd) 7.83
D (m) 7.57
E (m) 7.37
F (dd) 7.21
G (t) 7.15
H (d) 7.07
I (s) 4.24
J (s) 3.20
K (m) 3.00
L (s) 3.83
M (s) 3.82
N (m) 7.28
O (d) 7.25
f1 (ppm)

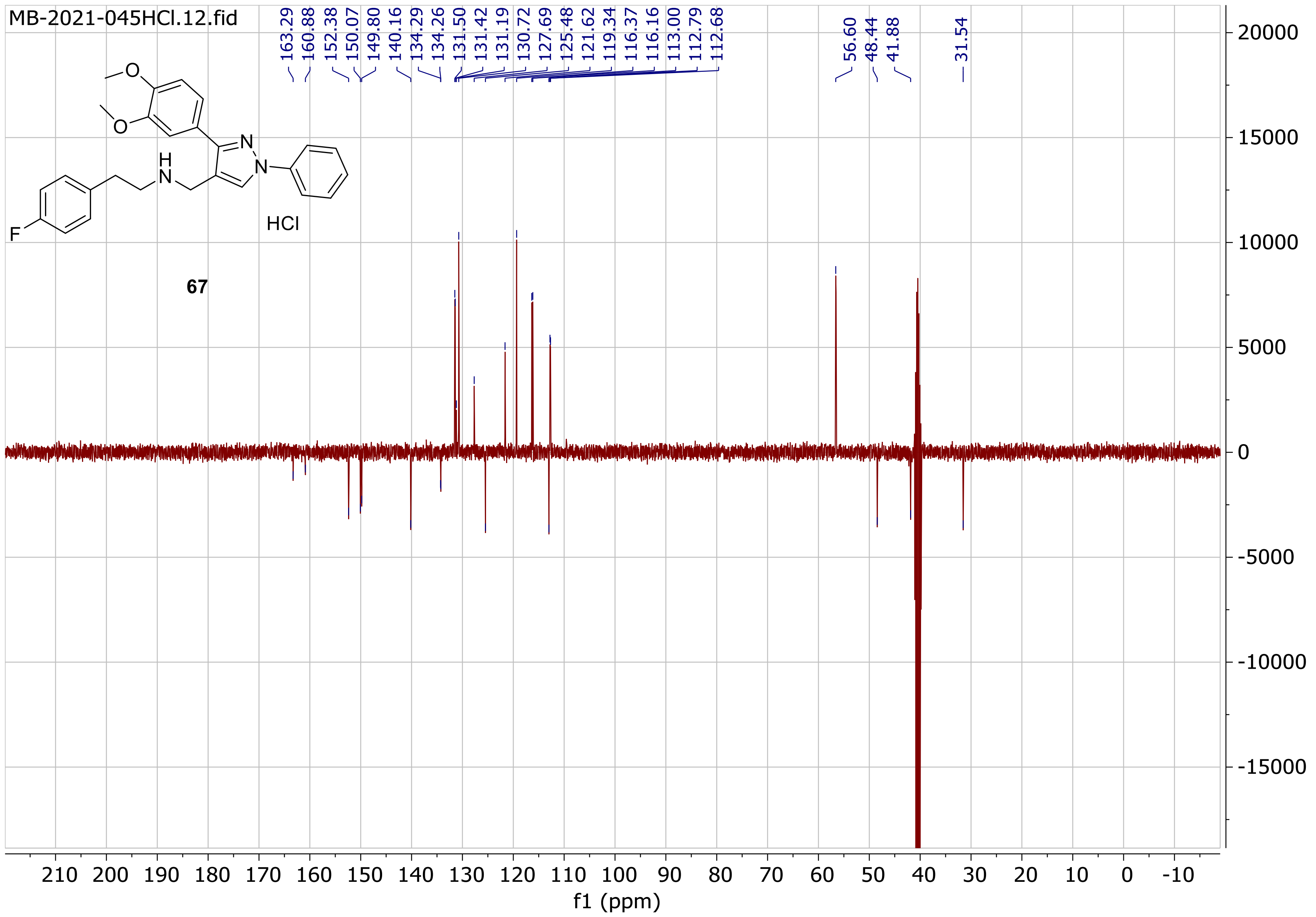
MB-2021-045HCl.12.fid
163.29
160.88
152.38
150.07
149.80
140.16
134.29
134.26
131.50
131.42
131.19
130.72
127.69
125.48
121.62
119.34
116.37
116.16
113.00
112.79
112.68
56.60
48.44
41.88
31.54
HCl
67
f1 (ppm)

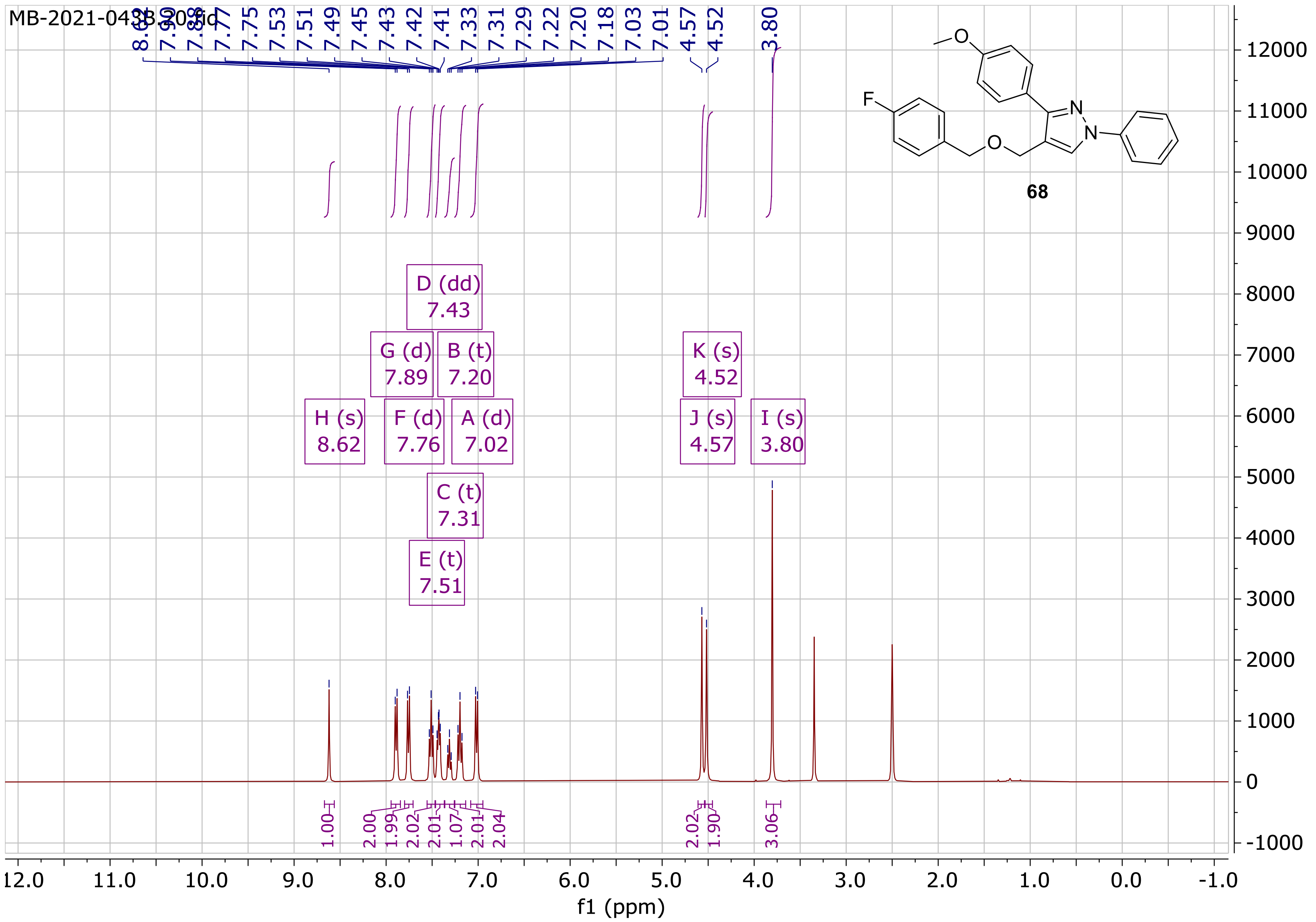

MB-2021-043[illegible]
8.62 7.90 7.88 7.77 7.75 7.53 7.51 7.49 7.45 7.43 7.42 7.41 7.33 7.31 7.29 7.22 7.20 7.18 7.03 7.01 4.57 4.52 3.80
D (dd) 7.43
G (d) 7.89
B (t) 7.20
K (s) 4.52
H (s) 8.62
F (d) 7.76
A (d) 7.02
J (s) 4.57
I (s) 3.80
C (t) 7.31
E (t) 7.51
1.00 2.00 1.99 2.02 2.01 1.07 2.01 2.04 2.02 1.90 3.06
F
O
N
N
O
68
12000 11000 10000 9000 8000 7000 6000 5000 4000 3000 2000 1000 0 -1000
12.0 11.0 10.0 9.0 8.0 7.0 6.0 5.0 4.0 3.0 2.0 1.0 0.0 -1.0
f1 (ppm)

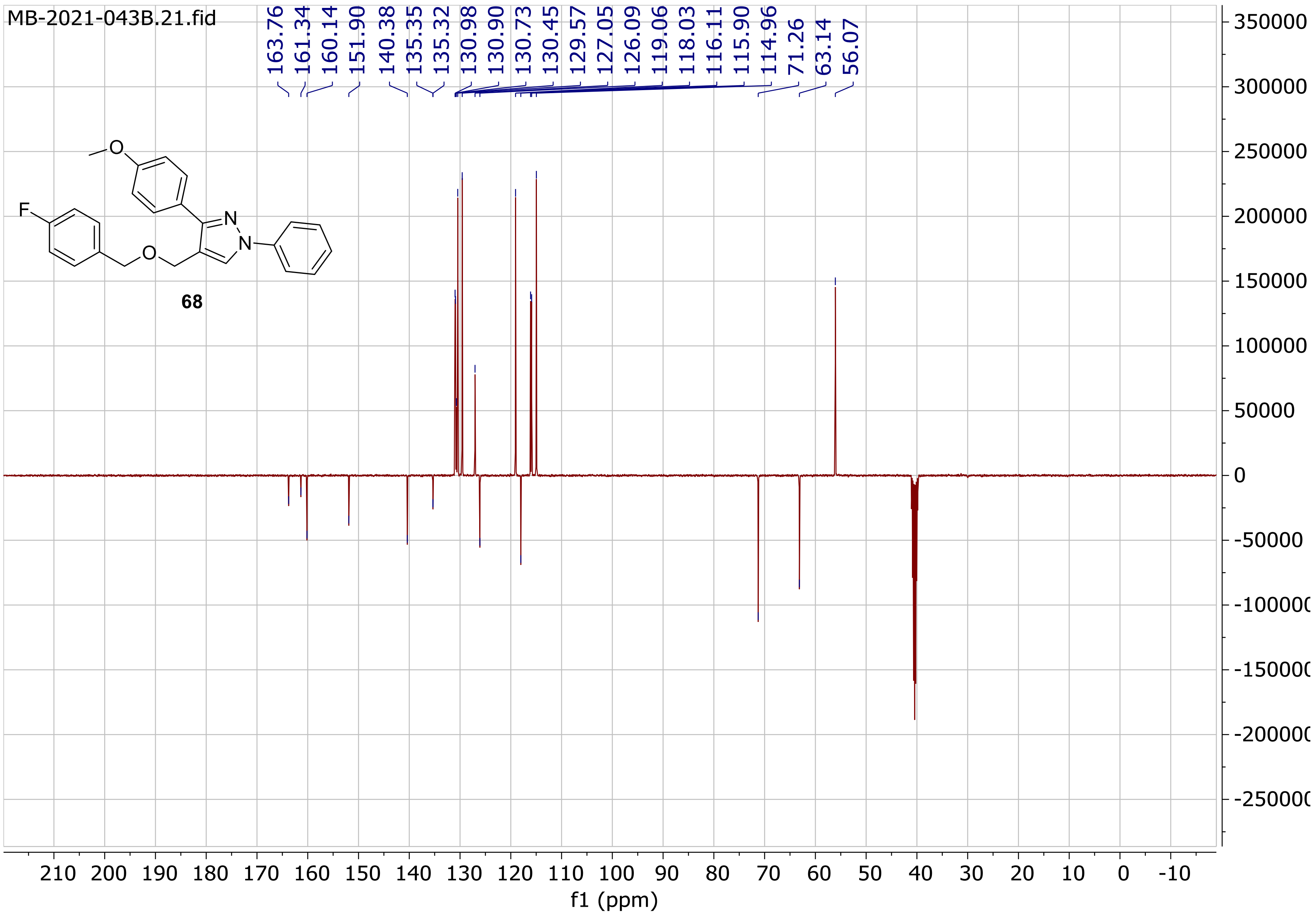

MB-2021-043B.21.fid
163.76
161.34
160.14
151.90
140.38
135.35
135.32
130.98
130.90
130.73
130.45
129.57
127.05
126.09
119.06
118.03
116.11
115.90
114.96
71.26
63.14
56.07
F
O
O
N
N
68
350000
300000
250000
200000
150000
100000
50000
0
-50000
210
200
190
180
170
160
150
140
130
120
110
100
90
80
70
60
50
40
30
20
10
0
-10
f1 (ppm)

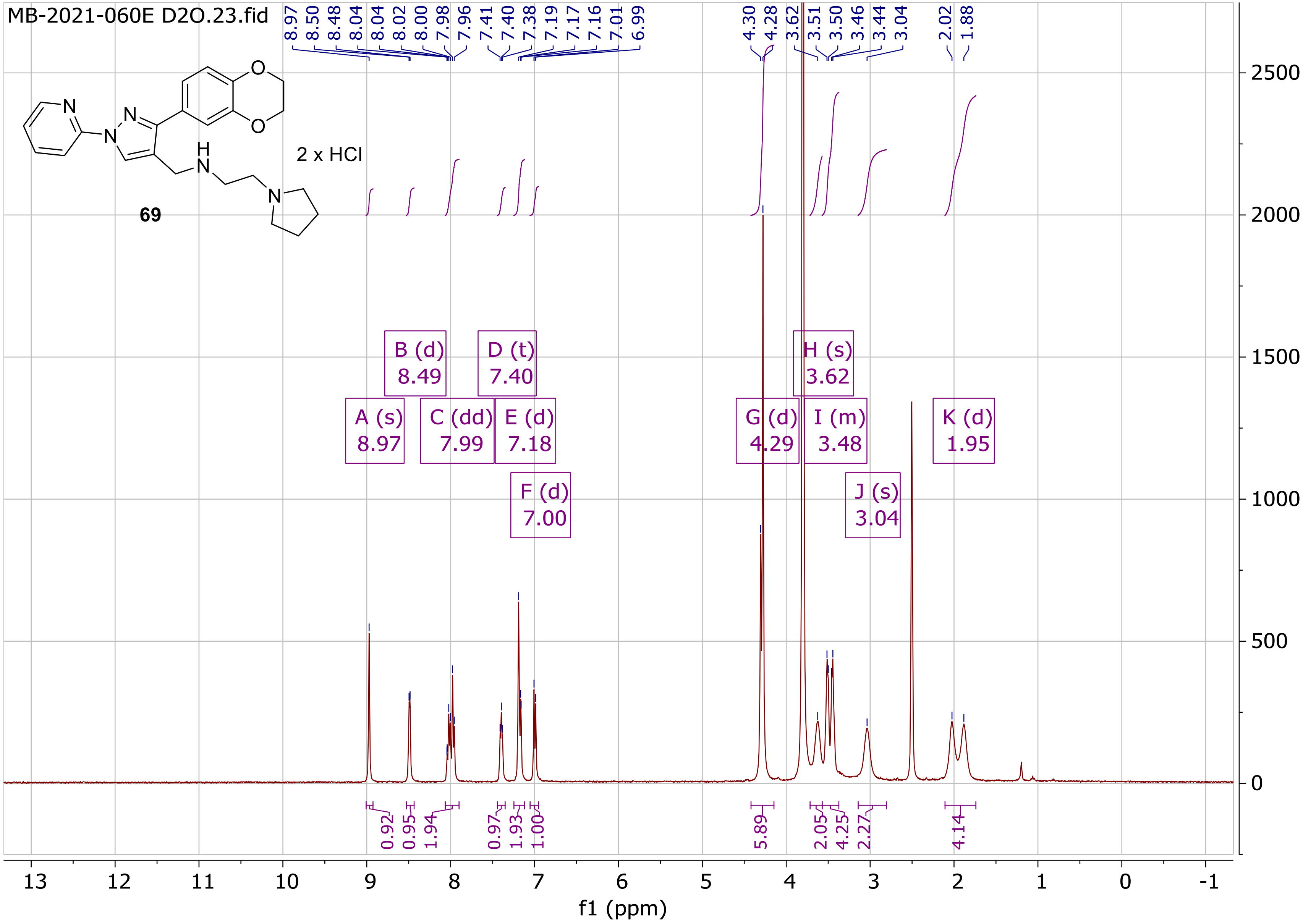

MB-2021-060E D2O.23.fid
2 x HCl
69
8.97 8.50 8.48 8.04 8.04 8.02 8.00 7.98 7.96 7.41 7.40 7.38 7.19 7.17 7.16 7.01 6.99
4.30 4.28 3.62 3.51 3.50 3.46 3.44 3.04 2.02 1.88
A (s) 8.97
B (d) 8.49
C (dd) 7.99
D (t) 7.40
E (d) 7.18
F (d) 7.00
G (d) 4.29
H (s) 3.62
I (m) 3.48
J (s) 3.04
K (d) 1.95
0.92 0.95 1.94 0.97 1.93 1.00 5.89 2.05 4.25 2.27 4.14
f1 (ppm)

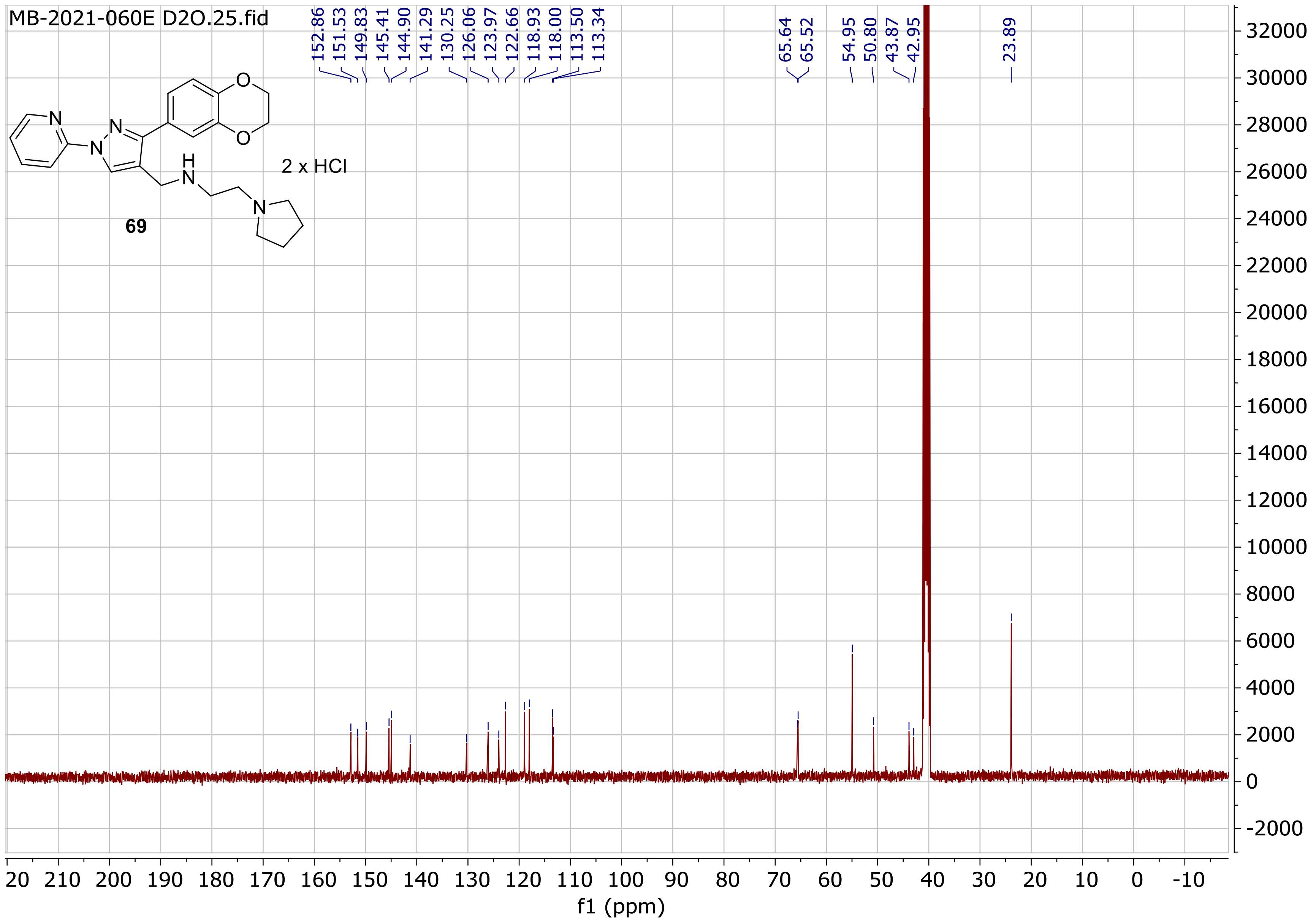
MB-2021-060E D2O.25.fid
152.86
151.53
149.83
145.41
144.90
141.29
130.25
126.06
123.97
122.66
118.93
118.00
113.50
113.34
65.64
65.52
54.95
50.80
43.87
42.95
23.89
2 x HCl
69
f1 (ppm)

2. UPLC and HRMS spectra of the studied samples

2.1 *N-((3-(2,3-dihydrobenzo[b][1,4]dioxin-6-yl)-1-phenyl-1H-pyrazol-4-yl)methyl)aniline* (13)

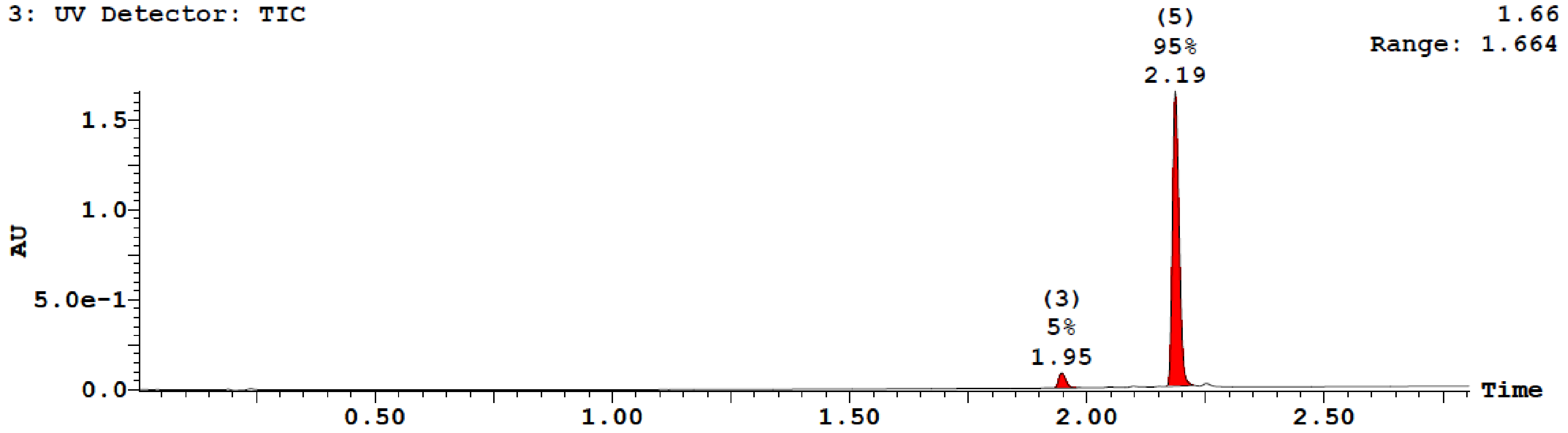

Peak Number	Compound	Time	AreaAbs	Area %Total	Width	Height	Mass Found
3		1.95	1e+003	5.26	0	8e+004	
5		2.19	3e+004	94.74	0	2e+006	

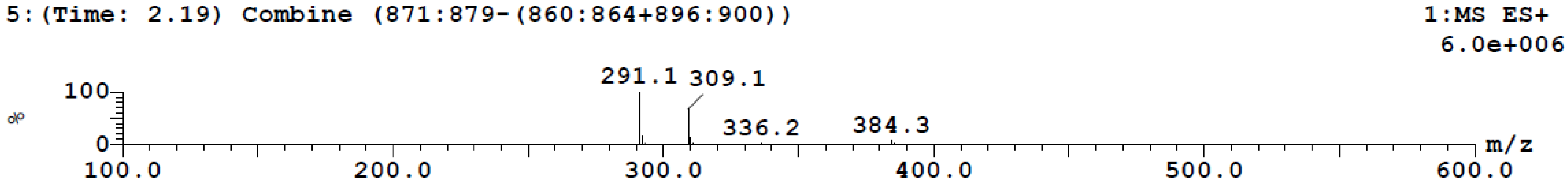

Peak ID	Compound	Time	Mass Found
1		0.18	

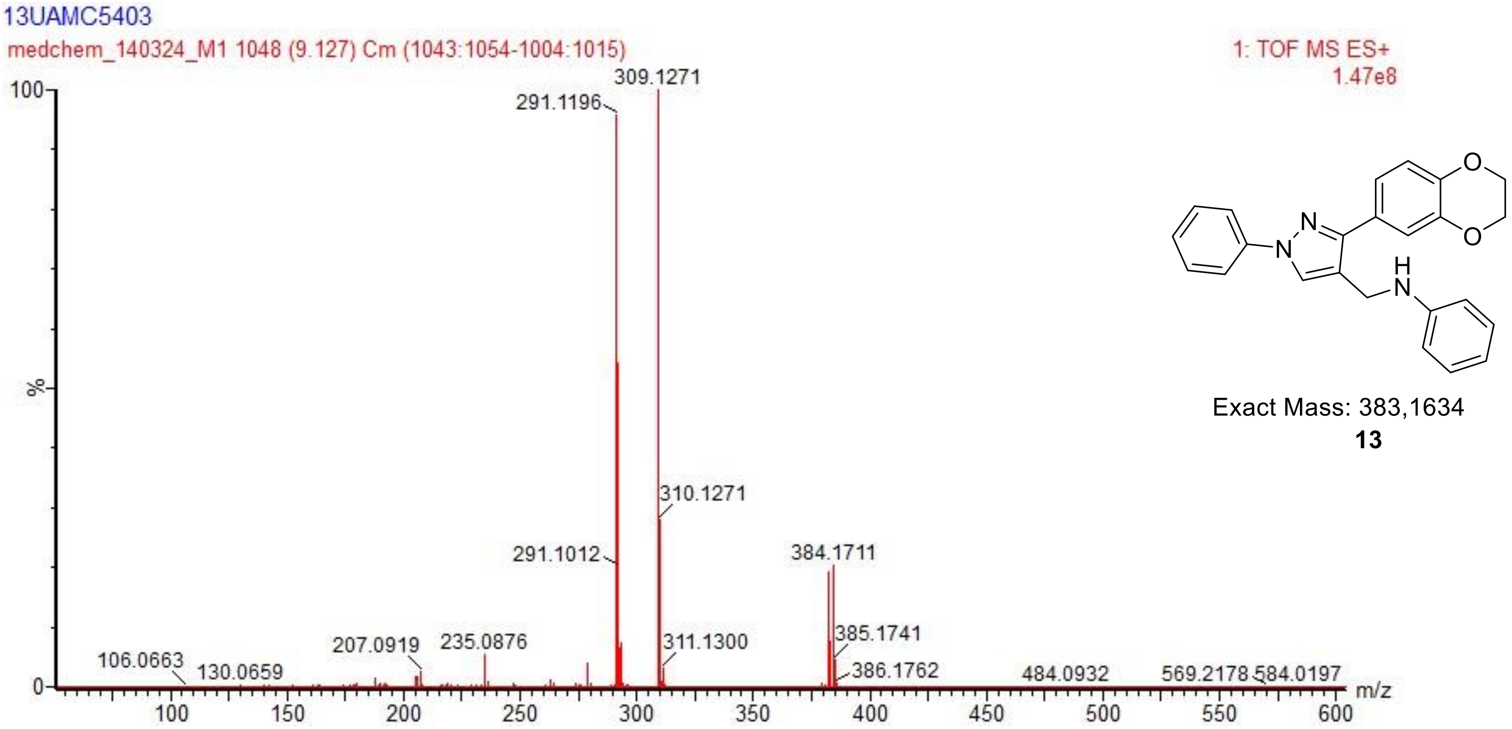

Exact Mass: 383,1634

13

2.2 *N-((3-(2,3-dihydrobenzo[b][1,4]dioxin-6-yl)-1-phenyl-1H-pyrazol-4-yl)methyl)-4-fluoroaniline* (**14**)

Sample 1 Vial 1:57 ID UAMC-5435 File UAMC-5435 Date 13-Apr-2023 Time 17:49:49 Description

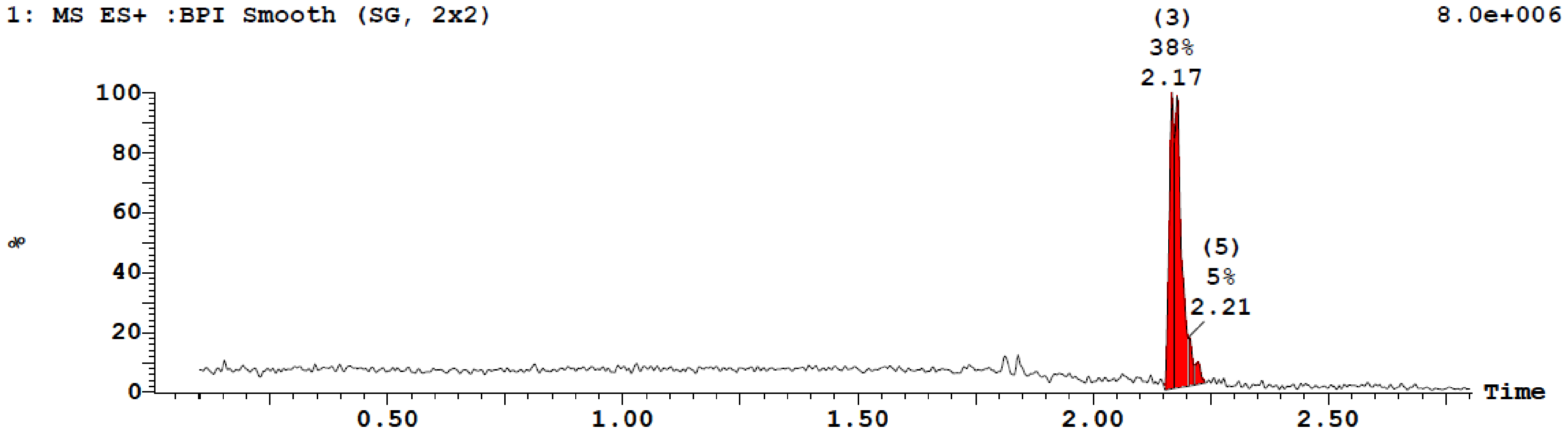

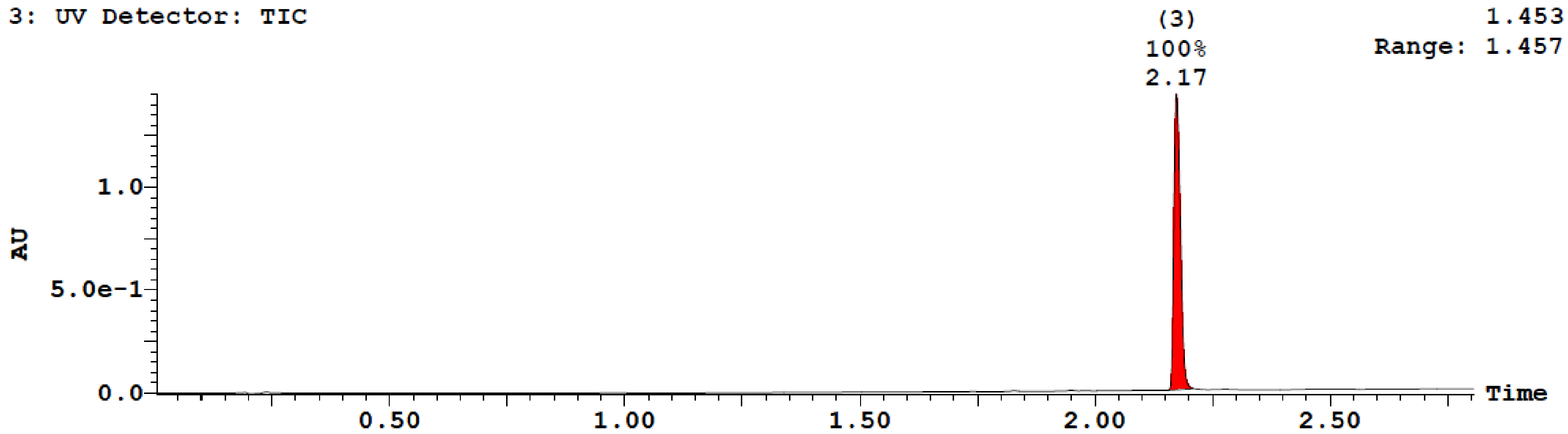

Peak Number	Compound	Time	AreaAbs	Area %Total	Width	Height	Mass Found
3		2.17	2e+004	100.00	0	1e+006	

3:(Time: 2.17) Combine (866:874-(855:859+890:894)) 1:MS ES+ 6.7e+006

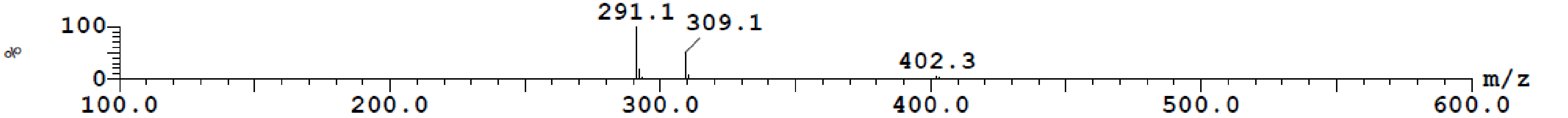

Peak ID	Compound	Time	Mass Found
4		2.18	

14UAMC5435

medchem_140324_M3 1043 (9.084) Cm (1040:1046-1075:1092) 1: TOF MS ES+ 1.00e8

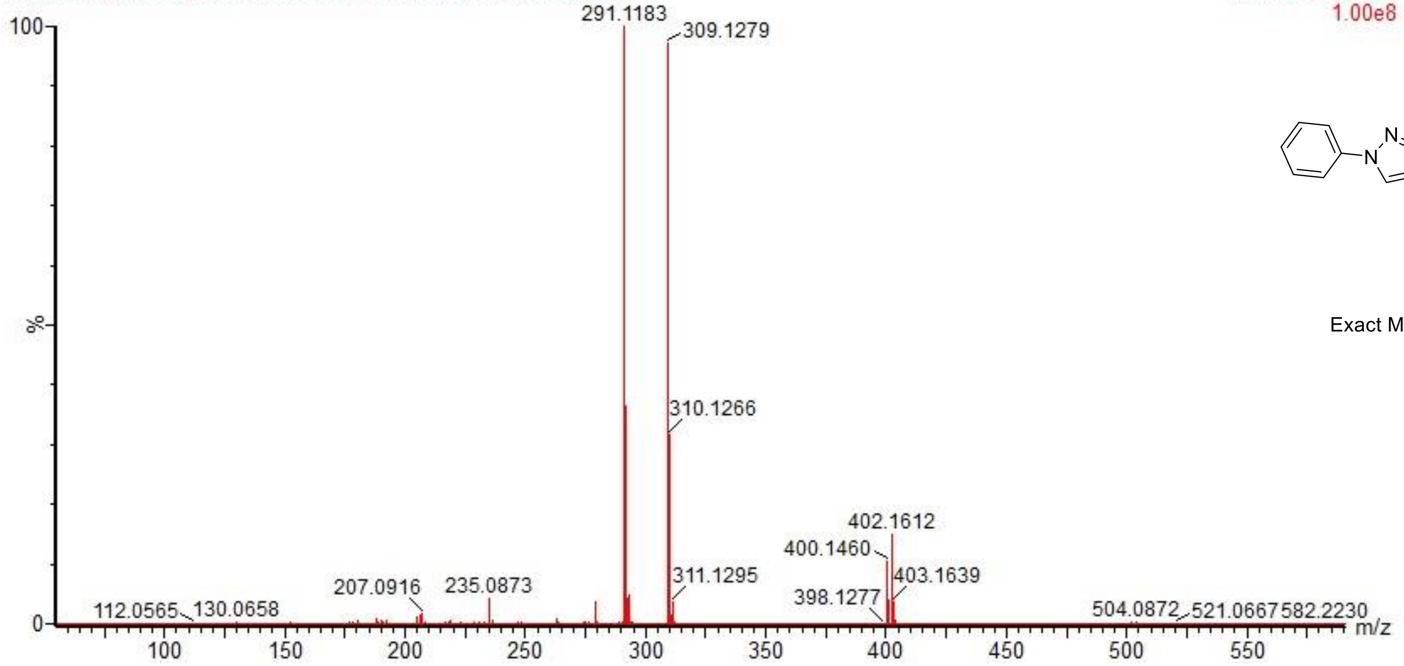

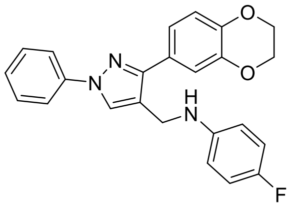

14

Exact Mass: 401,1540

2.3 *N-((3-(2,3-dihydrobenzo[b][1,4]dioxin-6-yl)-1-phenyl-1H-pyrazol-4-yl)methyl)-2-phenylethan-1-amine hydrochloride* **(17)**

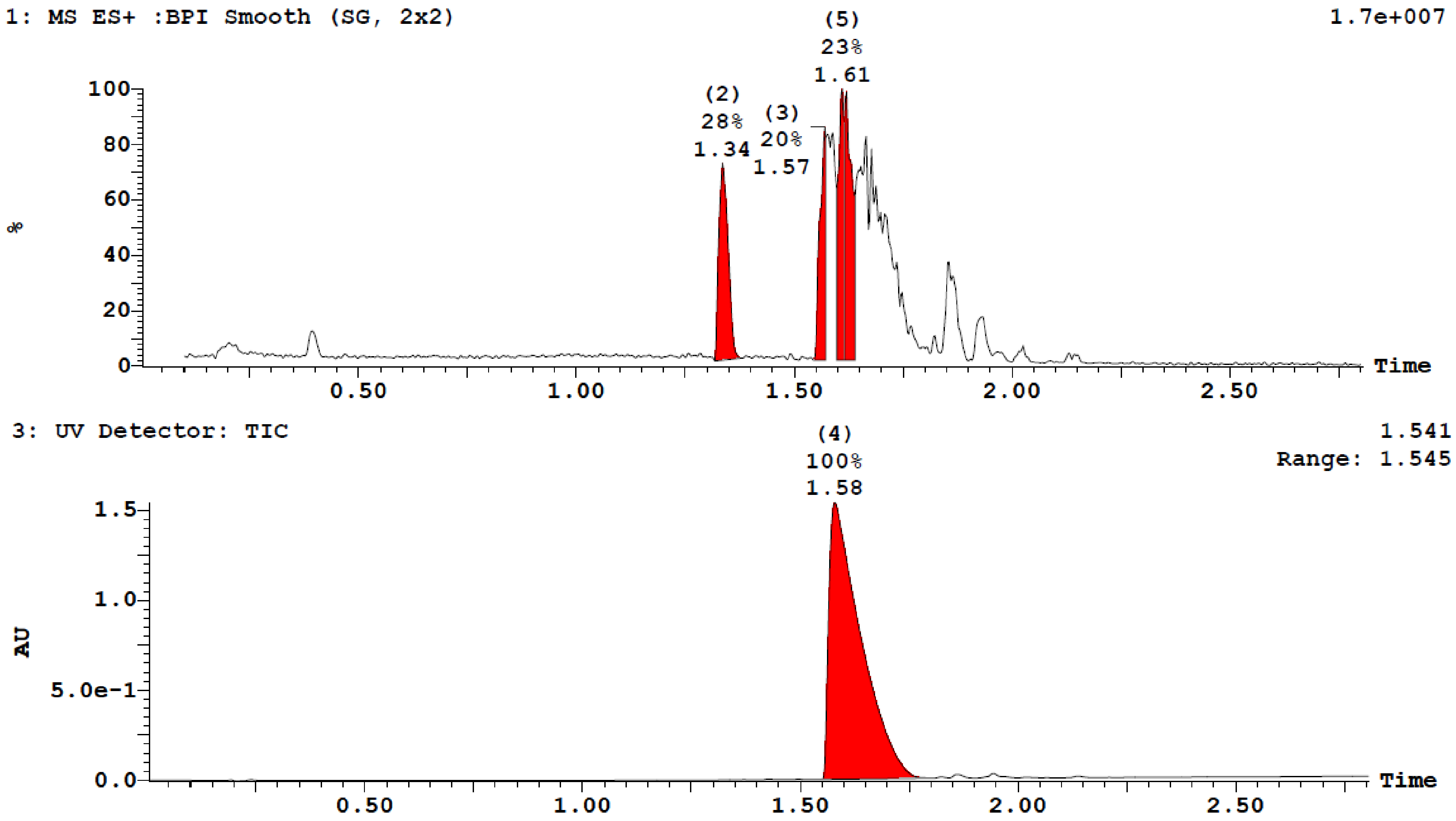

Peak Number	Compound	Time	AreaAbs	Area %Total	Width	Height	Mass Found
4		1.58	1e+005	100.00	0	2e+006	

Peak ID	Compound	Time	Mass Found
4		1.58	

4:(Time: 1.58) Combine (628:636-(610:614+727:731))

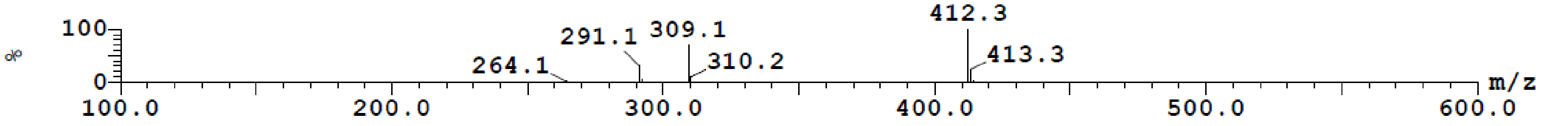

17UAMC5404

medchem_140324_M5 715 (6.232) Cm (711:719-754:784)

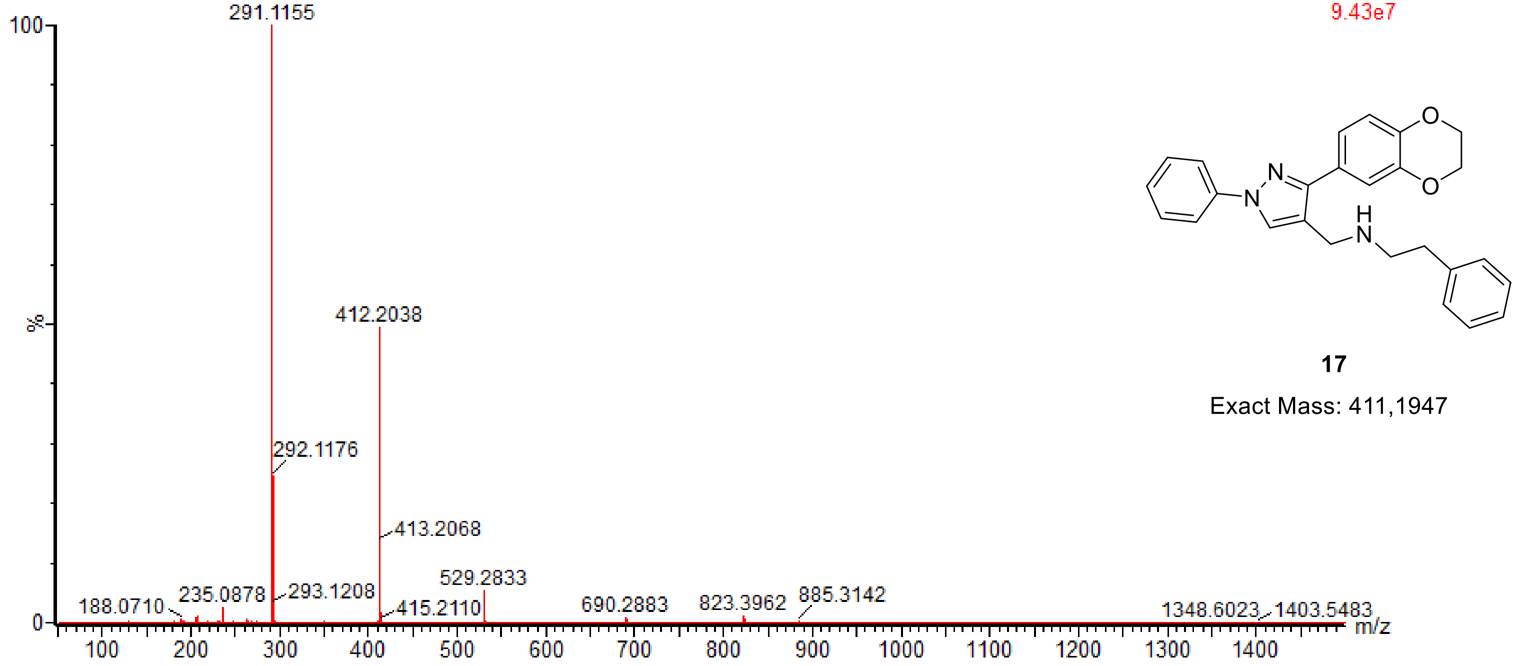

17

Exact Mass: 411,1947

2.4 *4-(4-(((3-(2,3-dihydrobenzo[b][1,4]dioxin-6-yl)-1-phenyl-1H-pyrazol-4-yl)methyl)amino)phenoxy)-N-methylpicolinamide* **(22)**

Sample 1 Vial 2:37 ID UAMC-5410 File UAMC-5410 Date 12-Apr-2023 Time 13:17:45 Description

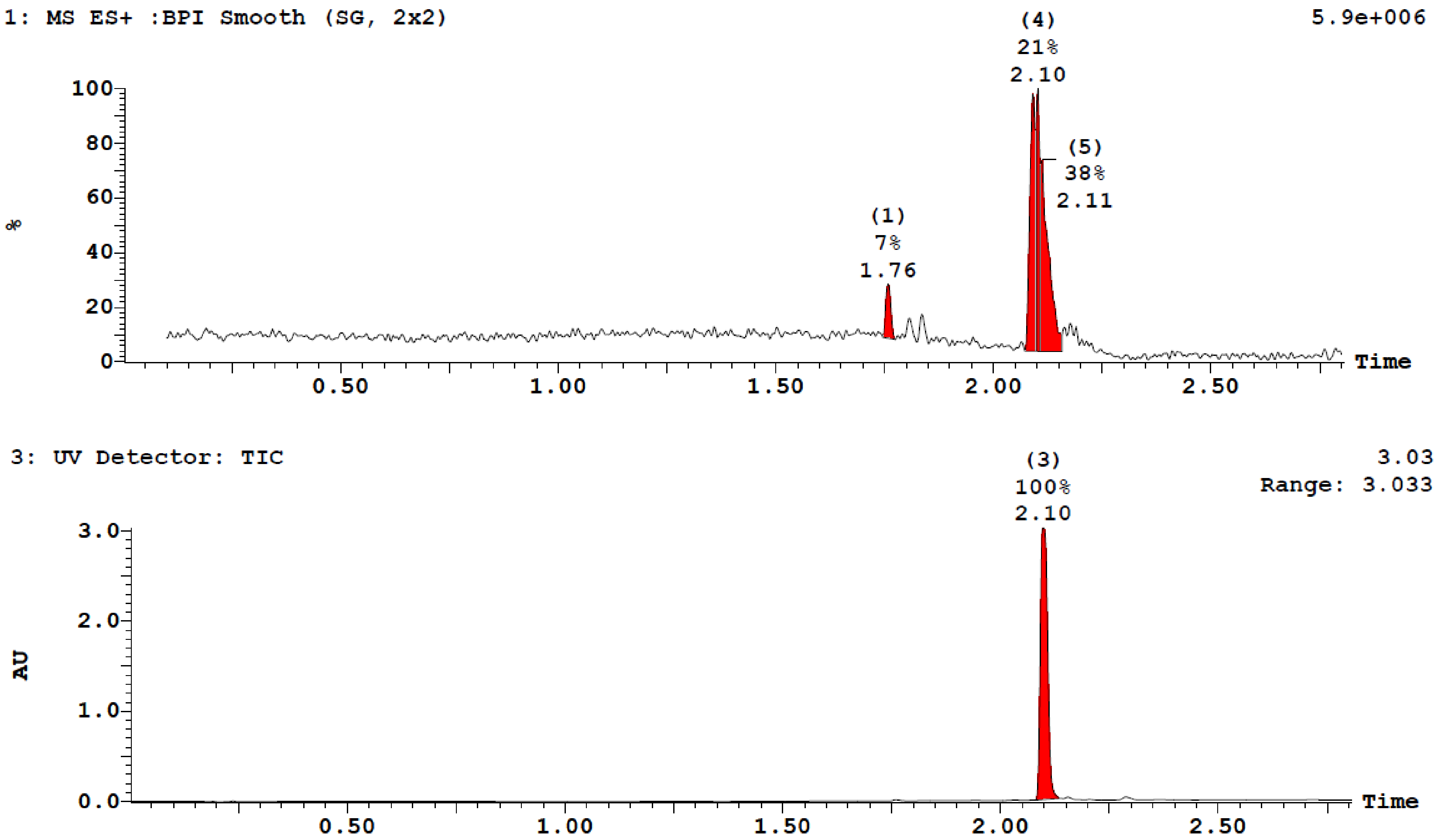

Peak Number	Compound	Time	AreaAbs	Area %Total	Width	Height	Mass Found
3		2.10	6e+004	100.00	0	3e+006	

Peak ID	Compound	Time	Mass Found
3		2.09	

3: (Time: 2.10) Combine (836:844-(824:828+861:865))

1:MS ES+
4.9e+006

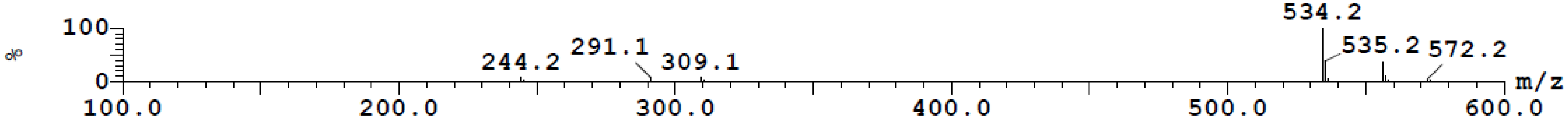

22UAMC5410

medchem_140324_M7 1009 (8.786) Cm (1007:1014-1096:1168)

1: TOF MS ES+
8.72e7

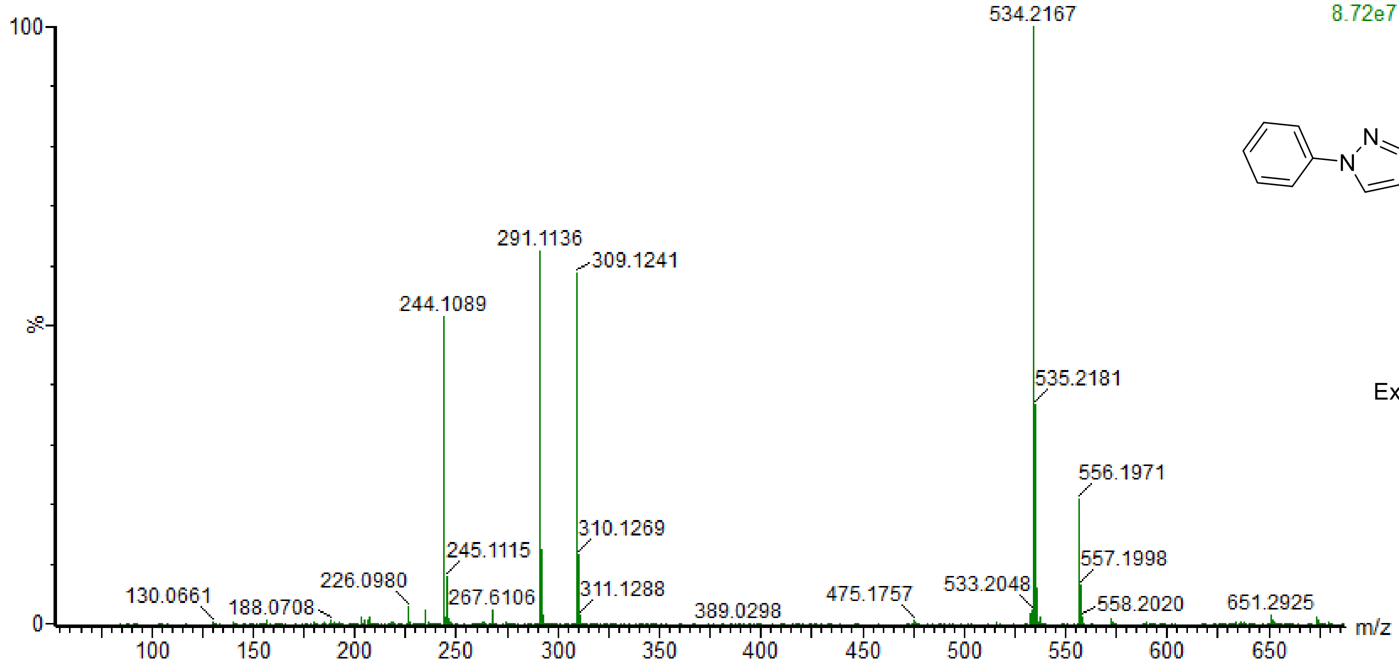

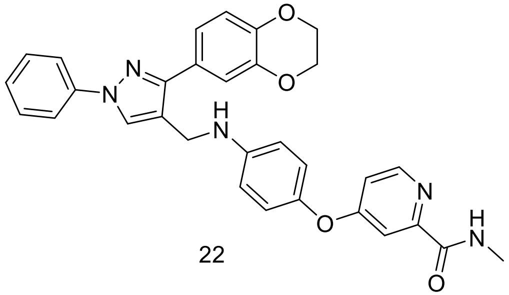

22

Exact Mass: 533,2063

2.5 *N-((3-(2,3-dihydrobenzo[b][1,4]dioxin-6-yl)-1-phenyl-1H-pyrazol-4-yl)methyl)-2-(pyrrolidin-1-yl)ethan-1-amine dihydrochloride* **(26)**

Sample 1 Vial 2:74 ID MB-2021-054B CHK File MB-2021-054B CHK Date 22-Feb-2023 Time 10:17:46 Description

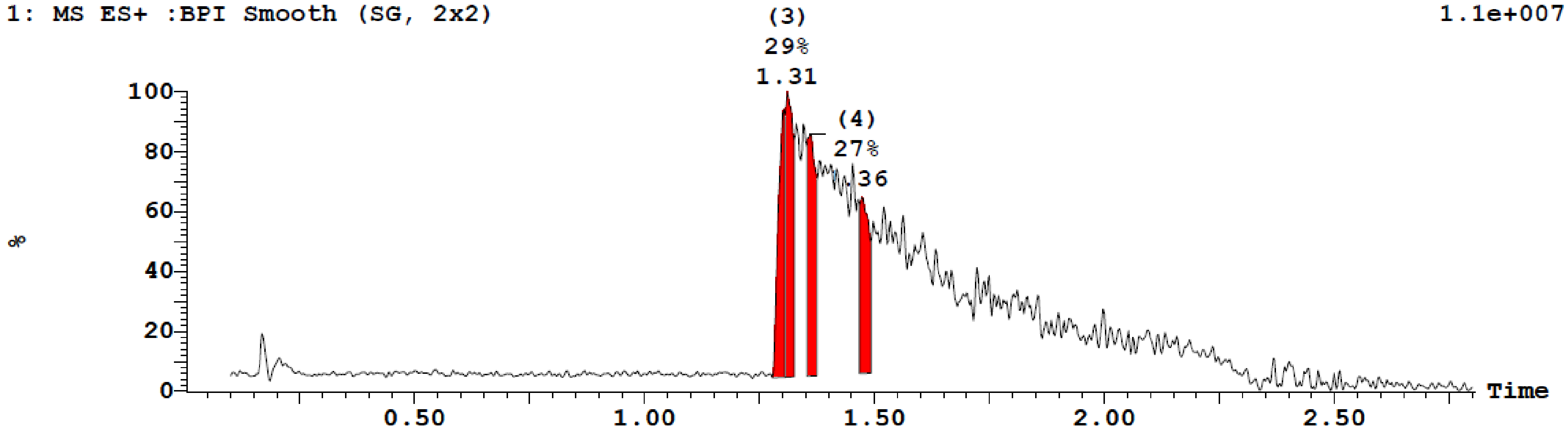

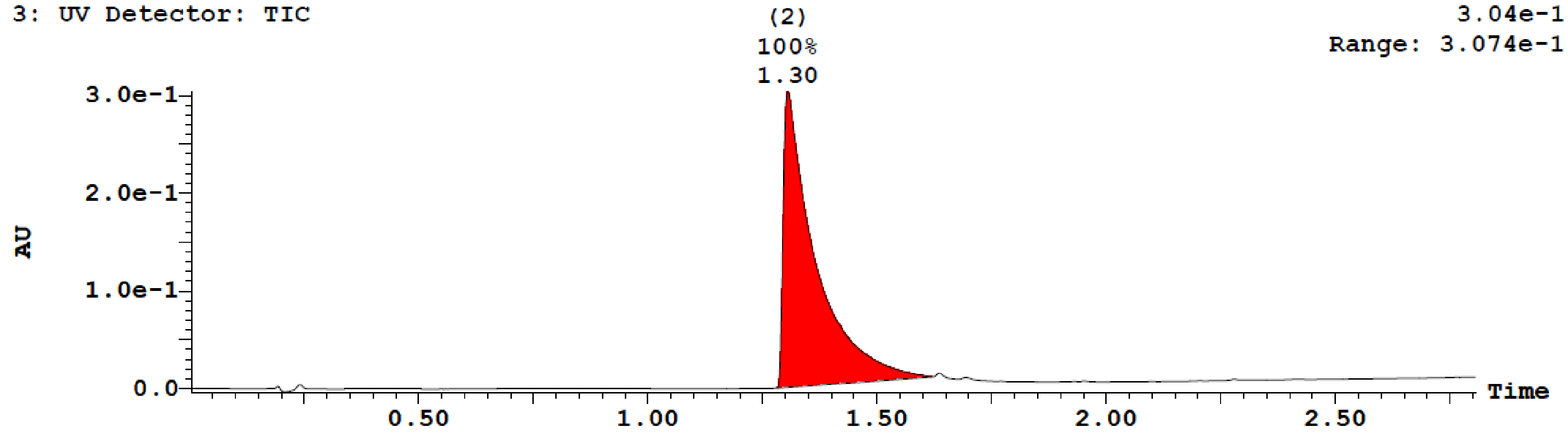

Peak Number	Compound	Time	AreaAbs	Area %Total	Width	Height	Mass Found
2		1.30	2e+004	100.00	0	3e+005	

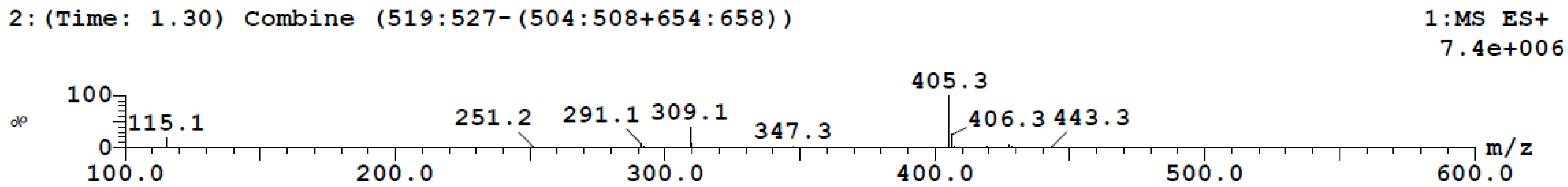

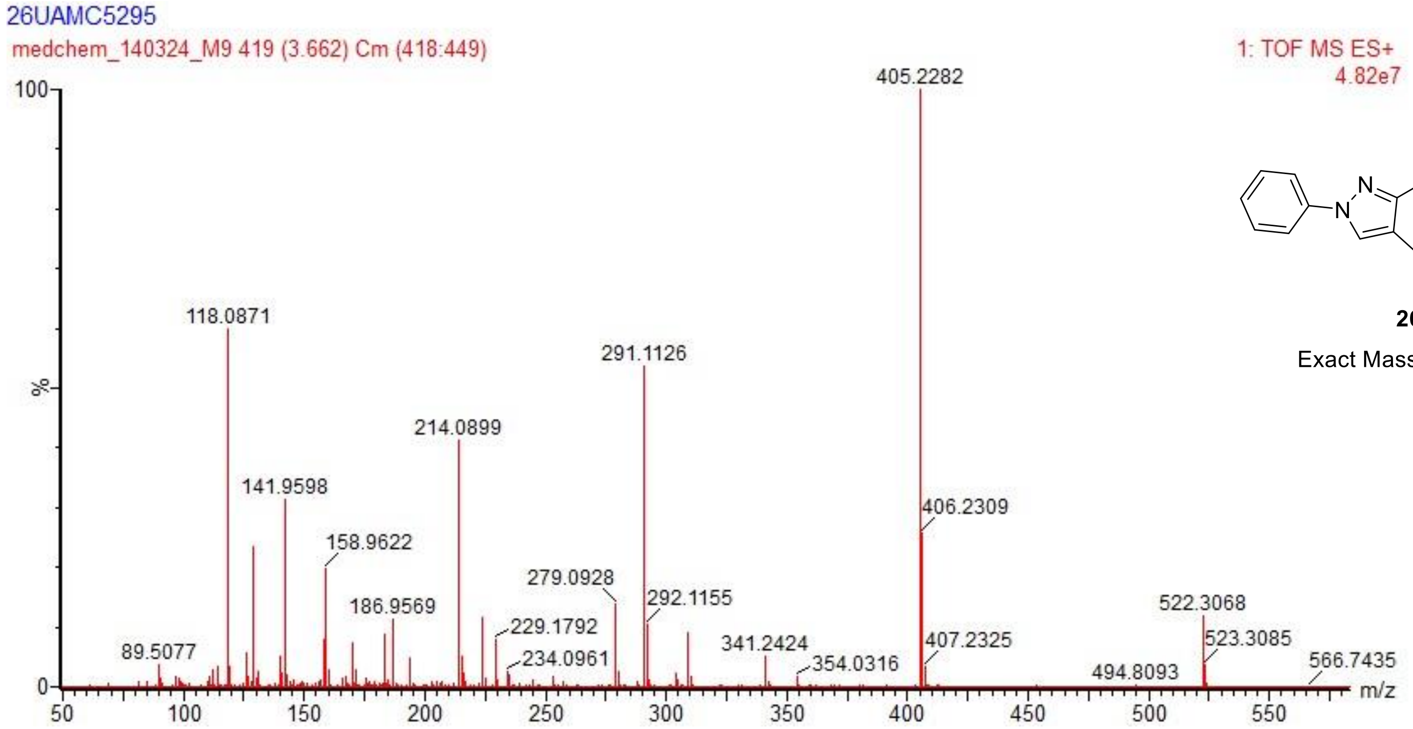

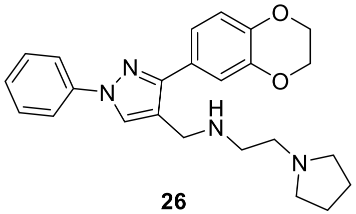

26

Exact Mass: 404,2212

2.6 *N*[1]*-((3-(2,3-dihydrobenzo[b][1,4]dioxin-6-yl)-1-phenyl-1H-pyrazol-4-yl)methyl)-N*[3]*,N*[3]*-dimethylpropane-1,3-diamine dihydrochloride* **(29)**

Sample 1 Vial 2:8 ID MB-2023-019 HCl File MB-2023-019 HCl Date 17-Feb-2023 Time 14:19:41 Description

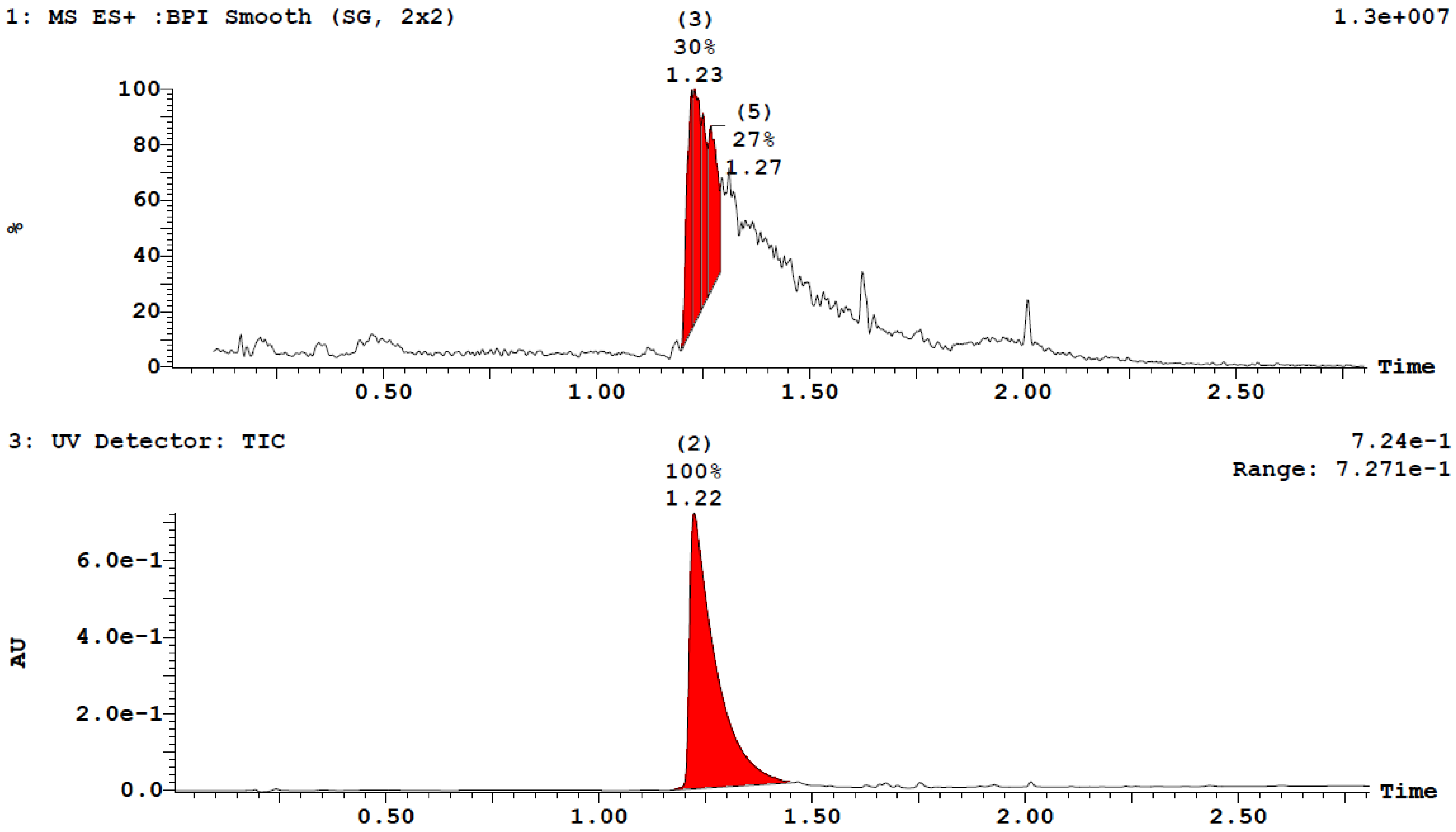

Peak Number	Compound	Time	AreaAbs	Area %Total	Width	Height	Mass Found
2		1.22	5e+004	100.00	0	7e+005	

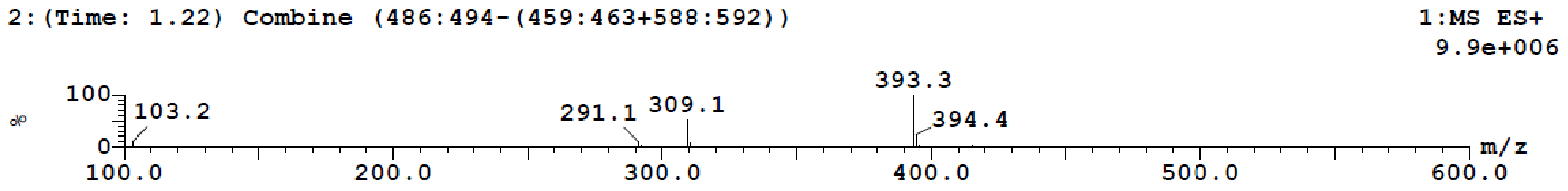

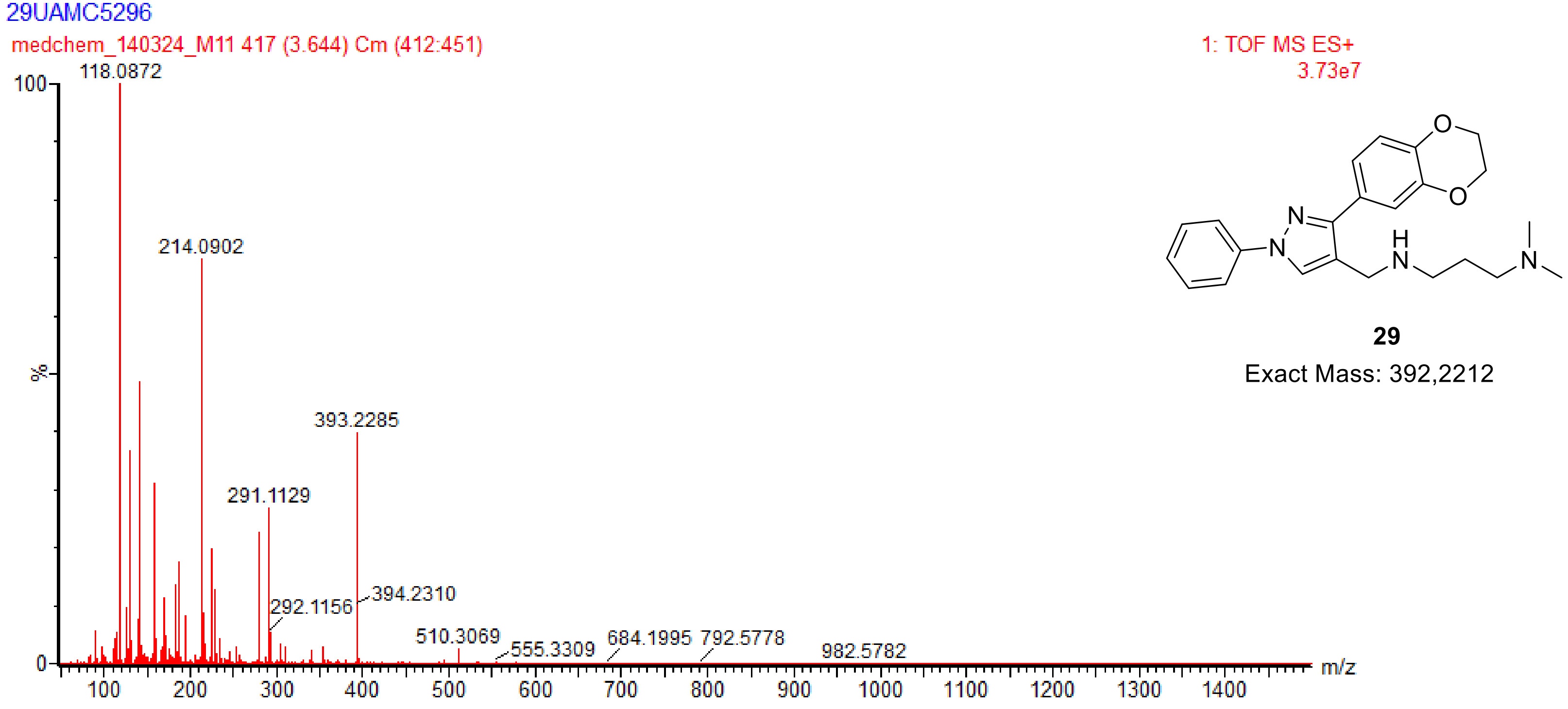

29

Exact Mass: 392,2212

2.7 *3-(2,3-dihydrobenzo[b][1,4]dioxin-6-yl)-N-(4-fluorobenzyl)-1-phenyl-1H-pyrazole-4-carboxamide* (33)

Sample 1 Vial 2:9 ID UAMC-5391 File UAMC-5391 Date 12-Apr-2023 Time 10:47:22 Description

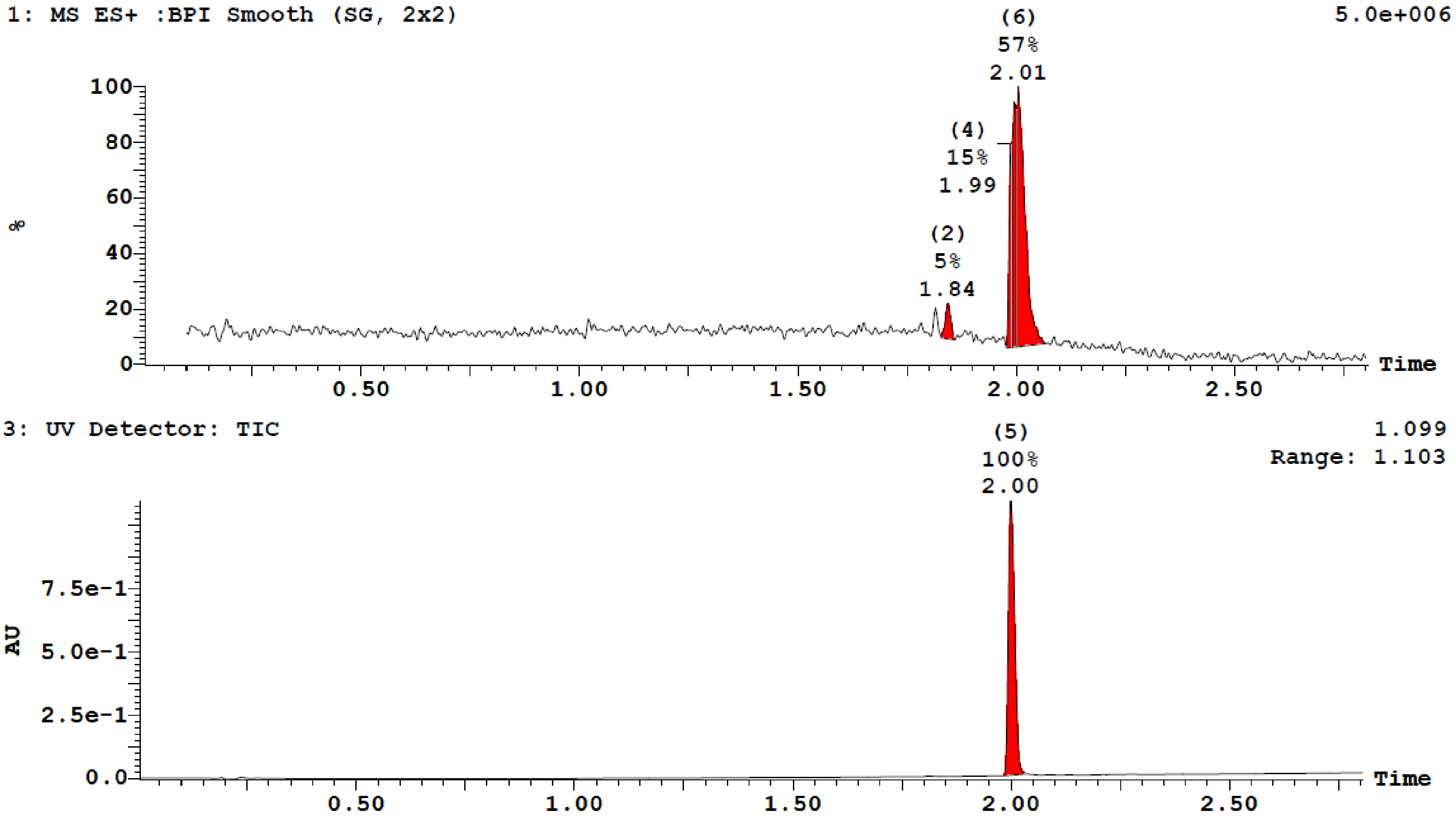

Peak Number	Compound	Time	AreaAbs	Area %Total	Width	Height	Mass Found
5		2.00	2e+004	100.00	0	1e+006	

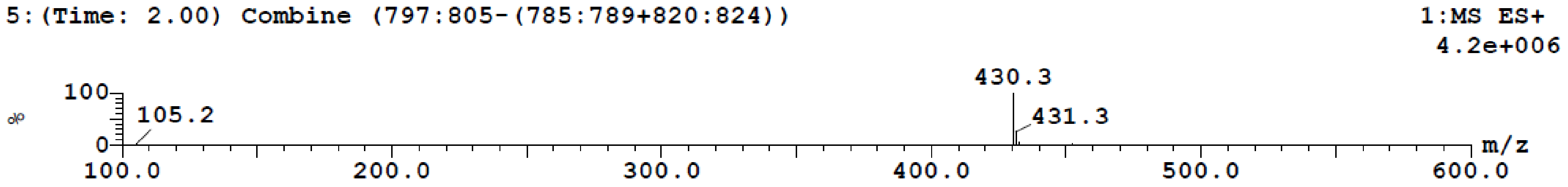

Peak ID	Compound	Time	Mass Found
6		2.01	

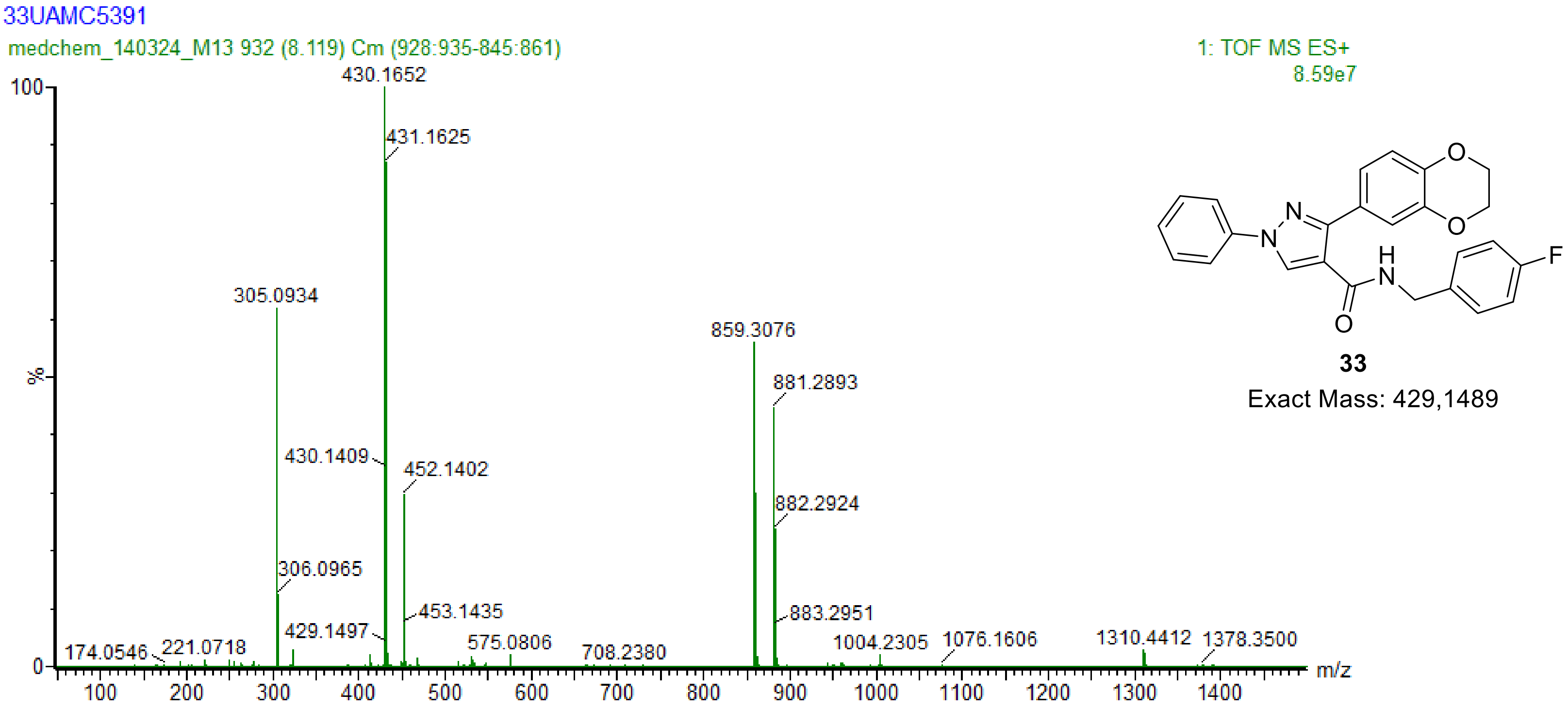

33

Exact Mass: 429,1489

2.8 *3-(2,3-dihydrobenzo[b][1,4]dioxin-6-yl)-1-phenyl-N-(2-(pyridin-4-yl)ethyl)-1H-pyrazole-4-carboxamide* (**35**)

Sample 1 Vial 2:13 ID UAMC-5395 File UAMC-5395 Date 12-Apr-2023 Time 11:04:05 Description

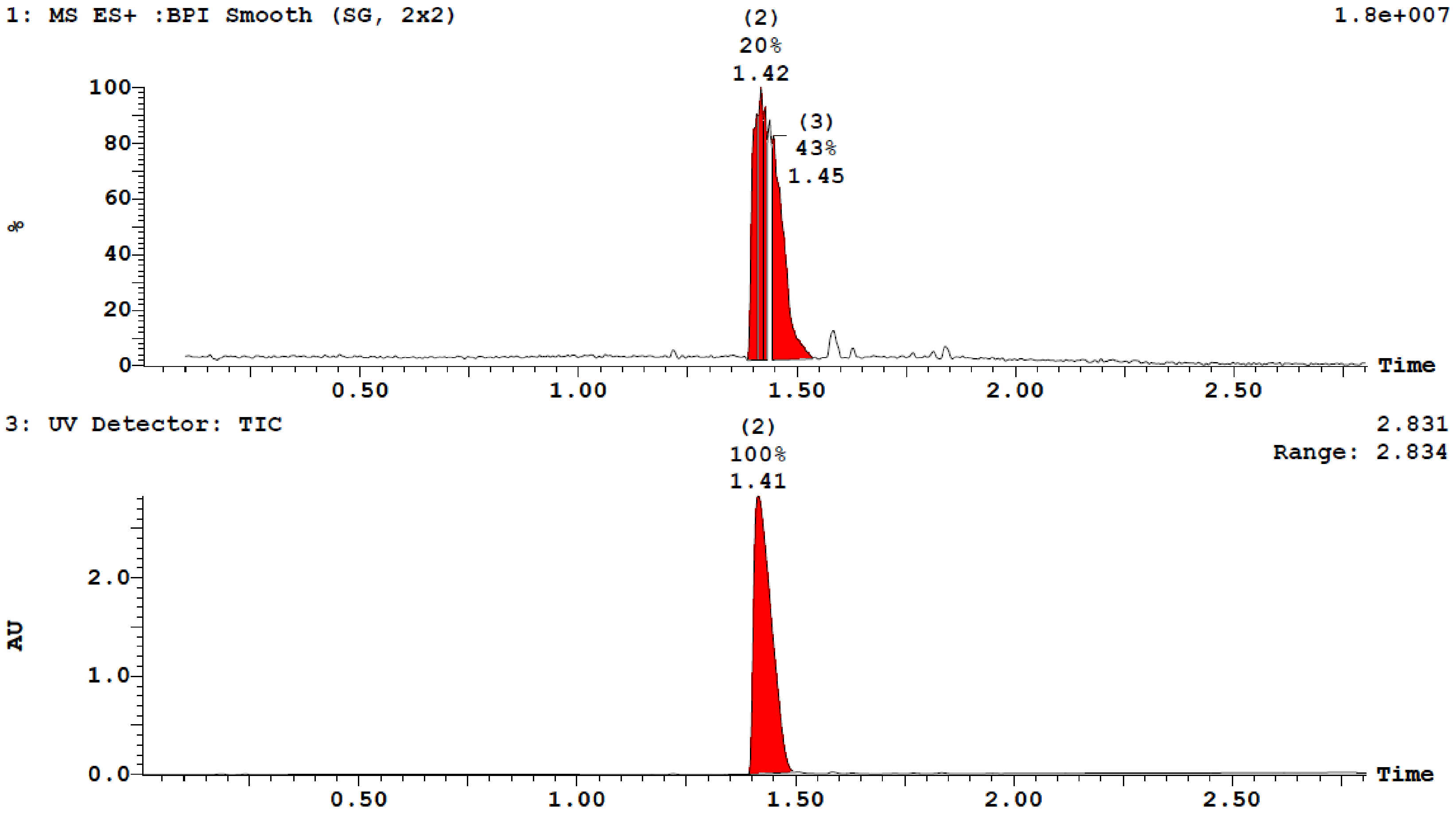

Peak Number	Compound	Time	AreaAbs	Area %Total	Width	Height	Mass Found
2		1.41	1e+005	100.00	0	3e+006	

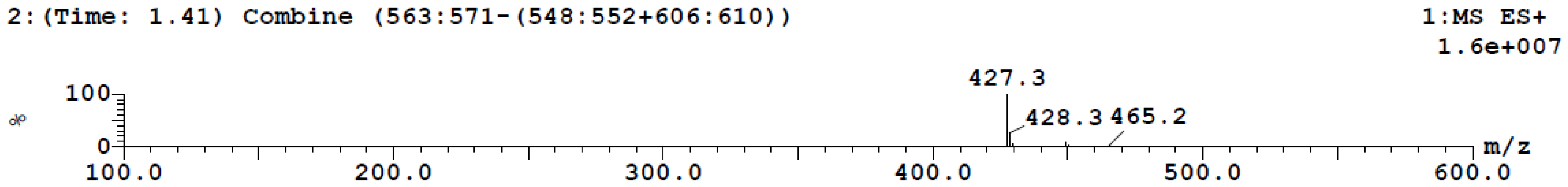

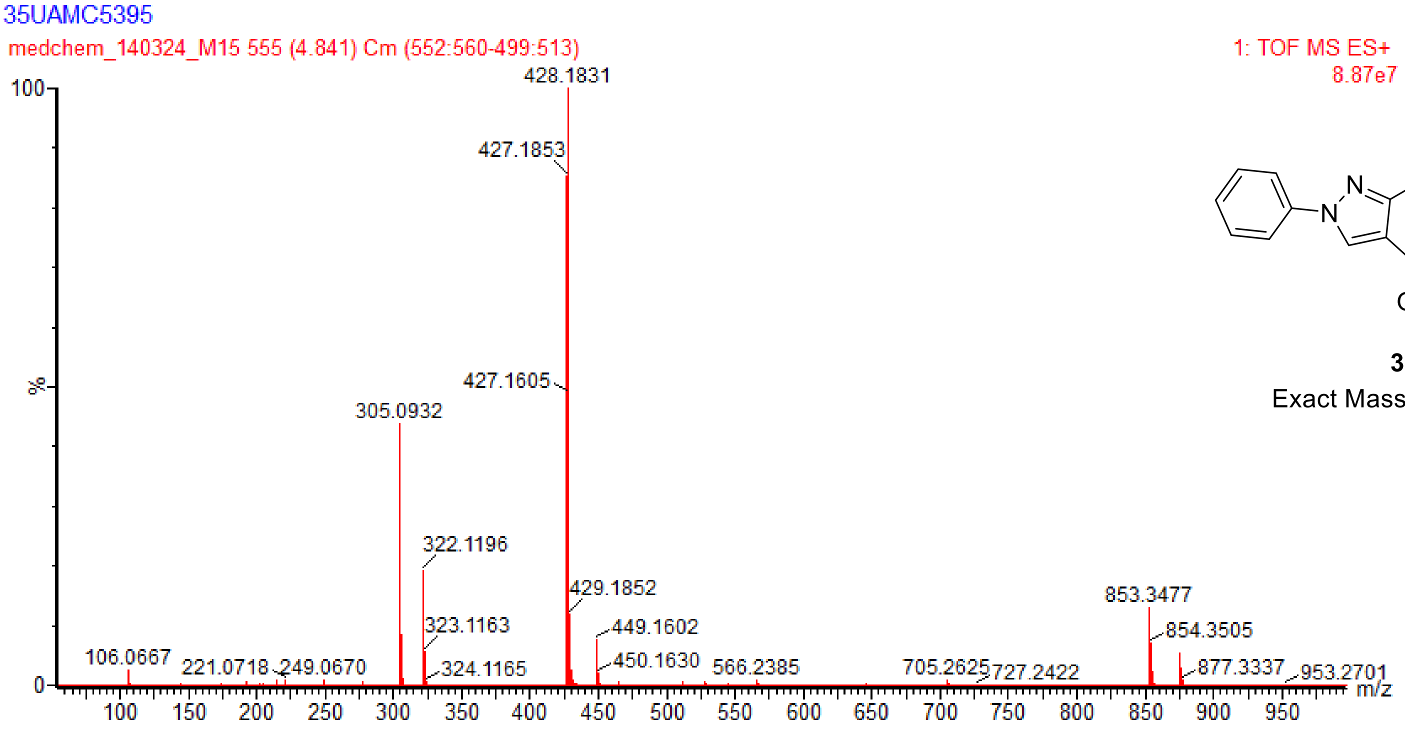

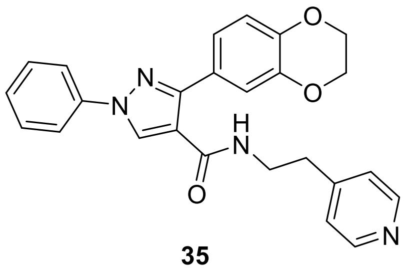

35

Exact Mass: 426,1692

2.9 4-(4-(3-(2,3-dihydrobenzo[b][1,4]dioxin-6-yl)-1-phenyl-1H-pyrazole-4-carboxamido)phenoxy)-N-methylpicolinamide **(38)**

Sample 1 Vial 1:50 ID UAMC-5393-2 File UAMC-5393-2 Date 13-Apr-2023 Time 16:59:32 Description

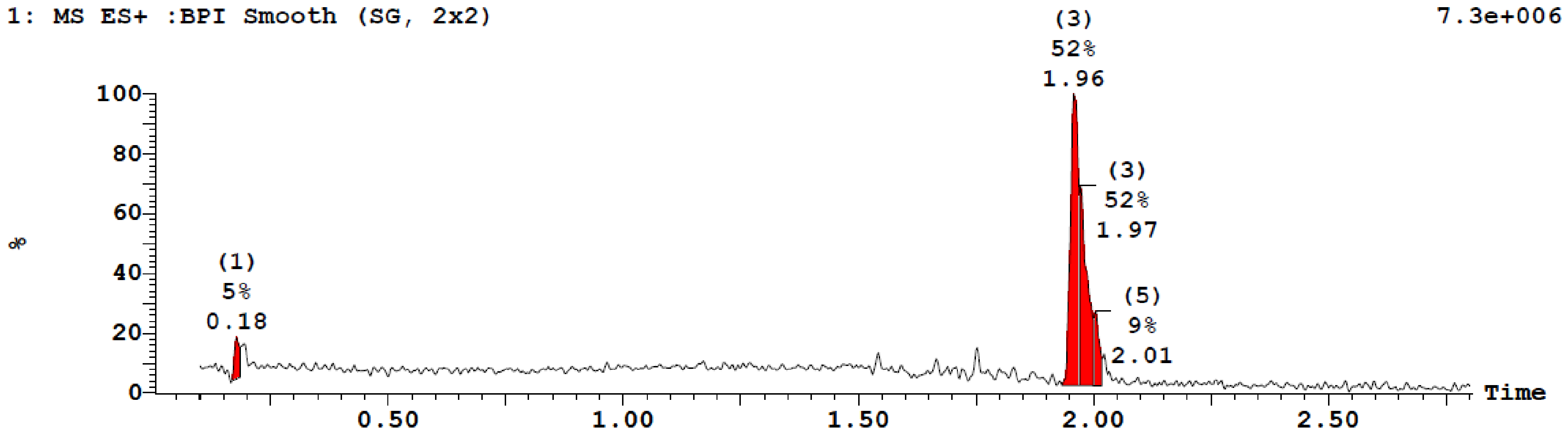

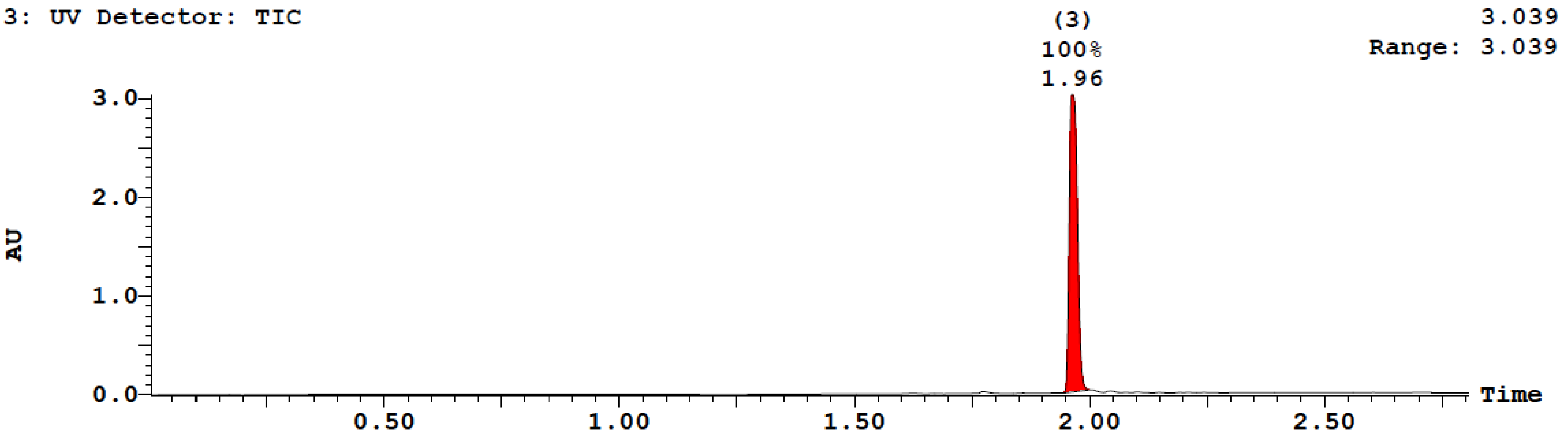

Peak Number	Compound	Time	AreaAbs	Area %Total	Width	Height	Mass Found
3		1.96	6e+004	100.00	0	3e+006	

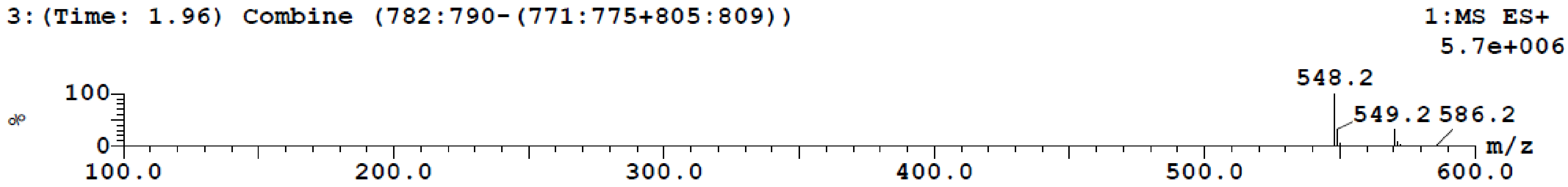

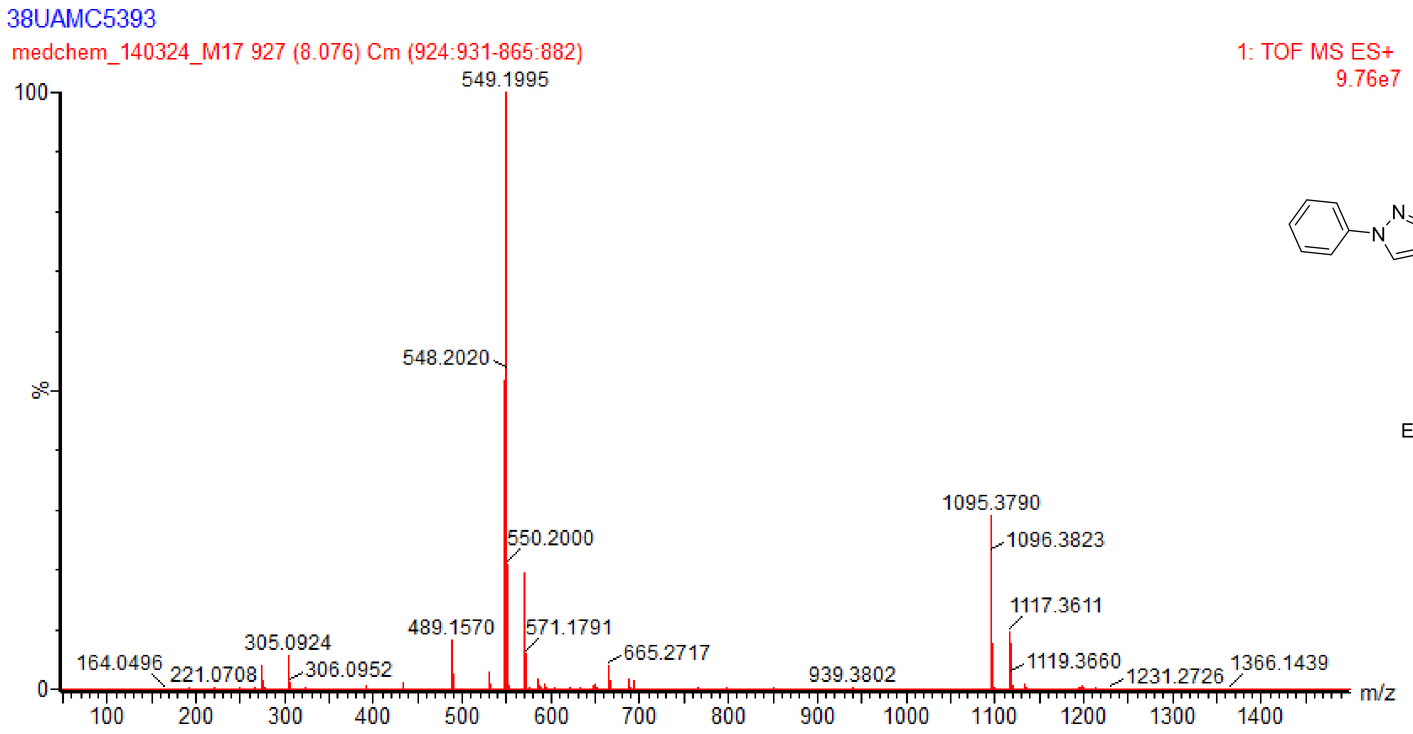

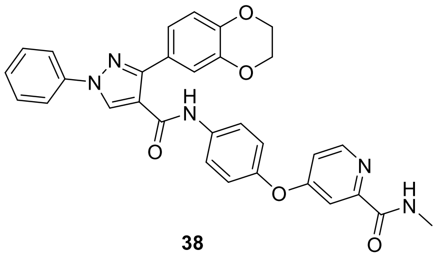

38

Exact Mass: 547,1856

2.10 N-((1r,3r,5r,7r)-adamantan-2-yl)-3-(2,3-dihydrobenzo[b][1,4]dioxin-6-yl)-1-phenyl-1H-pyrazole-4-carboxamide **(41)**

Sample 1 Vial 2:19 ID UAMC-5398 File UAMC-5398 Date 12-Apr-2023 Time 11:38:24 Description

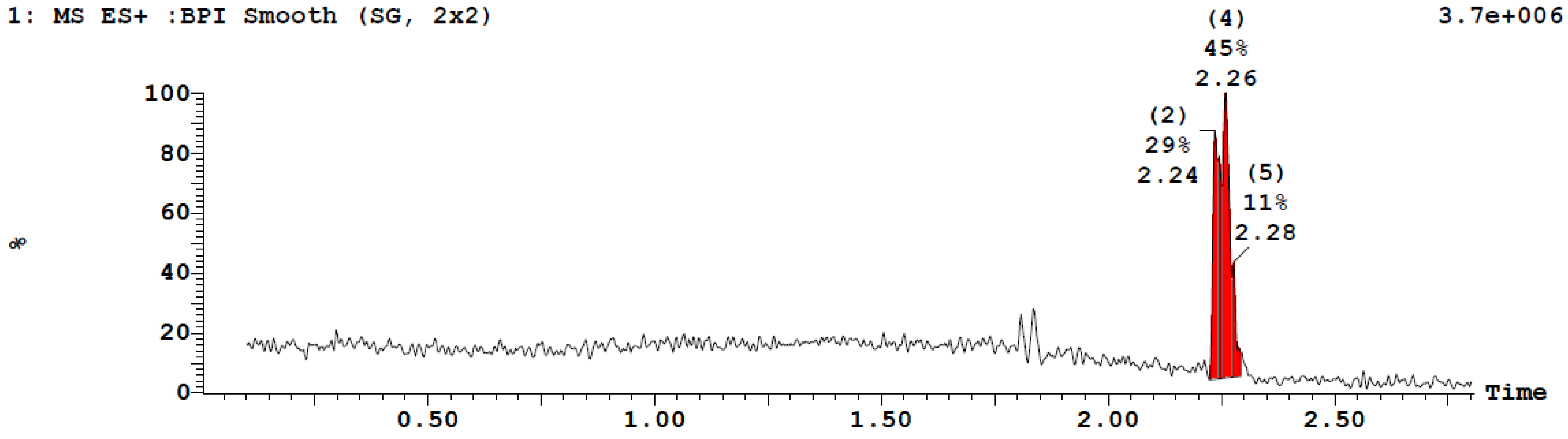

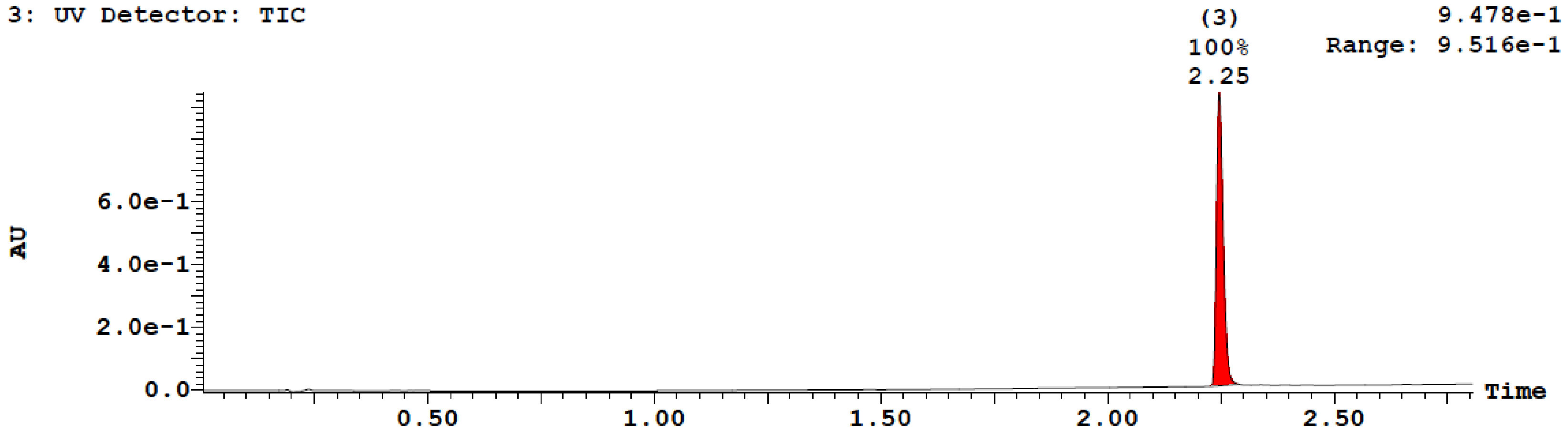

Peak Number	Compound	Time	AreaAbs	Area %Total	Width	Height	Mass Found
3		2.25	2e+004	100.00	0	9e+005	

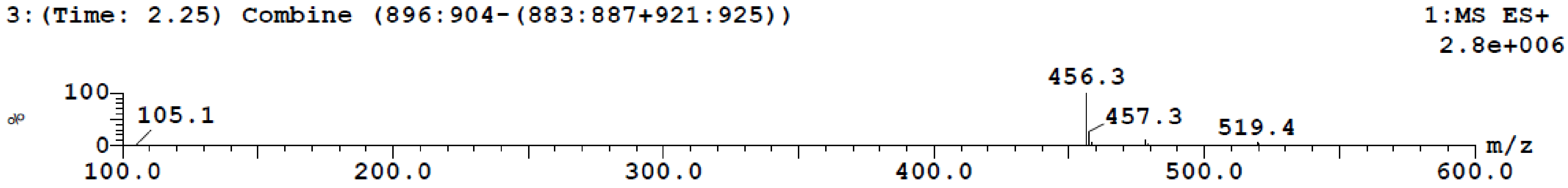

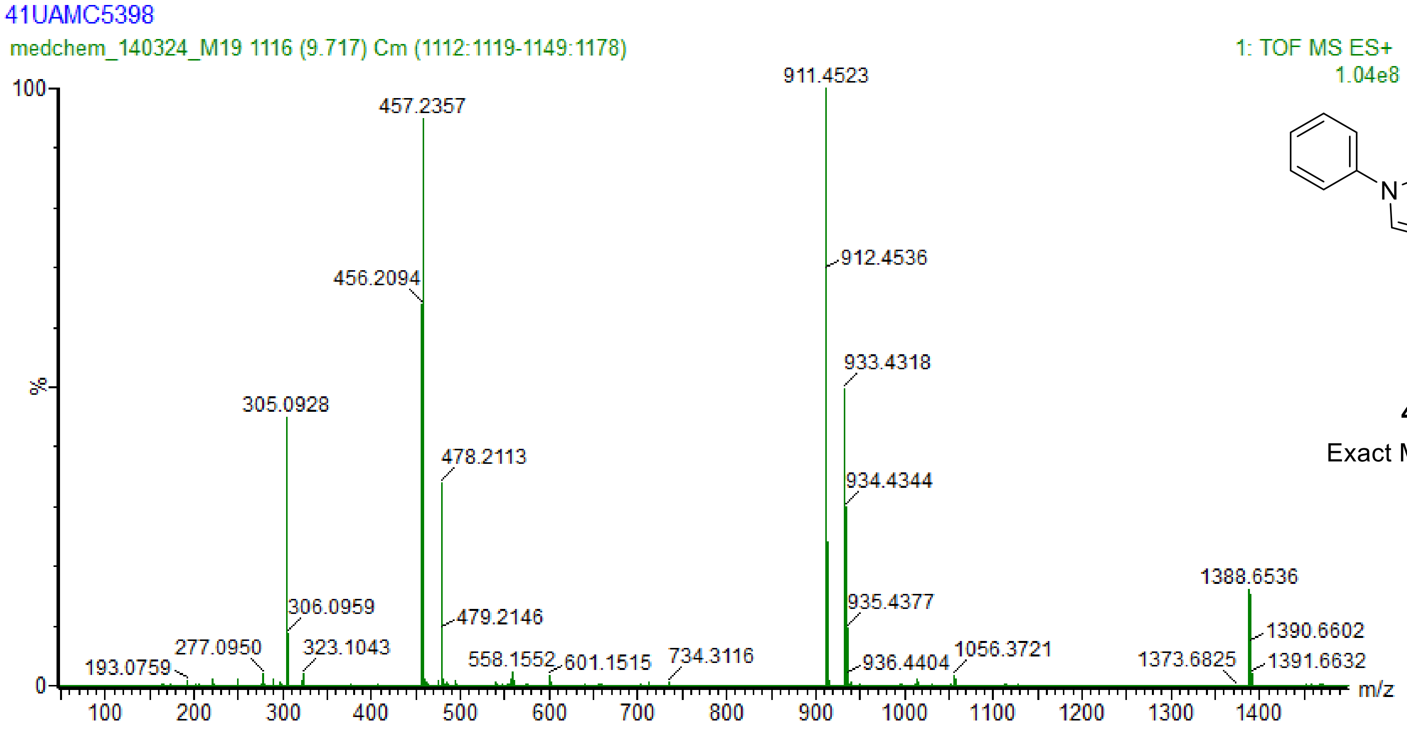

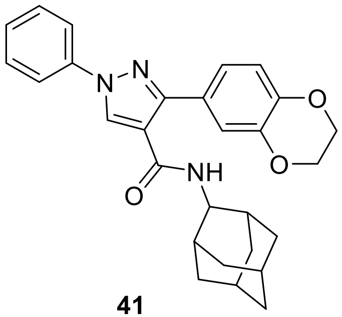

41

Exact Mass: 455,2209

2.11 *3-(2,3-dihydrobenzo[b][1,4]dioxin-6-yl)-N-(2-morpholinoethyl)-1-phenyl-1H-pyrazole-4-carboxamide hydrochloride* (**44**)

Sample 1 Vial 2:9 ID UAMC-5384 ACID FREE File UAMC-5384 ACID FREE Date 04-Nov-2023 Time 17:18:47 Description

1: MS ES+ :BPI Smooth (SG, 2x2) 9.8e+006

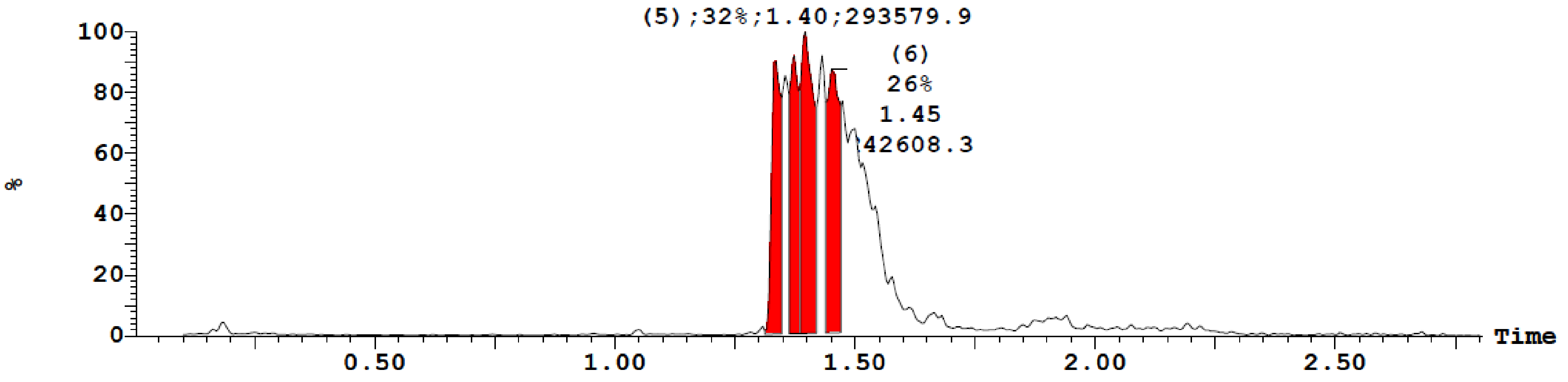

3: UV Detector: TIC 2.09

Range: 2.094

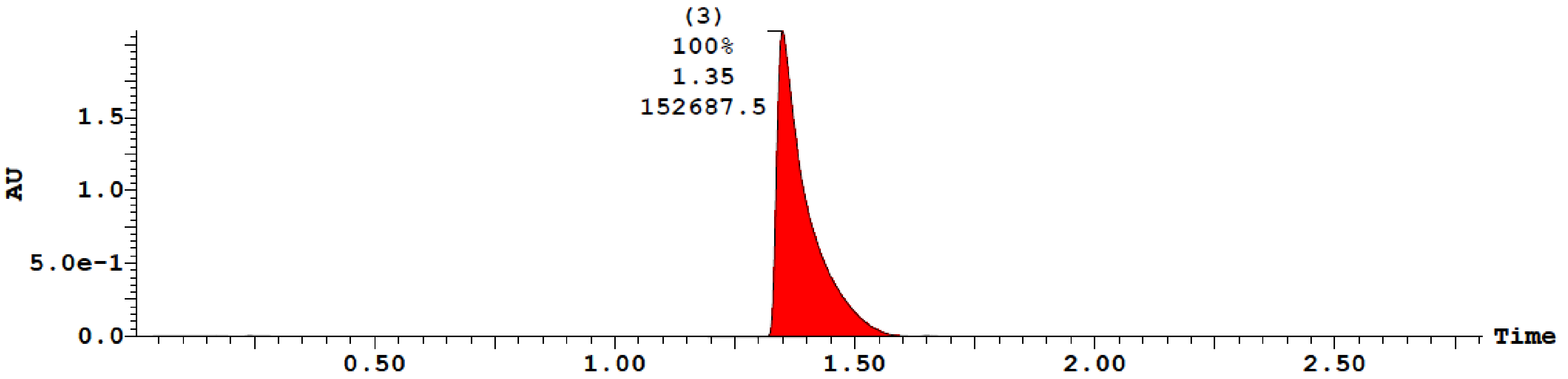

Peak Number	Compound	Time	AreaAbs	Area %Total	Width	Height	Mass Found
3		1.35	2e+005	100.00	0	2e+006	

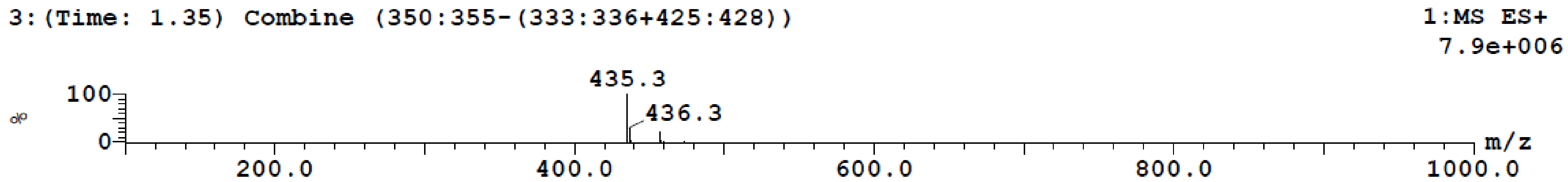

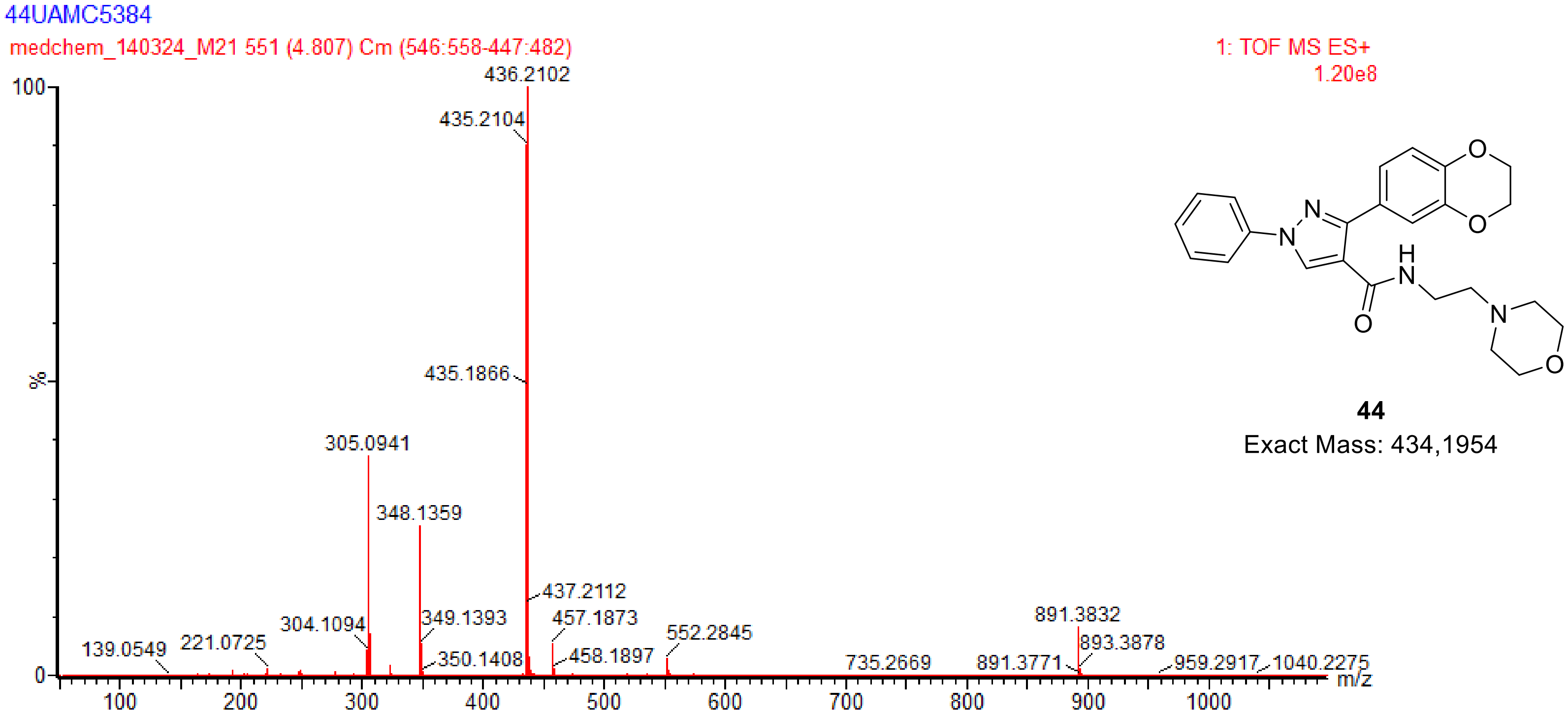

44

Exact Mass: 434,1954

2.12 *1-benzyl-4-(((3-(2,3-dihydrobenzo[b][1,4]dioxin-6-yl)-1-phenyl-1H-pyrazol-4-yl)methoxy)methyl)-1H-1,2,3-triazole* (48)

Sample 1 Vial 2:5 ID UAMC-5387 File UAMC-5387 Date 12-Apr-2023 Time 10:30:38 Description

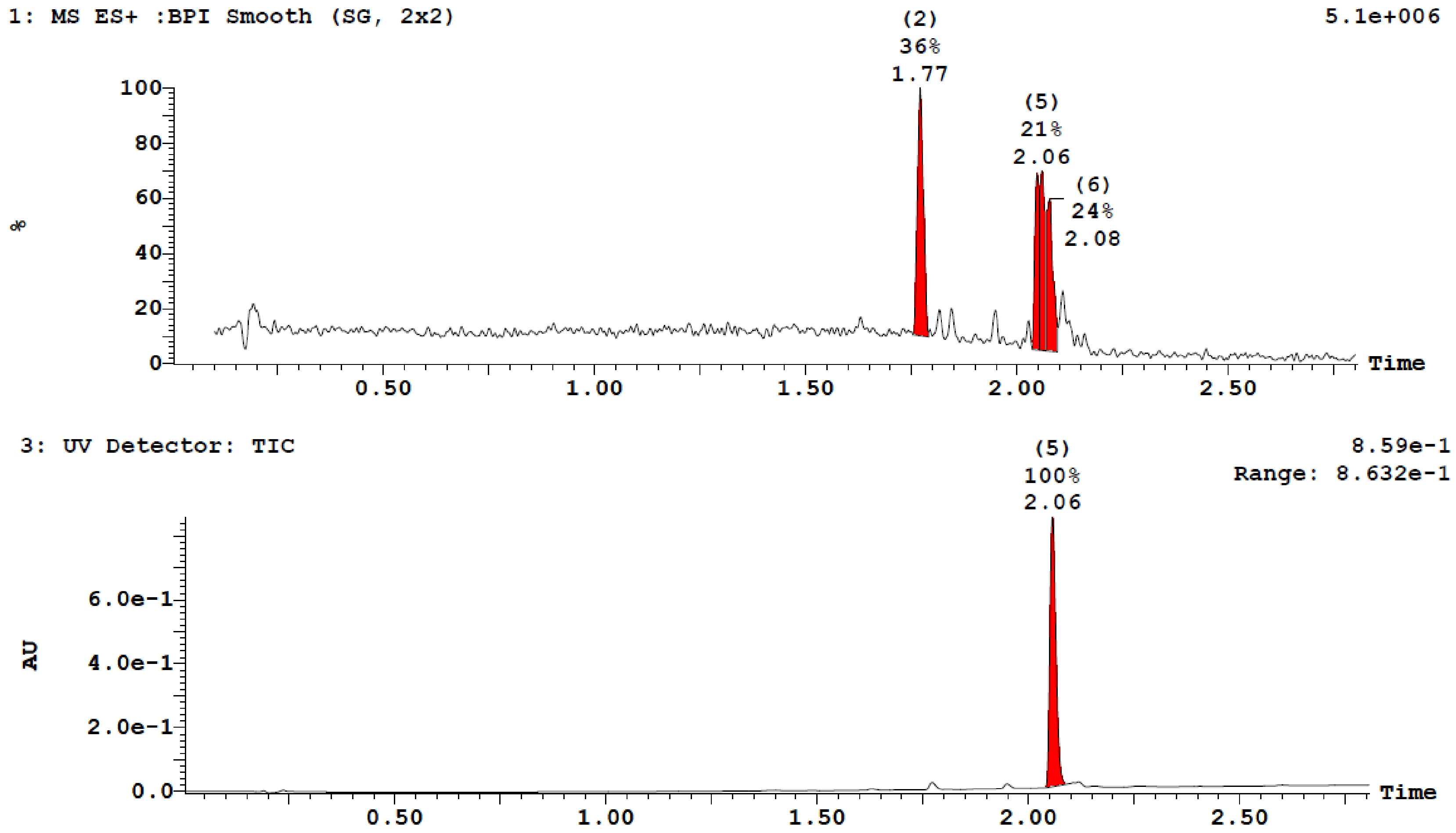

Peak Number	Compound	Time	AreaAbs	Area %Total	Width	Height	Mass Found
5		2.06	1e+004	100.00	0	8e+005	

5:(Time: 2.06) Combine (820:828-(809:813+842:846))

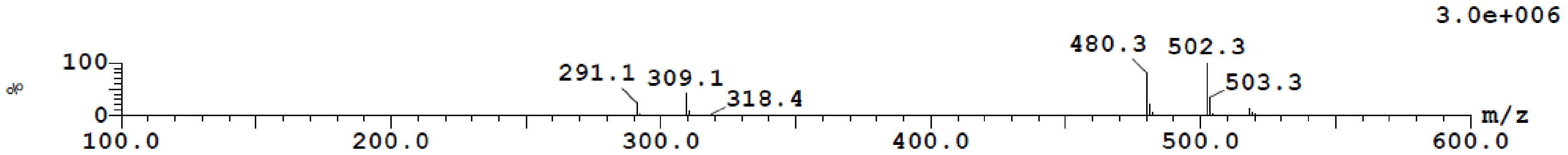

Peak ID	Compound	Time	Mass Found
6		2.08	

48UAMC5387

medchem_140324_M23 980 (8.537) Cm (974:987-820:876)

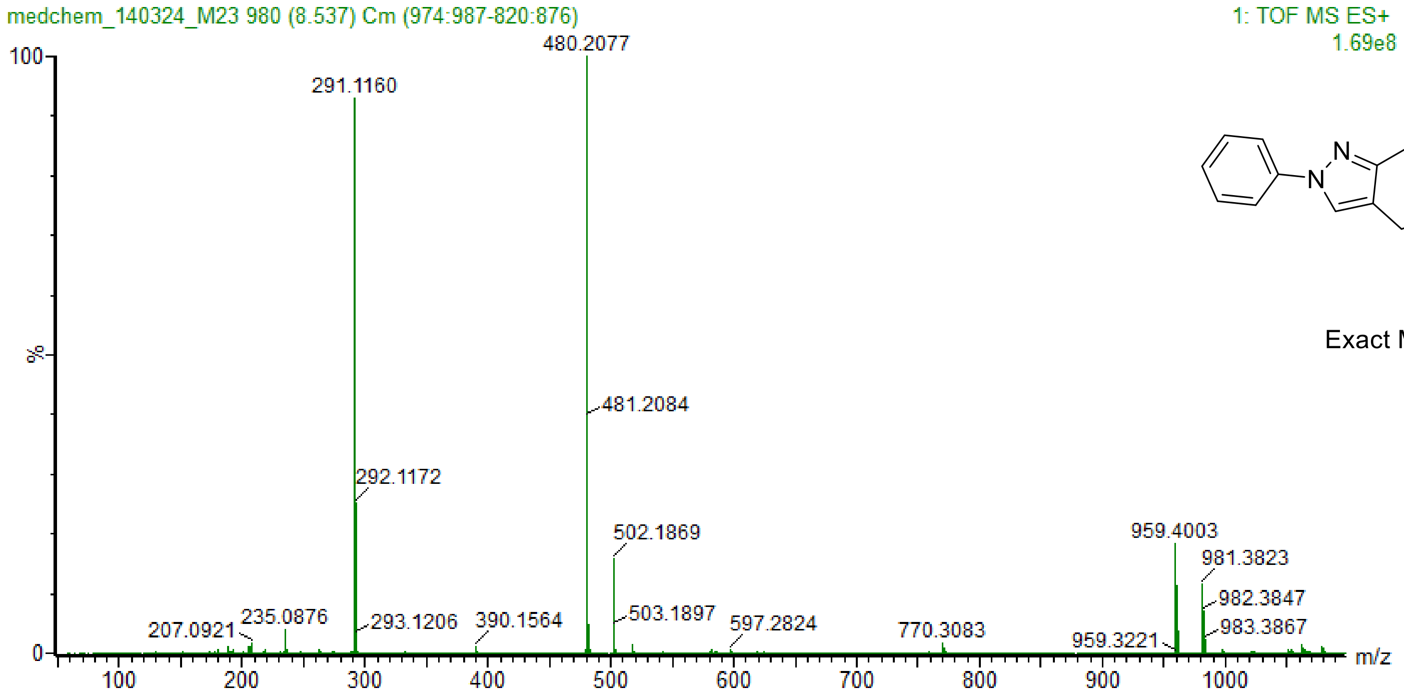

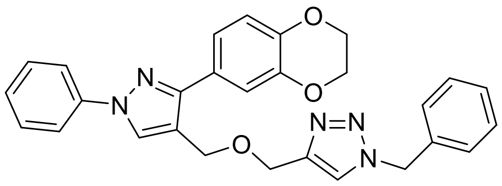

48

Exact Mass: 479,1957

2.13 *4-benzyl-3-(2,3-dihydrobenzo[b][1,4]dioxin-6-yl)-1-phenyl-1H-pyrazole* (**49**)

Sample 1 Vial 2:12 ID UAMC-5394 File UAMC-5394 Date 12-Apr-2023 Time 10:59:54 Description

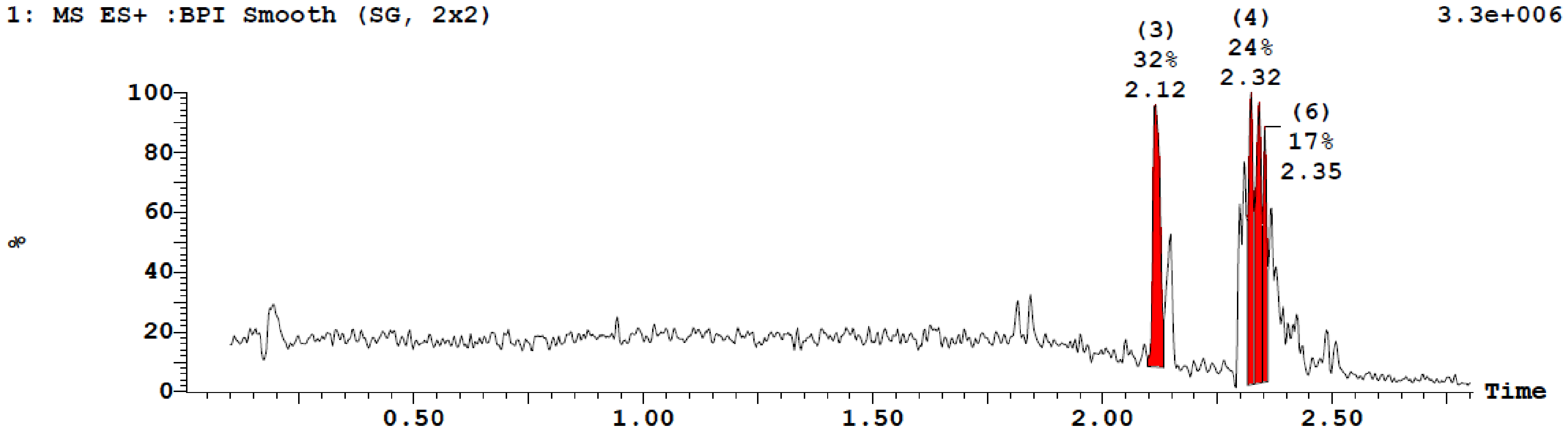

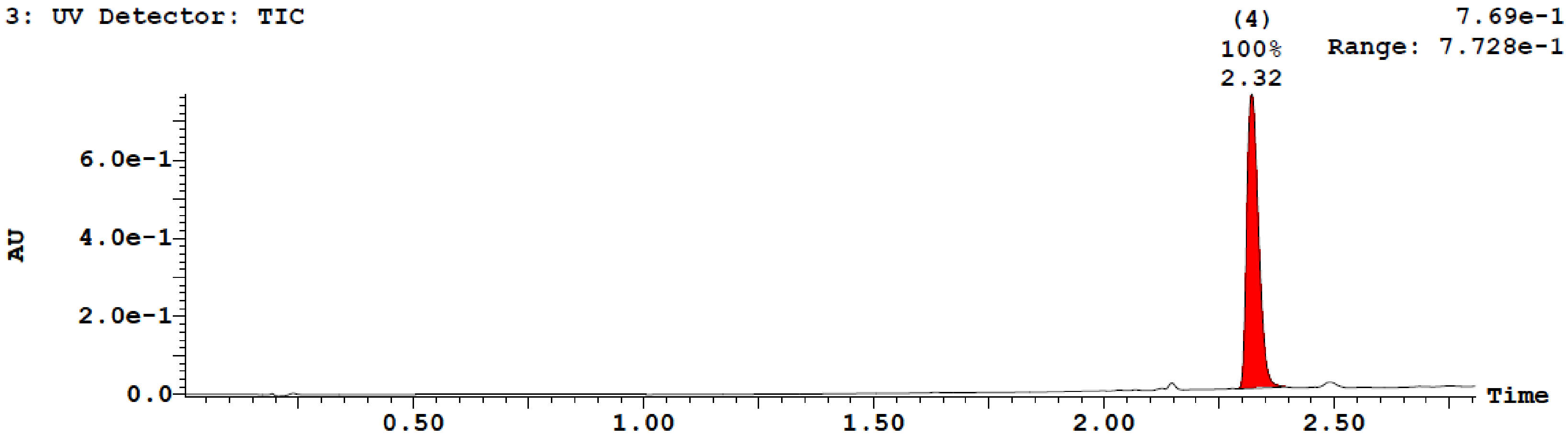

Peak Number	Compound	Time	AreaAbs	Area %Total	Width	Height	Mass Found
4		2.32	2e+004	100.00	0	8e+005	

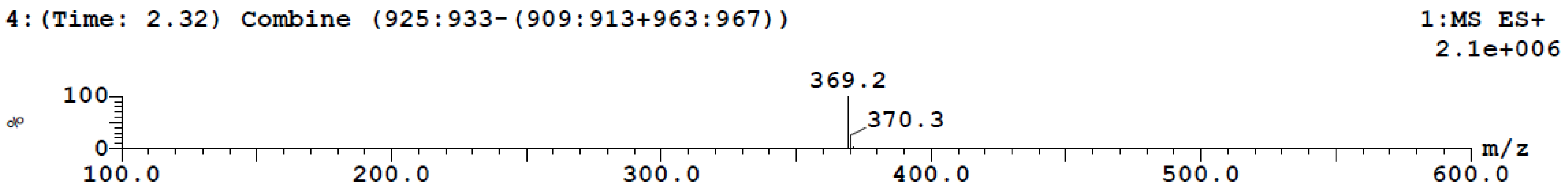

Peak ID	Compound	Time	Mass Found
5		2.34	

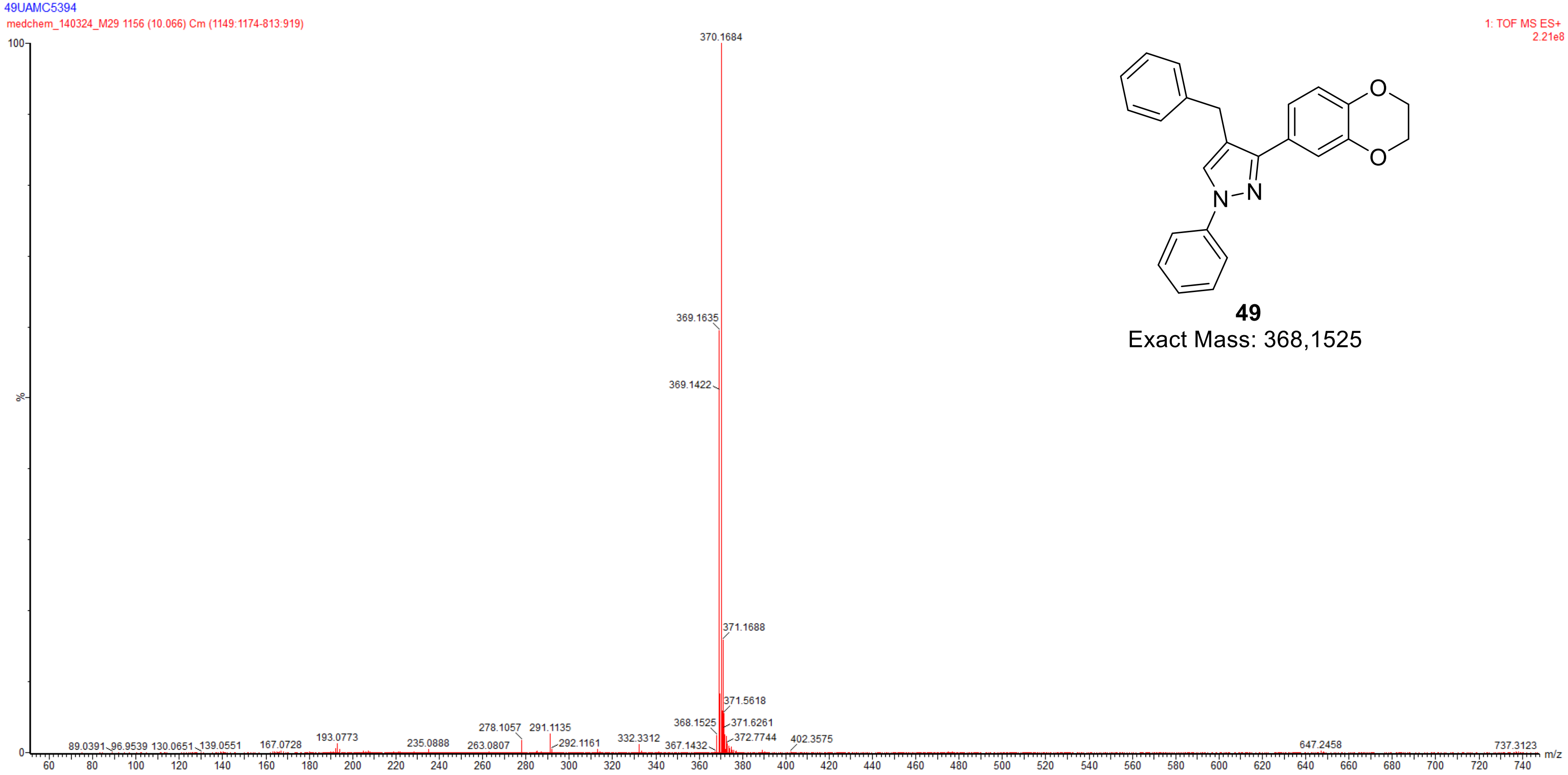

49

Exact Mass: 368,1525

2.14 *1-((3-(2,3-dihydrobenzo[b][1,4]dioxin-6-yl)-1-phenyl-1H-pyrazol-4-yl)methyl)-3-(4-fluorophenyl)urea* **(54)**

Sample 1 Vial 1:56 ID UAMC-5434 File UAMC-5434 Date 13-Apr-2023 Time 17:45:39 Description

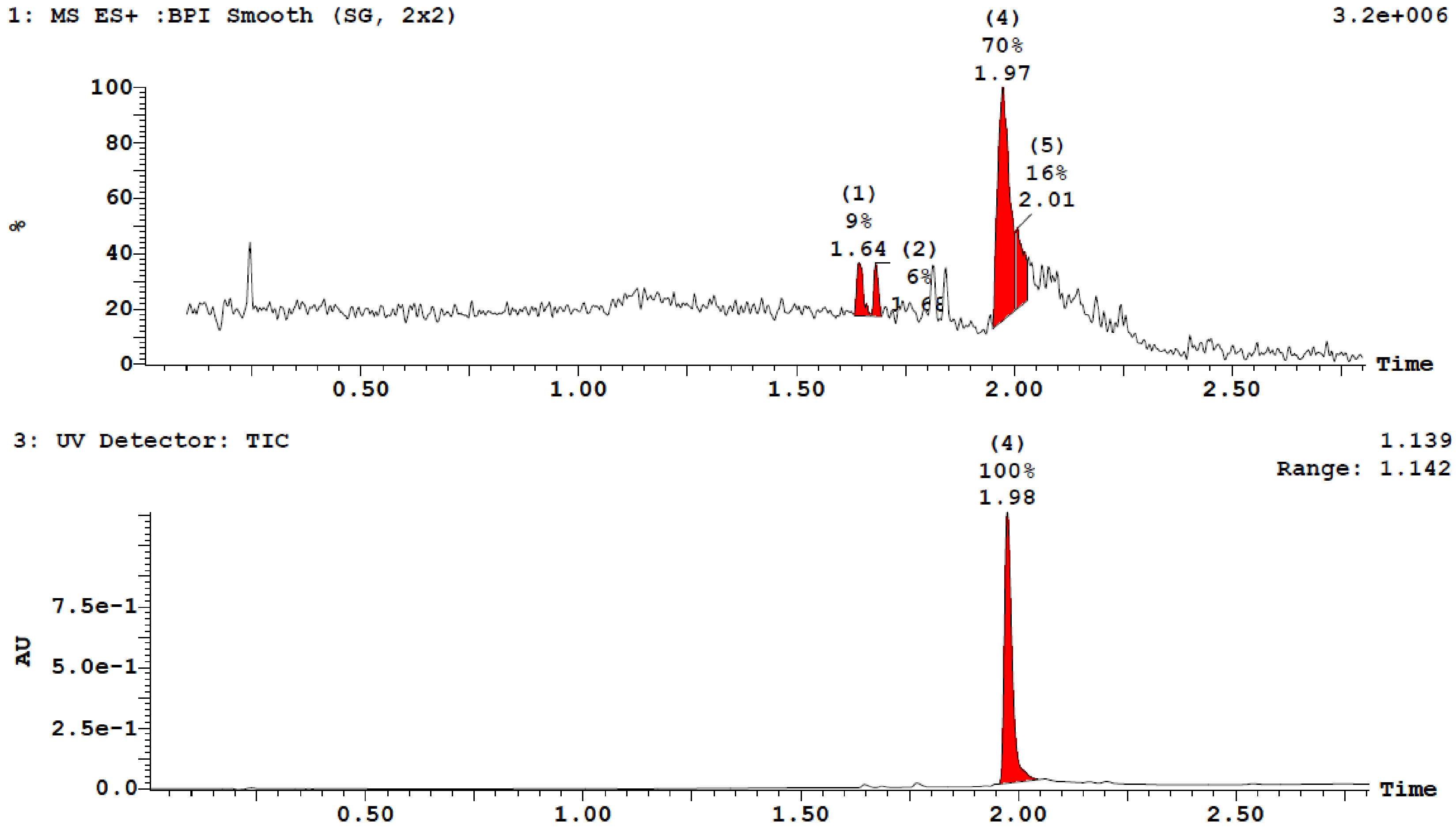

Peak Number	Compound	Time	AreaAbs	Area %Total	Width	Height	Mass Found
4		1.98	2e+004	100.00	0	1e+006	

Peak ID	Compound	Time	Mass Found
4		1.97	

4:(Time: 1.98) Combine (787:795-(774:778+826:830))

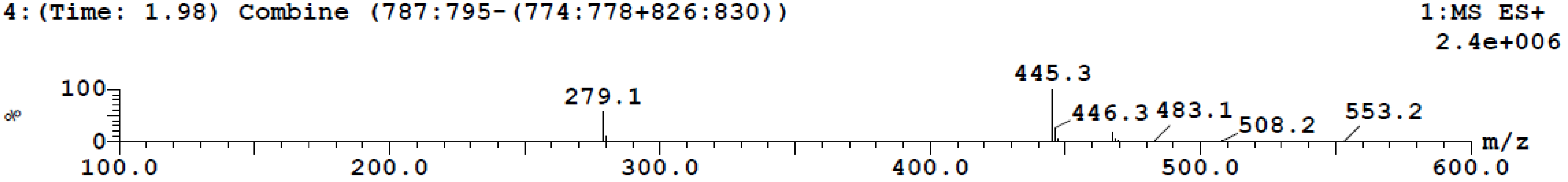

54UAMC5434

medchem_140324_M25 912 (7.941) Cm (905:919-790:823)

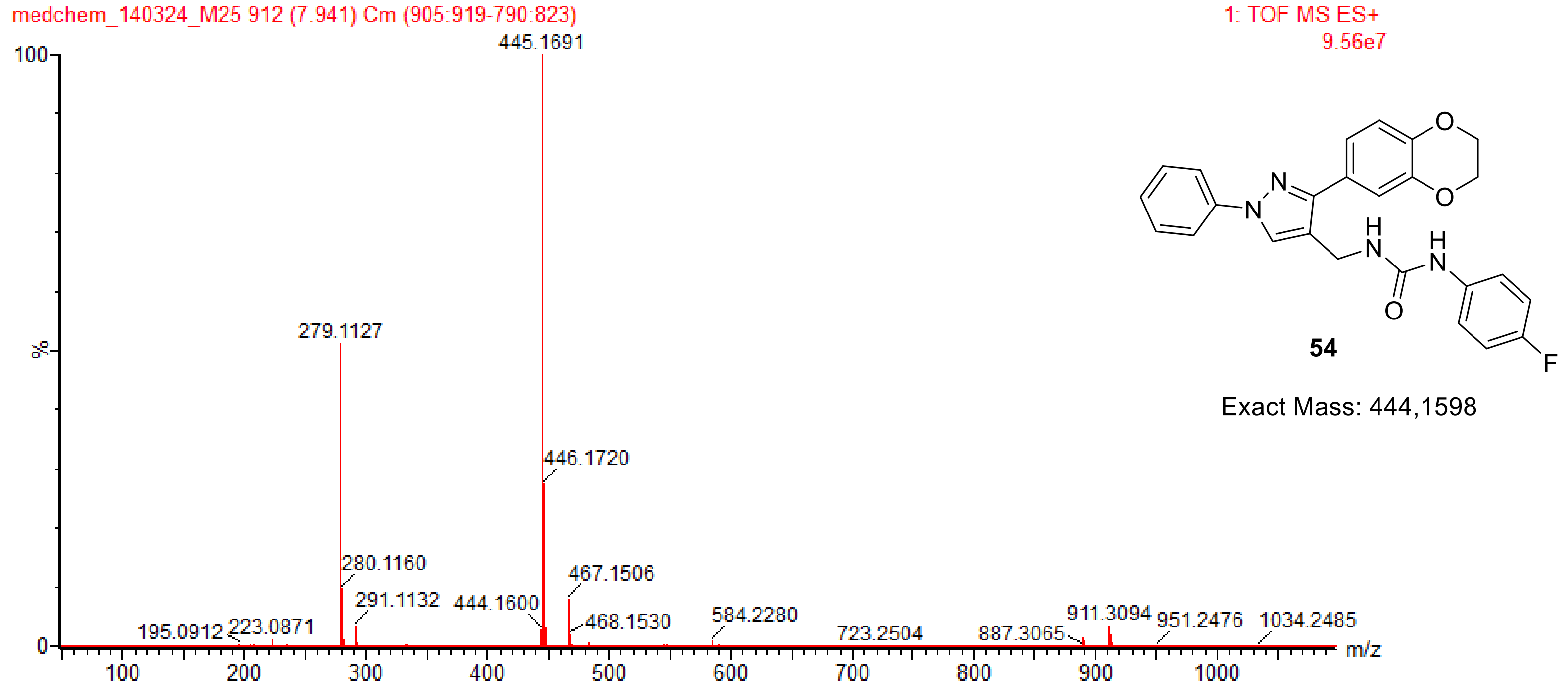

54

Exact Mass: 444,1598

2.15 *4-(((4-fluorobenzyl)oxy)methyl)-3-(4-methoxyphenyl)-1-phenyl-1H-pyrazole* **(68)**

Sample 1 Vial 2:46 ID UAMC-5419 File UAMC-5419 Date 12-Apr-2023 Time 13:59:34 Description

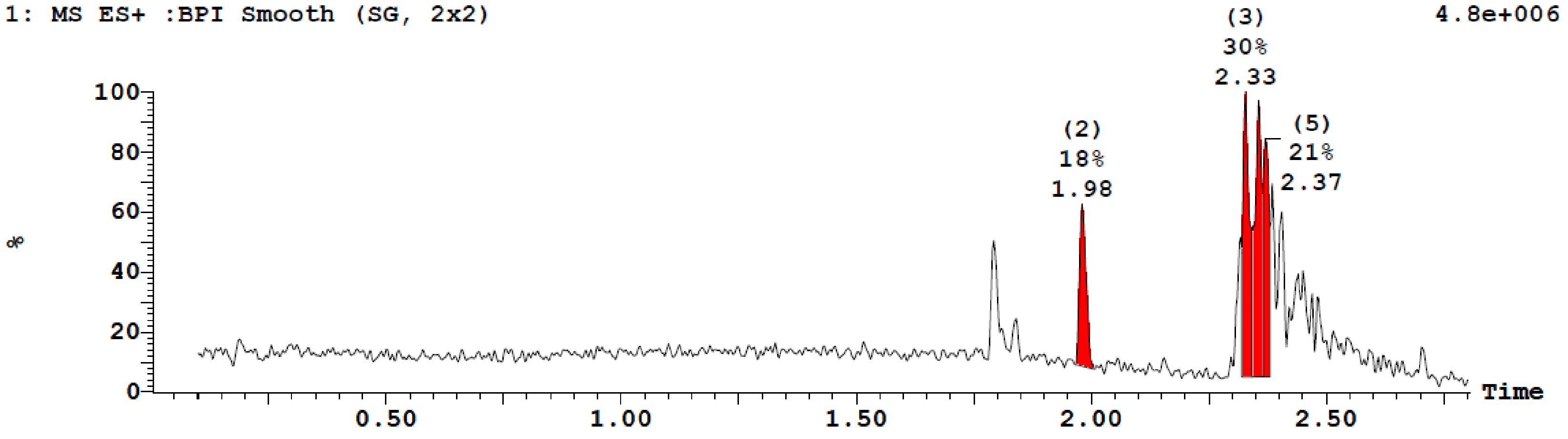

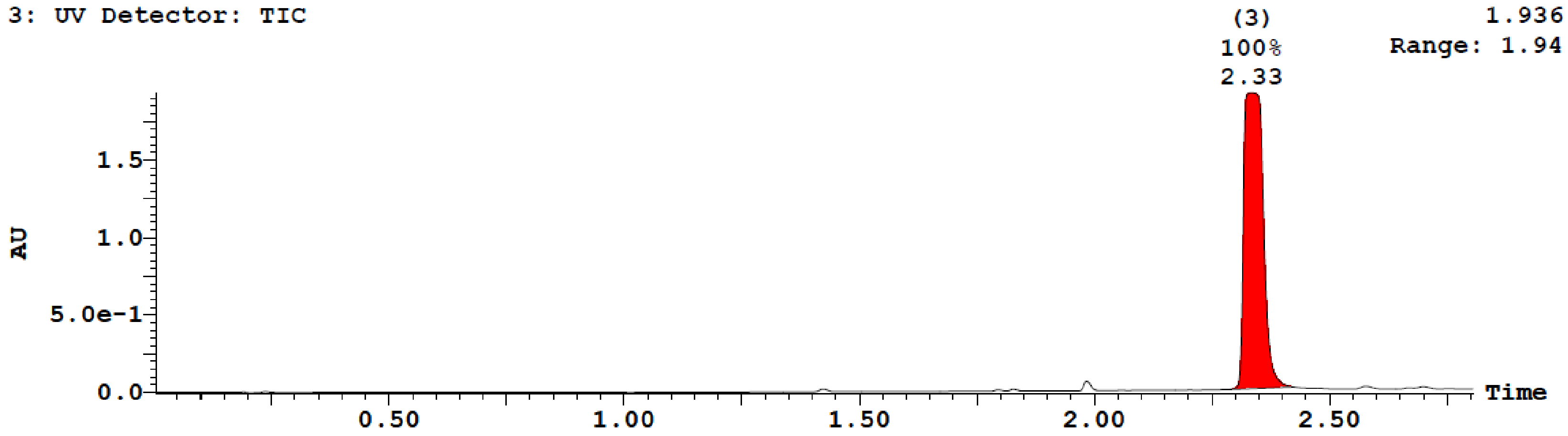

Peak Number	Compound	Time	AreaAbs	Area %Total	Width	Height	Mass Found
3		2.33	9e+004	100.00	0	2e+006	

Peak ID	Compound	Time	Mass Found
3		2.33	

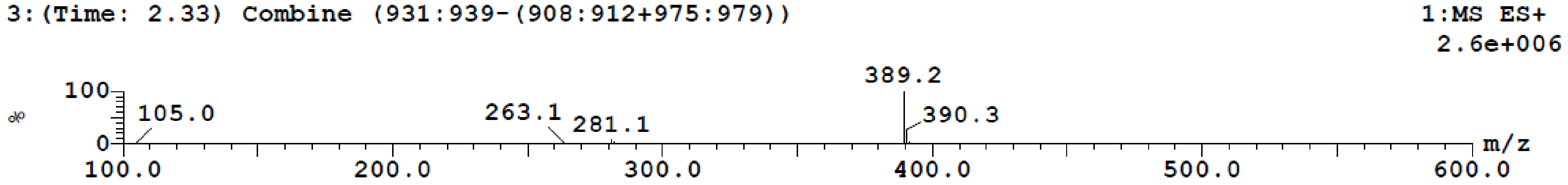

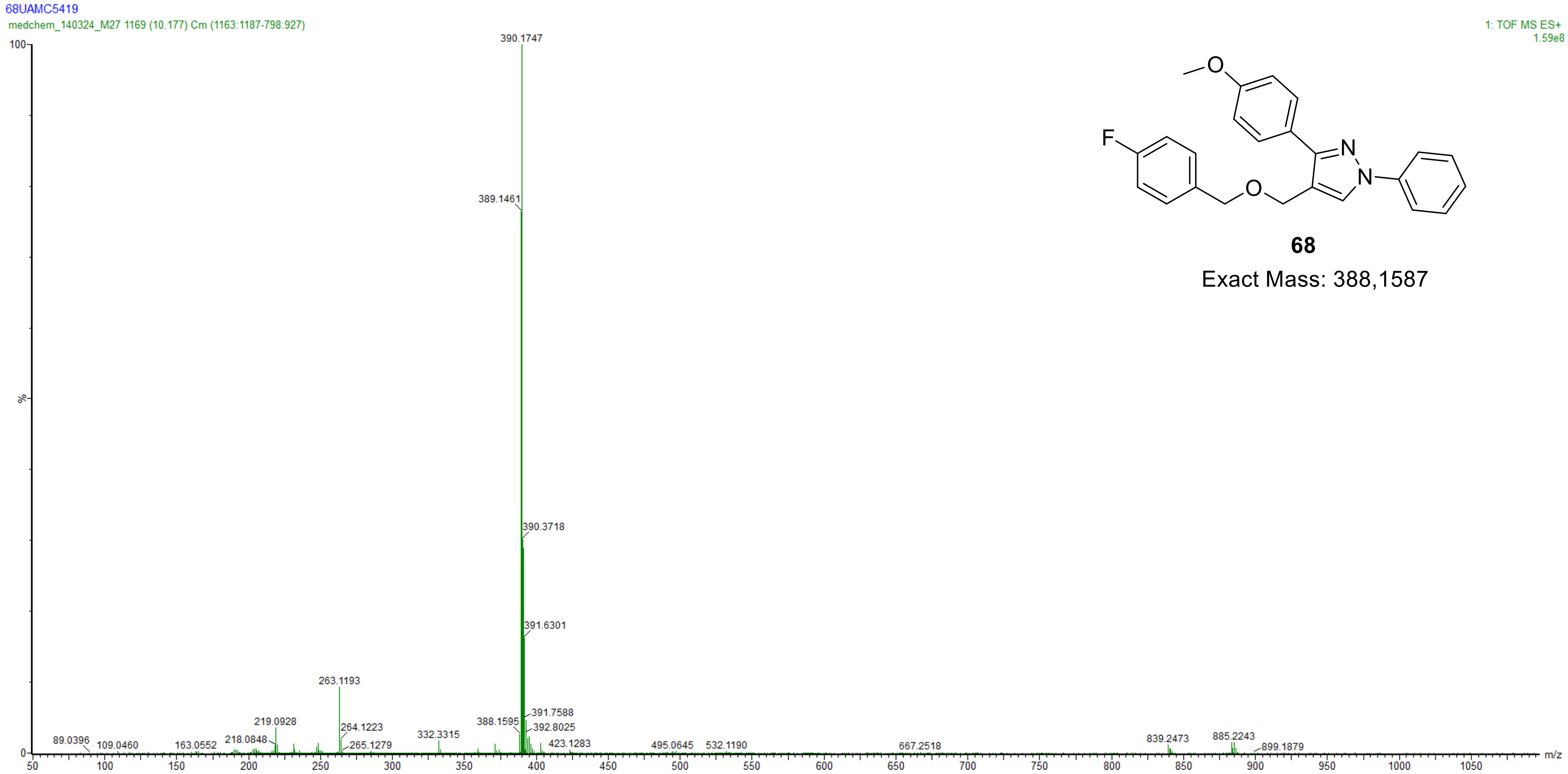

68

Exact Mass: 388,1587

2.16 *N-((3-(2,3-dihydrobenzo[b][1,4]dioxin-6-yl)-1-(pyridin-2-yl)-1H-pyrazol-4-yl)methyl)-2-(pyrrolidin-1-yl)ethan-1-amine dihydrochloride* **(69)**

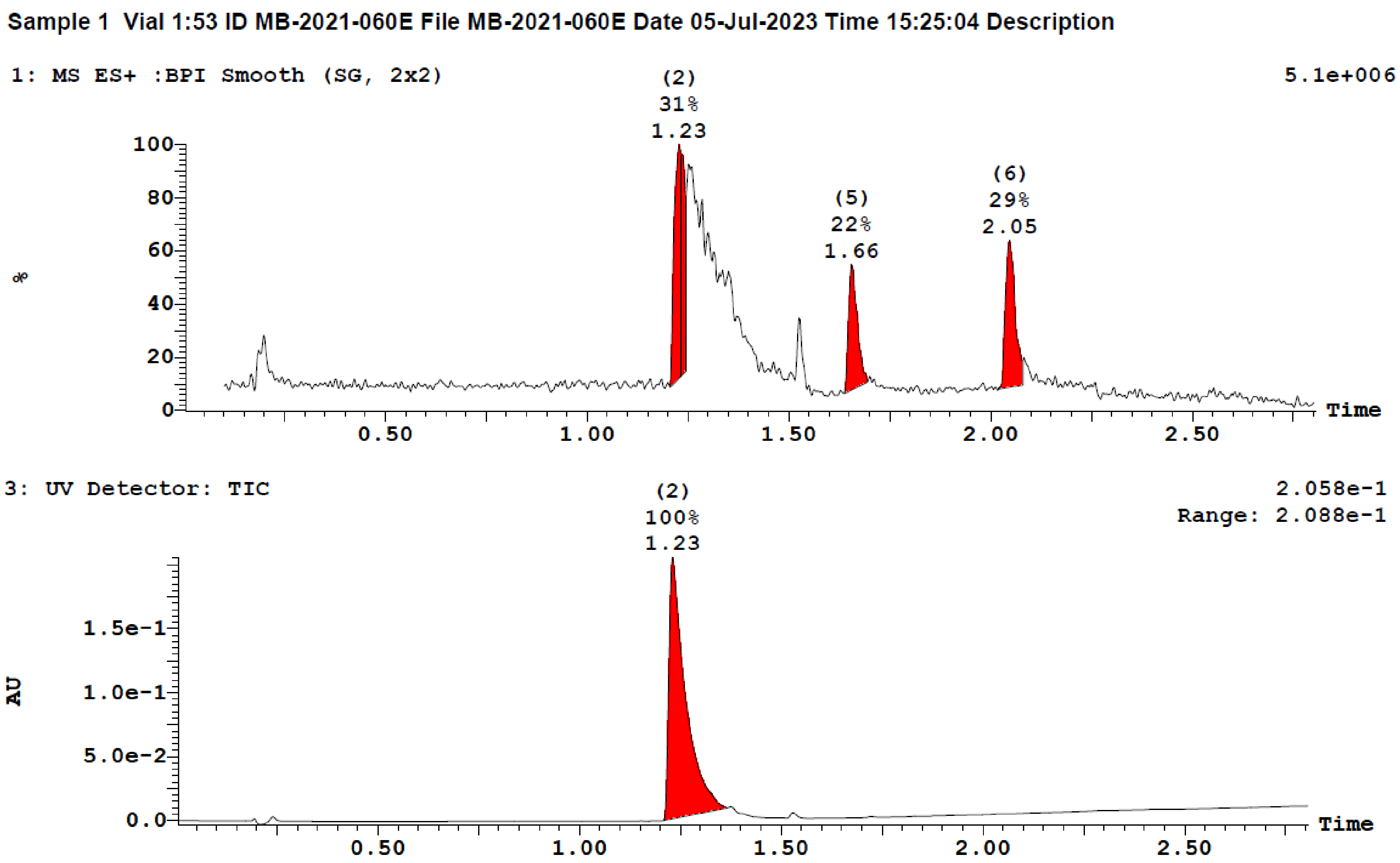

Peak Number	Compound	Time	AreaAbs	Area %Total	Width	Height	Mass Found
2		1.23	1e+004	100.00	0	2e+005	

Peak ID	Compound	Time	Mass Found
2		1.23	

2:(Time: 1.23) Combine (489:497-(475:479+552:556))

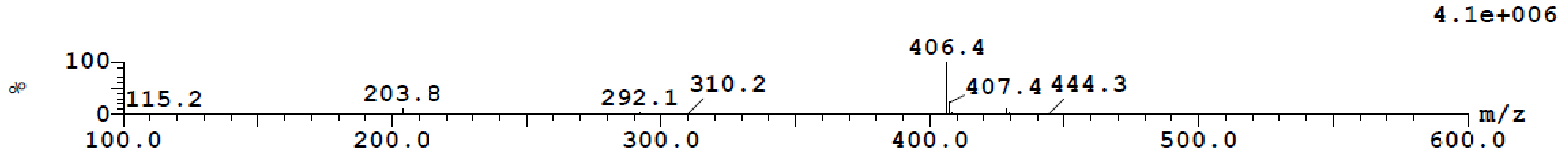

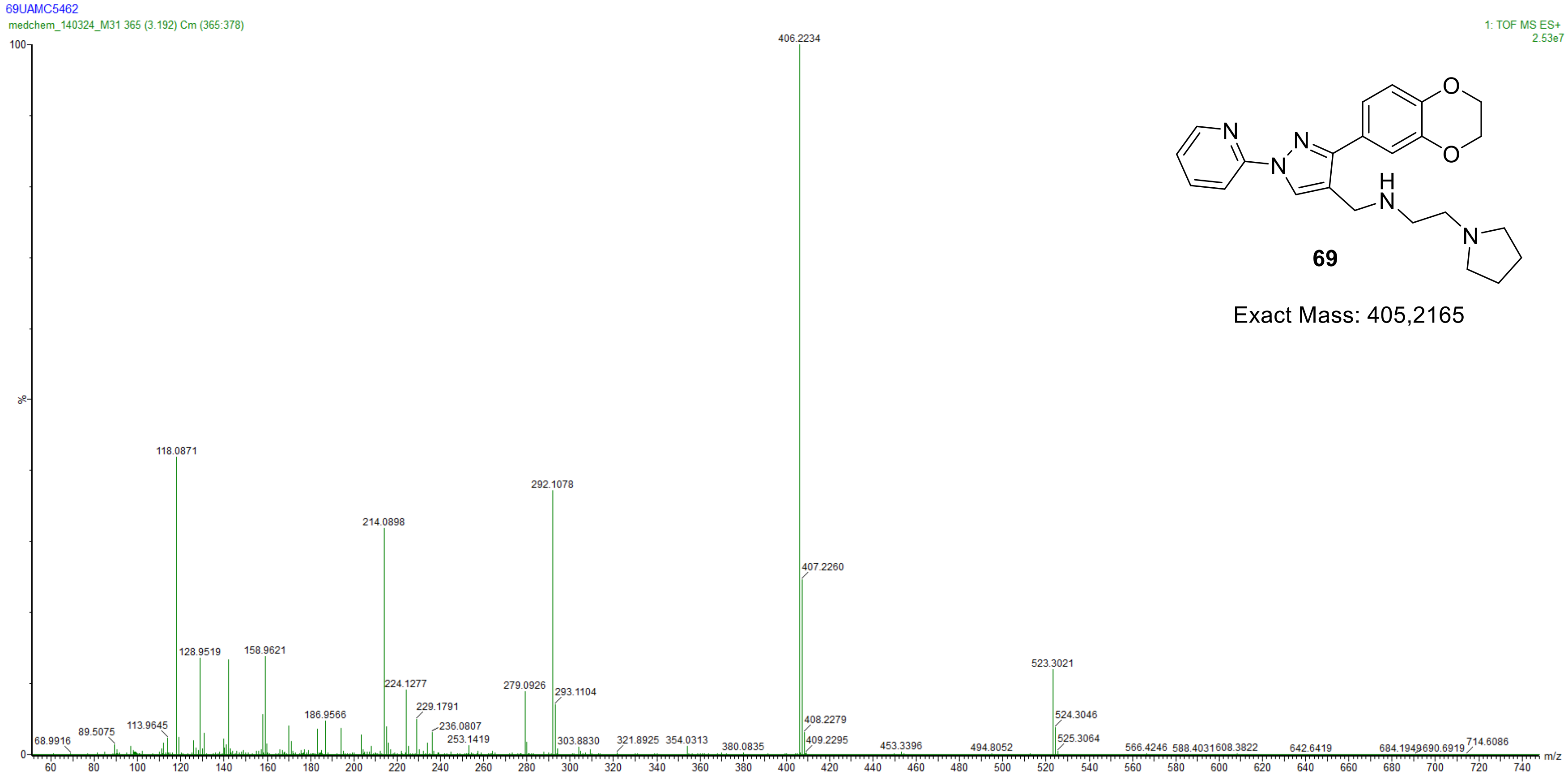

Supplement: Supplementary file 1 [file ijms-25-04693-s001.zip › ijms-2911141-supplementary.pdf]
